# Photocatalytic Synthesis of Difluorinated Glycoamino Acids and Neoglycopeptides via Hydrodifluoroacetamidation of Vinyl-C-glycosides

**DOI:** 10.1021/acs.joc.5c00030

**Published:** 2025-03-04

**Authors:** Emanuele F. Pissinati, Lívia M. S. Barreto, Till Opatz, Márcio W. Paixão

**Affiliations:** [a] Laboratory for Sustainable Organic Synthesis and Catalysis, Department of Chemistry, Federal University of São Carlos-UFSCar, 13565-905 São Carlos, São Paulo, Brazil; [b] Department of Chemistry, Johannes Gutenberg-University, Duesbergweg 10-14, 55128 Mainz, Germany

## Abstract

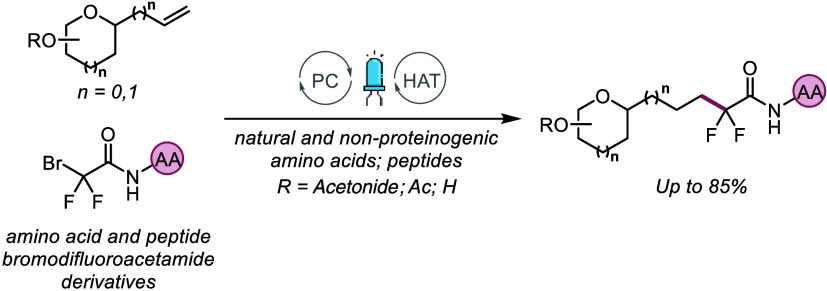

A photocatalytic approach for the synthesis of difluorinated glycoamino acids and neoglycopeptides from bromodifluoroacetamides and sugar-derived olefins is presented. This method stands out because of its simplicity, atomic economy, and mild reaction conditions, allowing compatibility with both natural and unnatural amino acids and peptides. Additionally, it demonstrates efficacy across a variety of carbohydrates, including furanoses, pyranoses, pentose, hexoses, and disaccharides, accommodating an extensive range of protecting groups, even in their deprotected forms.

Monosaccharides bearing a C=C bond have shown considerable potential in drug development programs.^1^ They also play a pivotal role as building blocks in the synthesis of natural products and biologically active molecules.^2,3^

In the field of photochemical reactions, catalytic radical techniques utilizing sugar olefins have recently emerged as greener alternatives.^4^ In this sense, glycals have been employed as nucleophilic acceptors in the stereoselective synthesis of 2-deoxyglycosides by photoacid catalysis.^5^ Moreover, these species are extensively utilized as acceptors for electrophilic radicals in cascade reactions, leveraging nitrogen-based open shell intermediates,^6^ along with carbon-centered perfluorinated radicals (Scheme 1A).^7^

**Scheme 1:**
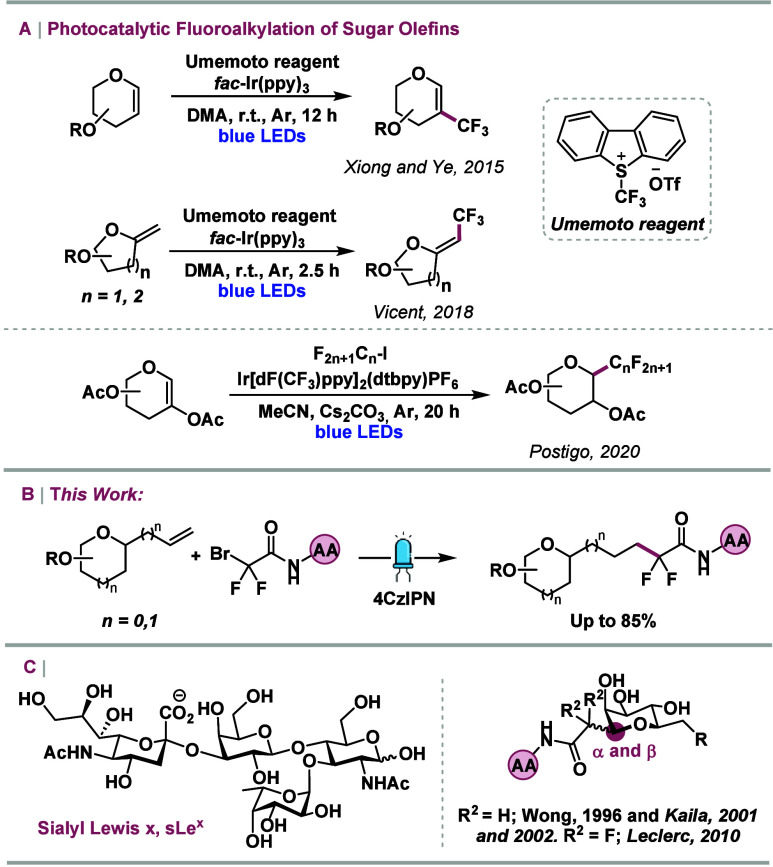
(A and B) Photocatalytic Strategies for Perfluoroalkylation of Sugar Olefins and (C) sLe^x^ and Its Mimetics

In 2021, the Molander group introduced a photochemical defluorinative alkylation involving the incorporation of difluoroacetate radicals into a single vinyl glycoside derivative.^8^ Despite its efficiency, the protocol’s reliance on less reactive radical precursors necessitates additional components and harsher conditions, which may impose limitations regarding sensitive substrates, scalability, and cost efficiency.

Building on this concept, the synthesis of fluorinated glycoamino acids and neoglycopeptides was envisioned through the addition of *gem*-difluoromethylene radicals, derived from amino acids and peptides,^9^ to sugar olefins in a straightforward and direct manner, without the necessity of any additional hydrogen-atom source in the reaction (Scheme 1B).^10^ The synthesis of neoglycopeptides represents an accessible option to mimic glycoproteins, presenting opportunities to explore new biochemical avenues crucial for drug development.^11^ Moreover, glycosylation of bioactive peptides has been recognized as a key modification for enhancing the stability of pharmaceutical peptide candidates.^12^ Glycosylation can induce significant changes in the properties of the peptide, influencing their preferred conformations and altering their chemical, physical, and biochemical properties, thereby modulating function.^12^

Similarly, the incorporation of fluorine atoms into various chemical architectures is a widely employed strategy in drug discovery and molecular interaction studies. Fluorination effectively enhances the properties of glycomimetics, improving their binding affinity and pharmacokinetic profiles.^13^

Based on previous research demonstrating C-glycopeptides as sialyl Lewis^x^ mimics,^14,15^ Leclerc and co-workers^16^ have detailed the synthesis of fluorinated C-mannopeptides and assessed their efficacy as inhibitors of E- and P-selectin (Scheme 1C). These mimetics are significant since sLe^x^ is a crucial ligand in selectin-mediated cellular interactions, pivotal for orchestrating leukocyte recruitment during the inflammatory cascade. Inappropriate or excessive recruitment of leukocytes can lead to severe tissue damage and contribute to inflammatory diseases such as stroke, reperfusion injury, psoriasis, rheumatoid arthritis, asthma, and lupus, among others.^17,18^

Given our current focus on developing photochemical methods for the selective synthesis and modification of biomolecules,^19^ efforts were directed toward developing a light-driven synthesis of novel fluorinated glycoamino acids and neoglycopeptides (Scheme 1B). Over the past decade, photocatalysis has emerged as a key synthetic strategy. In particular, incorporating biomolecules into photocatalytic processes is often seamlessly possible due to the mild, often water-compatible, and ambient-temperature conditions, critical factors for preserving the integrity of these sensitive compounds.^20^

The study began with the evaluation of the photocatalytic hydrodifluoroacetamidation of vinyl-C-glycoside **1a**, utilizing **2a** as the radical precursor. After a series of initial experiments, the optimal conditions were found to be a combination of 1 mol % 4CzIPN (1,2,3,5-tetrakis(carbazol-9-yl)-4,6-dicyanobenzene) and DMF (0.05 M) under blue light-emitting diode (LED) irradiation (450–456 nm, 3 W) at 20 °C for 10 h (Table 1). These conditions furnished the desired product **3a** in 81% isolated yield, with traces of **3a′** as a byproduct. Interestingly, when DMSO was used as a solvent, the brominated product became the major product through an ATRA (atom transfer radical addition) pathway, affording **3a′** in a 62% isolated yield with a 2.2:1 diastereomeric ratio (entry 2). The addition of tertiary organic bases, such as triethylamine and DIPEA, was further investigated as an additive in the photocatalytic protocol. However, under these reaction conditions, complete degradation of the starting materials was observed. Conversely, the addition of sodium ascorbate to the reaction medium provided the same yield as previously observed (entry 4 vs entry 1).

Furthermore, the use of an iridium-based photocatalyst did not lead to any increase in the chemical yield (entry 5). When a high-power blue lamp (40 W) served as the energy source, the hydrodifluoroacetamide product afforded a consistent 70% yield (entry 6). The reaction conducted in dry DMF exhibited a yield similar to that of the non-dry reaction (entry 7 vs entry 1). Adjustments to the reaction stoichiometry led to a decrease in the chemical yield. Finally, control experiments, reactions conducted with no light source, with no light with heating, and in the absence of the photocatalyst, produced only traces of the desired compounds (entries 9–11, respectively).

With the optimized reaction conditions established, attention turned to assessing the scope and limitations of the newly developed photochemical method. The reactivity of bromides derived from amino acids was initially investigated (Scheme 2). Gratifyingly, the photocatalytic method demonstrated broad compatibility, successfully incorporating β-amino acids (**3b**), polar amino acids prone to oxidation (**3c**–**e**), aromatic amino acids (**3f**–**l**), and the unnatural amino acid d-phenylalanine (**3g**). All products were obtained in good to excellent yields (61–85%), with the exception of the l-Trp derivative (**3i**, 37%), which was attributed to the reactive nature of the indole 2-position, previously investigated by Mitchell and co-workers.^21^ The study was then extended to bromides derived from dipeptides. In this context, glycopeptide **3j** with the peptide sequence l-Met-l-Ala-OMe was obtained in 51% isolated yield, while **3k**, containing β-Ala-l-Leu-OMe, was formed in 60% yield. The reaction scale for the formation of product **3a** was increased 15-fold (1.5 mmol), achieving an 80% yield after prolonged blue light irradiation (20 h) using one Kessil LED lamp (see the Supporting Information for more details). These results demonstrate the robustness and scalability of the protocol under the extended reaction conditions.

**Scheme 2:**
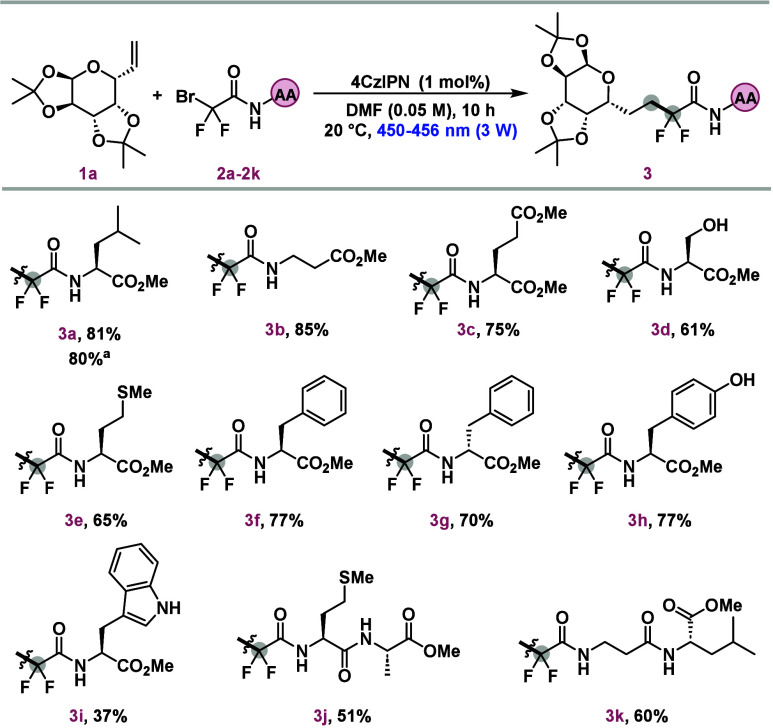
Scope of Bromides Derived from Amino Acids and Peptides Reaction performed at a 1.5 mmol scale using one Kessil blue lamp (456 nm, 40 W) at 22–25 °C for 20 h. Reaction conditions: **1a** (0.1 mmol), **2a** (0.15 mmol), 4CzIPN (1 mol %), DMF (0.05 M, 2 mL), LED lamps (450–456 nm 3 W), 20 °C, 10 h.

The focus then shifted to the synthesis of a diverse array of glycoamino acids by reacting compound **2a** with various carbohydrate-derived radical acceptors featuring double bonds in different positions. As outlined in Scheme 3, the photocatalytic methodology consistently produces satisfactory results, with yields ranging from 39% to 65%. Notably, furanose, pyranose, pentose, and hexose olefins were effectively employed, underscoring the versatility of the protocol. The method also demonstrated compatibility with a wide range of protecting groups, including acetonides (**4a** an **4f–h**), acetyl groups (**4b–e** and **4j**), and methyl ethers (**4e** and **4h**) as well as free hydroxyl groups (**4f**) and even fully deprotected carbohydrates (**4i**). Additionally, a sugar olefin derived from the disaccharide d-maltose was successfully employed, achieving a yield of 60% (**4j**). The robust and flexible protocol is well suited for various synthetic applications. The ability to maintain efficacy across different substrates and functional groups underscores the potential of this photocatalytic approach in the synthesis of complex glycoamino acids.

**Scheme 3:**
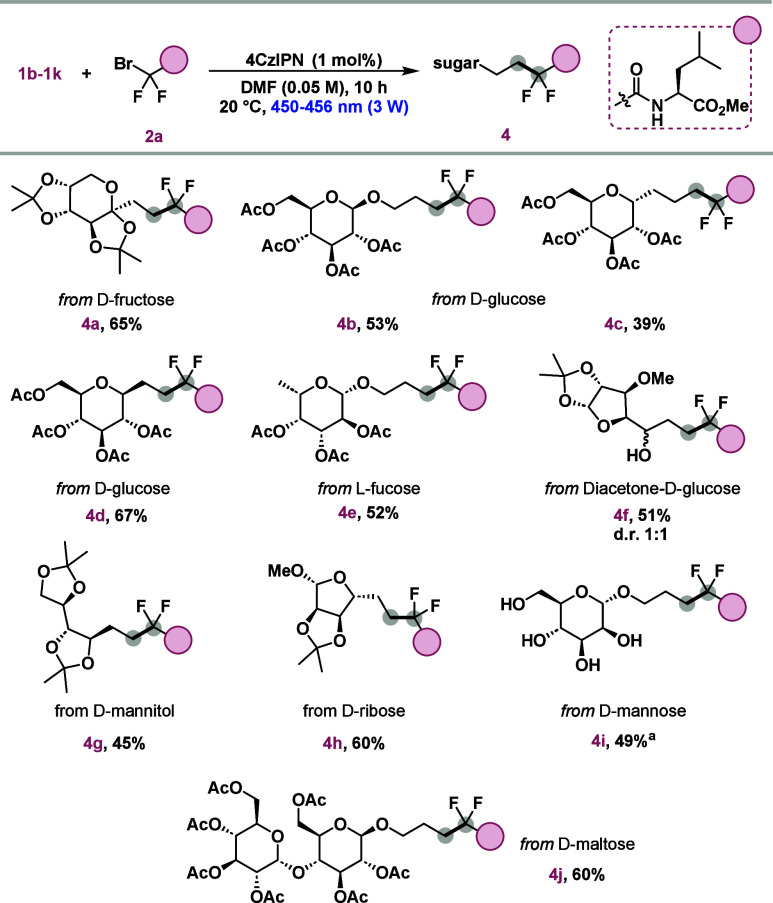
Scope of Sugar Olefins Bromide **2f** was used instead of **2a**. Reaction conditions: **1a** (0.1 mmol), **2a** (0.15 mmol), 4CzIPN (1 mol %), DMF (0.05 M, 2 mL), LED lamps (450–456 nm 3 W), 20 °C, 10 h.

To further broaden the applicability of the developed method, photocatalytic reactions were conducted using bromides with various C-terminal functionalities (Scheme 4). In this context, the difluoromethylene radical was successfully introduced into substrates with a free carboxylic acid unit (**5a**) and a primary amide group (**5b**), achieving good yields of 70% and 76%, respectively. Additionally, product **5c**, containing a single fluorine atom, was synthesized with a diastereomeric ratio (dr) of 1:1 and a yield of 70% from ethyl bromofluoroacetate.

**Scheme 4:**
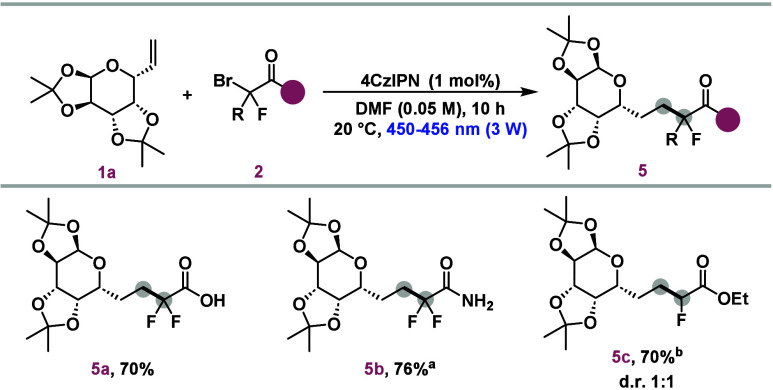
Scope of Fluorinated Sugars Using Different Bromides Irradiated with blue light for 20 h. Irradiated with blue light for 38 h. Reaction conditions: **1a** (0.1 mmol), **2** (0.15 mmol), 4CzIPN (1 mol %), DMF (0.05 M, 2 mL), LED lamps (450–456 nm 3 W), 20 °C, 10 h.

To gain a deeper understanding of the reaction, mechanistic studies were conducted (Scheme 5). First, deuteration experiments were performed using deuterated dimethylformamide as the reaction solvent to confirm the source of the hydrogen atom in the final elementary step of the reaction pathway (Scheme 5A). Under these conditions, deuterated compound **6** was isolated in only 12% chemical yield. The lower yield and significant degradation of **1a** or the radical intermediates, along with the simultaneous formation of the brominated product (**3a′**), highlight the necessity for a specific hydrogen donor, such as nondeuterated DMF (entry 2, Table 1), to effectively control product selectivity between the two potential reaction pathways. Radical trapping studies were also performed under the standard conditions (Scheme 5B). The addition of 2 equiv of 2,2,6,6-tetramethyl-1-piperidinyloxy (TEMPO) inhibited product formation, leading to the detection by HRMS of difluoroalkylated and dimethylformamide TEMPO adducts **7** and **8**, respectively.^22^ Furthermore, to determine whether the difluoromethylene radical is formed via an XAT process, the reaction with ethyl bromoacetate (**9**; P/P^*•*–^ = −1.43 V vs SCE)^23^ was performed, which lacks the potential to be directly reduced by 4CzIPN (PC^*•+*^/PC* = −1.18 V vs SCE)^25^ (Scheme 5C). No product formation was observed, ruling out the possibility of radical initiation in the reaction medium during this process. Additionally, a light on/off experiment, as illustrated in Scheme 5D, demonstrates that the reaction does not proceed in the dark, confirming its dependence on light, but does not rule out fast radical chains.^24^

**Table 1:**
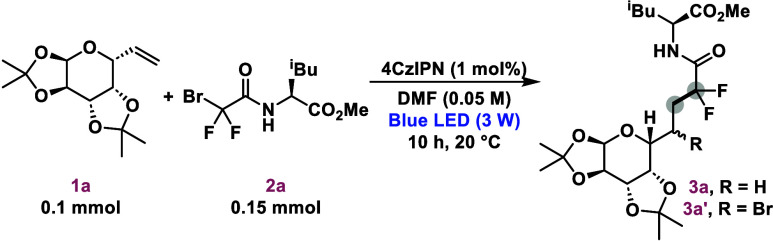
Optimization Study^a^

entry	deviation from the standard condition	yield of **3a** (%)	yield^b^ of **3a′** (%) [dr]
1	none	81	trace
2	DMSO as solvent, 20 h	trace	62 [2.2:1]
3^c^	Et_3_N or DIPEA as additive	ND	ND
4^c^	sodium ascorbate as additive	80	trace
5	Ir[dF(CF_3_)_2_(dtbpy)PF_6_] as photocatalyst	74	trace
6	blue LED (40 W), 35 °C, 20 h	70	trace
7	dry DMF (0.05 M)	80	trace
8^d^	inverted stoichiometry	57	trace
9^e^	without a blue LED, at 60 °C	trace	trace
10^e^	in the dark	trace	trace
11^e^	without a photocatalyst	trace	trace

a Reaction conditions: **1a** (0.1 mmol), **2a** (0.15 mmol), 4CzIPN (1 mol %), DMF (0.05 M, 2 mL), LED lamps (450–456 nm, 3 W), 20 °C, 10 h.

b Isolated yield.

c With 0.15 mmol of additive.

d **1a** (0.15 mmol), **2a** (0.10 mmol).

e ^19^F NMR analysis (crude).

**Scheme 5:**
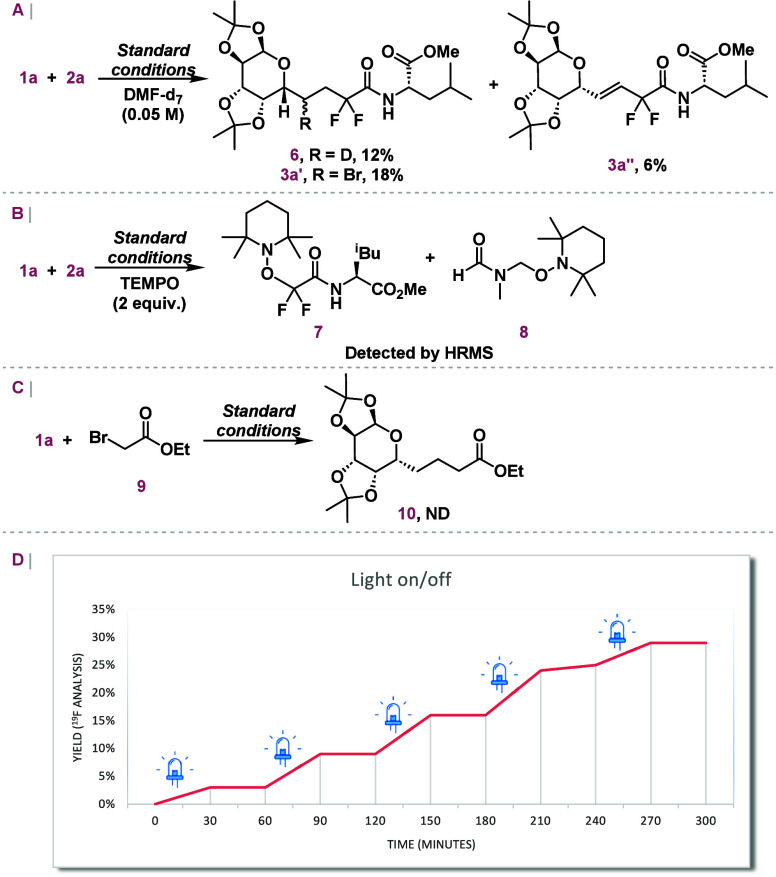
Mechanistic Studies (A) Deuteration experiment. (B) Radical trapping. (C) Evaluation of the XAT process. Standard conditions for reactions A–C: **1a** (0.1 mmol), **2a** or **9** (0.15 mmol), 4CzIPN (1 mol %), DMF-*d*_7_ or DMF (0.05 M, 2 mL), LED lamps (450–456 nm, 3 W), 20 °C, 10 h. (D) Light on/off experiment, in which standard conditions were employed. Aliquots were collected every 30 min to monitor the reaction. PhCF_3_ was used as the internal ^19^F NMR standard for quantification. Standard conditions: **1a** (0.1 mmol), **2a** (0.15 mmol), 4CzIPN (1 mol %), PhCF_3_ (1 equiv), DMF (0.05 M, 2 mL), LED lamps (450–456 nm, 3 W), 20 °C.

Drawing on the results of mechanistic studies, a mechanism is proposed based on principles of related photoredox-catalyzed radical reactions (Figure 1). The blue light photoexcitation of 4CzIPN generates 4CzIPN*, which reduces bromide **2** (−1.04 V vs SCE)^26^ to generate difluoroalkyl radical **I**.^27^ This radical then adds to olefin **1**, forming radical intermediate **II** that subsequently abstracts a hydrogen atom from the α-amino position of DMF, yielding final product **3** and generating species **III**.^28^ It is hypothesized that species **III** either decomposes or reacts with a nucleophilic species present in the reaction medium.

**Figure 1:**
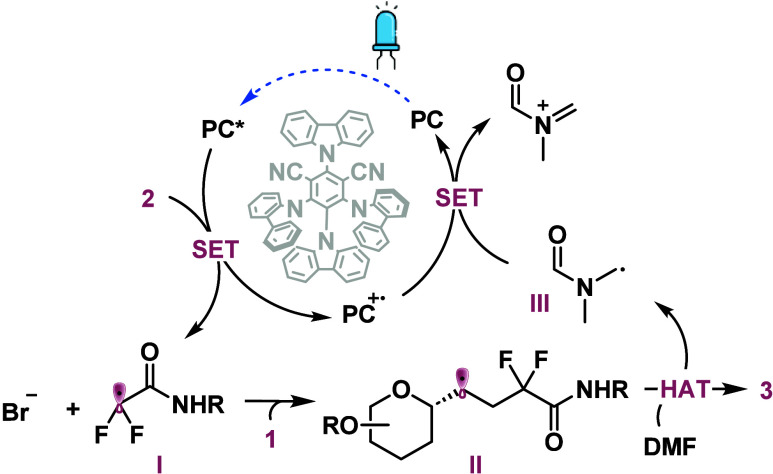
Proposed reaction mechanism.

In summary, a mild and versatile photocatalytic approach for synthesizing difluoroglyco compounds from simple, unactivated glyco-olefins is presented. The method employs a photoredox strategy combined with hydrogen-atom donors to reliably and effectively produce a broad range of glycoamino acids and glycopeptides in satisfactory yields. This approach represents as a valuable tool for advancing the development of new glyco-chemical entities.

## Data Availability

The data underlying this study are available in the published article and its Supporting Information.

## Supporting Information Available

Experimental procedures, characterization data, and spectra (PDF)
FAIR data, including the primary NMR FID files, for compounds **3a–k**, **4a–j**, **5a–c**, and **6** (ZIP)
