# Supplementary material for: Photocatalytic Synthesis of Difluorinated Glycoamino Acids and Neoglycopeptides via Hydrodifluoroacetamidation of Vinyl-C-glycosides
Source: J Org Chem. 2025 Mar 4;90(10):3733–8. doi: 10.1021/acs.joc.5c00030 (PMC11915378; doi:10.1021/acs.joc.5c00030)
Supplement: Supplementary file 1 — jo5c00030_si_001.pdf [file jo5c00030_si_001.pdf]

Supporting Information

Photocatalytic Synthesis of Difluorinated Glycoamino Acids and Neoglycopeptides via Hydrodifluoroacetamidation of Vinyl-C-glycosides

Emanuele F. Pissinati,[a,b] Lívia M. S. Barreto,[a] Till Opatz,[b,]* and Márcio W. Paixão[a,]*

[a] Laboratory for Sustainable Organic Synthesis and Catalysis, Department of Chemistry, Federal University of São Carlos, UFSCar, São Paulo 13565-905, Brazil. E-mail: mwpaixao@ufscar.br

[b] Department of Chemistry, Johannes Gutenberg-University, Duesbergweg 10-14, 55128, Mainz, Germany. E-mail:opatz@uni-mainz.de

Table of Contents

1. **General Information S2**
2. **Optimization Procedure: S2**
3. **Unsuccessful substrates: S3**
4. **General procedure 1: S3**
5. **Characterization data of compounds 3a-3l. S4**
6. **Characterization data of compounds 4a-4j S10**
7. **Characterization data of compounds 5a-5c S16**
8. **General procedure 2: Synthesis of sugar olefins 1a-1k S18**
9. **General procedure 3: Synthesis of 1-bromo-1,1-difluoroacetamides 2a-2i and 2m... S19**
10. **General procedure 4: Synthesis of 1-bromo-1,1-difluoroacetamides 2j-2k. S21**
11. **Characterization data of compounds 2j-2k. S21**
12. **Scale-Up Reaction S22**
13. **Mechanistical Studies S23**

Deuteration Experiment S23

Radical Trapping S26

UV-Vis Analysis S28

Light On/Off Experiment S28

14. **Characterization Spectra of compounds 3a-3 .... S30**
15. **Characterization Spectra of compounds 4a-4j S46**
16. **Characterization Spectra of compounds 5a-5c S61**
17. **Characterization Spectra for 1-Bromo-1,1-Difluoroacetamides 2b-d, 2h and 2j-k..... S66**

References........ S73

1. General Information

All solvents were dried and distilled before use. Reagents were purchased from Sigma-Aldrich, Oakwood Chemicals, BLD Pharm, TCI, Ambeed, Fischer Sci., and used as received. Glassware was dried in an oven or with a flame and under vacuum, being subsequently cooled under an inert atmosphere. Column flash chromatography was performed using silica gel 60 (230−400 mesh). Analytical thin-layer chromatography (TLC) was performed using silica gel aluminum sheets with a fluorescence indicator. Compounds were visualized on TLC by UV-light, or even sprayed with either $KMnO_4$, vanillin/H_2SO_4 or thymol/H_2SO_4 reagents. Reaction chemical yields refer to chromatographically and spectroscopically pure compounds unless otherwise noted. ^{1}H and ^{13}C NMR spectra were recorded on Bruker NMR spectrometers (400 or 600 MHz for ^{1}H, 100 or 150 MHz for ^{13}C and 377 MHz for ^{19}F). Chemical shifts (δ) are reported in parts per million relatively to the residual solvent signals[1], coupling constants (J) are reported in Hertz. The following abbreviations indicate the multiplicity of ^{1}H signals: (s), singlet; (d), doublet; (t), triplet; (q), quartet; (sept), septet; (m), multiplet; and combinations thereof. Accurate mass determinations were made on a G6545A Q-ToF (Agilent GmbH, Waldbronn, Germany) with electrospray ionization (ESI). Sample inlet was via a 1260 Infinity II HPLC system (Agilent GmbH, Waldbronn, Germany) with G7111B 1260 Quaternary Pump, G7129A 1260 Vial sampler, and G7116A 1260 Multicolumn Thermostat. Mass calibration was performed on the day of measurement using an external standard. The mass accuracy of the measurement results is better than 5 ppm. The specific rotation analysis of the enantiomerically pure final products was performed using a Perkin-Elmer Model 241 polarimeter, with the following parameters: detector wavelength (λ) = 589.44 nm, temperature = 20 °C, solvent = acetone, concentration = 0.2 g/100 mL, and optical path length = 100 mm.

2. Optimization Procedure:

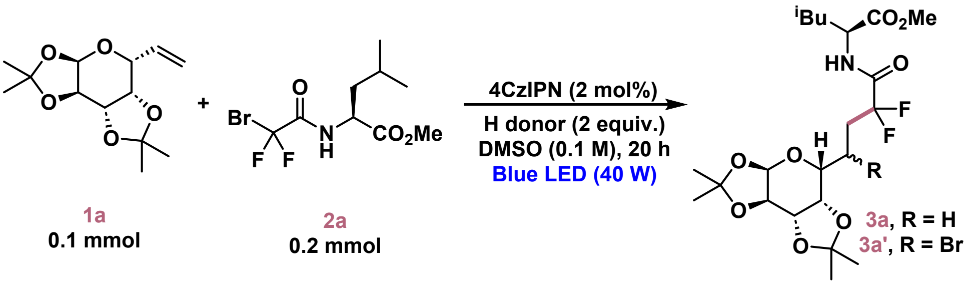

An oven-dried 10 mL Schlenk tube was charged with olefin (0.1 mmol, 1.0 equiv.), bromide (0.15 mmol, 1.5 equiv.), 4CzIPN (1 mol%) and a magnetic stir bar. After the addition of solvent (1.0 mL), the tube was closed with a septum and the mixture was degassed via a freeze–pump–thaw method. The reaction tube was placed in front of a Blue LED lamp (Kessil ~456 nm – 3 cm distance) and the reaction mixture was stirred for 20 h. Once the time has passed, the reaction mixture was diluted with EtOAc and washed with H2O (2 x 25 mL), brine (2 x 25 mL), dried over anhydrous Na2SO4 and concentrated under reduced pressure. Purification by flash column chromatography (EtOAc/hexanes) provided the desired product.

3. Unsuccessful substrates:

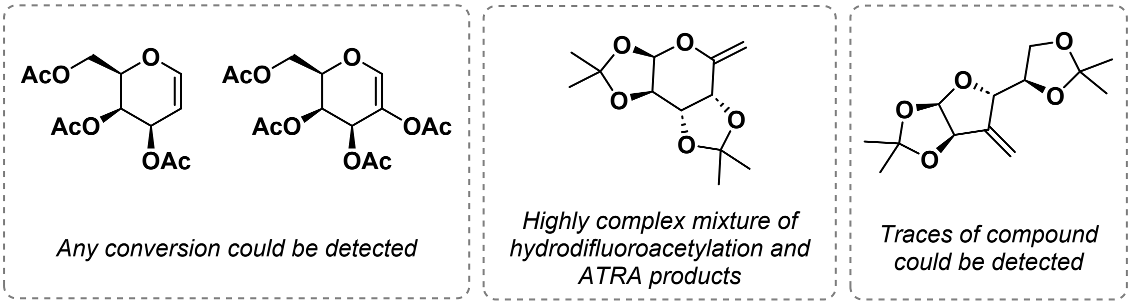

4. General procedure 1:

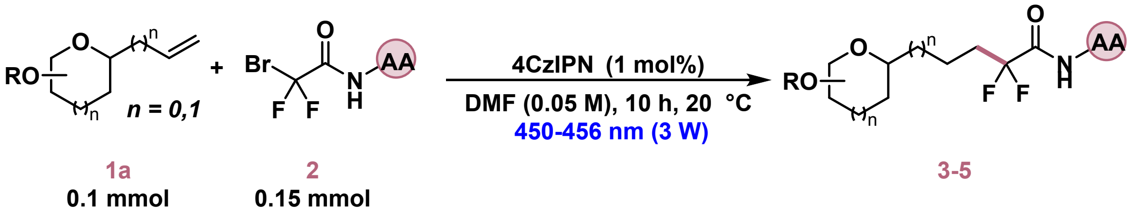

A 5 mL crimp cap reaction vial was charged with olefin (0.1 mmol, 1.0 equiv.), bromide (0.15 mmol, 1.5 equiv.), 4CzIPN (1 mol%) and a magnetic stir bar. After the addition of DMF (0.05 M), the crimp vial was capped and bubbled with Argon for 10 min. Then, the vial was placed in a thermostatic cooling block (20 °C) and irradiated through the panel bottom side of the vial by a 450-456 nm LED (3 W). Once the time has passed, the reaction mixture was diluted with EtOAc and washed with H_2O (2 x 25 mL), brine (2 x 25 mL), dried over anhydrous Na_2SO_4 and concentrated under reduced pressure. Purification by flash column chromatography (EtOAc/hexanes) provided the desired product.

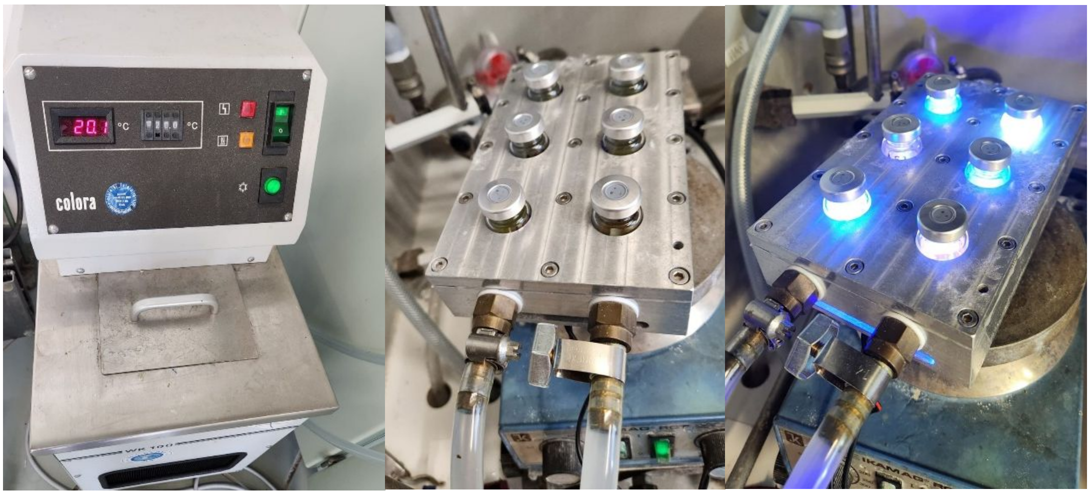

Figure S1. Representative pictures of reaction setup.

(This reactor setup is a custom-made device (University of Regensburg workshop) and is not a commercially available product).

5. Characterization data of compounds 3a-3l.

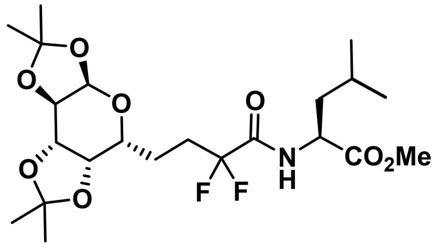

Methyl (2,2-difluoro-4-((3aR,5R,5aS,8aS,8bR)-2,2,7,7-tetramethyltetrahydro-5H-bis([1,3]dioxolo)[4,5-b:4',5'-d]pyran-5-yl)butanoyl)-L-leucinate **(3a)** was synthesized according to the general procedure 1: Flash column chromatography purification 7-12% of EtOAc in hexanes afforded the title as a pale yellow oil in 81**% (38.7 mg).** **^{1}H NMR (400 MHz, Acetone-*d*$_6$)** δ 8.11 (d, *J* = 8.3 Hz, 1H), 5.45 (d, *J* = 5.1 Hz, 1H), 4.61 (dd, *J* = 7.9, 2.4 Hz, 1H), 4.54 (ddd, *J* = 10.6, 8.3, 4.4 Hz, 1H), 4.32 (dd, *J* = 5.1, 2.3 Hz, 1H), 4.18 (dd, *J* = 7.9, 1.9 Hz, 1H), 3.77 (ddd, *J* = 9.3, 4.2, 1.8 Hz, 1H), 3.69 (s, 3H), 2.37 – 2.20 (m, 1H), 2.19 – 2.07 (m, 1H), 1.85 – 1.61 (m, 5H), 1.47 (s, 3H), 1.37 (s, 3H), 1.31 (d, *J* = 3.5 Hz, 6H), 0.95 – 0.91 (m, 6H). **^{13}C NMR (101 MHz, Acetone-*d*$_6$)** δ 172.8, 164.9 (t, *J* = 29.4 Hz), 119.2 (t, *J* = 250.7 Hz), 109.5, 108.9, 97.3, 73.4, 71.8, 71.3, 67.5, 52.5, 51.5, 40.5, 31.7 (t, *J* = 23.7 Hz), 26.4, 26.2, 25.4, 25.1, 24.6, 23.5 (t, *J* = 4.7 Hz), 23.2, 21.5. **^{19}F NMR (376 MHz, Acetone-*d*$_6$)** δ -106.36 (d, *J* = 250.6 Hz, 1F), -107.68 (d, *J* = 251.3 Hz, 1F). **HRMS (ESI)** *m/z:* $[M+Na]^+$ Calcd. for $C_{22}H_{35}F_2NO_8Na^+$ 502.2223; Found: 502.2222. **$[\alpha]_D^{20.0}$** = −61.6 (*c* = 0.2 in acetone).

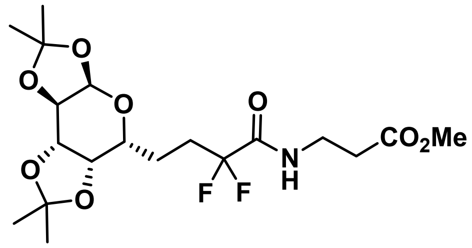

Methyl 3-(2,2-difluoro-4-((3aR,5R,5aS,8aS,8bR)-2,2,7,7-tetramethyltetrahydro-5H-bis([1,3]dioxolo)[4,5-b:4',5'-d]pyran-5-yl)butanamido)propanoate **(3b)** was synthesized according to the general procedure 1: Flash column chromatography purification 10-15% of EtOAc in hexanes afforded the title as a pale yellow oil in 85**% (37.09 mg).** **^{1}H NMR (400 MHz, Acetone-*d*$_6$)** δ 7.95 (s, 1H), 5.45 (d, *J* = 5.1 Hz, 1H), 4.60 (dd, *J* = 7.9, 2.4 Hz, 1H), 4.32 (dd, *J* = 5.1, 2.4 Hz, 1H), 4.18 (dd, *J* = 7.9, 1.9 Hz, 1H), 3.76 (ddd, *J* = 9.3, 4.3, 1.8 Hz, 1H), 3.64 (s, 3H), 3.55 (q, *J* = 6.5 Hz, 2H), 2.61 (t, *J* = 6.8 Hz, 2H), 2.32 – 2.18 (m, 1H), 2.18 – 1.97 (m, 1H), 1.81 – 1.70 (m, 1H), 1.68 – 1.59 (m, 1H), 1.46 (s, 3H), 1.37 (s, 3H), 1.31 (d, *J* = 2.5 Hz, 6H). **^{13}C NMR (101 MHz, Acetone-*d*$_6$)** δ 172.4, 164.7 (t, *J* = 28.9 Hz), 119.3 (t, *J* = 250.7 Hz), 109.5, 108.9, 97.3, 73.4, 71.8, 71.3, 67.5, 51.8, 36.0, 34.0, 31.5 (t, *J* = 23.8 Hz), 26.4, 26.2, 25.1, 24.6, 23.4 (t, *J* = 4.7 Hz). **^{19}F NMR (376 MHz, Acetone-*d*$_6$)** δ -106.54 (d, *J* = 251.6 Hz), -107.56 (d, *J* = 251.6 Hz). **HRMS (ESI)** *m/z:* [M+Na]$^+$ Calcd. for $C_{19}H_{29}F_2NO_8Na^+$ 460.1753; Found: 460.1750. **[α]$_D$$^{20.0}$** = −31.6 (*c* = 0.2 in acetone).

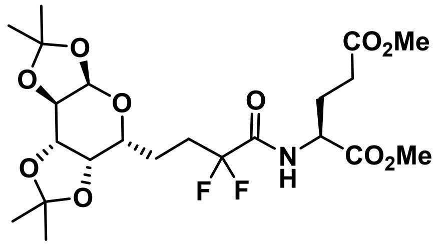

Dimethyl (2,2-difluoro-4-((3aR,5R,5aS,8aS,8bR)-2,2,7,7-tetramethyltetrahydro-5H-bis([1,3]dioxolo)[4,5-b:4',5'-d]pyran-5-yl)butanoyl)-L-glutamate **(3c)** was synthesized according to the general procedure 1: Flash column chromatography purification 15-25% of EtOAc in hexanes afforded the title as a pale yellow oil in 75**% (38.14 mg).** **^{1}H NMR (400 MHz, Acetone-*d*$_6$)** δ 8.16 (d, *J* = 8.0 Hz, 1H), 5.45 (d, *J* = 5.1 Hz, 1H), 4.60 (dd, *J* = 7.9, 2.4 Hz, 1H), 4.57 – 4.50 (m, 1H), 4.32 (dd, *J* = 5.1, 2.4 Hz, 1H), 4.18 (dd, *J* = 7.9, 1.9 Hz, 1H), 3.77 (ddd, *J* = 9.4, 4.3, 1.8 Hz, 1H), 3.71 (s, 3H), 3.63 (s, 3H), 2.51 – 2.43 (m, 2H), 2.36 – 2.20 (m, 3H), 2.16 – 2.07 (m, 1H), 1.85 – 1.75 (m, 1H), 1.72 – 1.63 (m, 1H), 1.47 (s, 3H), 1.37 (s, 3H), 1.31 (d, *J* = 3.5 Hz, 6H). **^{13}C NMR (101 MHz, Acetone-*d*$_6$)** δ 173.5, 171.9, 165.1 (t, *J* = 29.6 Hz), 119.2 (t, *J* = 250.9 Hz), 109.5, 108.9, 97.3, 73.4, 71.8, 71.3, 67.5, 52.6, 51.8, 31.6 (t, *J* = 23.6 Hz), 30.6, 26.8, 26.4, 26.3, 25.1, 24.6, 23.4 (t, *J* = 4.7 Hz). **^{19}F NMR (376 MHz, Acetone-*d*$_6$)** δ -106.35 (d, *J* = 251.6 Hz, 1F), -107.69 (d, *J* = 251.7 Hz, 1F). **HRMS (ESI)** *m/z:*

[M+Na]$^+$ Calcd. for $C_{22}H_{33}F_2NO_{10}Na^+$ 532.1965; Found: 532.1967. **$[\alpha]_D^{20.0}$** = −48.9 (*c* = 0.2 in acetone).

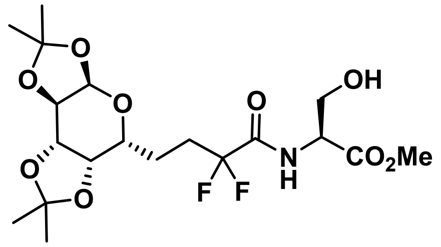

Methyl (2,2-difluoro-4-((3aR,5R,5aS,8aS,8bR)-2,2,7,7-tetramethyltetrahydro-5H-bis([1,3]dioxolo)[4,5-b:4',5'-d]pyran-5-yl)butanoyl)-L-serinate **(3d)** was synthesized according to the general procedure 1: Flash column chromatography isocratic purification 15% EtOAc/35% DCM/50% hexanes afforded the title as a colorless oil in 61**% (27.87 mg).** **^{1}H NMR (400 MHz, Acetone-*d*$_6$)** δ 7.84 – 7.71 (m, 1H), 5.46 (d, *J* = 5.1 Hz, 1H), 4.61 (dd, *J* = 7.9, 2.4 Hz, 1H), 4.59 – 4.54 (m, 1H), 4.37 – 4.31 (m, 2H), 4.19 (dd, *J* = 7.9, 1.9 Hz, 1H), 4.03 – 3.97 (m, 1H), 3.95 – 3.88 (m, 1H), 3.78 (ddd, *J* = 9.4, 4.3, 1.9 Hz, 1H), 3.72 (s, 3H), 2.41 – 2.23 (m, 1H), 2.20 – 2.07 (m, 1H), 1.88 – 1.77 (m, 1H), 1.74 – 1.65 (m, 1H), 1.47 (s, 3H), 1.37 (s, 3H), 1.31 (d, *J* = 3.6 Hz, 6H). **^{13}C NMR (101 MHz, Acetone-*d*$_6$)** δ 170.7, 164.8 (t, *J* = 29.5 Hz), 119.3 (t, *J* = 250.8 Hz), 109.5, 108.9, 97.3, 73.4, 71.8, 71.3, 67.6, 62.3, 55.8, 52.6, 31.5 (t, *J* = 23.6 Hz), 26.4, 26.3, 25.1, 24.6, 23.4 (t, *J* = 4.6 Hz). **^{19}F NMR (376 MHz, Acetone-*d*$_6$)** δ -106.43 (d, *J* = 250.6 Hz, 1F), -107.93 (d, *J* = 251.0 Hz, 1F). **HRMS (ESI)** *m/z:* [M+Na]$^+$ Calcd. for $C_{19}H_{29}F_2NO_9Na^+$ 476.1702; Found: 476.1706. **$[\alpha]_D^{20.0}$** = −33.6 (*c* = 0.2 in acetone).

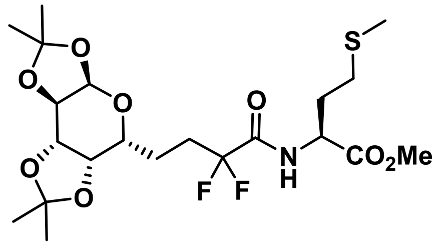

Methyl (2,2-difluoro-4-((3aR,5R,5aS,8aS,8bR)-2,2,7,7-tetramethyltetrahydro-5H-bis([1,3]dioxolo)[4,5-b:4',5'-d]pyran-5-yl)butanoyl)-L-methioninate **(3e)** was synthesized according to the general procedure 1: Flash column chromatography purification 7-12% of EtOAc in hexanes afforded the title as a pale yellow oil in 65**% (32.51 mg).** **^{1}H NMR (400 MHz, Acetone-*d*$_6$)** δ 8.15 (d, *J* = 8.2 Hz, 1H), 5.46 (d, *J* = 5.1 Hz, 1H), 4.66 (td, *J* = 8.7, 4.8 Hz, 1H), 4.60 (dd, *J* = 7.9, 2.4 Hz, 1H), 4.32 (dd, *J* = 5.1, 2.4 Hz, 1H), 4.18 (dd, *J* = 7.9, 1.9 Hz, 1H), 3.78 (ddd, *J* = 9.3, 4.2, 1.9 Hz, 1H), 3.72 (s, 3H), 2.67 – 2.53 (m, 2H), 2.36 – 2.24 (m, 1H), 2.24 – 2.10 (m, 3H), 2.08 (s, 3H), 1.86 – 1.75 (m, 1H), 1.72 – 1.62 (m, 1H), 1.47 (s, 3H), 1.38 (s, 3H), 1.31 (d,

J = 3.2 Hz, 6H). **^{13}C NMR (101 MHz, Acetone-*d_6*)** δ 172.1, 165.1 (t, J = 29.4 Hz), 119.2 (t, J = 250.8 Hz), 109.5, 108.9, 97.3, 73.5, 71.9, 71.4, 67.6, 52.6, 52.3, 31.7 (t, J = 23.7 Hz), 31.3, 30.8, 26.4, 26.3, 25.1, 24.6, 23.5 (t, J = 4.7 Hz), 15.1. **^{19}F NMR (376 MHz, Acetone-*d_6*)** δ -106.42 (d, J = 251.0 Hz, 1F), -107.53 (d, J = 251.0 Hz, 1F). **HRMS (ESI)** *m/z:* $[M+Na]^+$ Calcd. for $C_{21}H_{33}F_2NO_8SNa^+$ 520.1787; Found: 520.1786. **$[\alpha]_D^{20.0}$** = −38.9 (c = 0.2 in acetone).

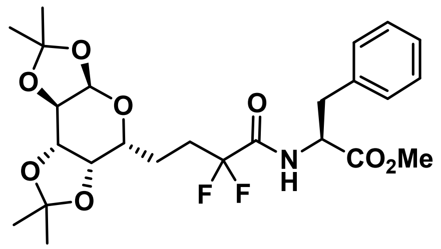

Methyl (2,2-difluoro-4-((3aR,5R,5aS,8aS,8bR)-2,2,7,7-tetramethyltetrahydro-5H-bis([1,3]dioxolo)[4,5-b:4',5'-d]pyran-5-yl)butanoyl)-L-phenylalaninate **(3f)** was synthesized according to the general procedure 1: Flash column chromatography purification 9-15% of EtOAc in hexanes afforded the title as a pale yellow solid in 77**% (39.7 mg). ^{1}H NMR (400 MHz, Acetone-*d_6*)** δ 8.03 (d, J = 8.3 Hz, 1H), 7.35 – 7.19 (m, 5H), 5.45 (d, J = 4.7 Hz, 1H), 4.79 – 4.72 (m, 1H), 4.63 – 4.57 (m, 1H), 4.35 – 4.28 (m, 1H), 4.17 – 4.11 (m, 1H), 3.75 – 3.67 (m, 4H), 3.27 (ddd, J = 14.0, 5.1, 1.8 Hz, 1H), 3.11 (ddd, J = 14.0, 9.4, 1.8 Hz, 1H), 2.28 – 2.10 (m, 1H), 2.02 – 1.88 (m, 1H), 1.78 – 1.64 (m, 1H), 1.54 – 1.45 (m, 4H), 1.37 (s, 3H), 1.33 (s, 3H), 1.30 (s, 3H). **^{13}C NMR (101 MHz, Acetone-*d_6*)** δ 171.8, 171.8, 164.9, 164.6, 164.3, 137.8, 130.0, 130.0, 129.3, 127.6, 121.6, 119.1, 116.6, 109.5, 108.9, 97.3, 73.4, 71.8, 71.3, 67.5, 54.5, 52.6, 37.4, 31.8, 31.5, 31.3, 26.4, 26.3, 25.1, 24.6, 23.4, 23.3, 23.3. **^{19}F NMR (376 MHz, Acetone-*d_6*)** δ -106.62 (d, J = 250.2 Hz, 1F), -107.79 (d, J = 251.1 Hz, 1F). **HRMS (ESI)** *m/z:* $[M+K]^+$ Calcd. for $C_{25}H_{33}F_2NO_8K^+$ 552.1806; Found: 552.1811. **Melting point:** 103.6 - 105.4 °C. **$[\alpha]_D^{20.0}$** = −39.8 (c = 0.2 in acetone).

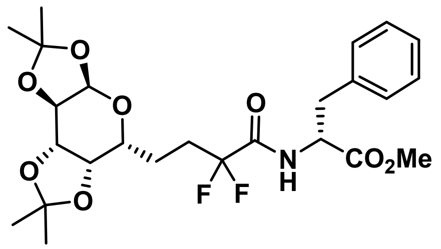

Methyl (2,2-difluoro-4-((3aR,5R,5aS,8aS,8bR)-2,2,7,7-tetramethyltetrahydro-5H-bis([1,3]dioxolo)[4,5-b:4',5'-d]pyran-5-yl)butanoyl)-D-phenylalaninate **(3g)** was synthesized according to the general procedure 1: Flash column chromatography purification 10-17% of EtOAc in hexanes afforded the title as a pale yellow solid in 70**% (35.71 mg). ^{1}H NMR (400 MHz, Acetone-*d_6*)** δ 8.01 (d, J

= 8.3 Hz, 1H), 7.34 – 7.21 (m, 5H), 5.46 (d, J = 5.1 Hz, 1H), 4.81 – 4.71 (m, 1H), 4.60 (dd, J = 7.9, 2.4 Hz, 1H), 4.32 (dd, J = 5.1, 2.4 Hz, 1H), 4.14 (dd, J = 7.9, 1.9 Hz, 1H), 3.73 – 3.68 (m, 4H), 3.28 (dd, J = 13.9, 5.1 Hz, 1H), 3.11 (dd, J = 13.9, 9.5 Hz, 1H), 2.27 – 2.09 (m, 1H), 2.02 – 1.88 (m, 1H), 1.76 – 1.65 (m, 1H), 1.58 – 1.50 (m, 1H), 1.48 (s, 3H), 1.37 (s, 3H), 1.32 (d, J = 2.8 Hz, 6H). **^{13}C NMR (101 MHz, Acetone-*d*$_6$)** δ 171.8, 164.6 (t, J = 29.4 Hz), 137.8, 130.0, 129.3, 127.7, 119.1 (t, J = 250.9 Hz), 109.5, 108.9, 97.3, 73.4, 71.8, 71.3, 67.5, 54.5, 52.6, 37.4, 31.6 (t, J = 23.7 Hz), 26.4, 26.3, 25.1, 24.6, 23.4 (t, J = 4.7 Hz). **^{19}F NMR (376 MHz, Acetone-*d*$_6$)** δ -106.91 (d, J = 248.6 Hz, 1F), -107.64 (d, J = 248.6 Hz, 1F). **HRMS (ESI)** *m/z:* [M+Na]$^+$ Calcd. for $C_{25}H_{33}F_2NO_8Na^+$ 536.2066; Found: 536.2065. **Melting point:** 84.5 - 86.4 °C. **[α]$_D$$^{20.0}$** = −22.6 ($c$ = 0.2 in acetone).

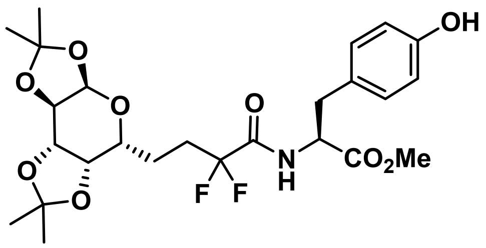

Methyl (2,2-difluoro-4-((3aR,5R,5aS,8aS,8bR)-2,2,7,7-tetramethyltetrahydro-5H-bis([1,3]dioxolo)[4,5-b:4',5'-d]pyran-5-yl)butanoyl)-L-tyrosinate **(3h)** was synthesized according to the general procedure 1: Flash column chromatography purification 15-25% of EtOAc in hexanes afforded the title as a white solid in 77**% (40.7 mg). ^{1}H NMR (400 MHz, Acetone-*d*$_6$)** δ 8.09 (s, 1H), 7.78 (d, J = 8.1 Hz, 1H), 6.95 (d, J = 8.4 Hz, 2H), 6.65 (d, J = 8.5 Hz, 2H), 5.32 (d, J = 5.1 Hz, 1H), 4.59 – 4.51 (m, 1H), 4.47 (dd, J = 7.9, 2.3 Hz, 1H), 4.19 (dd, J = 5.1, 2.3 Hz, 1H), 4.02 (dd, J = 7.9, 1.9 Hz, 1H), 3.62 – 3.58 (m, 1H), 3.57 (s, 3H), 3.03 (dd, J = 14.0, 5.2 Hz, 1H), 2.89 (dd, J = 14.1, 8.9 Hz, 1H), 2.17 – 2.00 (m, 1H), 1.90 – 1.80 (m, 1H), 1.68 – 1.56 (m, 1H), 1.42 (ddt, J = 13.7, 11.4, 4.7 Hz, 1H), 1.34 (s, 3H), 1.24 (s, 3H), 1.20 (s, 3H), 1.17 (s, 3H). **^{13}C NMR (101 MHz, Acetone-*d*$_6$)** δ 171.9, 164.6 (t, J = 29.4 Hz), 157.2, 131.0, 128.2, 119.1 (t, J = 250.8 Hz), 116.2, 109.5, 108.9, 97.3, 73.4, 71.8, 71.3, 67.5, 54.8, 52.5, 36.7, 31.5 (t, J = 23.6 Hz), 26.4, 26.3, 25.1, 24.6, 23.3 (t, J = 4.6 Hz). **^{19}F NMR (376 MHz, Acetone-*d*$_6$)** δ -106.79 (d, J = 249.2 Hz, 1F), -107.57 (d, J = 251.3 Hz, 1F). **HRMS (ESI)** *m/z:* [M+Na]$^+$ Calcd. for $C_{25}H_{33}F_2NO_9Na^+$ 552.2015; Found: 552.2005. **Melting point:** 50.8 – 56.5 °C. **[α]$_D$$^{20.0}$** = −31.3 ($c$ = 0.2 in acetone).

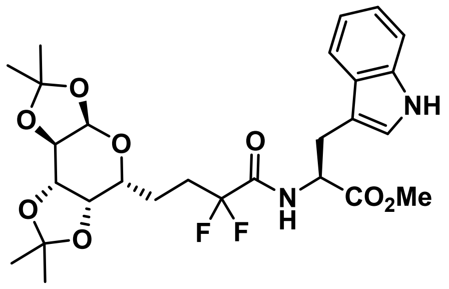

***Methyl (2,2-difluoro-4-((3aR,5R,5aS,8aS,8bR)-2,2,7,7-tetramethyltetrahydro-5H-bis([1,3]dioxolo)[4,5-b:4',5'-d]pyran-5-yl)butanoyl)-L-tryptophanate* (3i)** was synthesized according to the general procedure 1: Flash column chromatography purification 20-25% of EtOAc in hexanes afforded the title as a viscous yellow oil in 37**% (20.37 mg).** **^{1}H NMR (400 MHz, Acetone-*d*$_6$)** δ 10.06 (s, 1H), 7.89 (d, *J* = 8.0 Hz, 1H), 7.59 (d, *J* = 7.9 Hz, 1H), 7.39 (dt, *J* = 8.1, 1.0 Hz, 1H), 7.23 (d, *J* = 2.5 Hz, 1H), 7.11 (ddd, *J* = 8.1, 7.0, 1.3 Hz, 1H), 7.05 (ddd, *J* = 8.0, 7.0, 1.1 Hz, 1H), 5.46 (d, *J* = 5.1 Hz, 1H), 4.81 (td, *J* = 8.1, 5.2 Hz, 1H), 4.59 (dd, *J* = 7.9, 2.3 Hz, 1H), 4.32 (dd, *J* = 5.1, 2.3 Hz, 1H), 4.11 (dd, *J* = 7.9, 1.9 Hz, 1H), 3.74 – 3.69 (m, 4H), 3.41 (dd, *J* = 14.8, 5.2 Hz, 1H), 3.32 (dd, *J* = 14.8, 8.2 Hz, 1H), 2.32 – 2.14 (m, 1H), 2.03 – 1.91 (m, 1H), 1.79 – 1.68 (m, 1H), 1.59 – 1.49 (m, 1H), 1.47 (s, 3H), 1.37 (s, 3H), 1.32 (s, 3H), 1.31 (s, 3H). **^{13}C NMR (101 MHz, Acetone-*d*$_6$)** δ 172.1, 164.6 (t, *J* = 29.4 Hz), 137.6, 128.4, 124.5, 122.3, 119.8, 119.2 (t, *J* = 250.9 Hz), 119.0, 112.3, 110.5, 109.5, 108.9, 97.3, 73.4, 71.8, 71.3, 67.5, 54.0, 52.6, 31.5 (t, *J* = 23.7 Hz), 27.7, 26.4, 26.3, 25.1, 24.7, 23.4 (t, *J* = 4.7 Hz). **^{19}F NMR (376 MHz, Acetone-*d*$_6$)** δ -107.13, 2F. **HRMS (ESI)** *m/z:* $[M+K]^+$ Calcd. for $C_{27}H_{34}F_2N_2O_8K^+$ 591.1915; Found: 591.1913. **$[\alpha]_D^{20.0}$** = −39.1 (*c* = 0.2 in acetone).

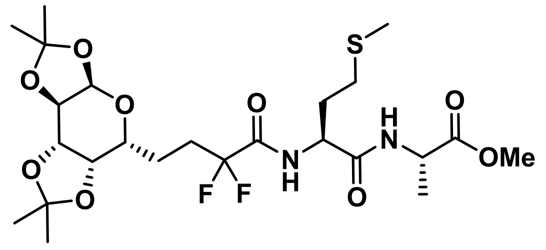

***Methyl (2,2-difluoro-4-((3aR,5R,5aS,8aS,8bR)-2,2,7,7-tetramethyltetrahydro-5H-bis([1,3]dioxolo)[4,5-b:4',5'-d]pyran-5-yl)butanoyl)-L-methionyl-L-alaninate* (3j)** was synthesized according to the general procedure 1: Flash column chromatography purification using a mixture of 5% Acetone, 50% DCM, 45% hexanes afforded the title as a colorless oil in 51**% (29.03 mg).** **^{1}H NMR (400 MHz, Acetone-*d*$_6$)** δ 7.89 (d, *J* = 7.9 Hz, 1H), 7.75 (d, *J* = 7.2 Hz, 1H), 5.45 (d, *J* = 4.6 Hz, 1H), 4.66 – 4.57 (m, 2H), 4.45 (p, *J* = 6.9 Hz, 1H), 4.34 – 4.30 (m, 1H), 4.20 – 4.14 (m, 1H), 3.80 – 3.75 (m, 1H), 3.68 (s, 3H), 2.60 – 2.53 (m, 2H), 2.36 – 2.22 (m, 2H), 2.21 – 2.10 (m, 2H), 2.07 (s, 3H), 1.84 – 1.73 (m, 1H), 1.71 – 1.62 (m, 1H), 1.46 (s, 3H), 1.40 – 1.35 (m, 6H), 1.30 (s, 6H). **^{13}C NMR (101 MHz, Acetone-*d*$_6$)** δ 173.6, 170.8, 164.6 (t, *J* = 29.1 Hz), 119.2 (t, *J* = 250.1

Hz), 109.5, 108.9, 97.3, 73.4, 71.8, 71.3, 67.5, 53.0, 52.4, 48.9, 32.7, 31.5 (t, *J* = 23.6 Hz), 26.4, 26.3, 25.1, 24.6, 23.5 (t, *J* = 4.6 Hz), 17.6, 15.2. **^{19}F NMR (376 MHz, Acetone-*d*$_6$)** δ -106.62 (d, *J* = 248.7 Hz, 1F), -107.32 (d, *J* = 252.2 Hz, 1F). **HRMS (ESI)** *m/z:* [M+Na]$^+$ Calcd. for $C_{24}H_{38}F_2N_2O_9SNa^+$ 591.2158; Found: 591.2161. **[α]$_D$$^{20.0}$** = −32.5 (*c* = 0.2 in acetone).

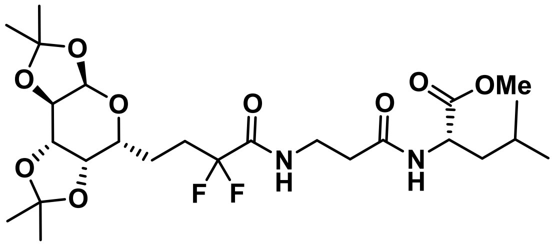

Methyl (3-(2,2-difluoro-4-((3aR,5R,5aS,8aS,8bR)-2,2,7,7-tetramethyltetrahydro-5H-bis([1,3]dioxolo)[4,5-b:4',5'-d]pyran-5-yl)butanamido)propanoyl)-L-leucinate **(3k)** was synthesized according to the general procedure 1: Flash column chromatography purification using a mixture of 5% Acetone, 50% DCM, 45% hexanes afforded the title as a white solid in 60**% (32.8 mg). ^{1}H NMR (400 MHz, Acetone-*d*$_6$)** δ 7.91 (s, 1H), 7.51 (d, *J* = 8.1 Hz, 1H), 5.45 (d, *J* = 4.7 Hz, 1H), 4.60 (d, *J* = 7.9 Hz, 1H), 4.50 (q, *J* = 7.5 Hz, 1H), 4.36 – 4.29 (m, 1H), 4.18 (d, *J* = 7.9 Hz, 1H), 3.79 – 3.74 (m, 1H), 3.67 (s, 3H), 3.52 (q, *J* = 6.0 Hz, 2H), 2.51 (t, *J* = 6.9 Hz, 2H), 2.35 – 2.20 (m, 1H), 2.17 – 2.08 (m, 1H), 1.81 – 1.65 (m, 3H), 1.60 – 1.55 (m, 2H), 1.47 (s, 3H), 1.37 (s, 3H), 1.31 (s, 6H), 0.94 – 0.88 (m, 6H)**. ^{13}C NMR (101 MHz, Acetone-*d*$_6$)** δ 173.8, 171.6, 164.6 (t, *J* = 29.0 Hz), 119.3 (t, *J* = 250.8 Hz), 109.5, 108.9, 97.3, 73.4, 71.8, 71.3, 67.5, 52.2, 51.4, 41.4, 36.5, 35.3, 31.4 (t, *J* = 23.9 Hz), 26.4, 26.3, 25.4, 25.1, 24.6, 23.4 (t, *J* = 4.5 Hz), 23.2, 21.8. **^{19}F NMR (376 MHz, Acetone-*d*$_6$)** δ -107.35, 2F. **HRMS (ESI)** *m/z:* [M+Na]$^+$ Calcd. for $C_{25}H_{40}F_2N_2O_9Na^+$ 573.2594; Found: 573.2593. **Melting point:** 140.9 - 142.7 °C. **[α]$_D$$^{20.0}$** = −33.9 (*c* = 0.2 in acetone).

6. Characterization data of compounds 4a-4j

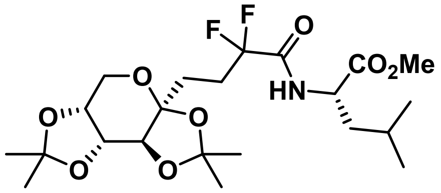

Methyl (2,2-difluoro-4-((3aS,5aR,8aR,8bS)-2,2,7,7-tetramethyltetrahydro-3aH-bis([1,3]dioxolo)[4,5-b:4',5'-d]pyran-3a-yl)butanoyl)-L-leucinate **(4a)** was synthesized according to the general procedure 1: Flash column chromatography purification 8-15% of EtOAc in cyclohexane afforded the title as a light yellow oil in 65**% (26.1 mg). ^{1}H NMR (400 MHz, Acetone-*d*$_6$)** δ 8.12 (d, *J* = 7.8 Hz, 1H), 4.59 (dd, *J* = 7.9, 2.4 Hz, 1H),

4.57 – 4.52 (m, 1H), 4.23 (ddd, J = 7.9, 2.0, 0.8 Hz, 1H), 4.18 (d, J = 2.5 Hz, 1H), 3.82 (dd, J = 13.0, 2.0 Hz, 1H), 3.71 (s, 3H), 3.59 (dd, J = 13.0, 0.8 Hz, 1H), 2.55 – 2.20 (m, 2H), 1.96 (dtd, J = 41.1, 13.2, 4.4 Hz, 2H), 1.84 – 1.63 (m, 3H), 1.48 (s, 3H), 1.41 (s, 3H), 1.36 (s, 3H), 1.33 (s, 3H), 0.97 – 0.92 (m, 6H). **^{13}C NMR (101 MHz, Acetone-*d*$_6$)** δ 172.8, 165.0 (t, J = 29.5 Hz), 119.1 (t, J = 250.8 Hz), 109.3, 108.2, 103.8, 74.6, 71.4, 71.3, 61.7, 52.5, 51.5, 40.5, 33.7 (t, J = 4.5 Hz), 29.8, 26.5, 26.1, 25.5, 25.2, 24.3, 23.2, 21.5. **^{19}F NMR (377 MHz, Acetone-*d*$_6$)** δ -107.10 (d, J = 249.8 Hz, 1F), -108.18 (d, J = 249.8 Hz, 1F). **HRMS (ESI)** *m/z:* [M+Na]$^+$ Calcd. for $C_{22}H_{35}F_2NO_8Na^+$ 502.2223; Found: 502.2214. **[α]$_D$$^{20.0}$** = −31.1 ($c$ = 0.2 in acetone).

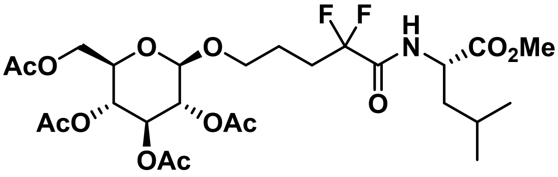

(2R,3R,4S,5R,6R)-2-(acetoxymethyl)-6-((4,4-difluoro-5-(((S)-1-methoxy-4-methyl-1-oxopentan-2-yl)amino)-5-oxopentyl)oxy)tetrahydro-2H-pyran-3,4,5-triyl triacetate **(4b)** was synthesized according to the general procedure 1: Flash column chromatography purification 15-30% of EtOAc in cyclohexane afforded the title as a colorless oil in 53**% (32.5 mg). ^{1}H NMR (400 MHz, CDCl$_3$)** δ 6.70 (d, J = 8.4 Hz, 1H), 5.37 (dd, J = 3.5, 1.2 Hz, 1H), 5.18 (dd, J = 10.5, 7.9 Hz, 1H), 5.00 (dd, J = 10.4, 3.4 Hz, 1H), 4.66 – 4.57 (m, 1H), 4.45 (d, J = 7.9 Hz, 1H), 4.14 (dddd, 2H), 3.94 – 3.86 (m, 2H), 3.75 (s, 3H), 3.55 (ddd, J = 9.8, 6.5 Hz, 1H), 2.22 – 2.16 (m, 1H), 2.16 – 2.08 (m, 4H), 2.04 (d, J = 6.6 Hz, 6H), 1.97 (s, 3H), 1.78 (ddd, J = 10.1, 5.9, 2.8 Hz, 1H), 1.72 – 1.58 (m, 4H), 0.97 – 0.92 (m, 6H). **^{13}C NMR (101 MHz, CDCl$_3$)** δ 172.4, 170.5, 170.3, 170.2, 169.6, 164.0 (t, J = 29.4 Hz), 118.1 (t, J = 252.2 Hz), 101.4, 71.1, 70.9, 69.0, 68.8, 67.2, 61.4, 52.6, 50.9, 41.5, 30.6 (t, J = 23.5 Hz), 25.0, 22.8, 22.0, 20.8, 20.7, 20.7. **^{19}F NMR (377 MHz, CDCl$_3$)** δ -105.12 (d, J = 255.2 Hz), -107.55 (d, J = 255.1 Hz). **HRMS (ESI)** *m/z:* [M+Na]$^+$ Calcd. for $C_{26}H_{39}F_2NO_{13}Na^+$ 634.2281; Found: 634.2274. **[α]$_D$$^{20.0}$** = −15.1 ($c$ = 0.2 in acetone).

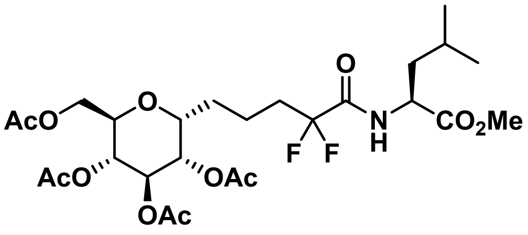

(2R,3R,4R,5S,6R)-2-(acetoxymethyl)-6-(4,4-difluoro-5-(((S)-1-methoxy-4-methyl-1-oxopentan-2-yl)amino)-5-oxopentyl)tetrahydro-2H-pyran-3,4,5-triyl triacetate **(4c)** was synthesized according to

the general procedure 1: Flash column chromatography purification 15-30% of EtOAc in cyclohexane afforded the title as a light yellow oil in 39**% (23.0 mg).** **^{1}H NMR (400 MHz, $CDCl_3$)** δ 6.73 (d, *J* = 8.5 Hz, 1H), 5.27 (t, *J* = 9.1 Hz, 1H), 5.05 (dd, *J* = 9.6, 5.6 Hz, 1H), 4.94 (t, *J* = 9.0 Hz, 1H), 4.65 – 4.55 (m, 1H), 4.21 (dd, *J* = 12.1, 5.7 Hz, 1H), 4.13 (ddd, *J* = 12.2, 6.0, 3.4 Hz, 1H), 4.06 (dd, *J* = 12.1, 2.5 Hz, 1H), 3.83 – 3.77 (m, 1H), 3.74 (s, 3H), 2.23 – 2.08 (m, 2H), 2.07 (s, 3H), 2.03 (s, 3H), 2.02 (s, 3H), 2.01 (s, 3H), 1.91 – 1.79 (m, 1H), 1.75 – 1.59 (m, 4H), 1.57 – 1.45 (m, 2H), 0.94 (d, *J* = 6.0 Hz, 6H). **^{13}C NMR (101 MHz, $CDCl_3$)** δ 172.4, 170.8, 170.2, 169.7, 169.7, 164.0 (t, *J* = 29.2 Hz), 118.0 (t, *J* = 252.5 Hz), 72.3, 70.4, 70.3, 68.9, 62.4, 52.7, 50.9, 41.4, 33.4 (t, *J* = 23.0 Hz), 25.0, 22.8, 21.9, 20.8, 20.7, 17.4 (t, *J* = 4.5 Hz). **^{19}F NMR (377 MHz, $CDCl_3$)** δ -104.78 (dd, *J* = 255.9, 3.3 Hz), -108.03 (dd, *J* = 255.9, 4.3 Hz). **HRMS (ESI)** *m/z:* $[M+Na]^+$ Calcd. for $C_{26}H_{39}F_2NO_{12}Na^+$ 618.2333; Found: 618.2326. **$[\alpha]_D^{20.0}$** = +23.6 (*c* = 0.1 in acetone).

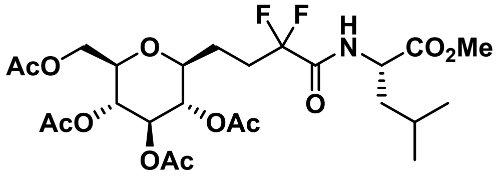

(2R,3R,4R,5S,6S)-2-(acetoxymethyl)-6-(3,3-difluoro-4-(((S)-1-methoxy-4-methyl-1-oxopentan-2-yl)amino)-4-oxobutyl)tetrahydro-2H-pyran-3,4,5-triyl triacetate (4d) was synthesized according to the general procedure 1: Flash column chromatography purification 15-30% of EtOAc in cyclohexane afforded the title as a white solid in 67**% (39.0 mg).** **^{1}H NMR (400 MHz, $CDCl_3$)** δ 6.70 (d, *J* = 8.4 Hz, 1H), 5.15 (t, *J* = 9.3 Hz, 1H), 5.02 (t, *J* = 9.7 Hz, 1H), 4.85 (t, *J* = 9.5 Hz, 1H), 4.63 – 4.54 (m, 1H), 4.22 (dd, *J* = 12.3, 4.8 Hz, 1H), 4.10 – 4.02 (m, 1H), 3.73 (s, 3H), 3.60 (ddd, *J* = 10.3, 5.4, 2.4 Hz, 1H), 3.42 (td, *J* = 9.4, 2.8 Hz, 1H), 2.40 – 2.22 (m, 1H), 2.21 – 2.10 (m, 1H), 2.06 (s, 3H), 2.03 (s, 3H), 2.00 (s, 3H), 1.97 (s, 3H), 1.82 – 1.72 (m, 1H), 1.72 – 1.54 (m, 4H), 0.96 – 0.91 (m, 6H). **^{13}C NMR (101 MHz, $CDCl_3$)** δ 172.4, 170.8, 170.4, 169.8, 169.6, 163.9 (t, *J* = 29.3 Hz), 117.8 (t, *J* = 252.5 Hz), 76.8, 75.8, 74.3, 71.7, 68.7, 62.3, 52.6, 50.8, 41.2, 29.8 (t, *J* = 23.9 Hz), 24.9, 23.6 (t, *J* = 4.3 Hz), 22.8, 21.9, 20.8, 20.7, 20.7. **^{19}F NMR (377 MHz, $CDCl_3$)** δ -105.18 (d, *J* = 255.2 Hz, 1F), -108.12 (d, *J* = 255.2 Hz, 1F). **HRMS (ESI)** *m/z:* $[M+Na]^+$ Calcd. for $C_{25}H_{37}F_2NO_{12}Na^+$ 604.2176; Found: 604.2167. **Melting point:** 84.7 – 85.7 °C. **$[\alpha]_D^{20.0}$** = −25.2 (*c* = 0.2 in acetone).

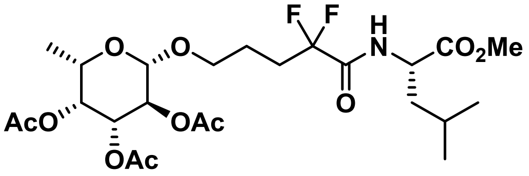

***(2S,3S,4R,5R,6S)-2-((4,4-difluoro-5-(((S)-1-methoxy-4-methyl-1-oxopentan-2-yl)amino)-5-oxopentyl)oxy)-6-methyltetrahydro-2H-pyran-3,4,5-triyl triacetate* (4e)** was synthesized according to the general procedure 1: Flash column chromatography purification 15-30% of EtOAc in cyclohexane afforded the title as a light yellow oil in 52**% (28.5 mg).** **^{1}H NMR (400 MHz, $CDCl_3$)** δ 6.71 (d, *J* = 8.4 Hz, 1H), 5.22 (dd, *J* = 3.5, 1.1 Hz, 1H), 5.16 (dd, *J* = 10.5, 7.9 Hz, 1H), 4.99 (dd, *J* = 10.5, 3.4 Hz, 1H), 4.65 – 4.57 (m, 1H), 4.41 (d, *J* = 7.9 Hz, 1H), 3.93 (dt, *J* = 9.9, 5.8 Hz, 1H), 3.82 – 3.76 (m, 1H), 3.75 (s, 3H), 3.51 (dt, *J* = 9.8, 6.5 Hz, 1H), 2.21 – 2.07 (m, 5H), 2.04 (s, 3H), 1.97 (s, 3H), 1.82 – 1.73 (m, 2H), 1.73 – 1.66 (m, 1H), 1.65 – 1.56 (m, 2H), 1.20 (d, *J* = 6.4 Hz, 3H), 0.97 – 0.92 (m, 6H). **^{13}C NMR (101 MHz, $CDCl_3$)** δ 172.3, 170.7, 170.2, 169.6, 163.9 (t, *J* = 29.3 Hz), 118.0 (t, *J* = 251.6 Hz), 101.0, 71.3, 70.3, 69.1, 68.9, 68.5, 52.6, 50.7, 41.3, 30.5 (t, *J* = 23.5 Hz), 24.8, 22.7, 21.8, 21.8 (t, *J* = 4.2 Hz), 20.7, 20.6, 16.0. **^{19}F NMR (377 MHz, $CDCl_3$)** δ -105.31 (d, *J* = 255.1 Hz, 1F), -107.38 (d, *J* = 254.8 Hz, 1F). **HRMS (ESI)** *m/z:* $[M+NH_4]^+$ Calcd. for $C_{24}H_{37}F_2NO_{11}NH_4^+$ 571.2673; Found: 571.2668. . **$[\alpha]_D^{20.0}$** = −10.9 (*c* = 0.2 in acetone).

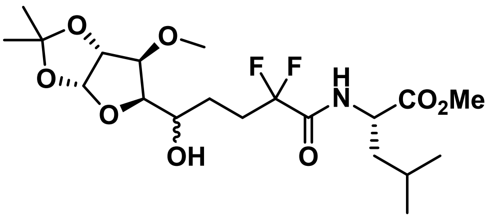

Methyl (2,2-difluoro-5-hydroxy-5-((3aR,5R,6S,6aR)-6-methoxy-2,2-dimethyltetrahydrofuro[2,3-d][1,3]dioxol-5-yl)pentanoyl)-L-leucinate (4f) was synthesized according to the general procedure 1: Flash column chromatography purification 15-40% of EtOAc in cyclohexane afforded the title as a colorless oil in 51**% (23.1 mg)** with a diastereomeric ratio of 1:1. **^{1}H NMR (400 MHz, Acetone-*d*$_6$)** δ 8.09 (s, 1H), 5.83 (dd, *J* = 19.3, 3.8 Hz, 1H), 4.67 (dd, *J* = 14.4, 3.8 Hz, 1H), 4.58 – 4.50 (m, 1H), 3.98 (d, *J* = 5.9 Hz, 0.5H), 3.95 – 3.86 (m, 1H), 3.85 – 3.80 (m, 1H), 3.80 – 3.78 (m, 1H), 3.71 (s, 0.5H), 3.70 (d, *J* = 4.4 Hz, 3H), 3.39 (d, *J* = 10.4 Hz, 3H), 2.46 – 2.27 (m, 1H), 2.21 – 2.10 (m, 1H), 1.90 – 1.62 (m, 4H), 1.59 – 1.47 (m, 1H), 1.41 (s, 3H), 1.28 (s, 3H), 0.96 – 0.91 (m, 6H). **^{13}C NMR (101 MHz, Acetone-*d*$_6$)** δ 172.9, 172.8, 165.1 (td, *J* = 29.5, 3.6 Hz), 119.5 (td, *J* = 250.5, 11.4 Hz), 111.8, 111.8, 106.0, 105.7, 85.3, 84.4, 84.4, 83.9, 82.3, 82.1, 69.4, 67.5, 57.8, 57.5, 52.5, 52.5, 51.6, 51.5, 40.5, 40.4, 31.6 (t, *J* = 24.0 Hz), 31.4 (t, *J* = 23.5 Hz), 27.8 (t, *J* = 4.3 Hz), 27.1, 27.0, 26.5, 26.4, 26.0 (t, *J* = 4.5 Hz), 25.5, 25.4, 23.3,

23.2, 21.5, 21.4. **^{19}F NMR (377 MHz, Acetone-*d*$_6$)** δ -106.25 (d, *J* = 249.7 Hz, 0.5F), -106.74 (d, *J* = 248.1 Hz, 0.5F), -107.21 (d, *J* = 249.7 Hz, 0.5F), -107.89 (d, *J* = 249.7 Hz, 0.5F). **HRMS (ESI)** *m/z:* $[M+Na]^+$ Calcd. for $C_{20}H_{33}F_2NO_8Na^+$ 476.2066; Found: 476.2068.

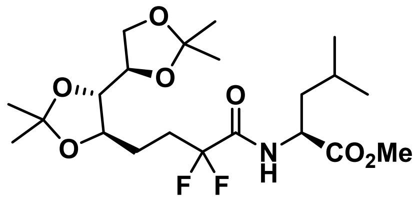

Methyl (2,2-difluoro-4-((4S,4'R,5R)-2,2,2',2'-tetramethyl-[4,4'-bi(1,3-dioxolan)]-5-yl)butanoyl)-L-leucinate **(4g)** was synthesized according to the general procedure 1: Flash column chromatography purification 10-12% of EtOAc in cyclohexane afforded the title as a colorless oil which solidified on the fridge to give a white solid in 45**% (20.3 mg). ^{1}H NMR (400 MHz, Acetone-*d*$_6$)** δ 8.10 (d, *J* = 8.4 Hz, 1H), 4.54 (ddd, *J* = 10.4, 8.3, 4.5 Hz, 1H), 4.14 – 4.01 (m, 2H), 3.95 (td, *J* = 7.9, 3.6 Hz, 1H), 3.87 (dd, *J* = 8.1, 4.8 Hz, 1H), 3.70 (s, 3H), 3.56 (t, *J* = 7.7 Hz, 1H), 2.36 – 2.14 (m, 2H), 1.99 – 1.86 (m, 1H), 1.83 – 1.62 (m, 4H), 1.35 (s, 3H), 1.34 (s, 3H), 1.31 (s, 3H), 1.29 (s, 3H), 0.96 – 0.91 (m, 6H). **^{13}C NMR (101 MHz, Acetone-*d*$_6$)** δ 172.8, 164.9 (t, *J* = 29.4 Hz), 119.0 (t, *J* = 250.8 Hz), 110.1, 109.6, 82.0, 80.0, 77.8, 68.1, 52.5, 51.6, 40.5, 31.7 (t, *J* = 23.8 Hz), 27.6, 27.2, 27.0, 26.5 (t, *J* = 4.6 Hz), 25.5, 25.5, 23.2, 21.5. **^{19}F NMR (377 MHz, Acetone-*d*$_6$)** δ -106.59 (d, *J* = 250.9 Hz, 1F), -107.91 (d, *J* = 250.9 Hz, 1F). **HRMS (ESI)** *m/z:* $[M+Na]^+$ Calcd. for $C_{21}H_{35}F_2NO_7Na^+$ 474.2274; Found: 474.2267. **Melting point:** 71.2 - 71.7 °C. **$[\alpha]_D^{20.0}$** = −7.5 (*c* = 0.2 in acetone).

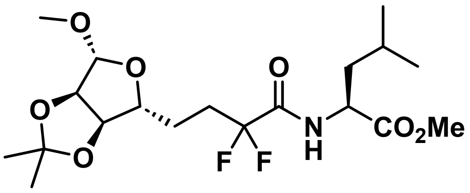

Methyl (2,2-difluoro-4-((3aR,4R,6R,6aR)-6-methoxy-2,2-dimethyltetrahydrofuro[3,4-d][1,3]dioxol-4-yl)butanoyl)-L-leucinate **(4h)** was synthesized according to the general procedure 1: Flash column chromatography purification 8-15% of EtOAc in cyclohexane afforded the title as a light yellow oil in 60**% (25.3 mg). ^{1}H NMR (400 MHz, Acetone-*d*$_6$)** δ 8.03 (d, *J* = 8.0 Hz, 1H), 4.88 (s, 1H), 4.59 (s, 2H), 4.58 – 4.51 (m, 1H), 4.10 (dd, *J* = 9.2, 6.1 Hz, 1H), 3.70 (s, 3H), 3.31 (s, 3H), 2.38 – 2.10 (m, 2H), 1.82 – 1.62 (m, 5H), 1.40 (s, 3H), 1.28 (s, 3H), 0.96 – 0.91 (m, 6H). **^{13}C NMR (101 MHz, Acetone-*d*$_6$)** δ 172.8, 164.8 (t, *J* = 29.4 Hz), 119.1 (t, *J* = 250.9 Hz), 112.6, 110.6, 86.9, 86.3, 84.8, 55.2, 52.5, 51.6, 40.5,

32.1 (t, J = 23.8 Hz), 28.1 (t, J = 4.4 Hz), 26.8, 25.5, 25.1, 23.2, 21.5. **^{19}F NMR (377 MHz, Acetone-d_6)** δ -106.14 (d, J = 251.6 Hz, 1F), -107.15 (d, J = 251.5 Hz, 1F). **HRMS (ESI)** *m/z:* $[M+Na]^+$ Calcd. for $C_{19}H_{31}F_2NO_7Na^+$ 446.1961; Found: 446.1960. **$[\alpha]_D^{20.0}$** = −46.6 (c = 0.2 in acetone).

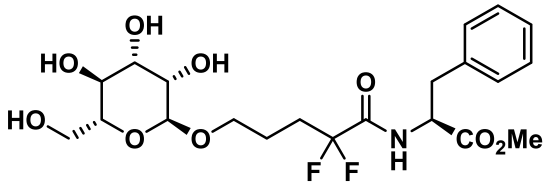

***Methyl (2,2-difluoro-5-(((2S,3S,4S,5S,6R)-3,4,5-trihydroxy-6-(hydroxymethyl)tetrahydro-2H-pyran-2-yl)oxy)pentanoyl)-L-phenylalaninate* (4i)** was synthesized according to the general procedure 1: Flash column chromatography purification 0-4% of MeOH in EtOAc afforded the title as a light yellow oil in 49**% (23.2 mg).** **^{1}H NMR (400 MHz, DMSO-d_6)** δ 9.11 (d, J = 8.3 Hz, 1H), 7.30 – 7.19 (m, 5H), 4.74 (d, J = 5.3 Hz, 1H), 4.72 (d, J = 4.4 Hz, 1H), 4.66 – 4.59 (m, 1H), 4.58 (d, J = 5.7 Hz, 1H), 4.56 (d, J = 1.6 Hz, 1H), 4.44 (t, J = 6.0 Hz, 1H), 3.68 – 3.63 (m, 4H), 3.60 – 3.56 (m, 1H), 3.51 (ddd, J = 9.7, 7.1, 5.6 Hz, 1H), 3.47 – 3.41 (m, 2H), 3.40 – 3.38 (m, 1H), 3.28 – 3.22 (m, 2H), 3.17 (dd, J = 13.8, 4.7 Hz, 1H), 3.01 (dd, J = 13.8, 11.0 Hz, 1H), 2.00 – 1.84 (m, 2H), 1.42 – 1.32 (m, 1H), 1.31 – 1.23 (m, 1H). **^{13}C NMR (101 MHz, DMSO-d_6)** δ 171.1, 163.5 (t, J = 29.6 Hz), 137.2, 129.1, 129.1, 128.3, 128.3, 126.6, 118.1 (t, J = 249.4 Hz), 99.7, 74.1, 71.0, 70.3, 67.0, 65.0, 61.3, 53.4, 52.3, 40.4, 35.6, 31.0 (t, J = 23.5 Hz), 21.4 (t, J = 4.5 Hz). **^{19}F NMR (377 MHz, DMSO-d_6)** δ -104.12 (d, J = 246.8 Hz, 1F), -105.60 (d, J = 246.7 Hz, 1F). **HRMS (ESI)** *m/z:* $[M+Na]^+$ Calcd. for $C_{21}H_{29}F_2NO_9Na^+$ 500.1702; Found: 500.1697. **$[\alpha]_D^{20.0}$** = +18.4 (c = 0.2 in acetone).

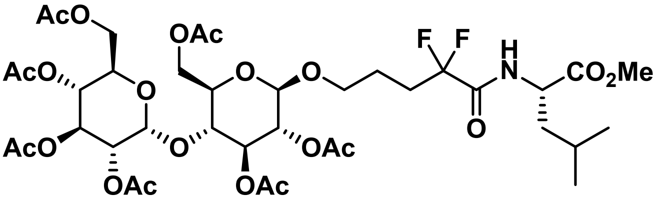

***(2R,3R,4S,5R,6R)-2-(acetoxymethyl)-6-(((2R,3R,4S,5R,6R)-4,5-diacetoxy-2-(acetoxymethyl)-6-((4,4-difluoro-5-(((S)-1-methoxy-4-methyl-1-oxopentan-2-yl)amino)-5-oxopentyl)oxy)tetrahydro-2H-pyran-3-yl)oxy)tetrahydro-2H-pyran-3,4,5-triyl triacetate* (4j)** was synthesized according to the general procedure 1: Flash column chromatography purification 20-45% of EtOAc in cyclohexane afforded the title as a colorless foam in 60**% (53.6 mg).** **^{1}H NMR (400 MHz, $CDCl_3$)** δ 6.74 (d, J = 8.4 Hz,

1H), 5.38 (d, J = 4.0 Hz, 1H), 5.37 – 5.28 (m, 1H), 5.22 (t, J = 9.1 Hz, 1H), 5.02 (t, J = 9.9 Hz, 1H), 4.85 – 4.75 (m, 2H), 4.63 – 4.54 (m, 1H), 4.51 – 4.43 (m, 2H), 4.26 – 4.14 (m, 2H), 4.05 – 3.90 (m, 3H), 3.85 (dt, J = 9.8, 5.9 Hz, 1H), 3.73 (s, 3H), 3.68 – 3.61 (m, 1H), 3.57 – 3.48 (m, 1H), 2.19 – 2.11 (m, 1H), 2.11 (s, 3H), 2.07 (s, 3H), 2.01 (s, 3H), 2.00 (d, J = 0.9 Hz, 6H), 1.97 (s, 6H), 1.86 – 1.78 (m, 1H), 1.76 – 1.68 (m, 2H), 1.67 – 1.55 (m, 3H), 0.95 – 0.89 (m, 6H). **^{13}C NMR (101 MHz, CDCl$_3$)** δ 172.4, 170.6, 170.6, 170.3, 170.1, 169.8, 169.5, 164.0 (t, J = 29.2 Hz), 118.0 (t, J = 252.2 Hz), 100.3, 95.6, 75.4, 72.7, 72.2, 72.2, 70.1, 69.4, 68.8, 68.6, 68.1, 62.8, 61.6, 52.7, 52.6, 50.8, 41.3, 30.5 (t, J = 23.6 Hz), 24.9, 22.8, 21.9, 21.0, 20.9, 20.8, 20.7, 20.7. **^{19}F NMR (377 MHz, CDCl$_3$)** δ -105.20 (d, J = 254.9 Hz, 1F), -107.52 (d, J = 255.0 Hz, 1F). **HRMS (ESI)** *m/z:* [M+Na]$^+$ Calcd. for $C_{38}H_{55}F_2NO_{21}Na^+$ 922.3127; Found: 922.3121. **Melting point:** 50.9 – 52.7 °C. **[α]$_D^{20.0}$** = +28.1 (c = 0.2 in acetone).

7. Characterization data of compounds 5a-5c

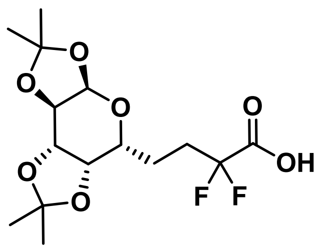

2,2-difluoro-4-((3aR,5R,5aS,8aS,8bR)-2,2,7,7-tetramethyltetrahydro-5H-bis([1,3]dioxolo)[4,5-b:4',5'-d]pyran-5-yl)butanoic acid **(5a)** was synthesized according to the general procedure 1: Flash column chromatography purification using a mixture of 1% Acetic acid, 20% EtOAC, 79% hexanes afforded the title as a white solid in 70**% (24.65 mg). ^{1}H NMR (400 MHz, DMSO-*d*$_6$)** δ 5.43 (d, J = 5.0 Hz, 1H), 4.56 (dd, J = 7.9, 2.3 Hz, 1H), 4.32 (dd, J = 5.1, 2.3 Hz, 1H), 4.14 (dd, J = 7.9, 1.8 Hz, 1H), 3.70 – 3.63 (m, 1H), 2.25 – 2.10 (m, 1H), 2.07 – 1.93 (m, 1H), 1.69 – 1.53 (m, 2H), 1.43 (s, 3H), 1.34 (s, 3H), 1.27 (d, J = 6.8 Hz, 6H). **^{13}C NMR (101 MHz, DMSO-*d*$_6$)** δ 165.1 (t, J = 31.4 Hz), 116.6 (t, J = 248.4 Hz), 108.1, 107.7, 95.6, 71.7, 70.1, 69.6, 65.9, 30.2 (t, J = 23.3 Hz), 25.8, 25.6, 24.8, 24.2, 22.3. **^{19}F NMR (377 MHz, DMSO-*d*$_6$)** δ -104.92 (d, J = 250.4 Hz, 1F), -105.78 (d, J = 250.3 Hz, 1F). **HRMS (ESI)** *m/z:* [M-H]$^+$ Calcd. for $C_{15}H_{21}F_2O_7^-$ 351.1261; Found: 351.1258. **[α]$_D^{20.0}$** = −31.4 (c = 0.2 in acetone).

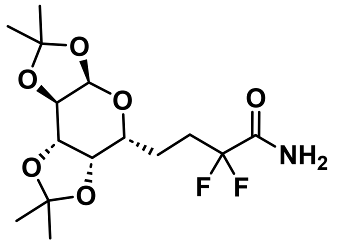

2,2-difluoro-4-((3aR,5R,5aS,8aS,8bR)-2,2,7,7-tetramethyltetrahydro-5H-bis([1,3]dioxolo)[4,5-b:4',5'-d]pyran-5-yl)butanamide **(5b)** was synthesized according to the general procedure 1: Flash column chromatography purification 15-30% of EtOAc in cyclohexane afforded the title white solid in 76**% (26.8 mg).** **^{1}H NMR (600 MHz, Acetone-d$_6$)** δ 7.56 (s, 1H), 7.17 (s, 1H), 5.45 (d, *J* = 5.1 Hz, 1H), 4.60 (dd, *J* = 7.9, 2.4 Hz, 1H), 4.32 (dd, *J* = 5.1, 2.3 Hz, 1H), 4.19 (dd, *J* = 7.9, 1.9 Hz, 1H), 3.78 (ddd, *J* = 9.4, 4.1, 1.8 Hz, 1H), 2.35 – 2.21 (m, 1H), 2.15 – 2.05 (m, 1H), 1.83 – 1.75 (m, 1H), 1.70 – 1.63 (m, 1H), 1.47 (s, 3H), 1.37 (s, 3H), 1.31 (d, *J* = 4.6 Hz, 6H). **^{13}C NMR (151 MHz, Acetone-d$_6$)** δ 166.6 (t, *J* = 29.3 Hz), 119.2 (t, *J* = 250.9 Hz), 109.4, 108.8, 97.3, 73.4, 71.8, 71.3, 67.5, 31.4 (t, *J* = 23.8 Hz), 26.3, 26.2, 25.1, 24.6, 23.5 (t, *J* = 4.7 Hz). **^{19}F NMR (377 MHz, Acetone-d$_6$)** δ -106.67 (d, *J* = 250.8 Hz, 1F), -106.69 (d, *J* = 250.9 Hz, 1F). **HRMS (ESI)** *m/z:* [M+Na]$^+$ Calcd. for $C_{15}H_{23}F_2NO_6Na^+$ 374.1385; Found: 374.1382. **Melting point:** 172 – 173.4 °C. **$[\alpha]_D^{20.0}$** = –45.7 (*c* = 0.2 in acetone).

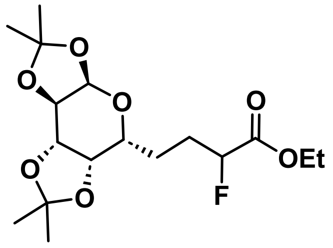

Ethyl 2-fluoro-4-((3aR,5R,5aS,8aS,8bR)-2,2,7,7-tetramethyltetrahydro-5H-bis([1,3]dioxolo)[4,5-b:4',5'-d]pyran-5-yl)butanoate **(5c)** was synthesized according to the general procedure 1: Flash column chromatography purification 5-15% of EtOAc in cyclohexane afforded the title light yellow oil in 70**% (25.43 mg).** **^{1}H NMR (600 MHz, Acetone-d$_6$)** δ 5.46 (d, *J* = 5.0 Hz, 1H), 5.10 – 4.98 (m, 1H), 4.60 (dt, *J* = 7.9, 2.5 Hz, 1H), 4.32 (dt, *J* = 5.0, 2.4 Hz, 1H), 4.25 – 4.19 (m, 2H), 4.17 (ddd, *J* = 7.9, 2.0, 0.8 Hz, 1H), 3.79 – 3.75 (m, 1H), 2.14 – 2.06 (m, 0.5H), 2.03 – 1.91 (m, 1H), 1.90 – 1.83 (m, 0.5H), 1.81 – 1.73 (m, 1H), 1.69 – 1.61 (m, 1H), 1.47 (d, *J* = 3.2 Hz, 3H), 1.37 (d, *J* = 2.5 Hz, 3H), 1.31 (d, *J* = 2.1 Hz, 6H), 1.26 (td, *J* = 7.1, 2.3 Hz, 3H). **^{13}C NMR (151 MHz, Acetone-d$_6$)** δ 170.2 (dd, *J* = 23.9, 5.4 Hz), 109.4, 109.4, 108.8, 108.8, 97.4, 97.3, 89.6 (dd, *J* = 181.6, 55.9 Hz), 73.5, 73.4, 71.8, 71.8, 71.3, 67.8, 67.3, 61.7, 61.7, 29.3, 26.4, 26.2, 26.2, 25.9 (dd, *J* = 72.5, 3.3 Hz), 25.12, 25.10, 24.64, 24.62, 14.42. **^{19}F NMR (377 MHz, Acetone-d$_6$)** δ -192.56 (s, 1F), -193.09 (s, 1F). **HRMS (ESI)** *m/z:* [M+NH$_4$]$^+$ Calcd. for $C_{17}H_{27}FO_7NH_4^+$ 380.2079; Found: 380.2080.

8. General procedure 2: Synthesis of sugar olefins 1a-1k

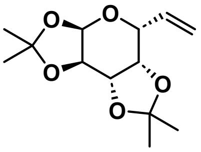

1a

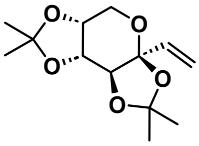

1b

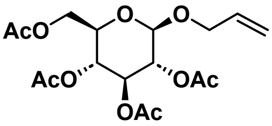

1c

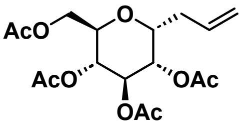

1d

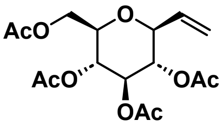

1e

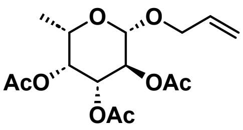

1f

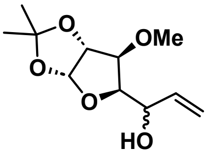

1g

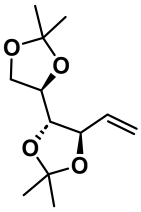

1h

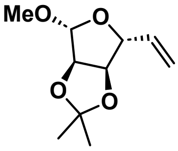

1i

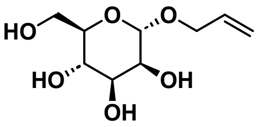

1j

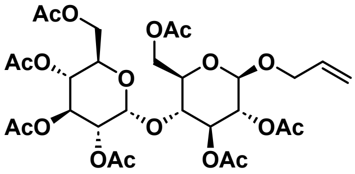

1k

The sugar olefins **1a** and **1b** are known compounds and were prepared according to the following protocol sequence:

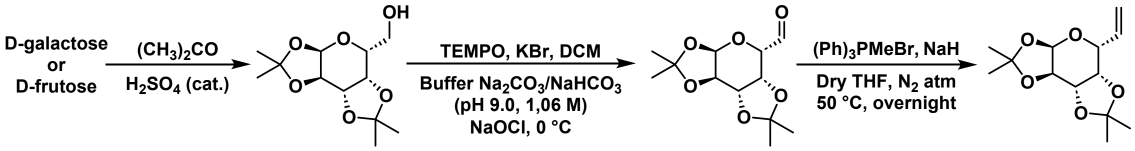

The protected sugars were obtained using McDougall's protocol.[2]

The corresponding aldehydes were synthetized following Iida's method.[3]

The corresponding olefins were prepared using the Wittig reaction, as reported by Ling.[4]

Note: Compounds **1c**, **1f**, **1j** and **1k**[5]; **1d**[6]; **1e**[7]; **1g**[8]; **1h**[9]; and **1i**[10] are known compounds and were prepared according to the literature.

9. General procedure 3: Synthesis of 1-bromo-1,1-difluoroacetamides 2a-2i and 2m

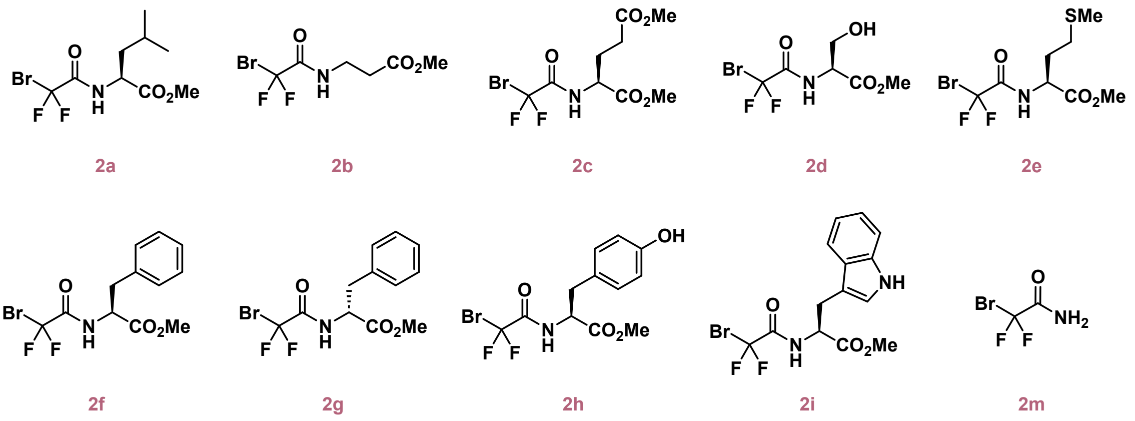

Structures of 1-bromo-1,1-difluoroacetamides 2a-2i and 2m

Note: Compounds **2a**[11], **2d**[11], **2e**[12], **2f**[11], **2h**[11], **2i**[13] and **2m**[14] are known compounds and were prepared according to the literature.

The unknown bromides were synthetized by an adaptation on the protocol reported by Sureshkumar, 2023.[15]

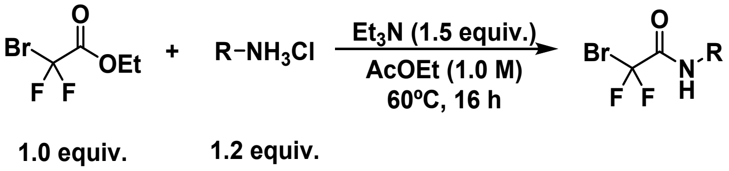

A typical procedure for preparing bromides **2** is as follows: a round bottom flask was charged with the corresponding hydrochloride salt of amino acid (1.2 equiv.). Then, EtOAC (1.0 M), ethyl bromodifluoroacetate (1.0 equiv.), and trimethylamine (1.5 equiv.) were added. The reaction was stirred at 60 °C overnight. The reaction mixture was diluted with EtOAc and washed with water (2x) and brine. The organic layer was dried over Na_2SO_4, filtered, and concentrated. The residue was purified with silica gel chromatography (Hexane/Ethyl Acetate) to give the corresponding 1-bromo-1,1-difluoroacetamides 2.

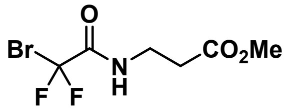

Methyl 3-(2-bromo-2,2-difluoroacetamido)propanoate **(2b)** was synthesized according to the general procedure 3: Flash column chromatography purification 0-10% of EtOAc in hexanes afforded the title as a colorless liquid in 53**% (206 mg).** **^{1}H NMR (400 MHz, CDCl$_3$)** δ 7.01 (s, 1H), 3.73 (s, 3H), 3.62 (q, J = 6.0 Hz, 2H), 2.62 (t, J = 5.9 Hz, 2H). **^{13}C NMR (101 MHz, CDCl$_3$)** δ 172.8, 160.1 (t, J = 27.6 Hz), 111.8 (t, J = 316.0 Hz), 52.3, 35.6, 33.0. **^{19}F NMR (376 MHz, CDCl$_3$)** δ -60.70, 2F. **HRMS (ESI)** *m/z:* [M-H]$^-$ Calcd. for $C_6H_7BrF_2NO_3$ 257.9583; Found: 257.9583.

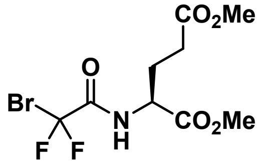

Dimethyl (2-bromo-2,2-difluoroacetyl)-L-glutamate **(2c)** was synthesized according to the general procedure 3: Flash column chromatography purification 0-5% of EtOAc in hexanes afforded the title as a colorless liquid in 44**% (217 mg).** **^{1}H NMR (400 MHz, CDCl$_3$)** δ 7.47 (d, J = 7.8 Hz, 1H), 4.56 (td, J = 7.9, 5.0 Hz, 1H), 3.74 (s, 3H), 3.64 (s, 3H), 2.47 – 2.31 (m, 2H), 2.31 – 2.18 (m, 1H), 2.13 – 2.04 (m, 1H). **^{13}C NMR (101 MHz, CDCl$_3$)** δ 173.3, 170.8, 160.0 (t, J = 28.2 Hz), 111.3 (t, J = 315.9 Hz), 52.9, 52.4, 52.1, 29.9, 26.5. **^{19}F NMR (376 MHz, CDCl$_3$)** δ -60.99, 2F. **HRMS (ESI)** *m/z:* [M+Na]$^+$ Calcd. for $C_9H_{12}BrF_2NO_5Na^+$ 353.9759; Found: 353.9754.

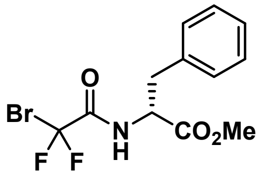

Methyl (2-bromo-2,2-difluoroacetyl)-D-phenylalaninate **(2g)** was synthesized according to the general procedure 3: Flash column chromatography purification 0-5% of EtOAc in hexanes afforded the title as a white solid in 38**% (191.27 mg).** **^{1}H NMR (400 MHz, CDCl$_3$)** δ 7.32 – 7.13 (m, 3H), 7.02 (d, J = 7.5 Hz, 2H), 6.74 (d, J = 6.2 Hz, 1H), 4.78 (q, J = 6.2 Hz, 1H), 3.69 (s, 3H), 3.17 (dd, J = 14.0, 5.6 Hz, 1H), 3.08 (dd, J = 14.0, 5.8 Hz, 1H). **^{13}C NMR (101 MHz, CDCl$_3$)** δ 170.6, 159.4 (t, J = 28.2 Hz), 134.8, 129.3, 128.9, 127.6, 111.3 (t, J = 315.9 Hz), 53.8, 52.9, 37.4. **^{19}F NMR (376 MHz, CDCl$_3$)** δ -60.79 (d, J = 4.8 Hz, 2F). **HRMS (ESI)** *m/z:* [M-H]$^-$ Calcd. for $C_{12}H_{11}BrF_2NO_3$ 333.9896; Found: 333.9890. **Melting point:** 60.1 – 60 .7 °C.

10. General procedure 4: Synthesis of 1-bromo-1,1-difluoroacetamides 2j-2k.

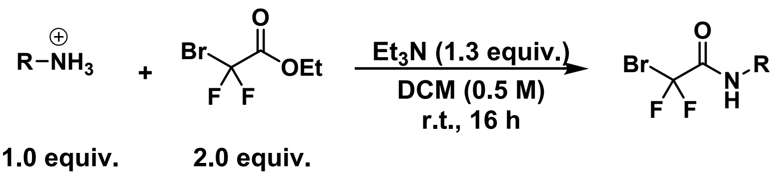

In a round bottom-flask, with a magnetic stirring bar, the correspondent amine (1.0 equiv) and DCM (0.5 M) was added, followed by addition of ethyl 2-bromo-2,2-difluoroacetate (2.0 equiv). The reaction was placed in an ice bath and triethylamine (1.3 equiv.) was added. The resulting solution was left to at room temperature and stirred overnight. Then, the solution was diluted with DCM, and the combined organic phases were washed with water, 10% HCl, $NaHCO_3$ sat., brine, dried over Na_2SO_4, filtered, concentrated in vacuo and purified *via* silica gel column chromatography, if necessary.

11. Characterization data of compounds 2j-2k.

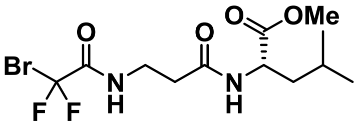

Methyl (3-(2-bromo-2,2-difluoroacetamido)propanoyl)-L-leucinate **(2j)** was synthesized according to the general procedure 4 afforded the title as a beige solid in 73% (251.91 mg). Purification was not necessary. Rf = 0.5 - 0.6 (hexanes/EtOAc, 6:4**). ^{1}H NMR (400 MHz, $CDCl_3$)** δ 7.65 – 7.51 (m, 1H), 6.38 (d, J = 8.2 Hz, 1H), 4.53 (td, J = 8.6, 4.5 Hz, 1H), 3.67 (s, 3H), 3.64 – 3.49 (m, 2H), 2.48 (t, J = 5.9 Hz, 2H), 1.64 – 1.42 (m, 3H), 0.87 (d, J = 5.7 Hz, 6H). **^{13}C NMR (101 MHz, $CDCl_3$)** δ 173.5, 171.2, 160.2 (t, J = 27.5 Hz), 111.7 (t, J = 316.1 Hz), 52.5, 50.9, 41.2, 36.2, 34.3, 24.9, 22.7, 21.8. **^{19}F NMR (377 MHz, $CDCl_3$)** δ -60.66. **HRMS (ESI)** *m/z:* $[M+Na]^+$ Calcd. For $C_{12}H_{19}BrF_2N_2O_4Na^+$ 395.0388; Found: 395.0396. **Melting point:** 94.7 – 96.6 °C.

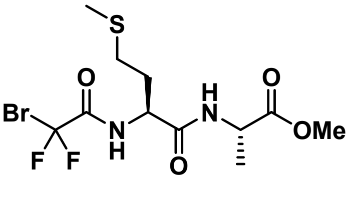

Methyl (2-bromo-2,2-difluoroacetyl)-L-methionyl-L-alaninate **(2k)** was synthesized according to the general procedure 4: Flash column chromatography purification 15-40% of EtOAc in hexanes afforded the title as a white solid in 36% (210.0 mg). Purification was not necessary. R_f = 0.4 - 0.5 (hexanes/EtOAc,

4:6). **^{1}H NMR (400 MHz, CDCl$_3$)** δ 7.58 (d, J = 7.8 Hz, 1H), 7.02 (d, J = 7.4 Hz, 1H), 4.73 (q, J = 7.0 Hz, 1H), 4.56 (p, J = 7.3 Hz, 1H), 3.75 (s, 3H), 2.63 (td, J = 7.3, 2.1 Hz, 2H), 2.14 – 2.06 (m, 5H), 1.42 (d, J = 7.2 Hz, 3H). **^{13}C NMR (101 MHz, CDCl$_3$)** δ 172.8, 169.3, 159.8 (t, J = 28.2 Hz), 111.3 (t, J = 316.2 Hz), 52.6, 52.3, 48.3, 31.3, 29.5, 17.9, 15.0. **^{19}F NMR (376 MHz, CDCl$_3$)** δ -60.81. **HRMS (ESI)** *m/z:* $[M+Na]^+$ Calcd. For $C_{11}H_{17}BrF_2N_2O_4SNa^+$ 412.9953; Found: 412.9954. **Melting point:** 89.9 – 91.9 °C.

12. Scale-Up Reaction

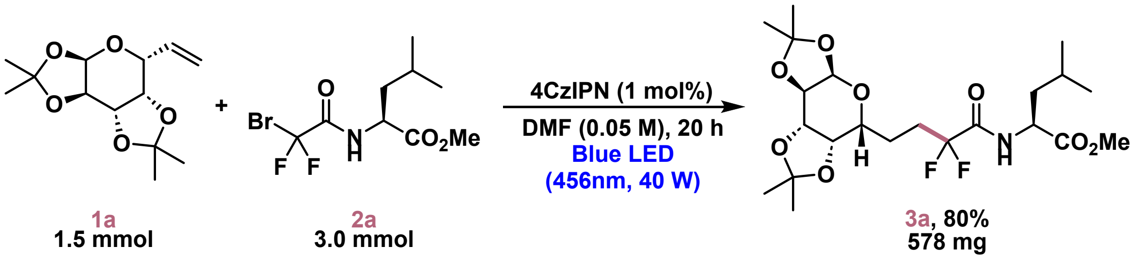

An oven-dried 100 mL schlenk flask was charged with **1a** (0.15 mmol, 1.0 equiv.), **2a** (0.3 mmol, 1.5 equiv.), 4CzIPN (1 mol%) and a magnetic stir bar. After the addition of DMF (0.05 M), the flask was closed with a rubber septum and argon was bubbled through the content over 10 min. Then, the flask was placed in front of a Blue LED lamp (Kessil 456 nm – 3 cm distance) equipped with a fan, and the reaction mixture was stirred for 20 h. Once the time has passed, the reaction mixture was diluted with EtOAc and washed with H_2O (3 x 50 mL), brine (3 x 50 mL), dried over anhydrous Na_2SO_4, and concentrated under reduced pressure. Purified by chromatography column (EtOAc/Cyclohexane) afforded the product as a pale yellow oil in 80% **(578 mg).**

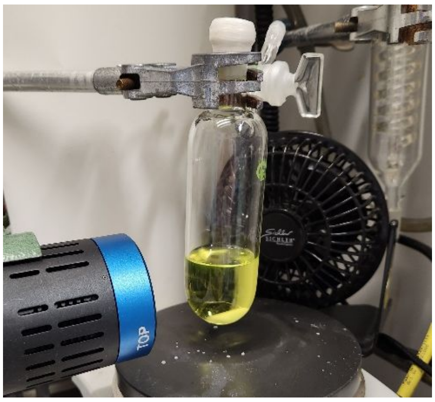

Figure S2. Representative picture of reaction scale-up.

13. Mechanistical Studies

- **Deuteration experiment**

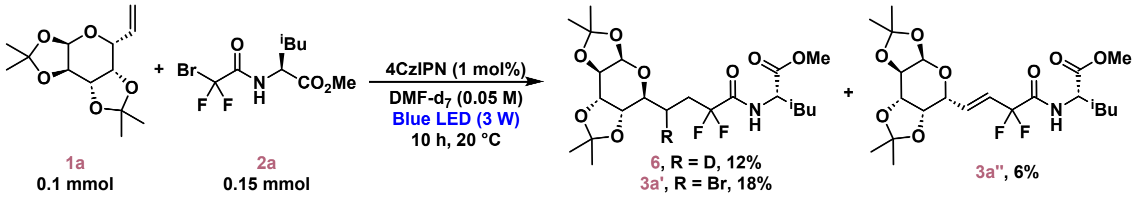

A 5 mL crimp cap reaction vial was charged with **1a** (0.1 mmol, 1.0 equiv.), **2a** (0.15 mmol, 1.5 equiv.), 4CzIPN (1 mol%) and a magnetic stir bar. After the addition of DMF-d_7 (0.05 M), the crimp vial was capped and argon was bubbled through the content over 10 min. Then, the vial was placed in a thermostatic cooling block (20 °C) and irradiated through the panel bottom side of the vial by a 450-456 nm LED (3 W). Once the time has passed, the reaction mixture was diluted with EtOAc and washed with H_2O (2 x 25 mL), brine (2 x 25 mL), dried over anhydrous Na_2SO_4 and concentrated under reduced pressure. **^{1}H NMR (400 MHz, Acetone-*d*$_6$)** δ 8.10 (d, *J* = 8.3 Hz, 1H), 5.46 (d, *J* = 5.0 Hz, 1H), 4.61 (dd, *J* = 7.9, 2.3 Hz, 1H), 4.57 – 4.50 (m, 1H), 4.32 (dd, *J* = 5.1, 2.3 Hz, 1H), 4.18 (dd, *J* = 7.9, 1.9 Hz, 1H), 3.80 – 3.74 (m, 1H), 3.70 (s, 3H), 2.38 – 2.21 (m, 1H), 2.17 – 2.08 (m, 1H), 1.83 – 1.60 (m, 4H), 1.47 (s, 3H), 1.37 (s, 3H), 1.31 (d, *J* = 3.5 Hz, 6H), 0.96 – 0.90 (m, 6H). **^{13}C NMR (101 MHz, Acetone-*d*$_6$)** δ 172.8, 165.0 (t, *J* = 29.4 Hz), 119.3 (t, *J* = 250.7 Hz), 109.5, 108.9, 97.3, 73.4, 73.4, 71.8, 71.3, 67.5, 67.5, 52.5, 51.6, 40.5, 31.6 (t, *J* = 23.7 Hz), 26.4, 26.3, 25.5, 25.1, 24.6, 23.5 (t, *J* = 4.7 Hz), 23.2, 21.5. **^{19}F NMR (377 MHz, Acetone-*d*$_6$)** δ -106.35 (dd, *J* = 250.9, 6.8 Hz), -107.66 (dd, *J* = 250.5, 3.7 Hz). **HRMS (ESI)** *m/z:* $[M+Na]^+$ Calcd. For $C_{22}H_{34}DF_2NO_8Na^+$ 503.2286; Found: 503.2277. **$[\alpha]_D^{20.0}$** = −49.0 (*c* = 0.2 in acetone).

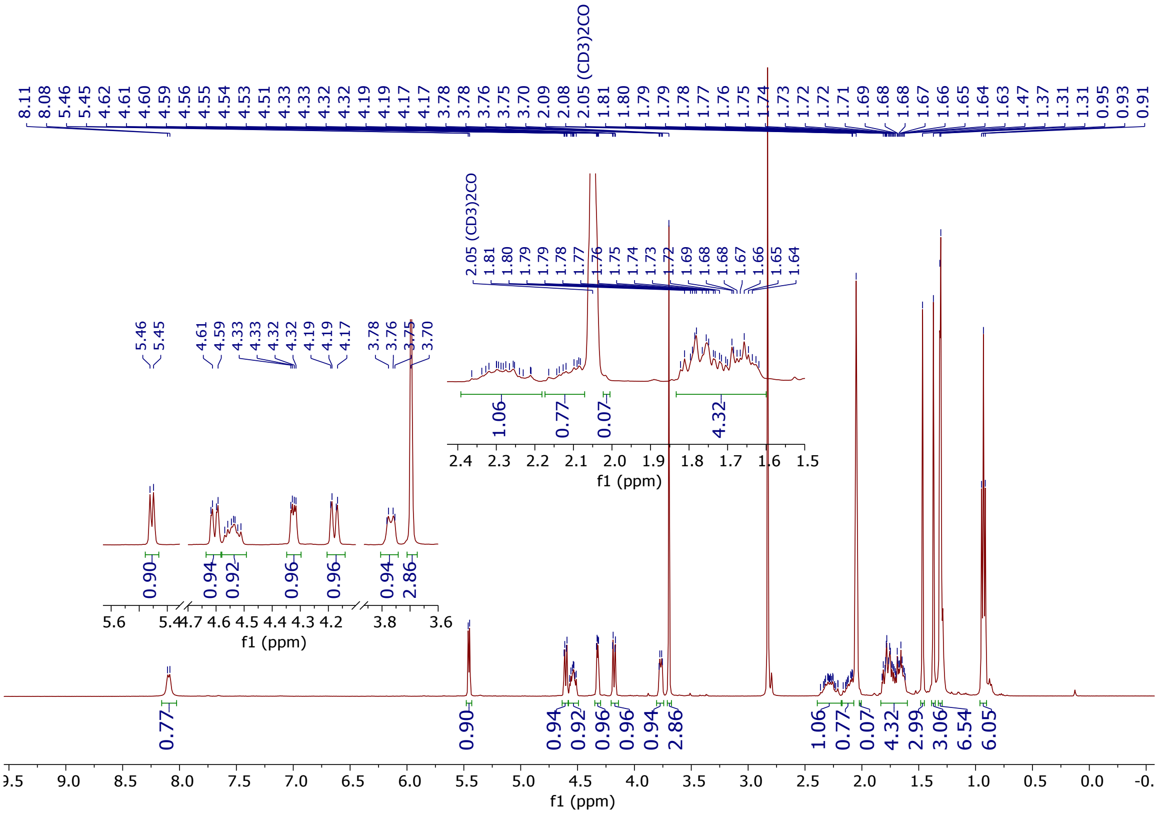

^{1}H NMR (400 MHz, Acetone-d_6) of compound 6

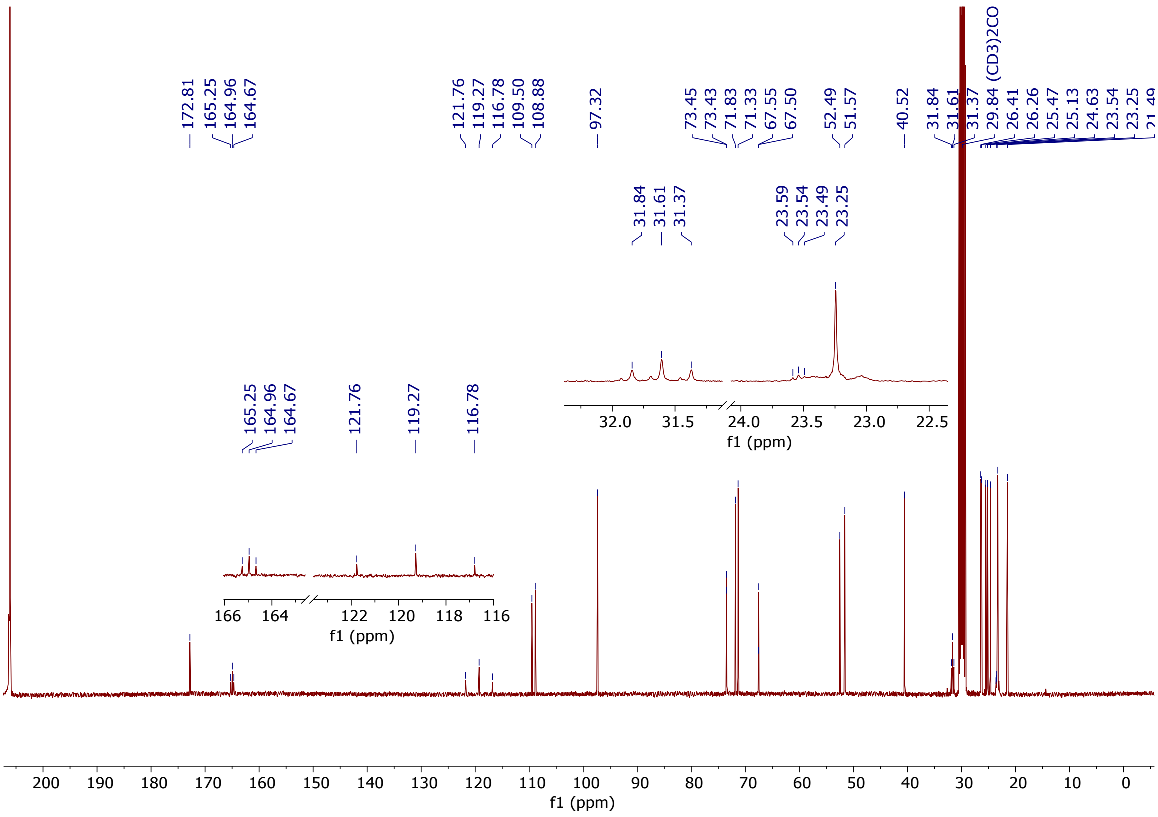

^{13}C NMR (101 MHz, Acetone-d_6) of compound 6

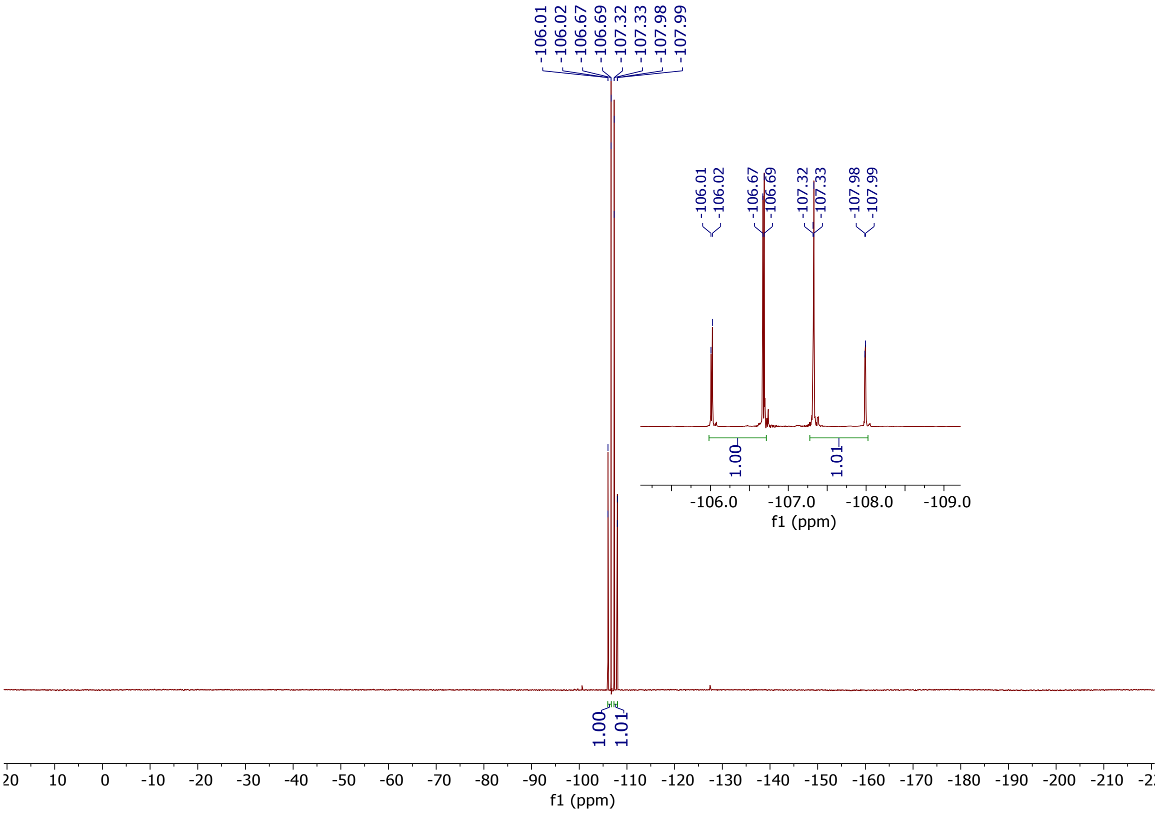

^{19}F NMR (376 MHz, Acetone-d_6) of compound 6

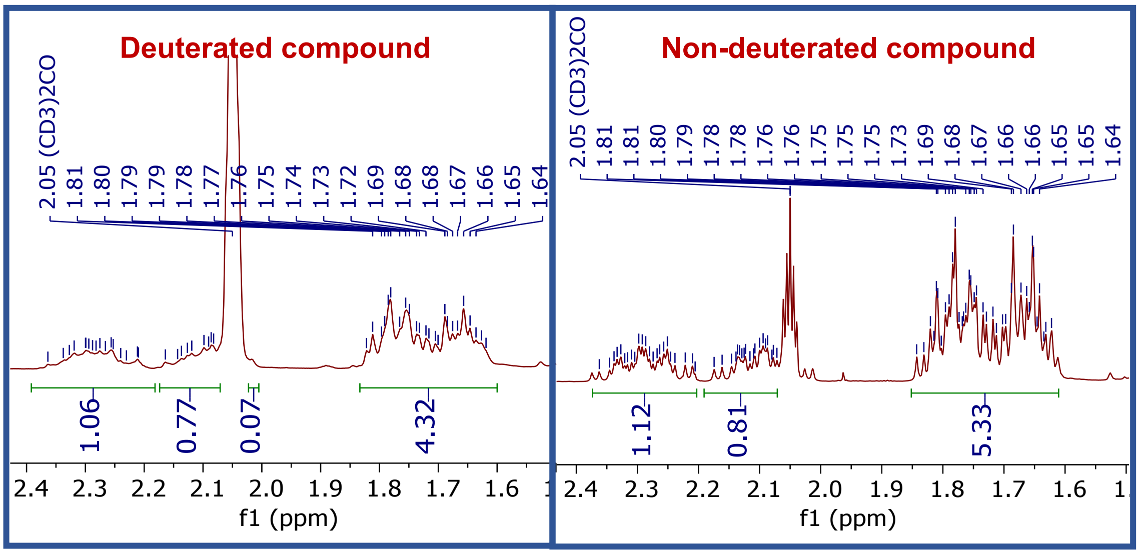

Figure S3. ^{1}H NMR comparation of deuterated (**5**) and non-deuterated (**3a**) compounds.

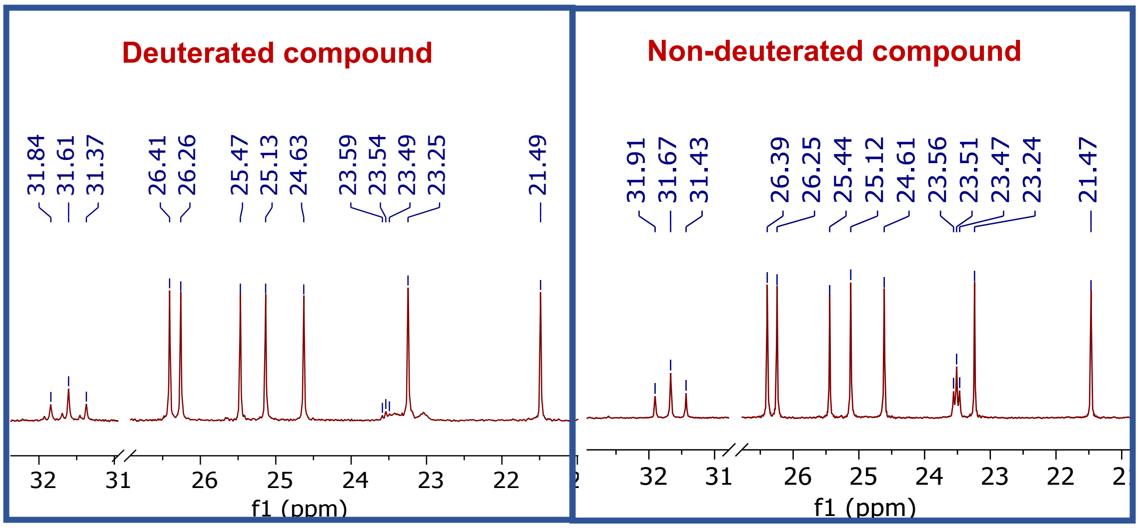

Figure S4. ^{13}C NMR comparation of deuterated (**5**) and non-deuterated (**3a**) compounds.

- **Radical trapping**

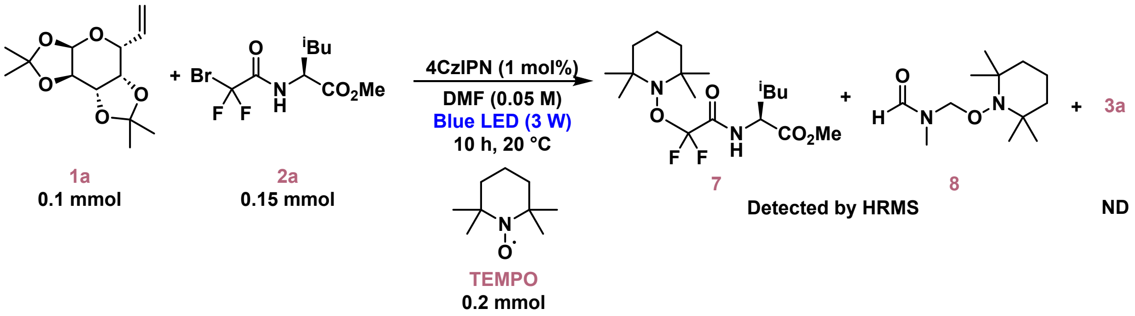

A 5 mL crimp cap reaction vial was charged with **1a** (0.1 mmol, 1.0 equiv.), **2a** (0.15 mmol, 1.5 equiv.), 4CzIPN (1 mol%), TEMPO (0.2 mmol, 2 equiv.) and a magnetic stir bar. After the addition of DMF (0.05 M), the crimp vial was capped and argon was bubbled through the content over 10 min. Then, the vial was placed in a thermostatic cooling block (20 °C) and irradiated through the panel bottom side of the vial by a 450-456 nm LED (3 W). Once the time has passed, the reaction mixture was diluted with EtOAc and washed with H_2O (2 x 25 mL), brine (2 x 25 mL), dried over anhydrous Na_2SO_4 and concentrated under reduced pressure. The crude material was analyzed by LC/MS, HRMS and ^{19}F NMR.

Compound **7** could be detected by ^{19}F NMR analysis of the crude of the reaction and the spectrum is show bellow: **^{19}F NMR (377 MHz, Acetone-*d*$_6$)** δ -74.94 (d, J = 139.8 Hz), -75.92 (d, J = 139.9 Hz).

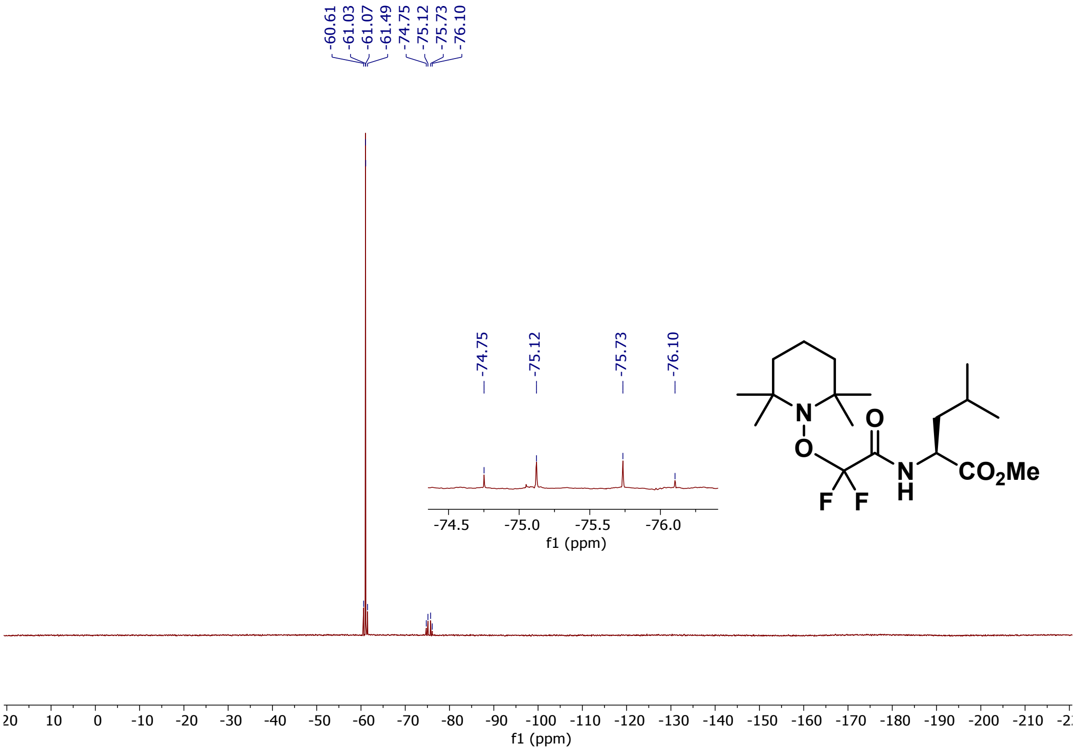

^{19}F NMR (377 MHz, Acetone-d_6) of crude reaction of TEMPO trapping

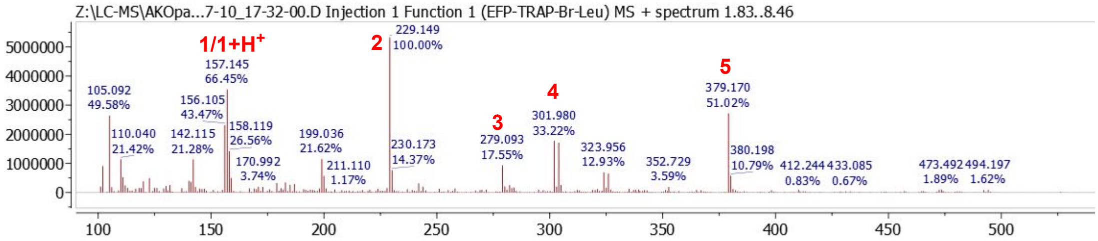

1

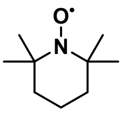

Chemical Formula: $C_9H_{18}NO^{\bullet}$
Exact Mass: 156,1388

2

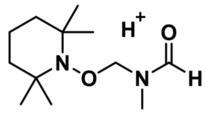

Chemical Formula: $C_{12}H_{25}N_2O_2^+$
Exact Mass: 229,1911

3

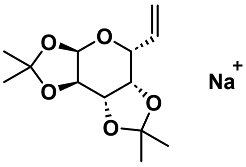

Chemical Formula: $C_{13}H_{20}NaO_5^+$
Exact Mass: 279,1203

4

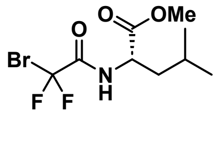

Chemical Formula: $C_9H_{14}BrF_2NO_3$
Exact Mass: 301,0125

5

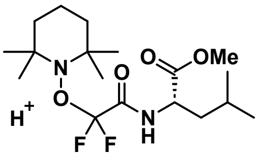

Chemical Formula: $C_{18}H_{33}F_2N_2O_4^+$
Exact Mass: 379,2403

6

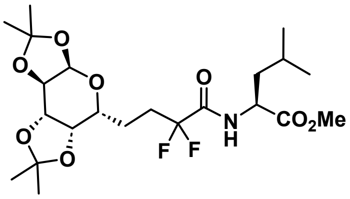

Chemical Formula: $C_{22}H_{35}F_2NO_8$
Exact Mass: 479,2331

Figure S5. Detected compounds in trapping experiment.

- **UV-Vis Analysis**

0.1 M solutions of **1a**, **2a**, 4CzIPN, and the equimolar mixtures of **1a** + 4CzIPN, **2a** + 4CzIPN and **1a** + **2a** + 4CzIPN were prepared, as shown by figure S2 and any shift could be observed.

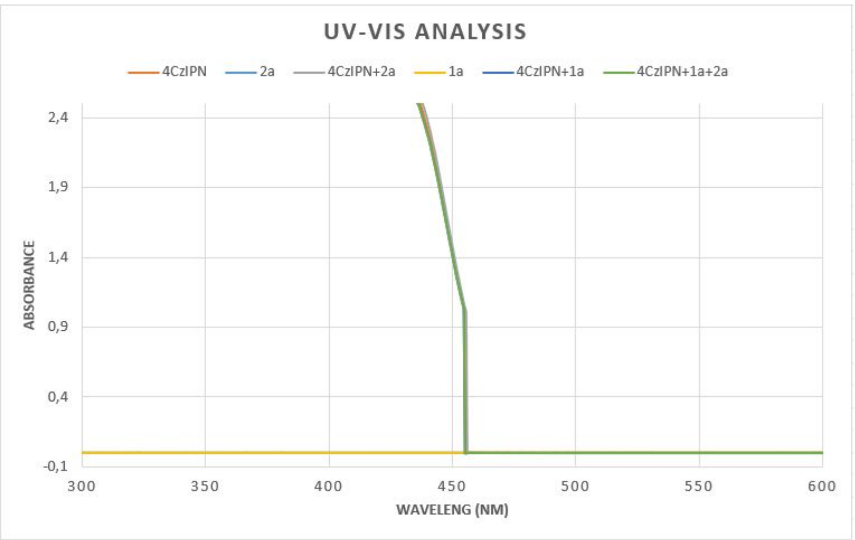

Figure S6. UV-Visible spectra.

- **Light On/Off Experiment**

A 5 mL crimp cap reaction vial was charged with **1a** (0.1 mmol, 1.0 equiv.), **2a** (0.15 mmol, 1.5 equiv.), 4CzIPN (1 mol%), $PhCF_3$ (1 equiv., NMR standard) and a magnetic stir bar. After the addition of DMF (0.05 M), the crimp vial was capped and argon was bubbled through the content over 10 min. Then, the vial was placed in a thermostatic cooling block (20 °C) and irradiated through the panel bottom side of the vial by a 450-456 nm LED (3 W). Aliquots were collected every 30 minutes to monitor the reaction by ^{19}F NMR.

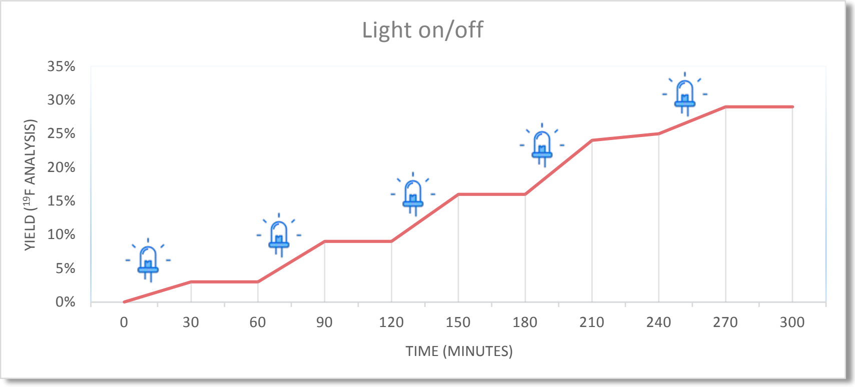

Figure S7. Light on/off experiment.

Table S1. Data obtained from light on/off experiment.

**Time (minutes)**	**Light**	**Integral**	**Yield (^{19}F analysis)**
0	on	0	**0%**
30		3,34	**3%**
60	off	3,31	**3%**
90	on	8,84	**9%**
120	off	9,46	**9%**
150	on	15,78	**16%**
180	off	15,53	**16%**
210	on	23,65	**24%**
240	off	24,6	**25%**
270	on	28,6689	**29%**
300	off	29,38	**29%**

14. Characterization Spectra of compounds 3a-3

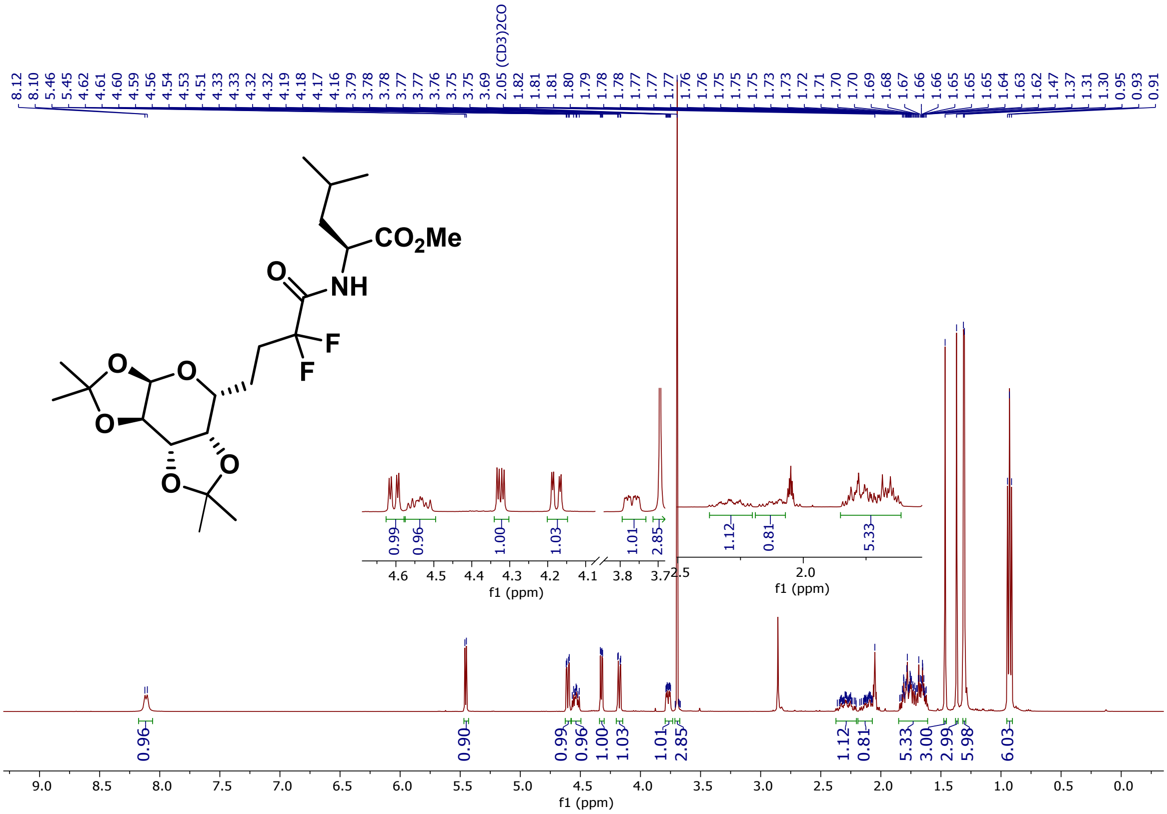

^{1}H NMR (400 MHz, acetone-d$_6$) of compound 3a

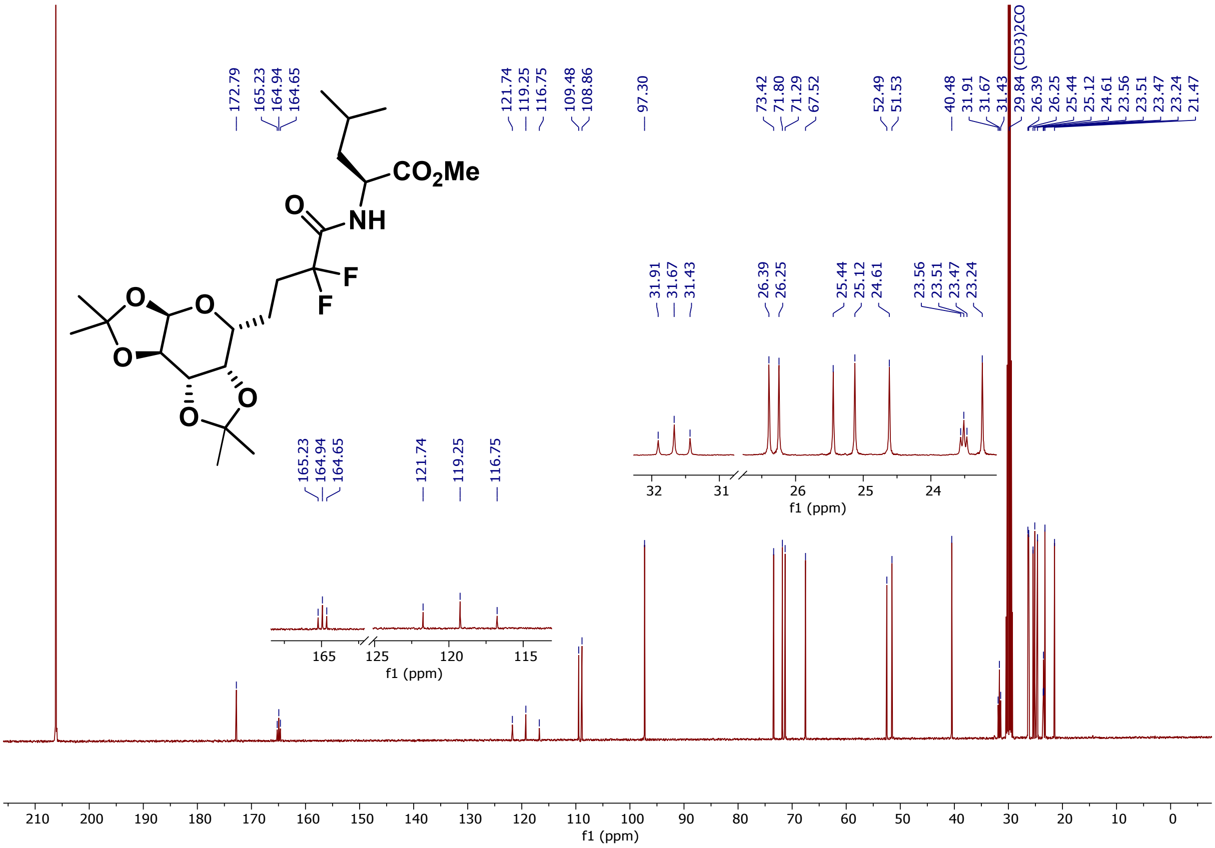

^{13}C NMR (101 MHz, acetone-d$_6$) of compound 3a

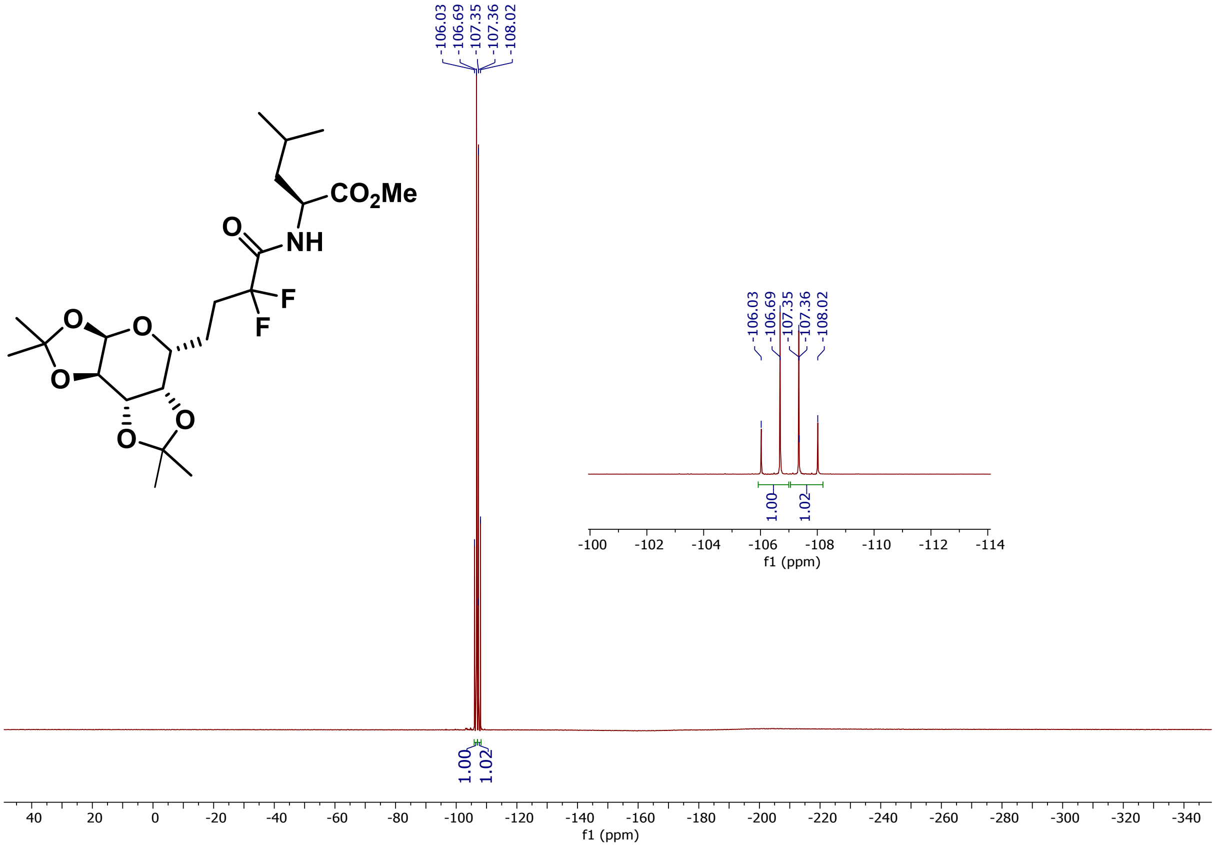

^{19}F NMR (376 MHz, acetone-d$_6$) of compound 3a

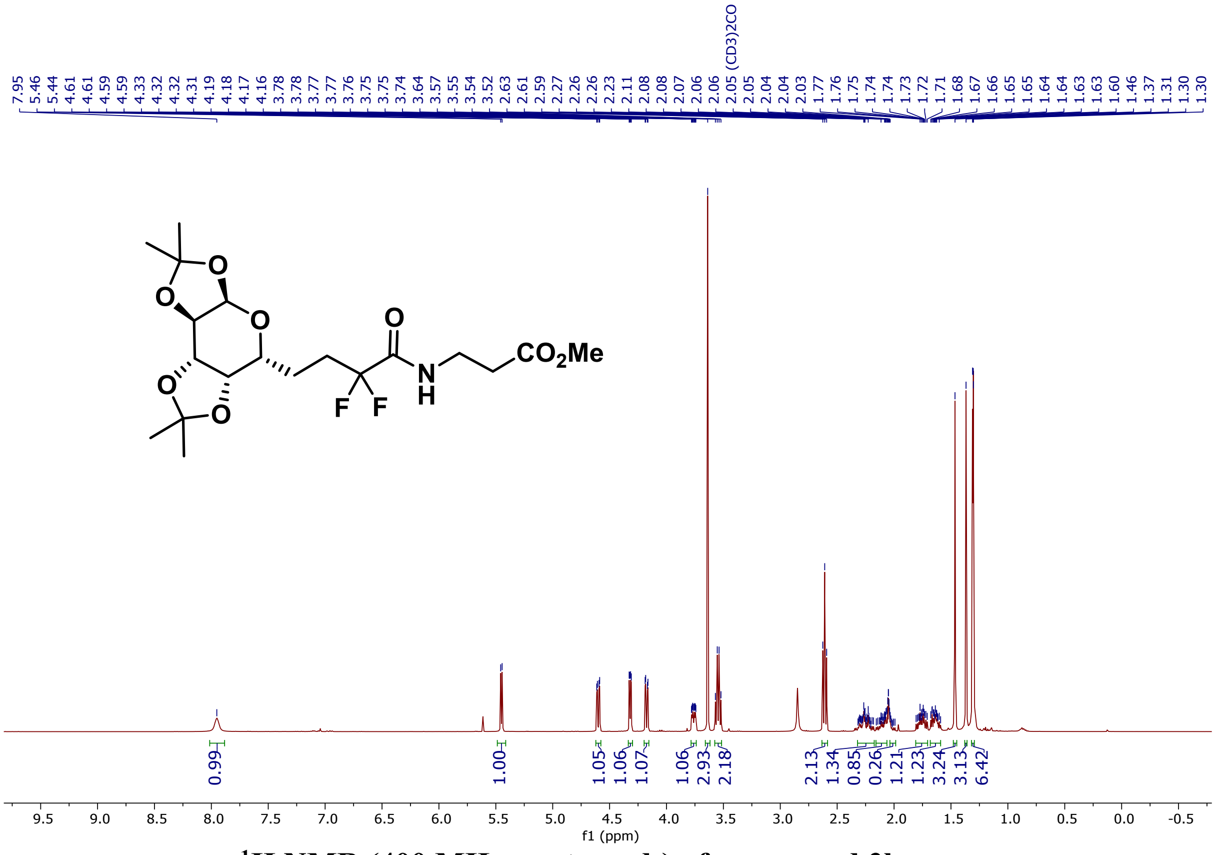

^{1}H NMR (400 MHz, acetone-d$_6$) of compound 3b

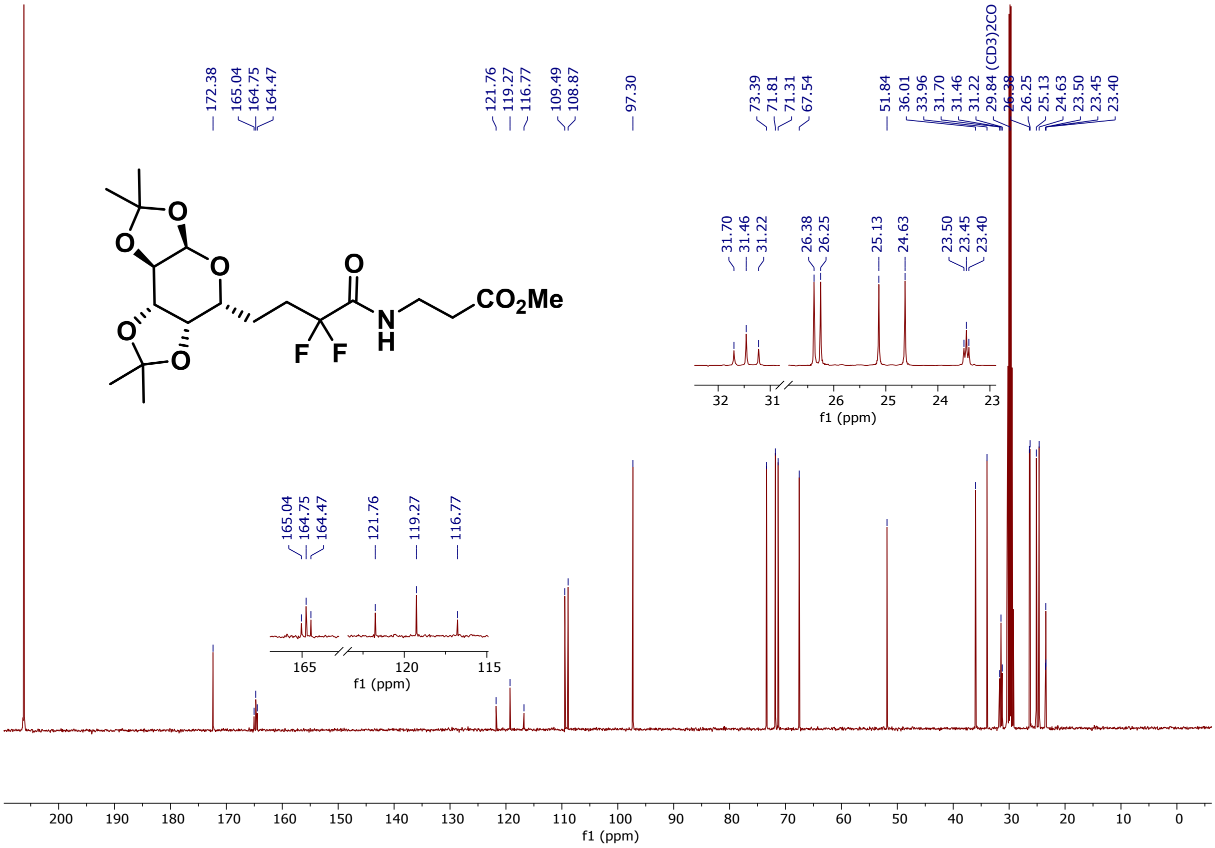

^{13}C NMR (101 MHz, acetone-d$_6$) of compound 3b

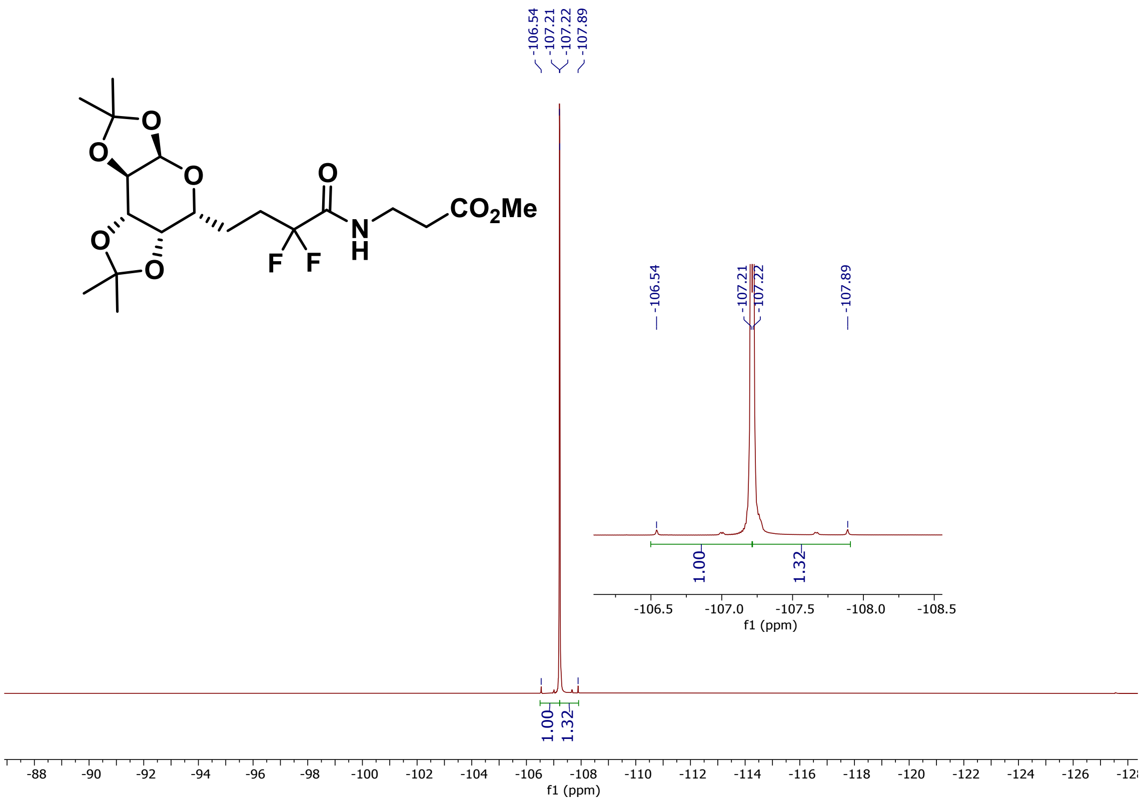

^{19}F NMR (376 MHz, acetone-d$_6$) of compound 3b

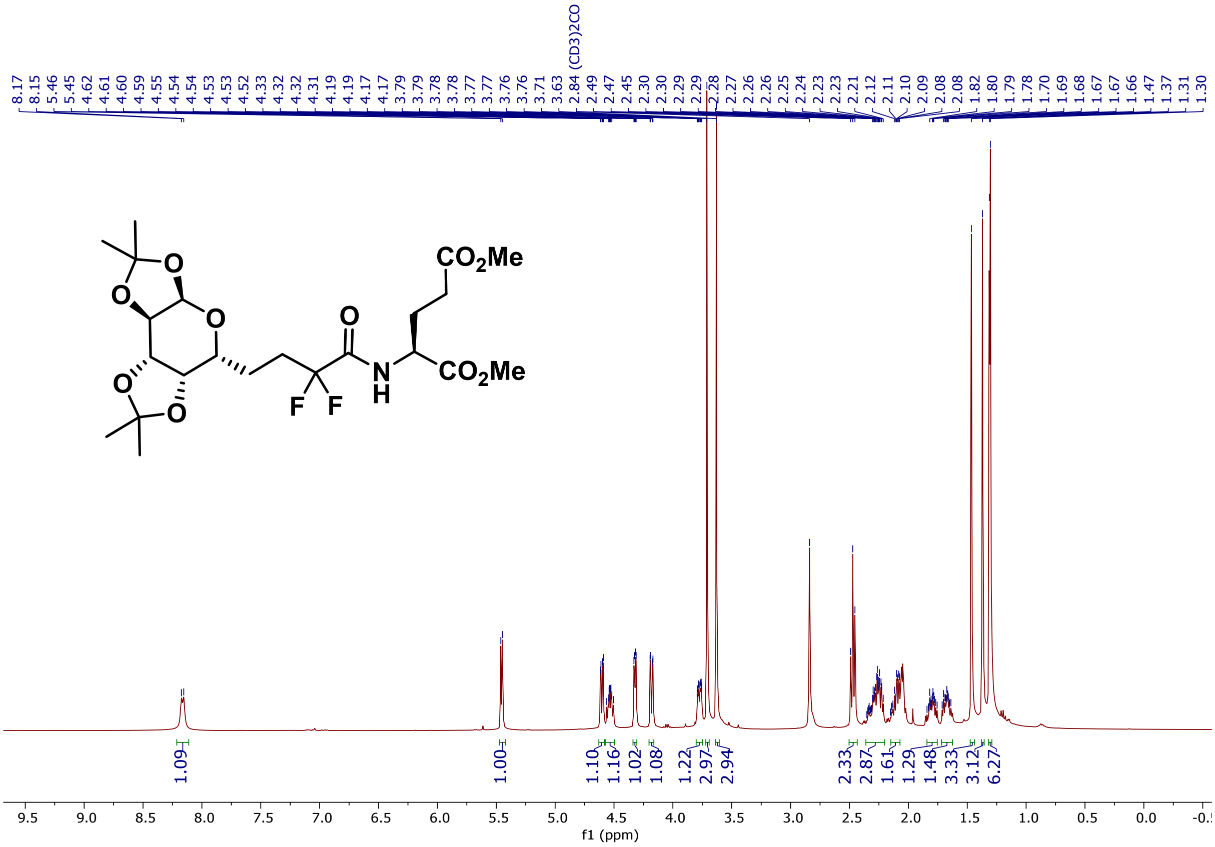

^{1}H NMR (400 MHz, acetone-d_6) of compound 3c

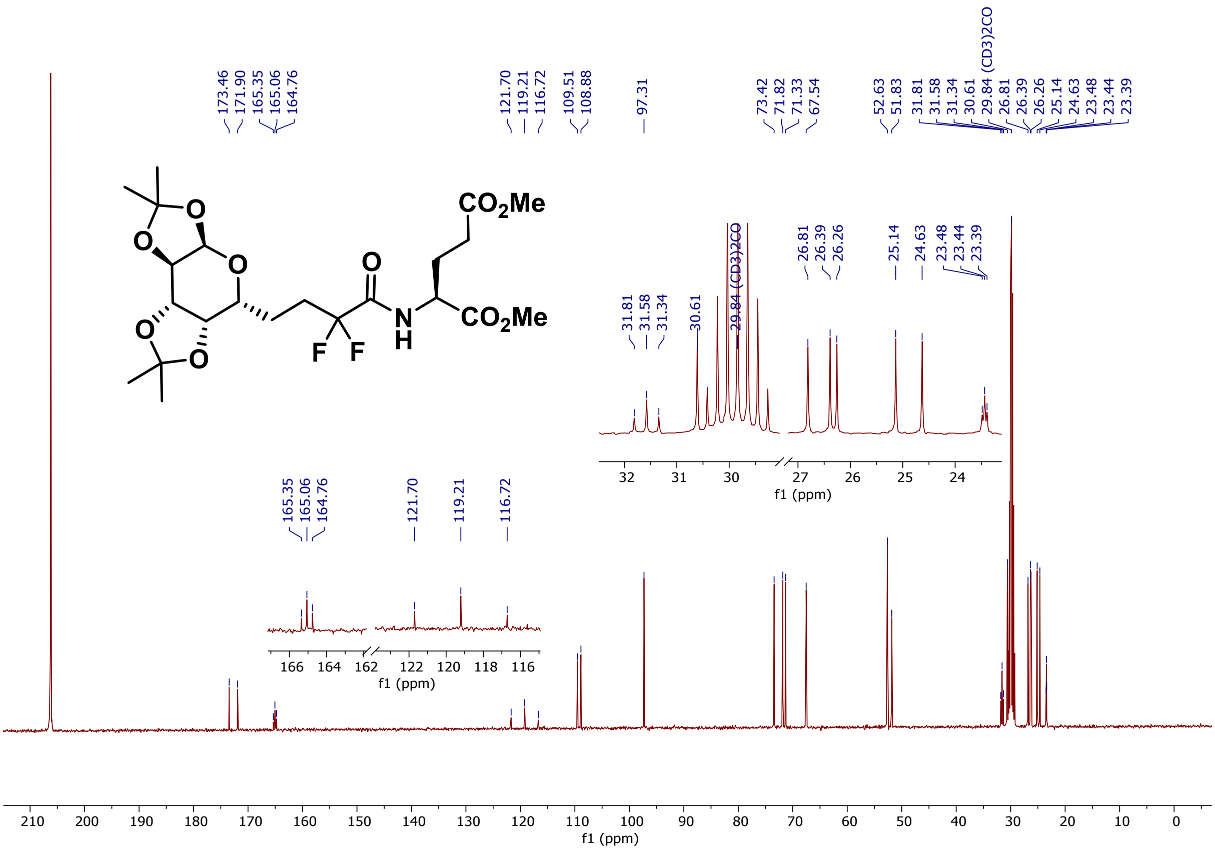

^{13}C NMR (101 MHz, acetone-d_6) of compound 3c

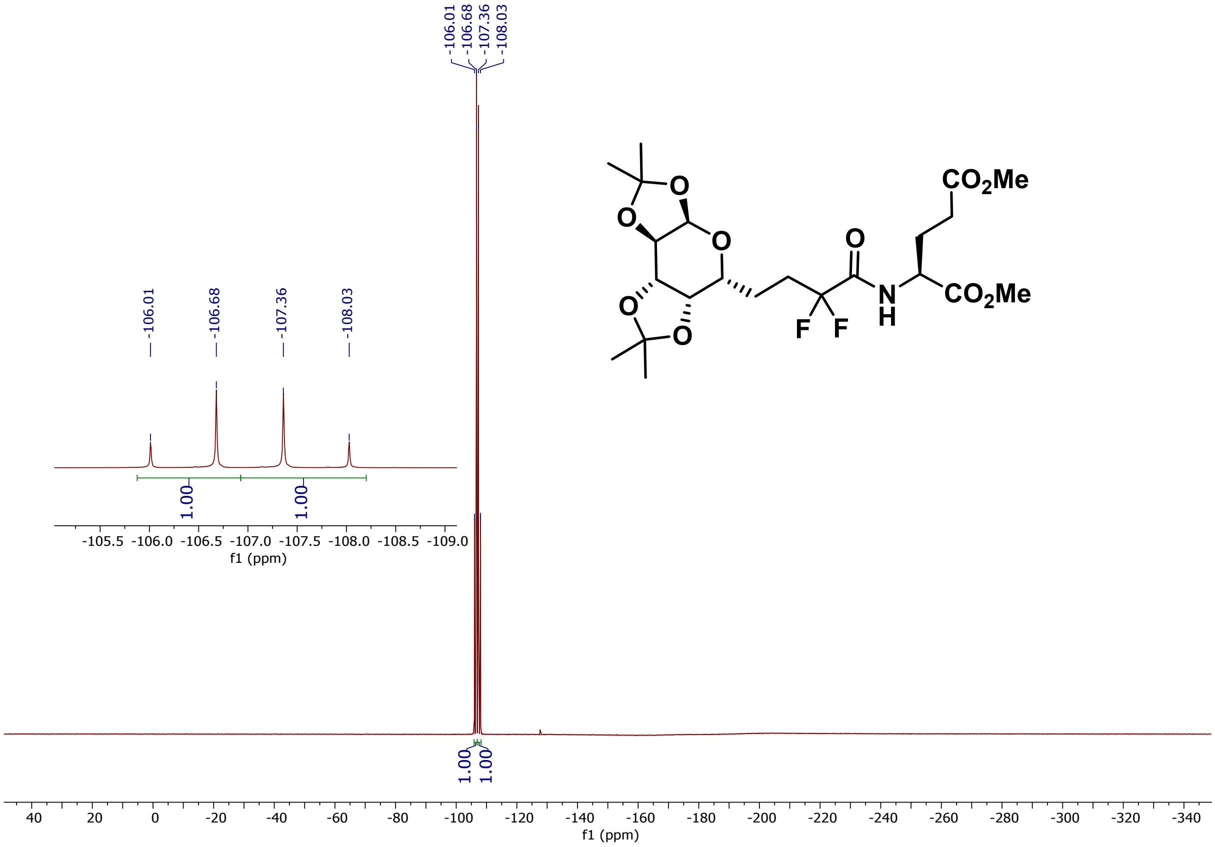

^{19}F NMR (376 MHz, acetone-d$_6$) of compound 3c

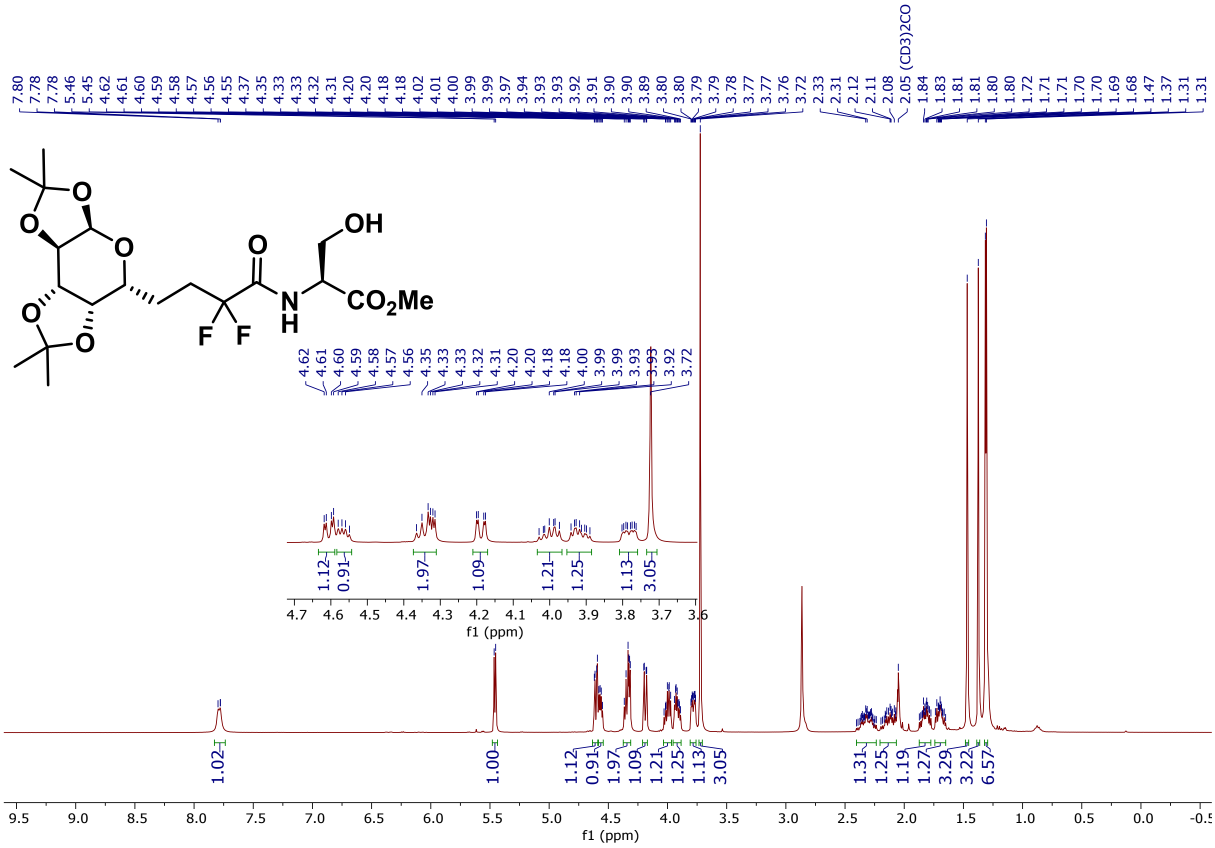

^{1}H NMR (400 MHz, acetone-d$_6$) of compound 3d

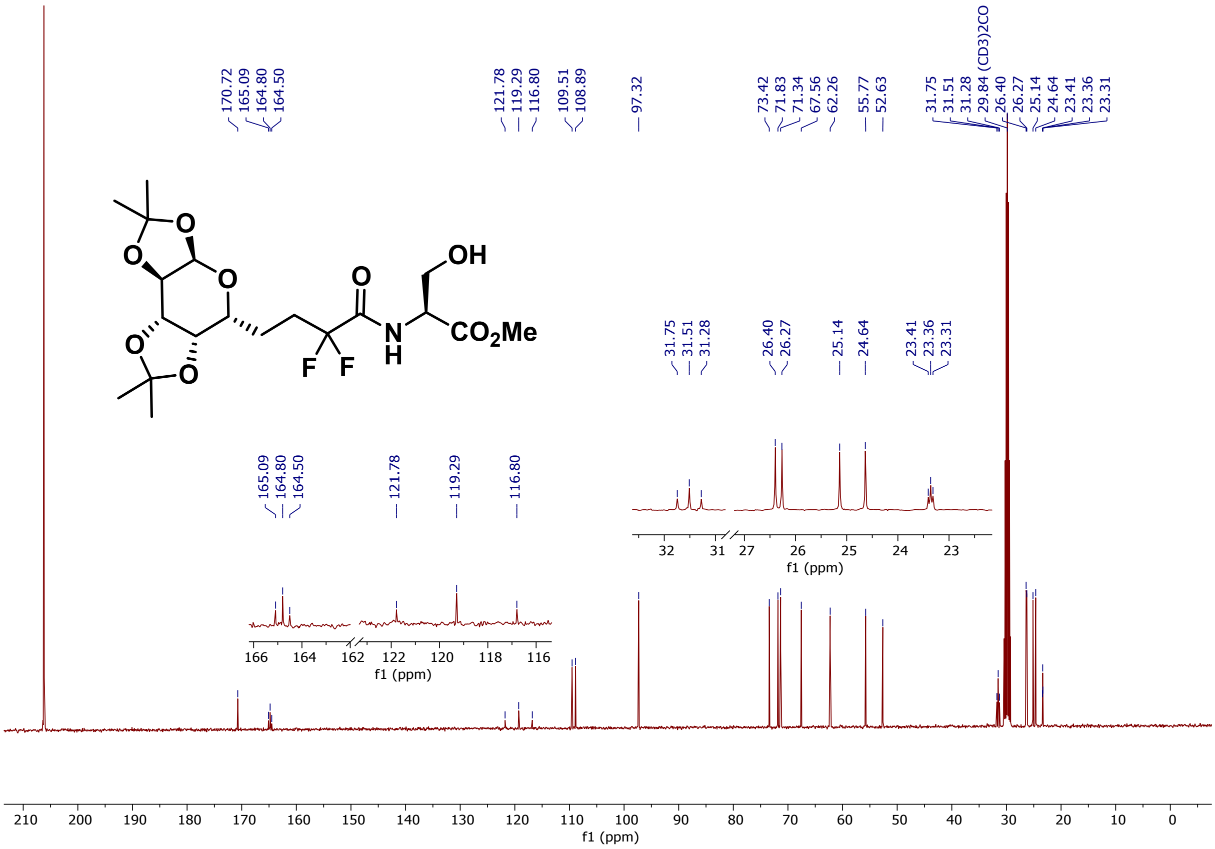

^{13}C NMR (101 MHz, acetone-d$_6$) of compound 3d

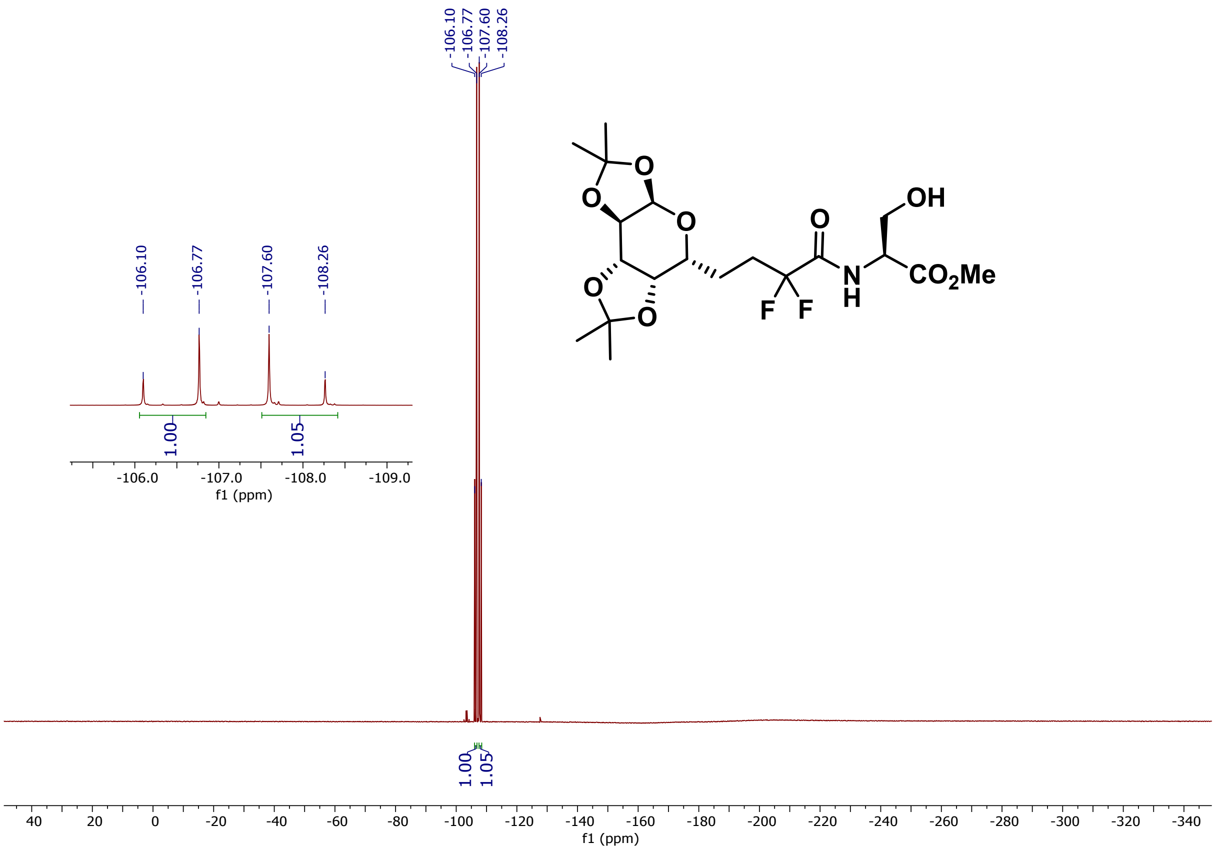

^{19}F NMR (376 MHz, acetone-d$_6$) of compound 3d

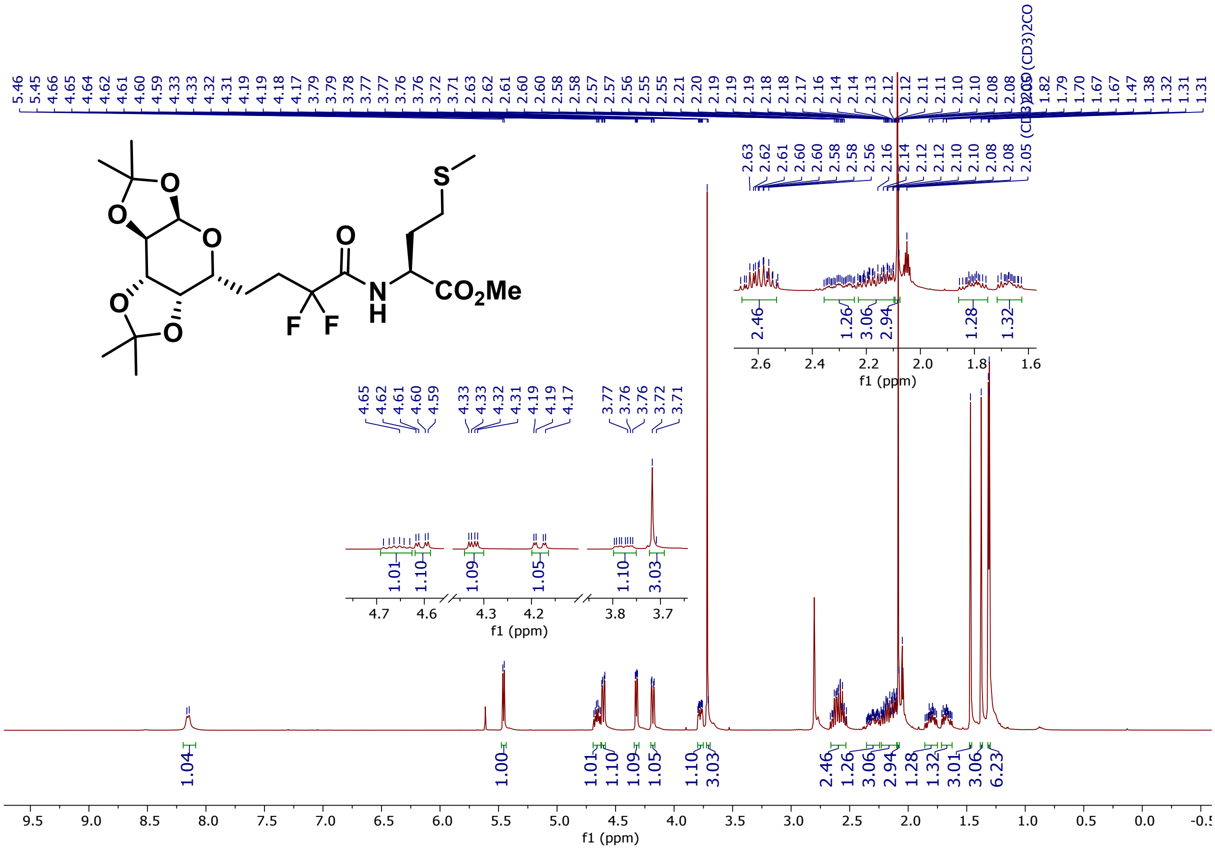

^{1}H NMR (400 MHz, acetone-d$_6$) of compound 3e

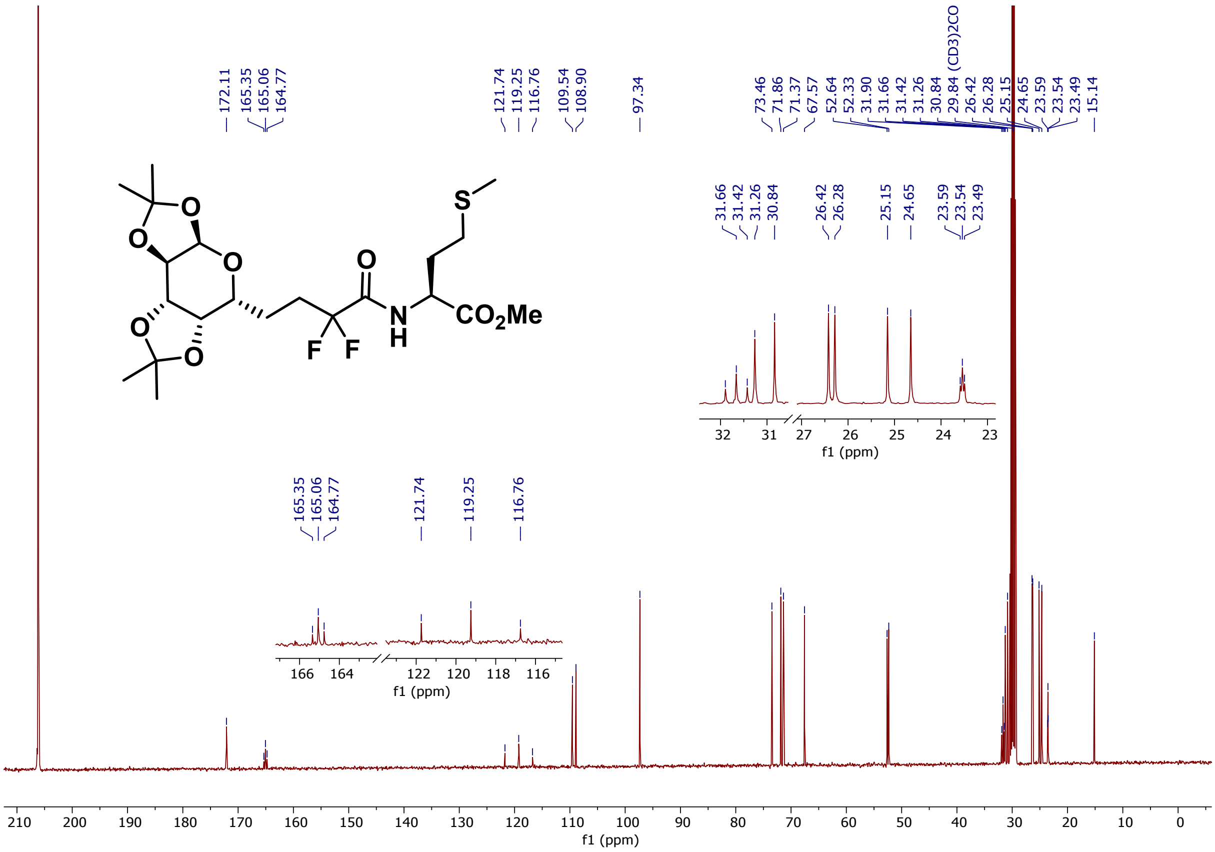

^{13}C NMR (101 MHz, acetone-d$_6$) of compound 3e

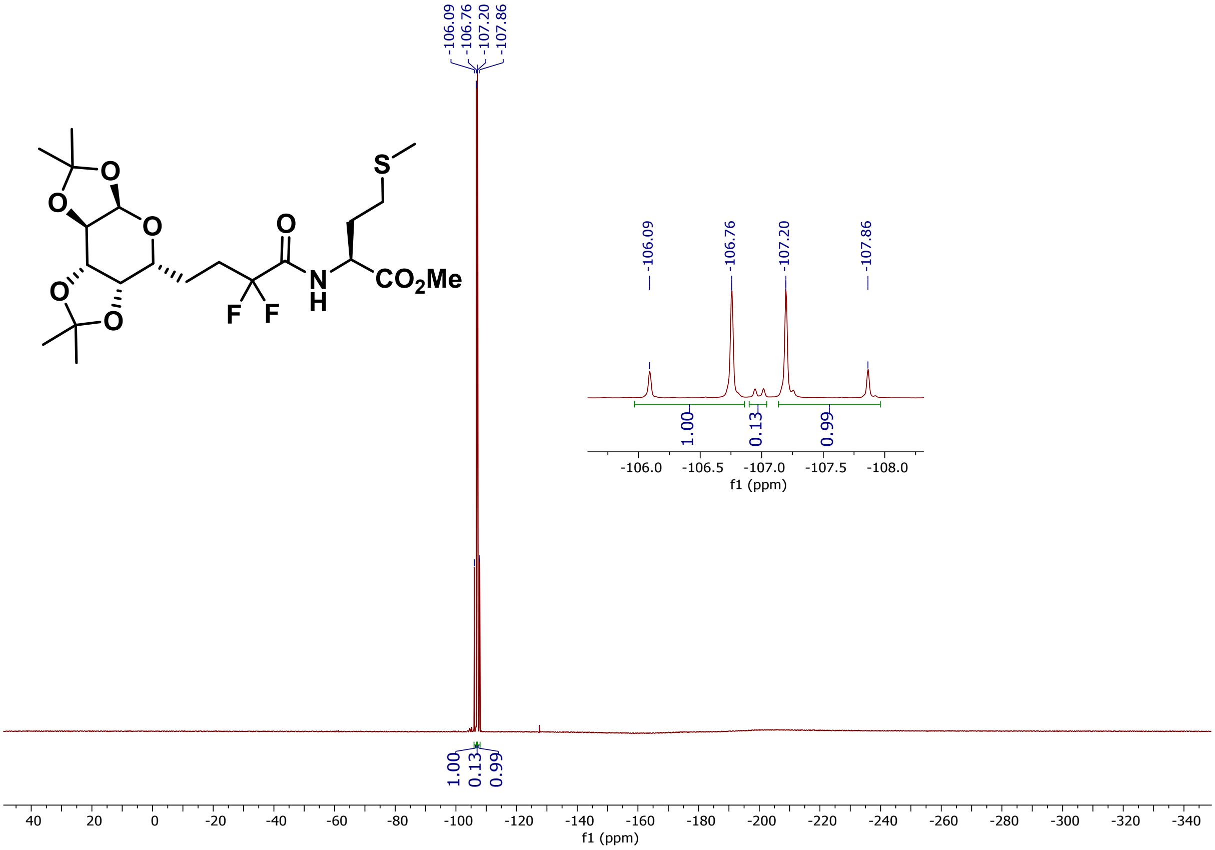

^{19}F NMR (376 MHz, acetone-d$_6$) of compound 3e

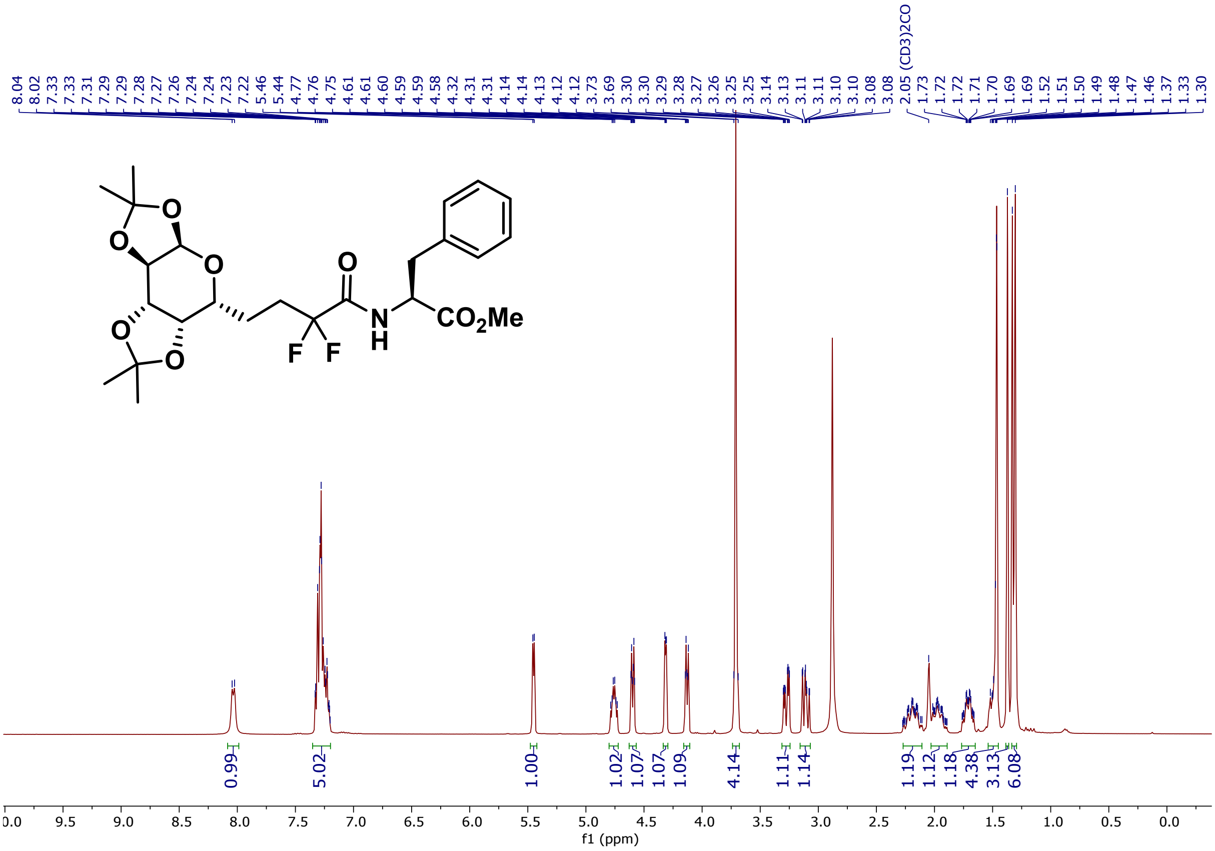

^{1}H NMR (400 MHz, acetone-d$_6$) of compound 3f

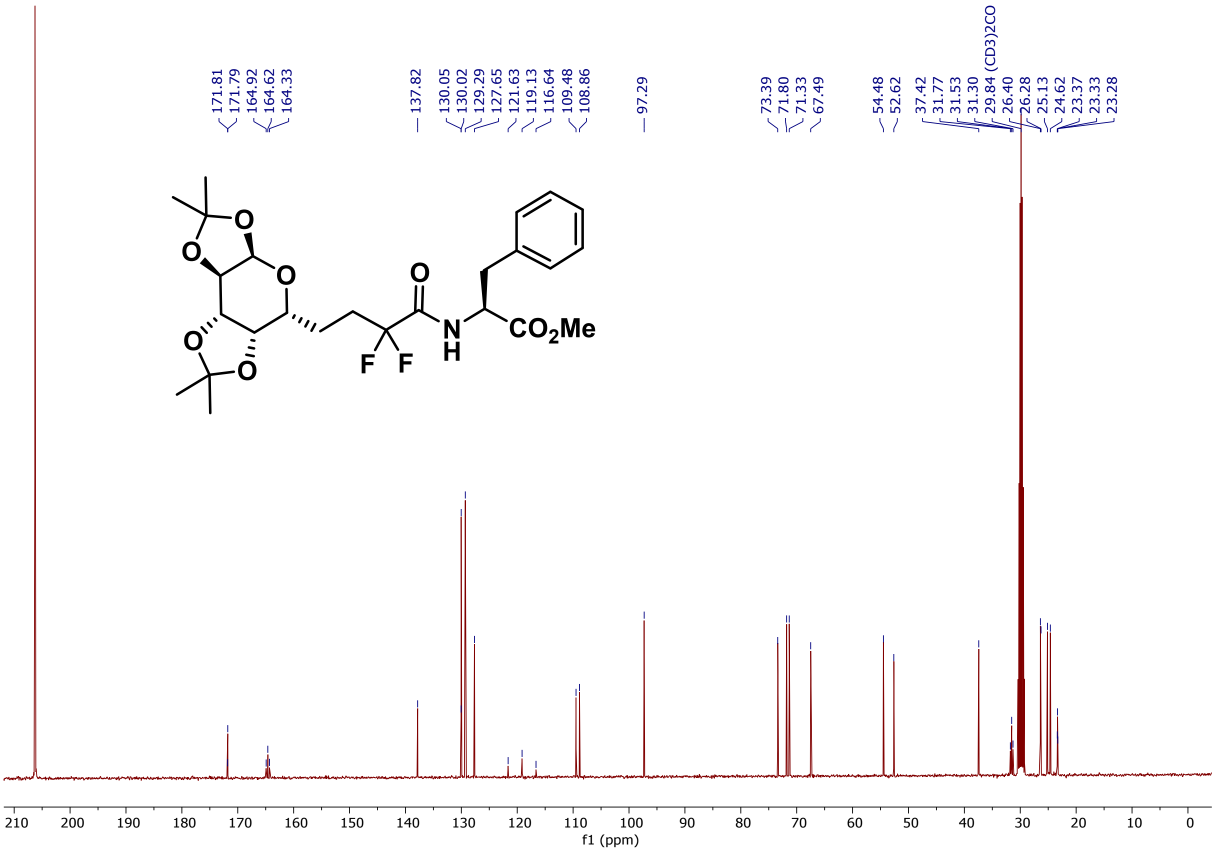

^{13}C NMR (101 MHz, acetone-d_6) of compound 3f

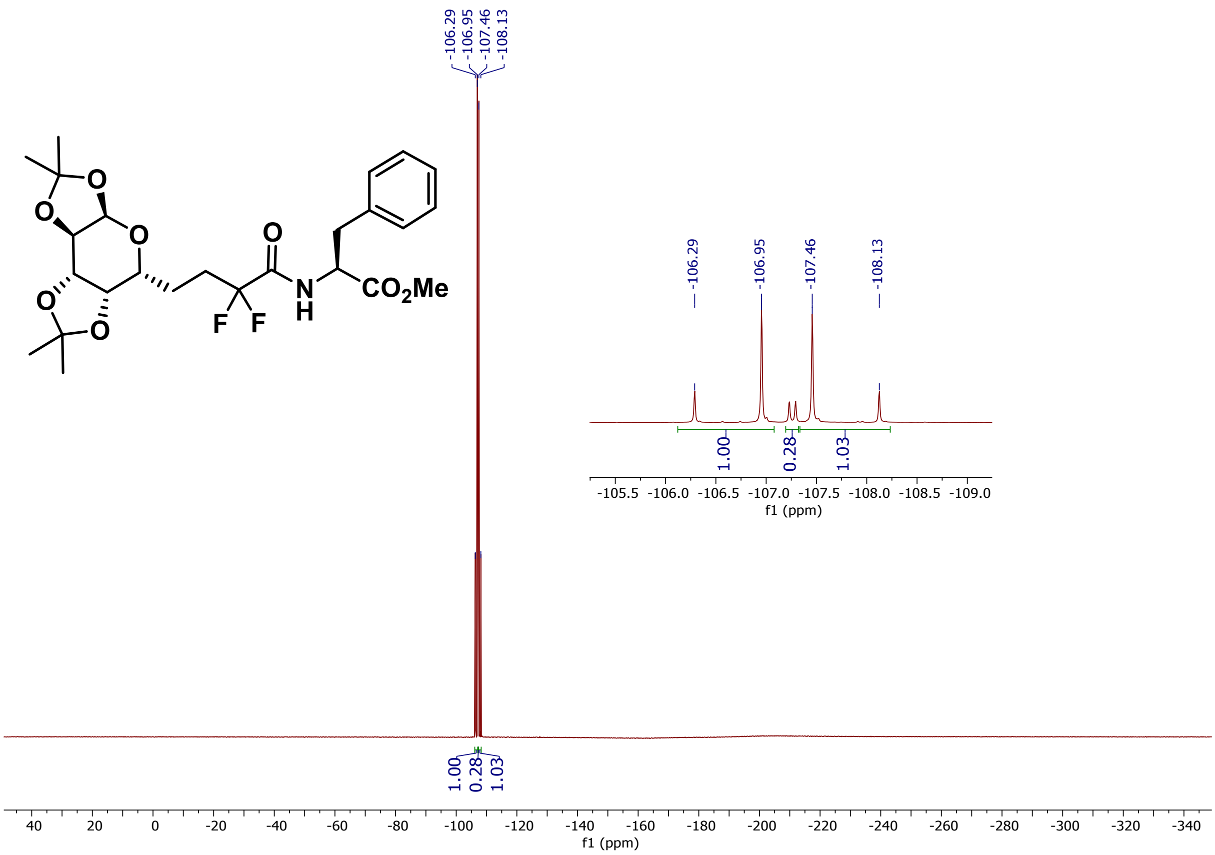

^{19}F NMR (376 MHz, acetone-d_6) of compound 3f

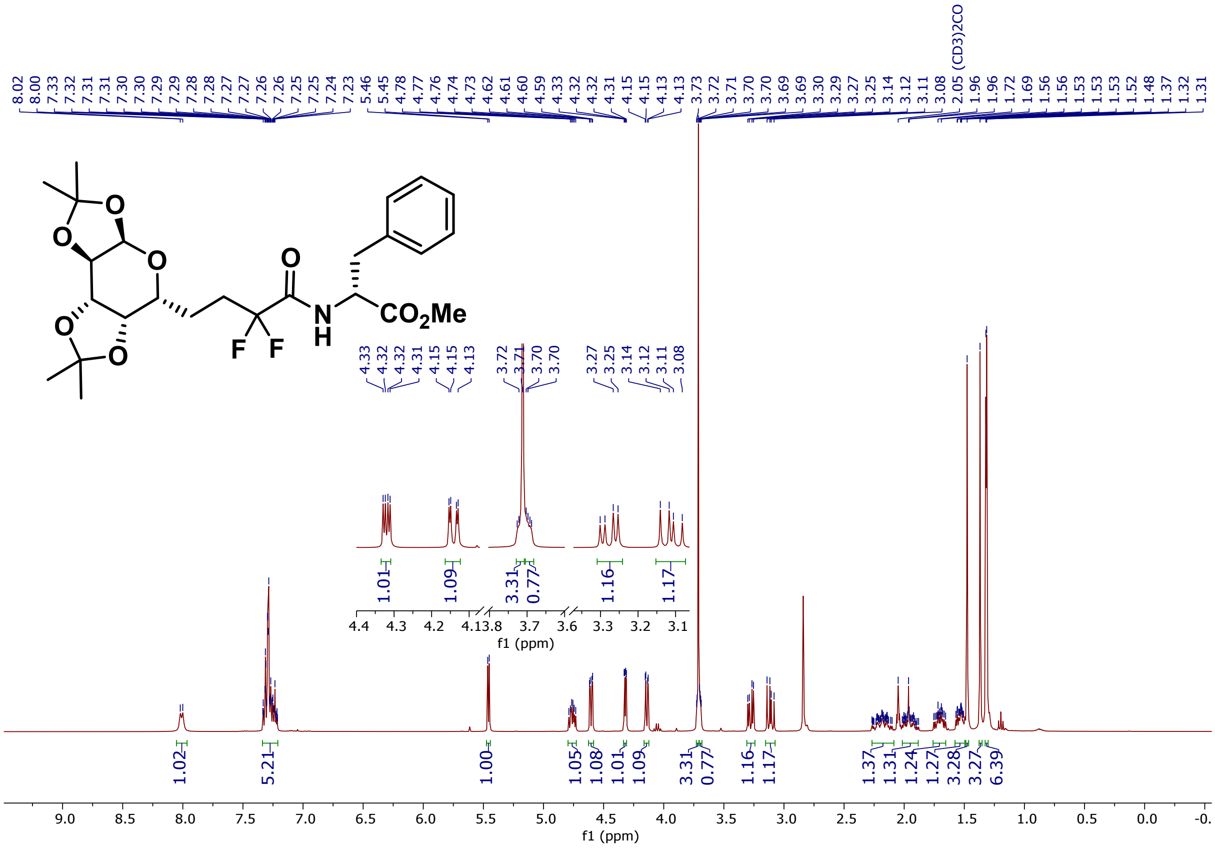

^{1}H NMR (400 MHz, acetone-d_6) of compound 3g

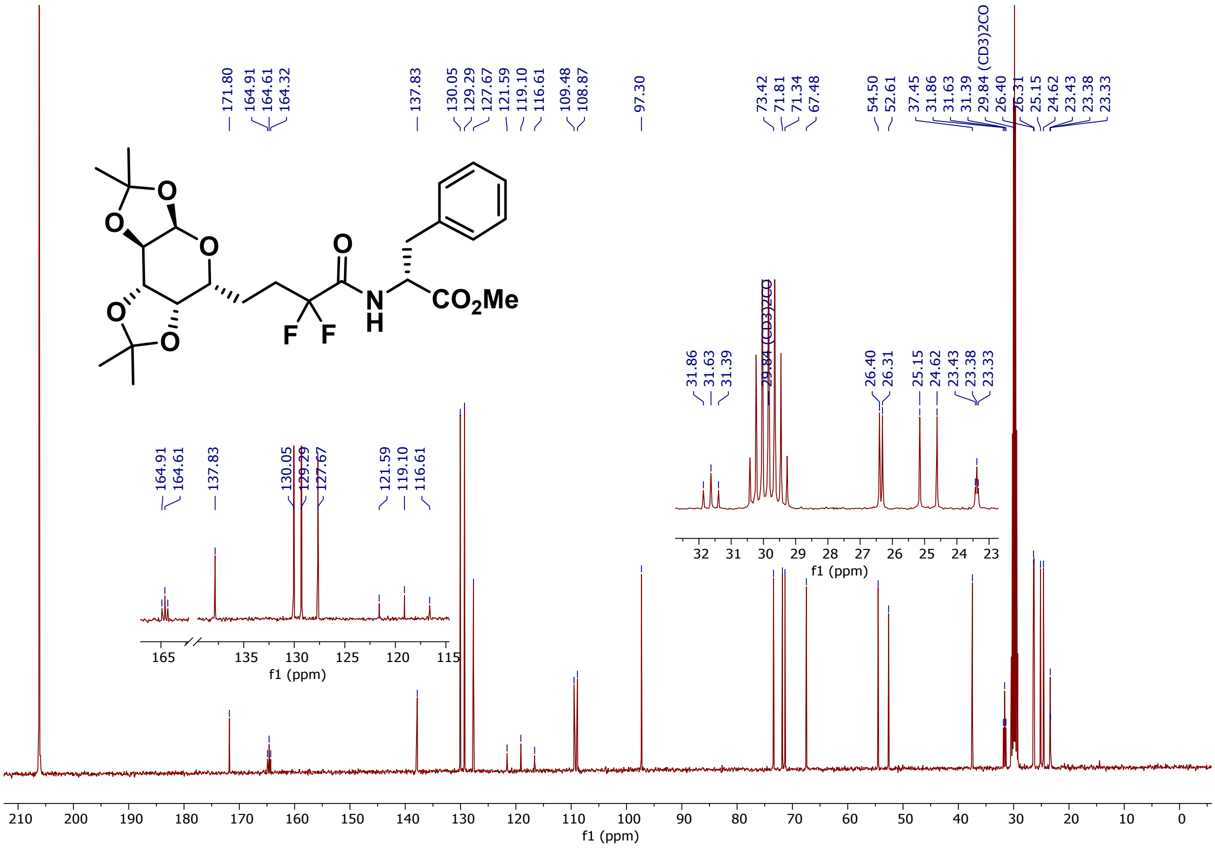

^{13}C NMR (101 MHz, acetone-d_6) of compound 3g

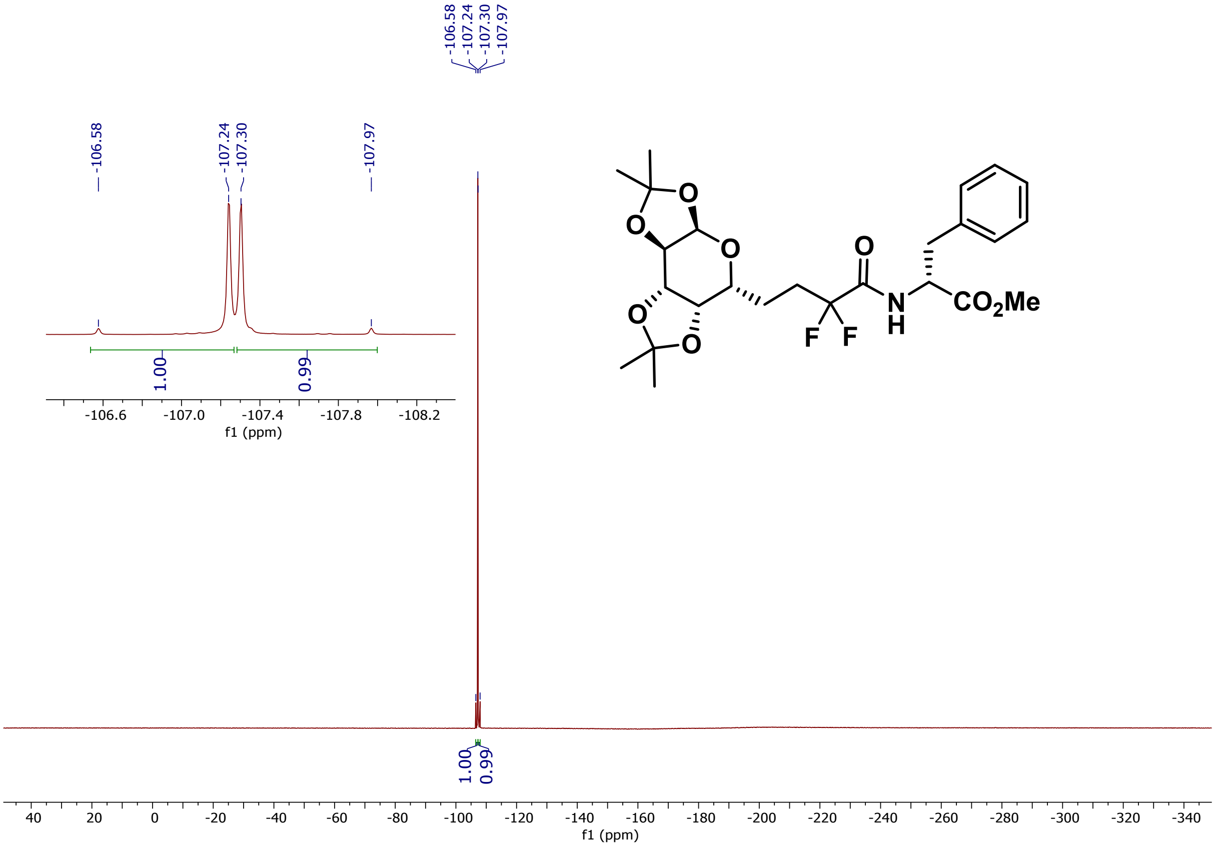

^{19}F NMR (376 MHz, acetone-d$_6$) of compound 3g

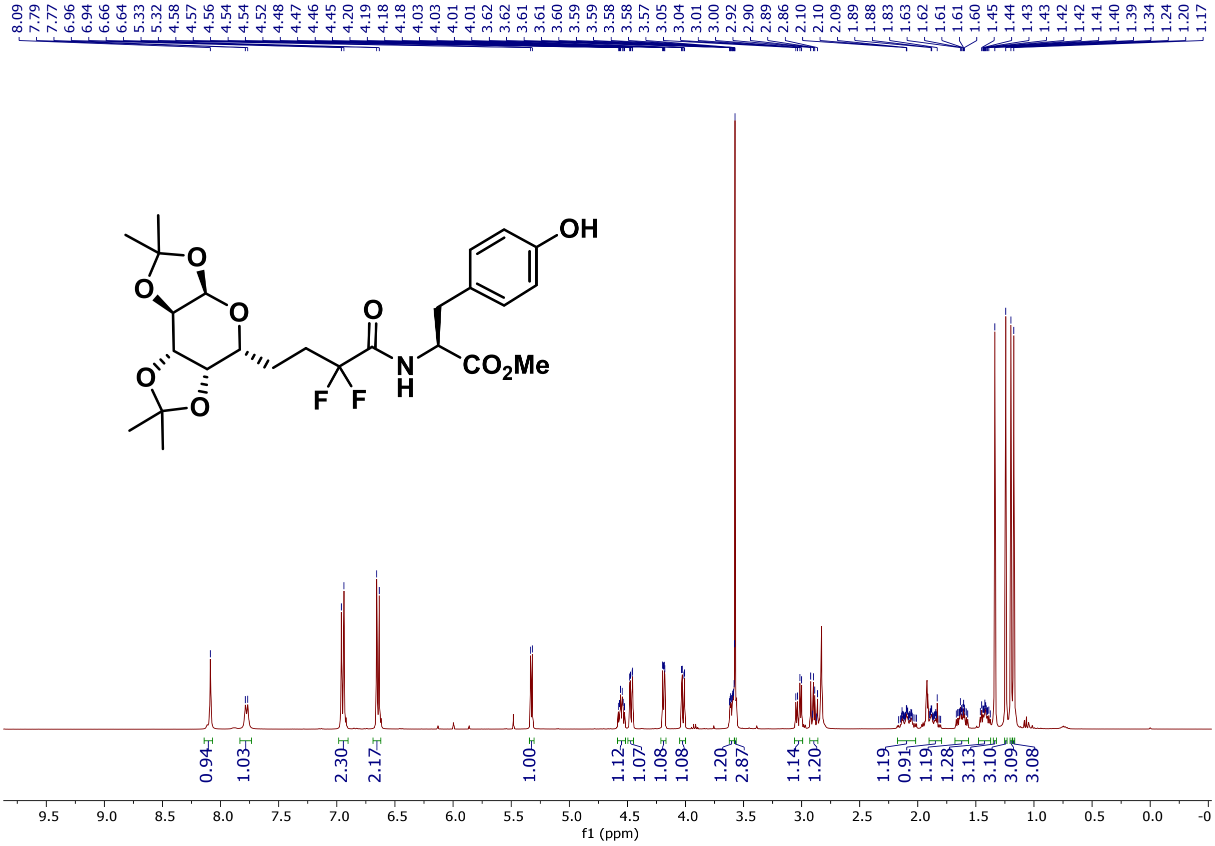

^{1}H NMR (400 MHz, acetone-d$_6$) of compound 3h

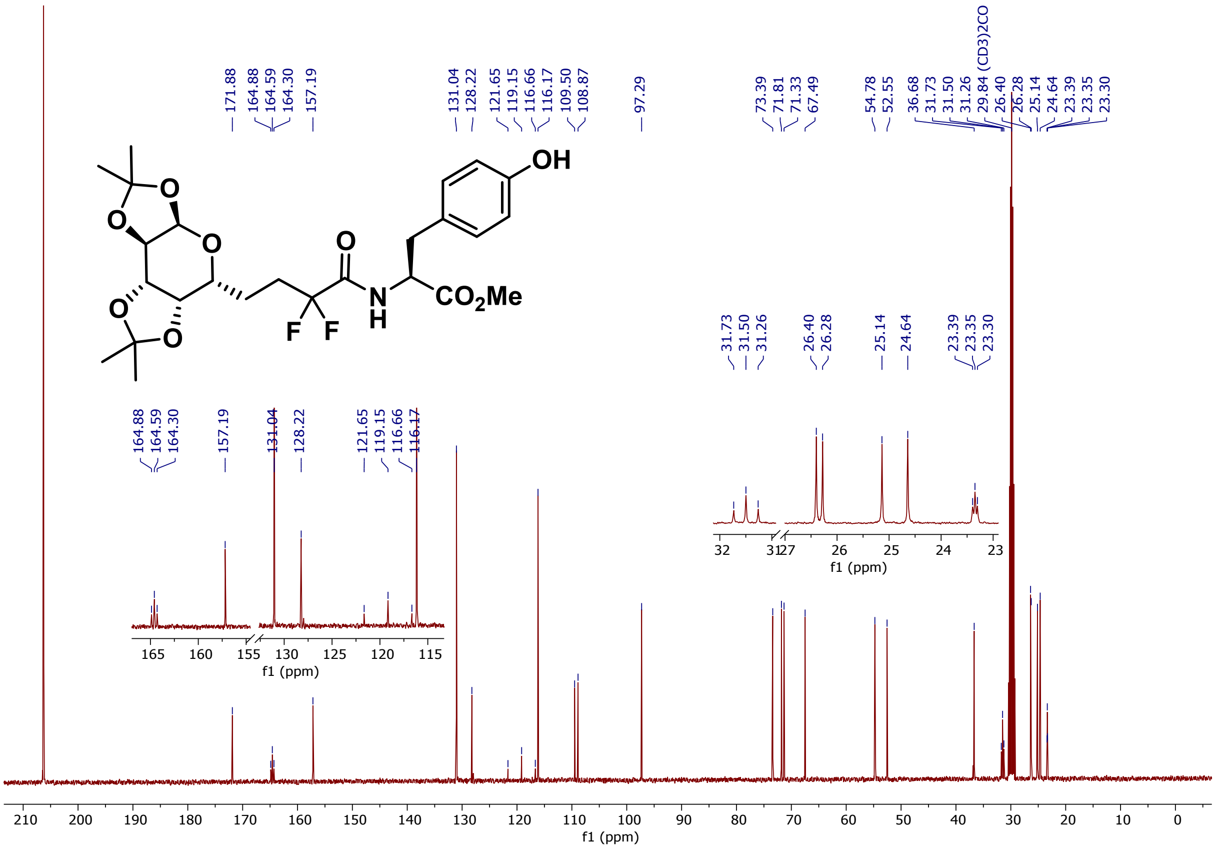

^{13}C NMR (101 MHz, acetone-d$_6$) of compound 3h

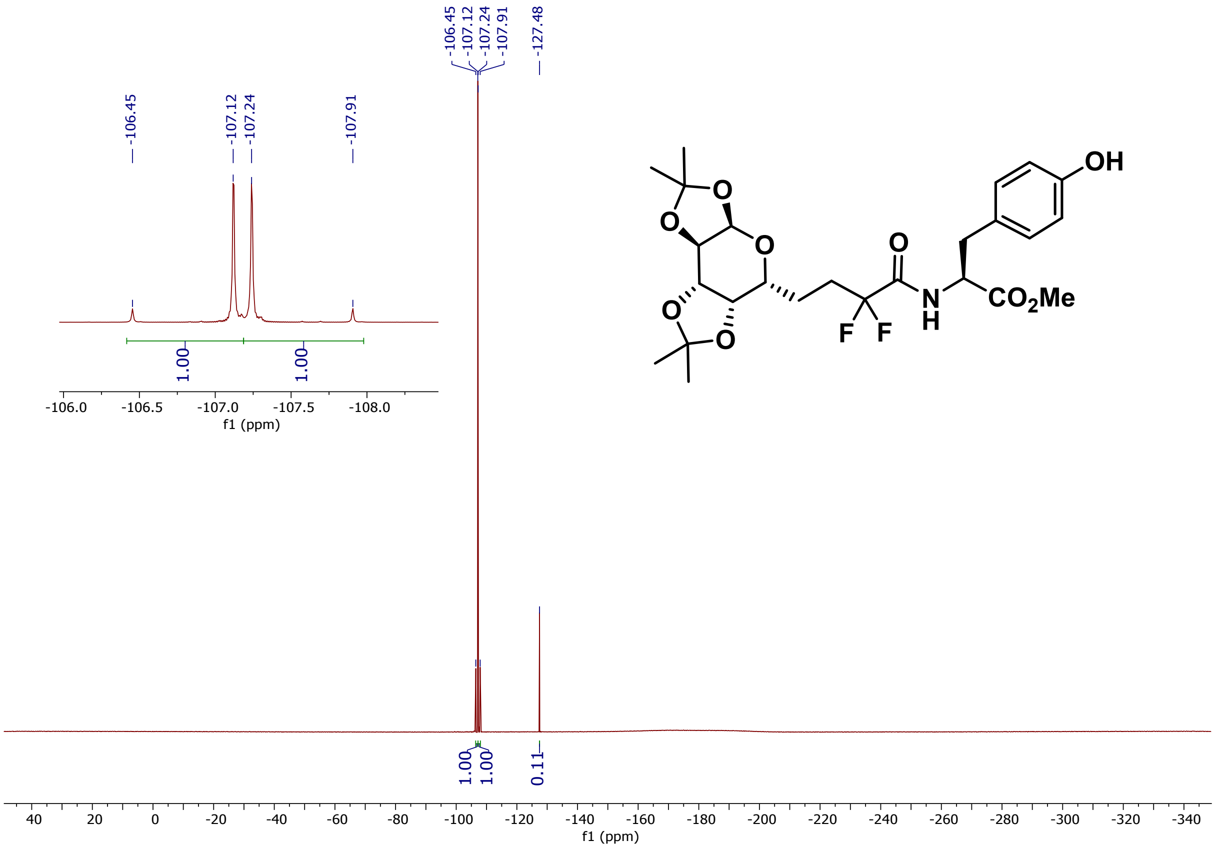

^{19}F NMR (376 MHz, acetone-d$_6$) of compound 3h

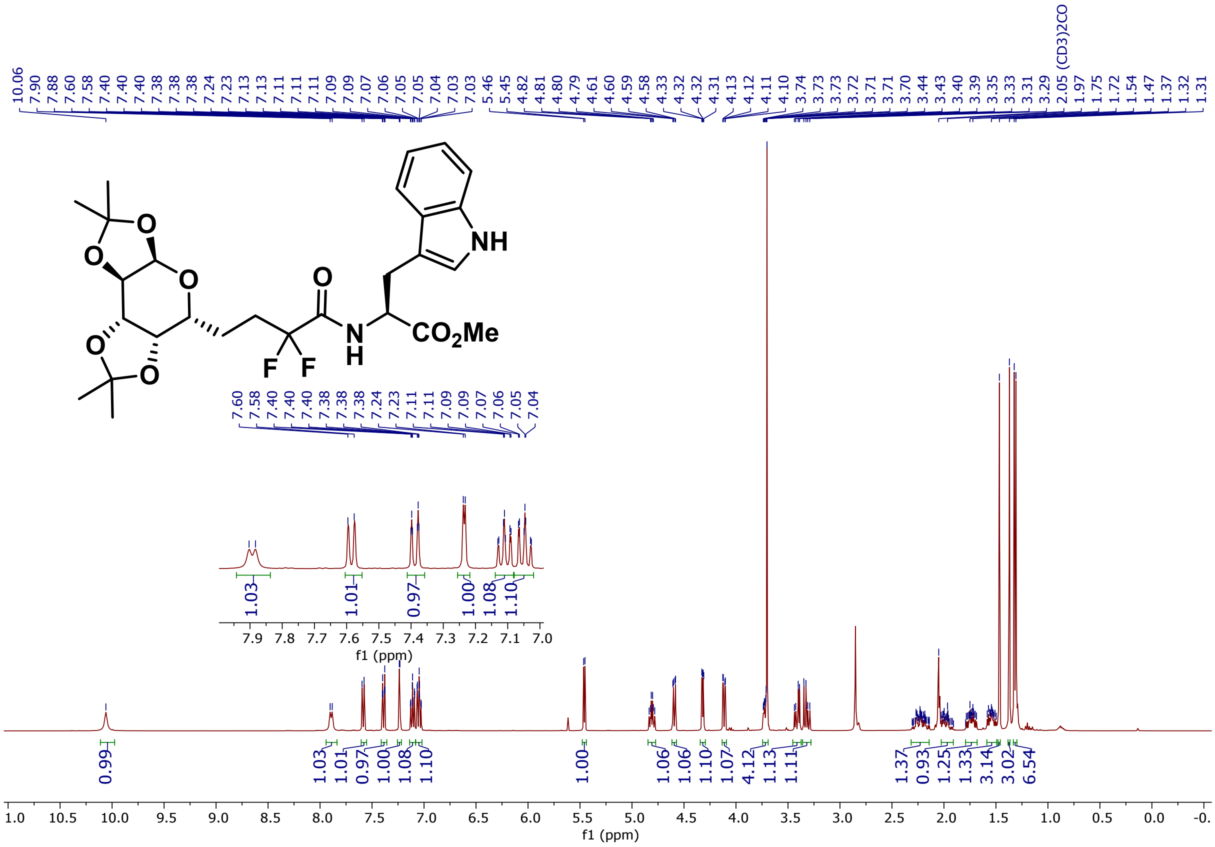

¹H NMR (400 MHz, acetone-d₆) of compound 3i

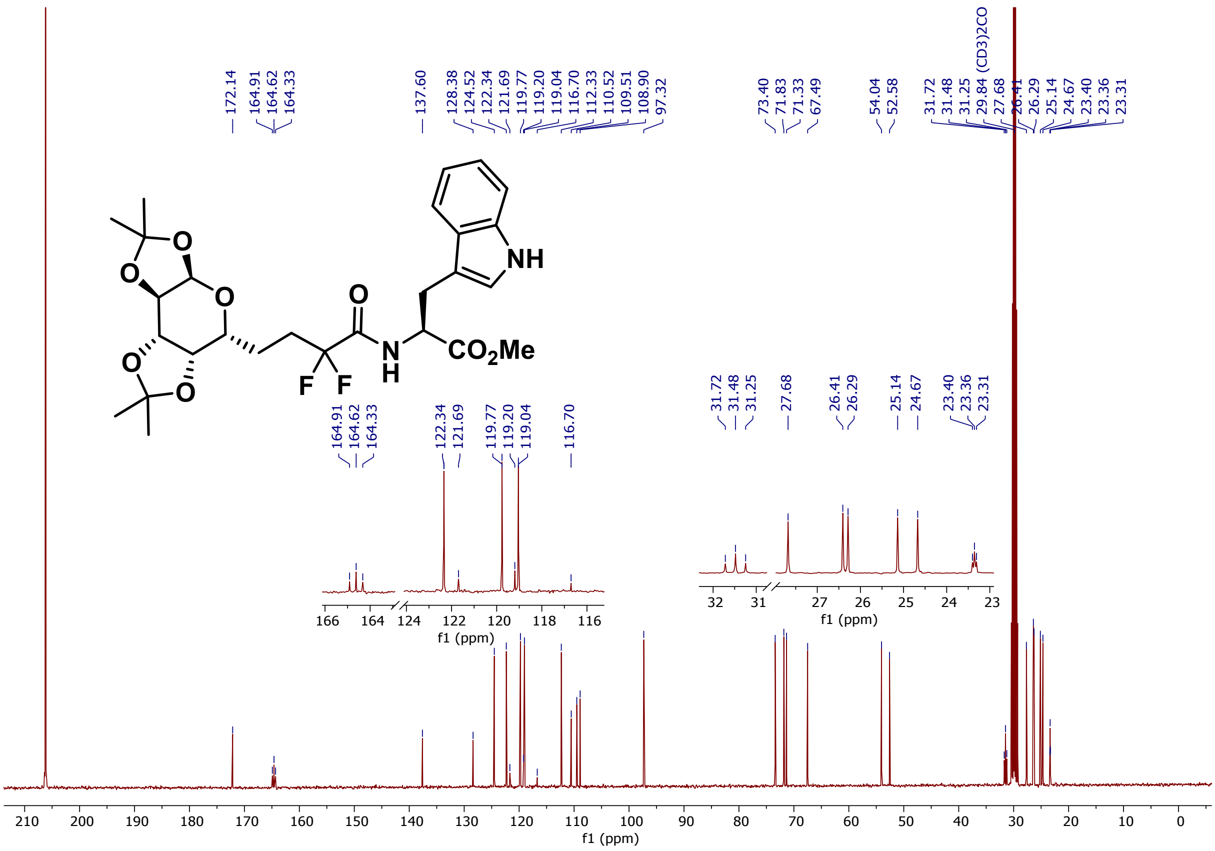

¹³C NMR (101 MHz, acetone-d₆) of compound 3i

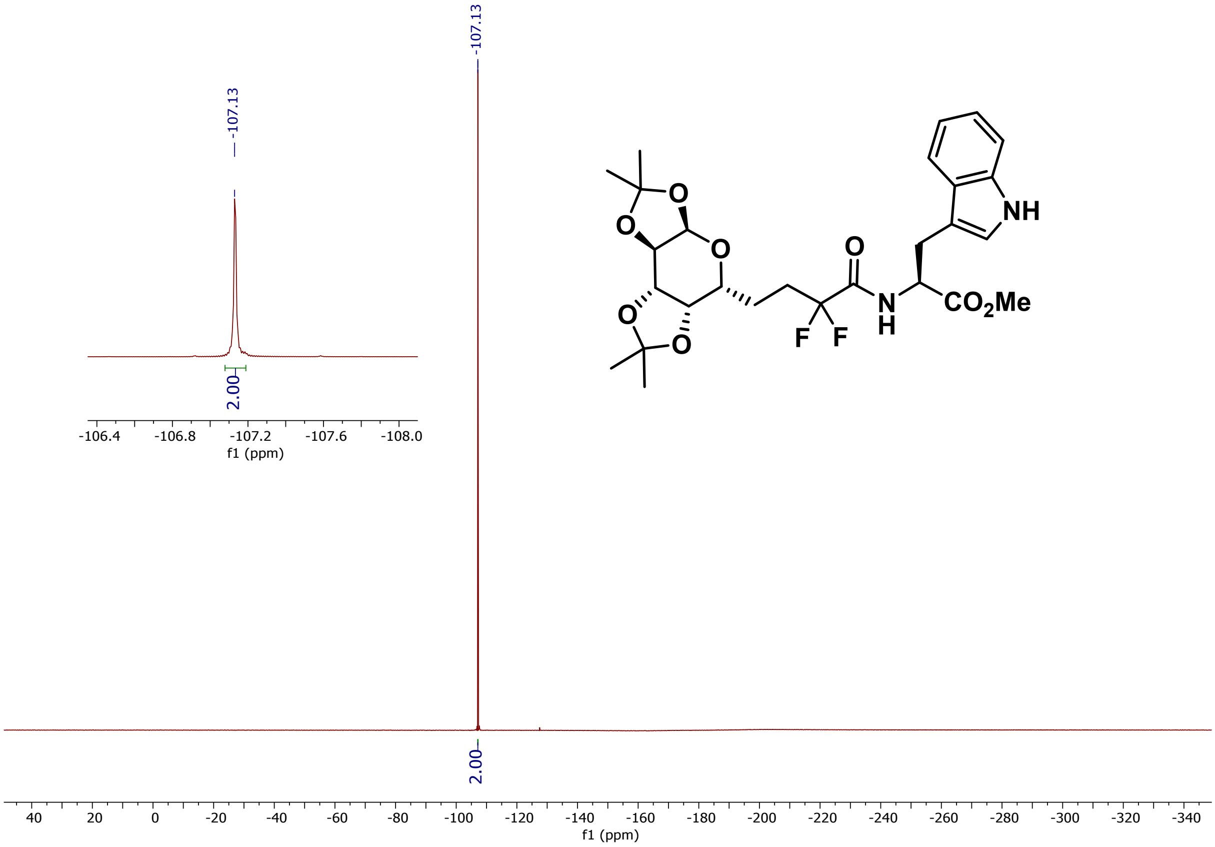

^{19}F NMR (376 MHz, acetone-d$_6$) of compound 3i

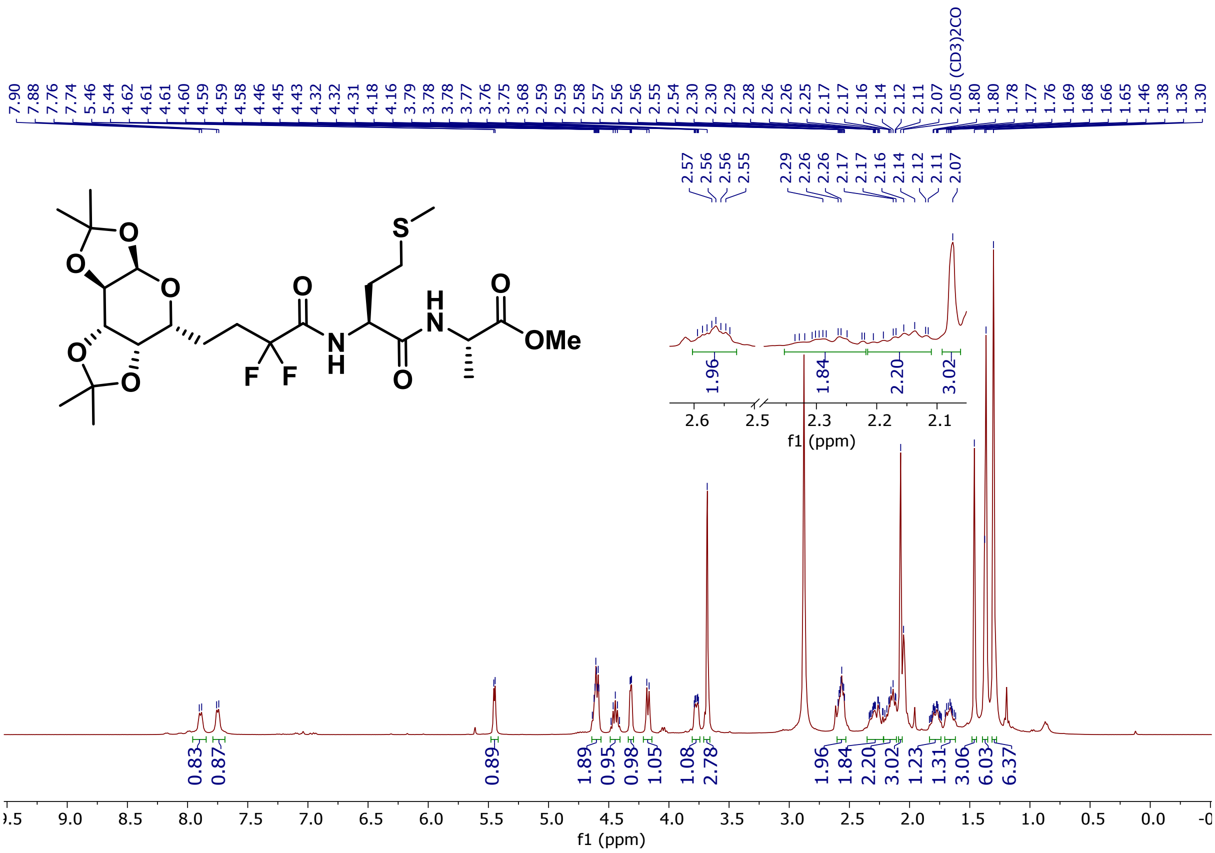

^{1}H NMR (400 MHz, acetone-d$_6$) of compound 3j

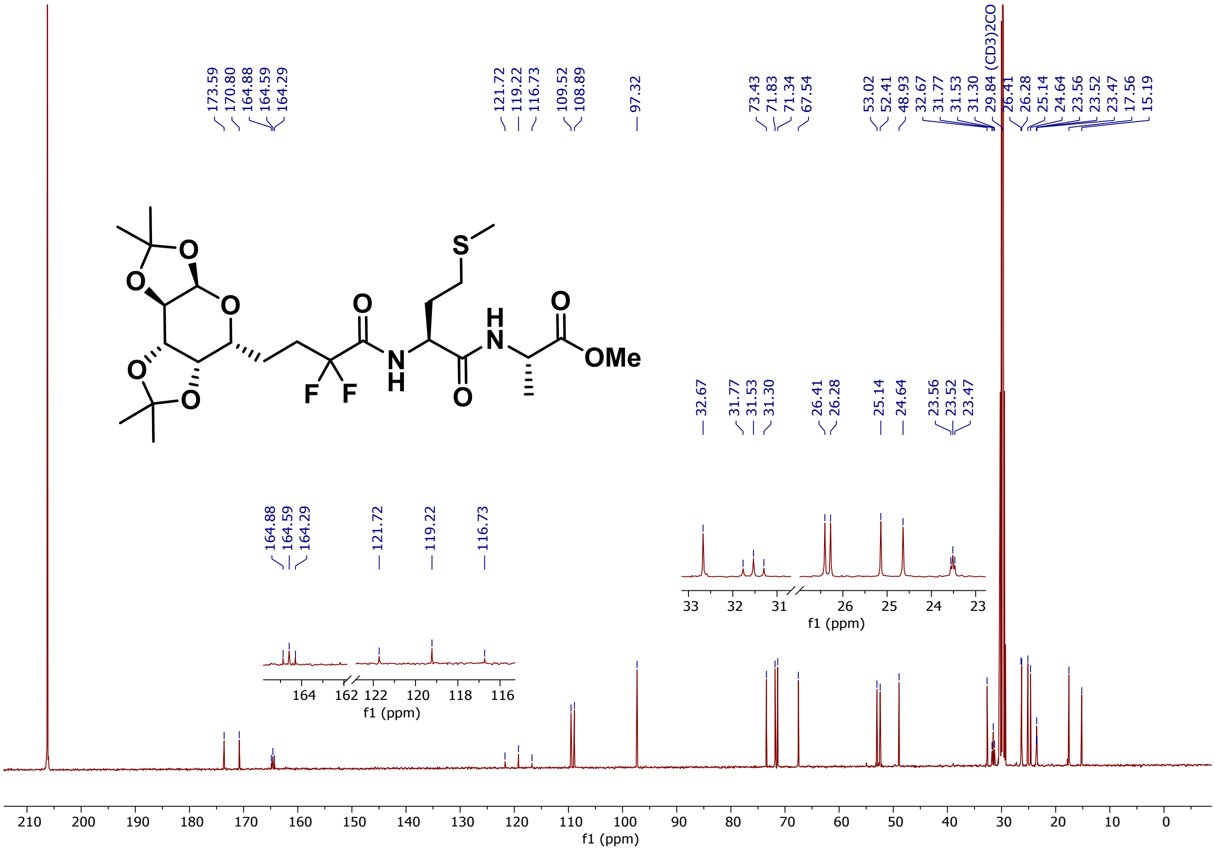

^{13}C NMR (101 MHz, acetone-d_6) of compound 3j

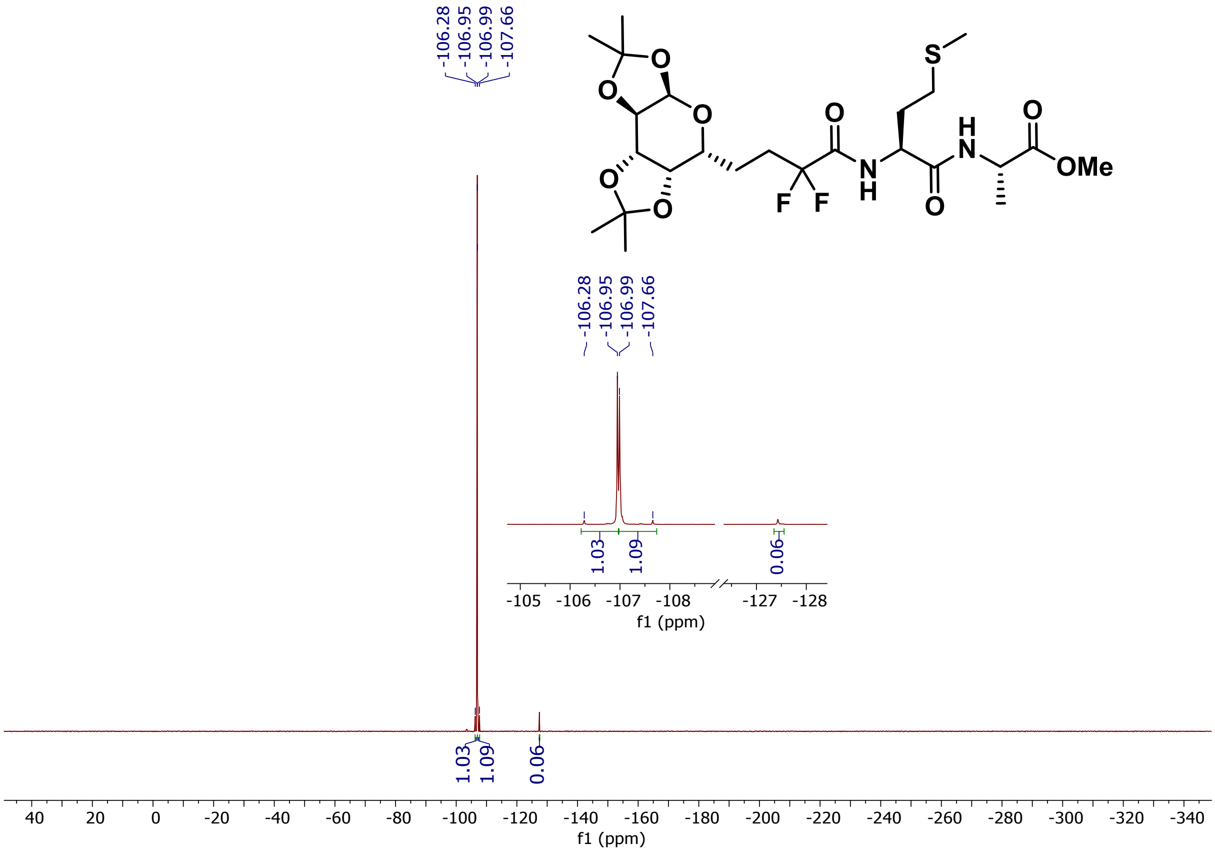

^{19}F NMR (376 MHz, acetone-d_6) of compound 3j

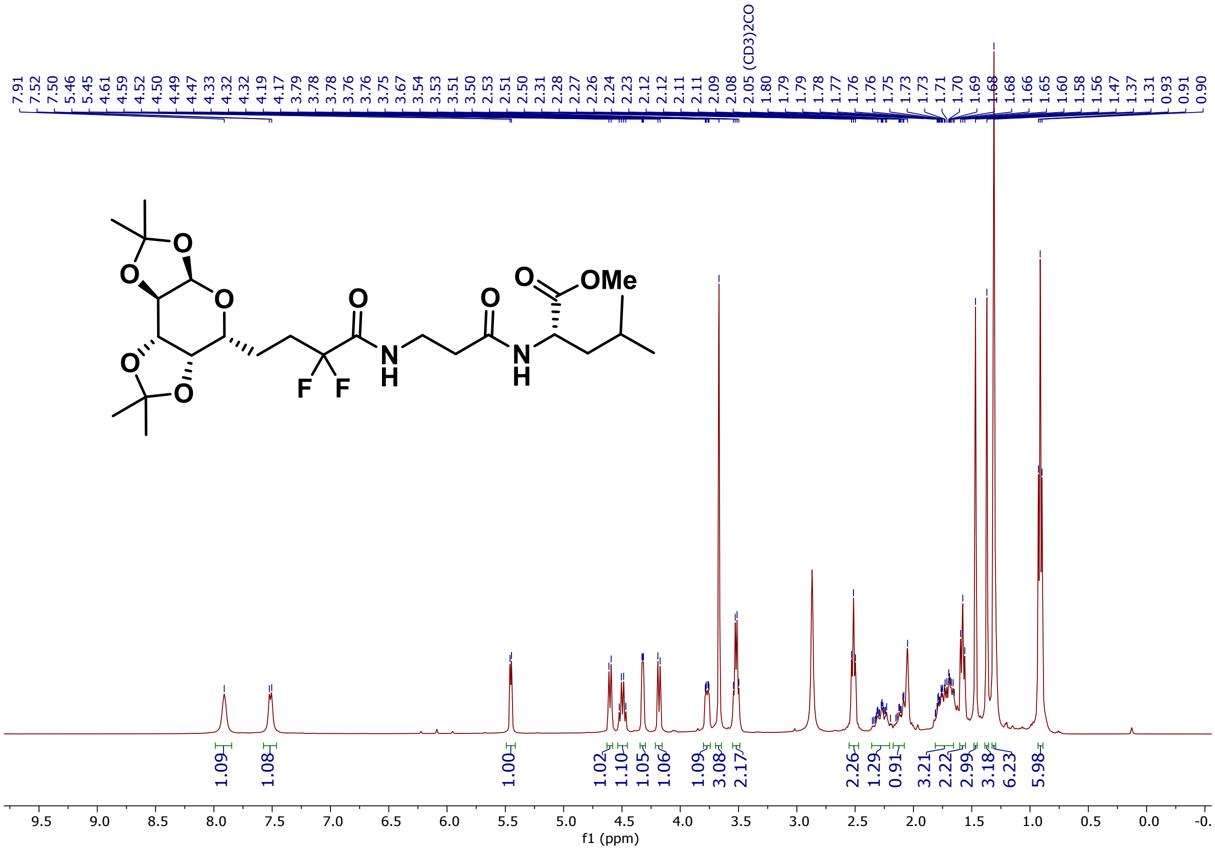

^{1}H NMR (400 MHz, acetone-d$_6$) of compound 3k

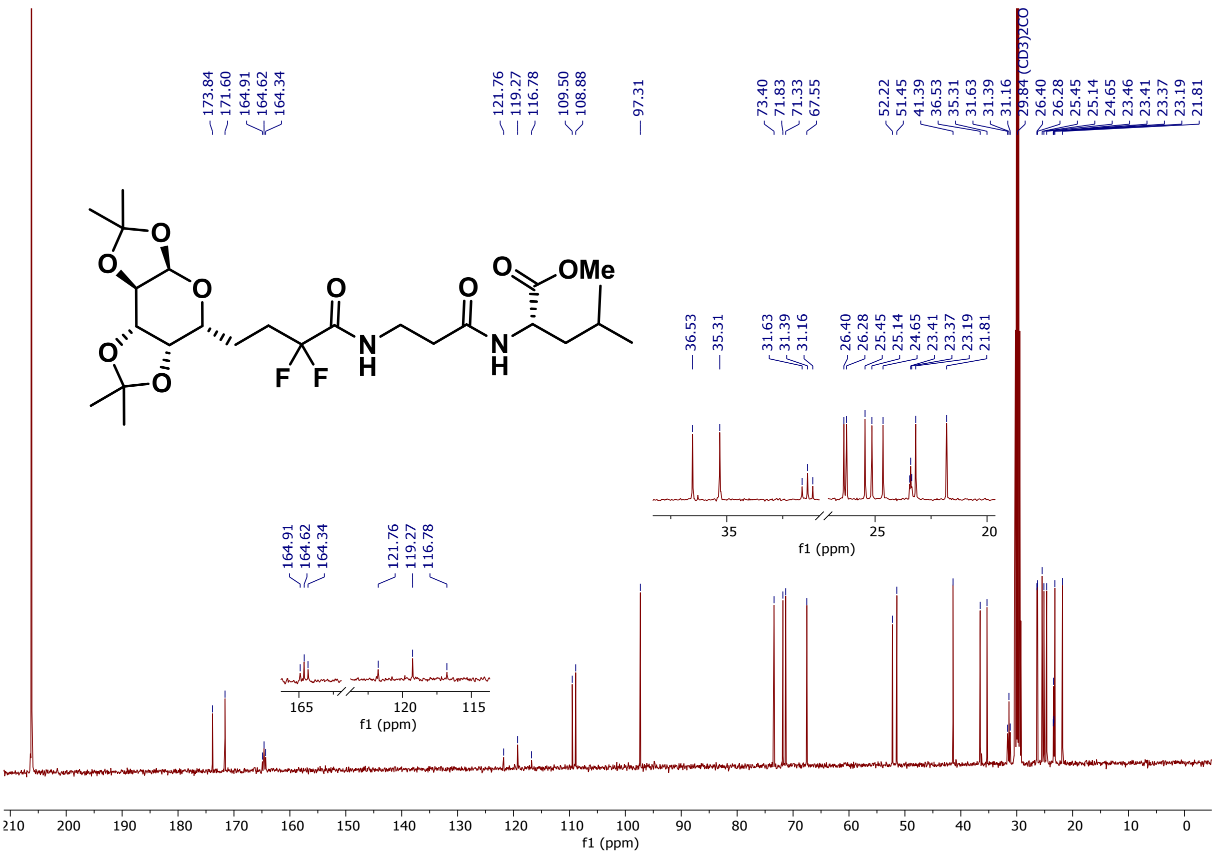

^{13}C NMR (101 MHz, acetone-d$_6$) of compound 3k

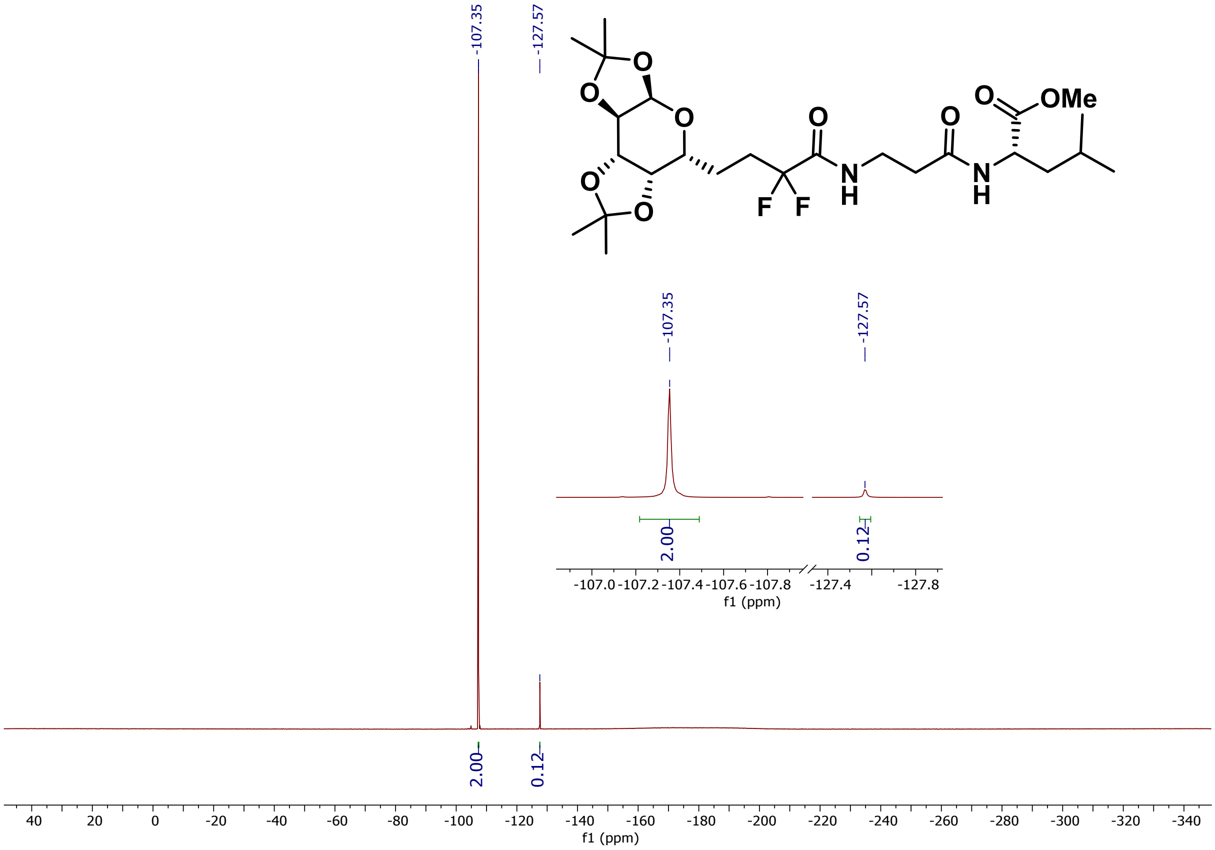

^{19}F NMR (376 MHz, acetone-d$_6$) of compound 3k

15. Characterization Spectra of compounds 4a-4j

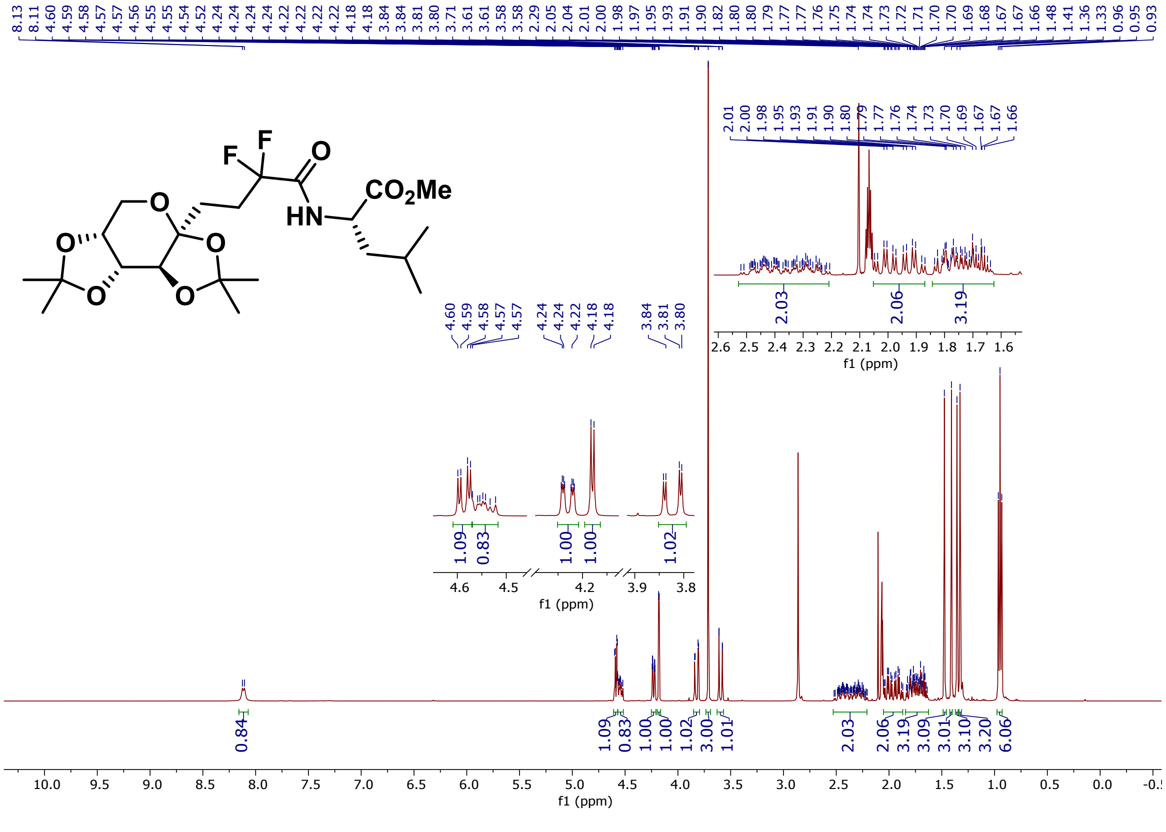

^{1}H NMR (400 MHz, acetone-d_6) of compound 4a

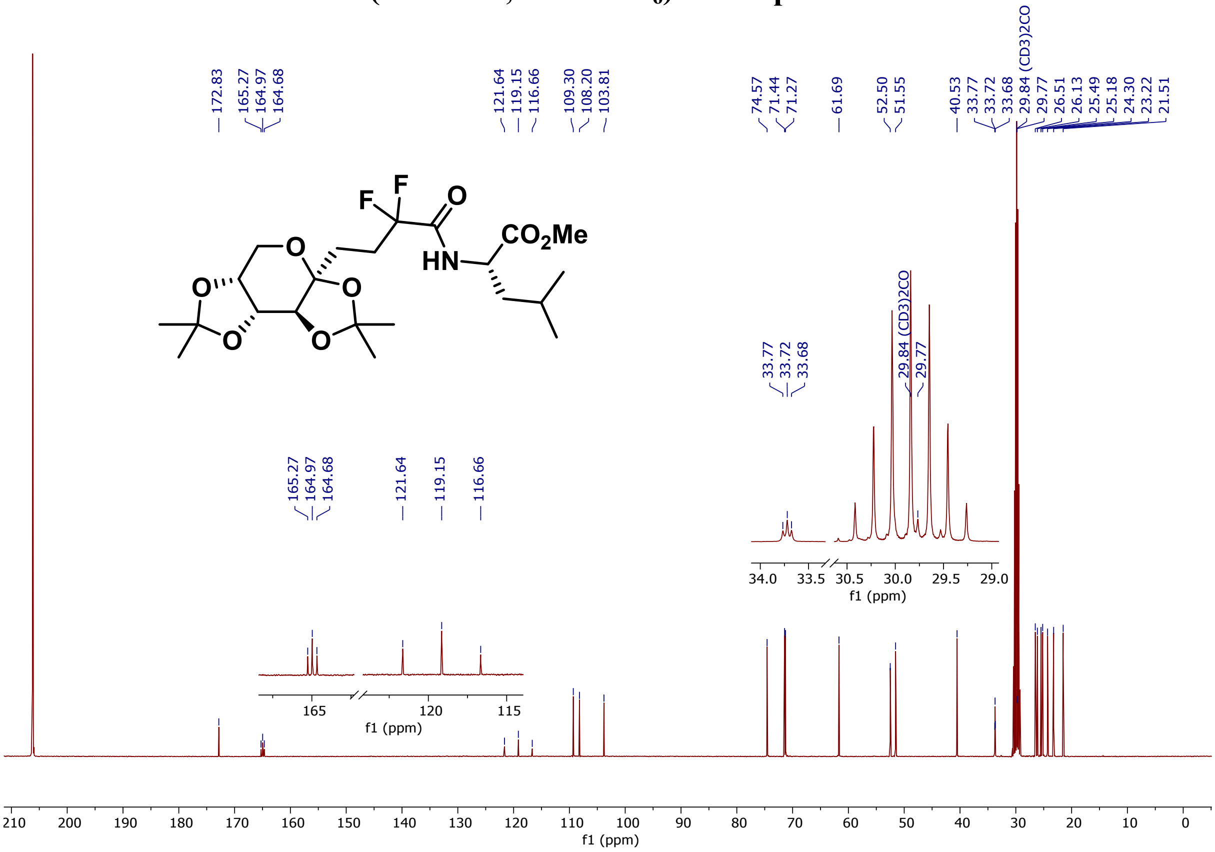

^{13}C NMR (101 MHz, acetone-d_6) of compound 4a

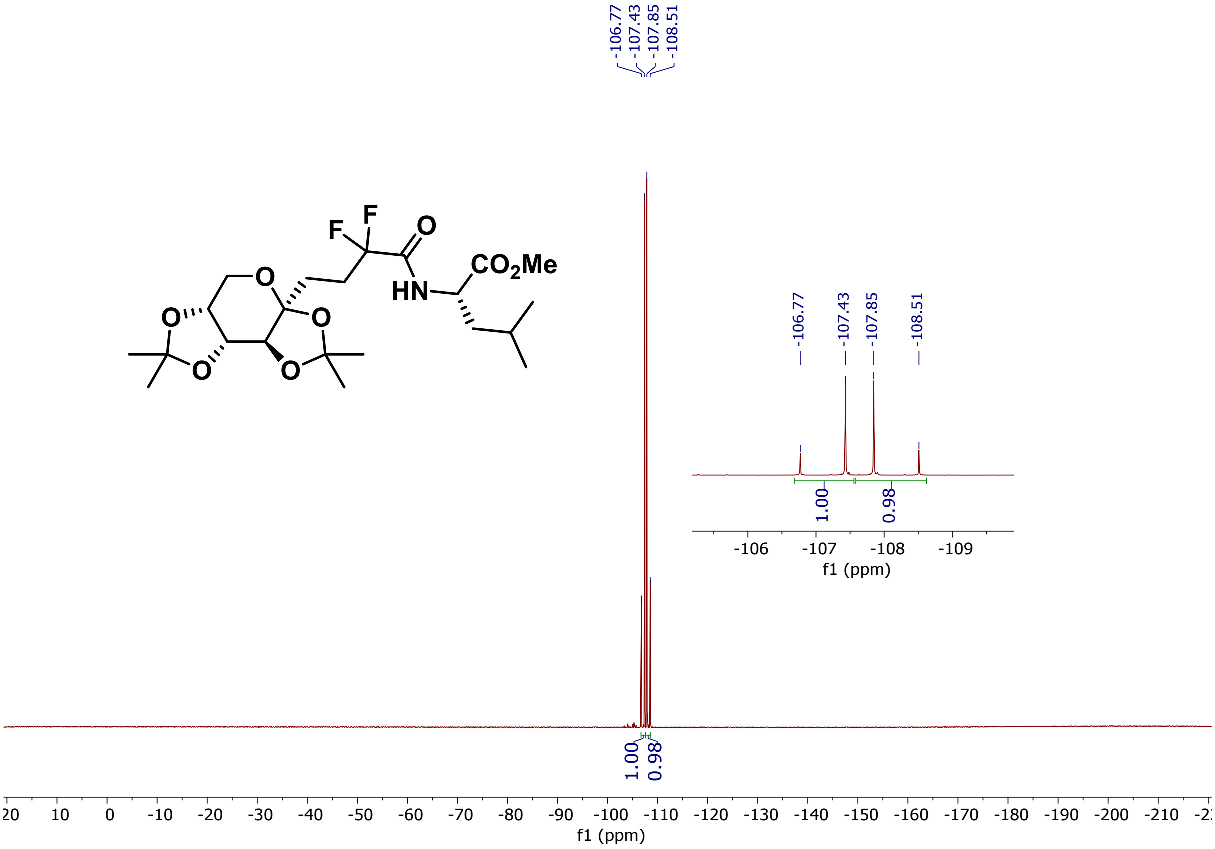

^{19}F NMR (377 MHz, acetone-d_6) of compound 4a

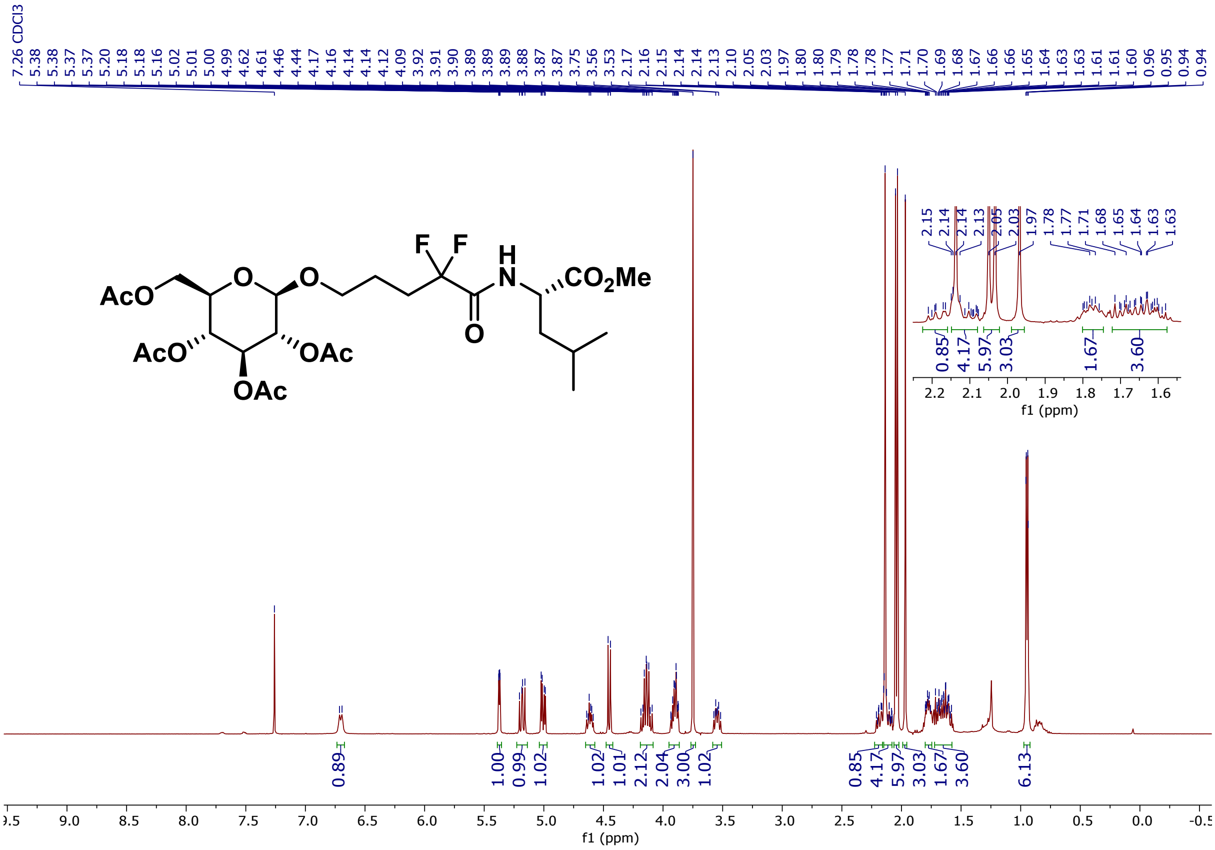

^{1}H NMR (400 MHz, $CDCl_3$) of compound 4b

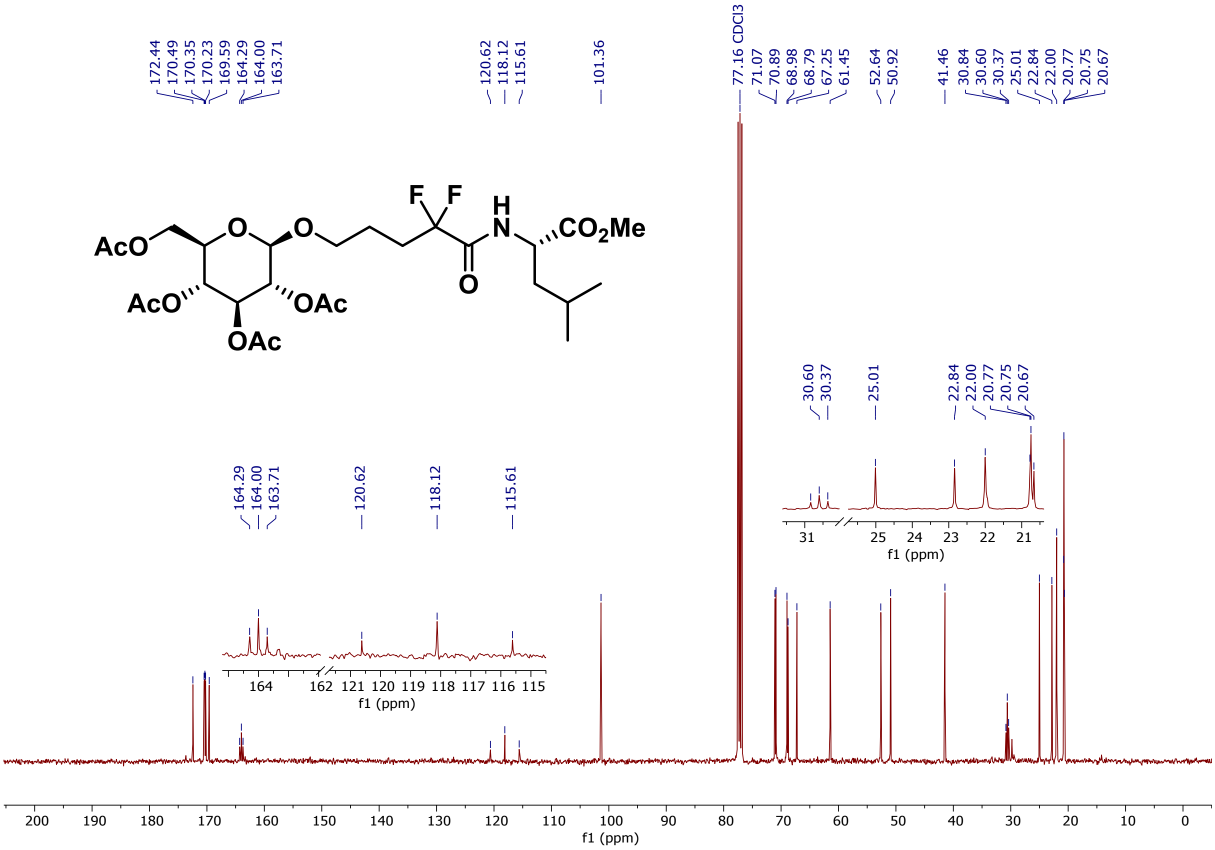

^{13}C NMR (101 MHz, $CDCl_3$) of compound 4b

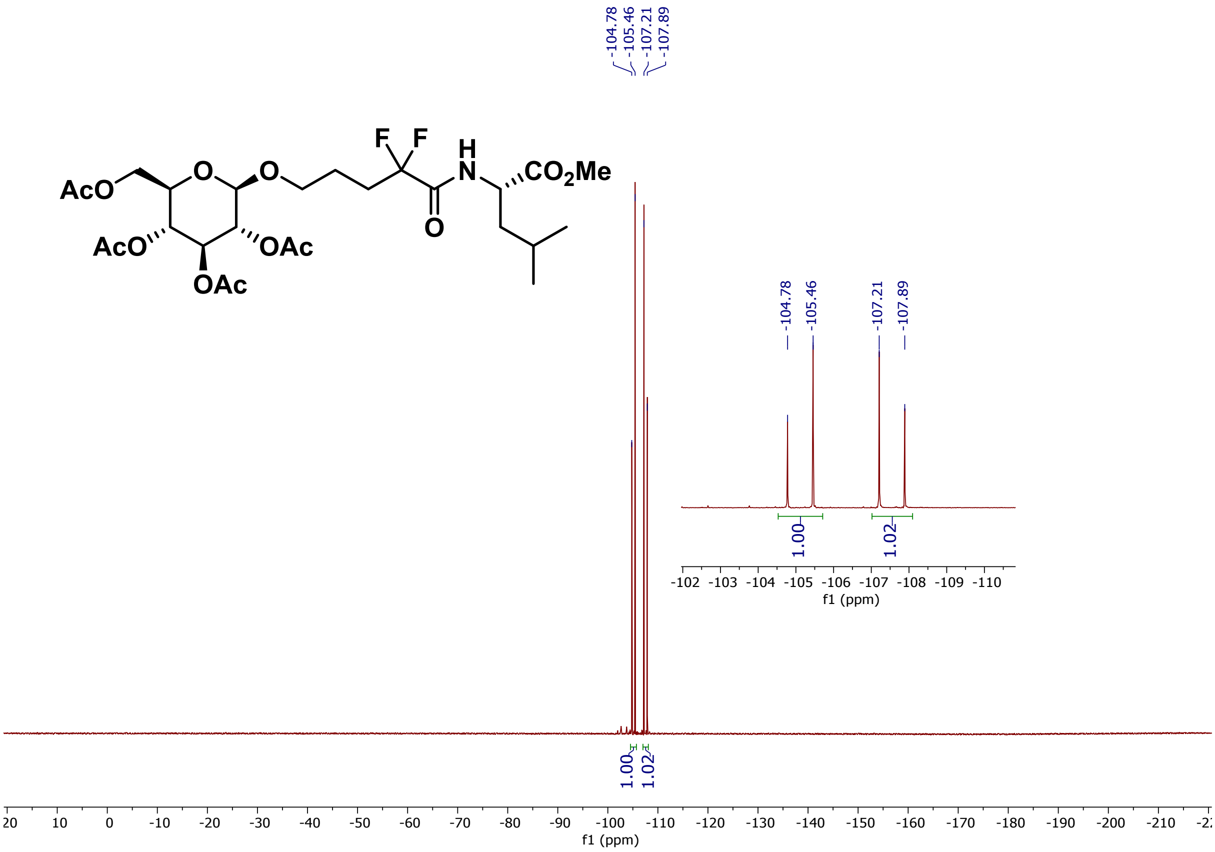

^{19}F NMR (377 MHz, $CDCl_3$) of compound 4b

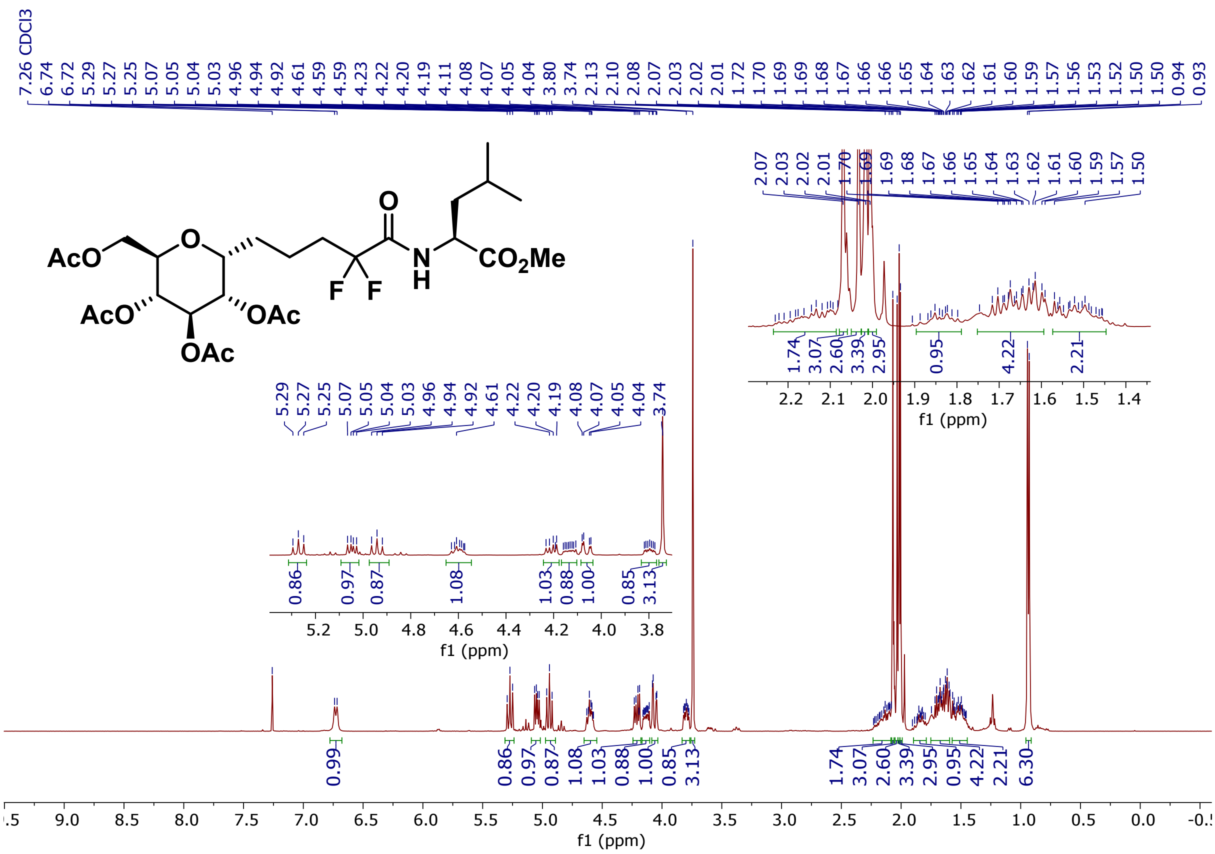

^{1}H NMR (400 MHz, $CDCl_3$) of compound 4c

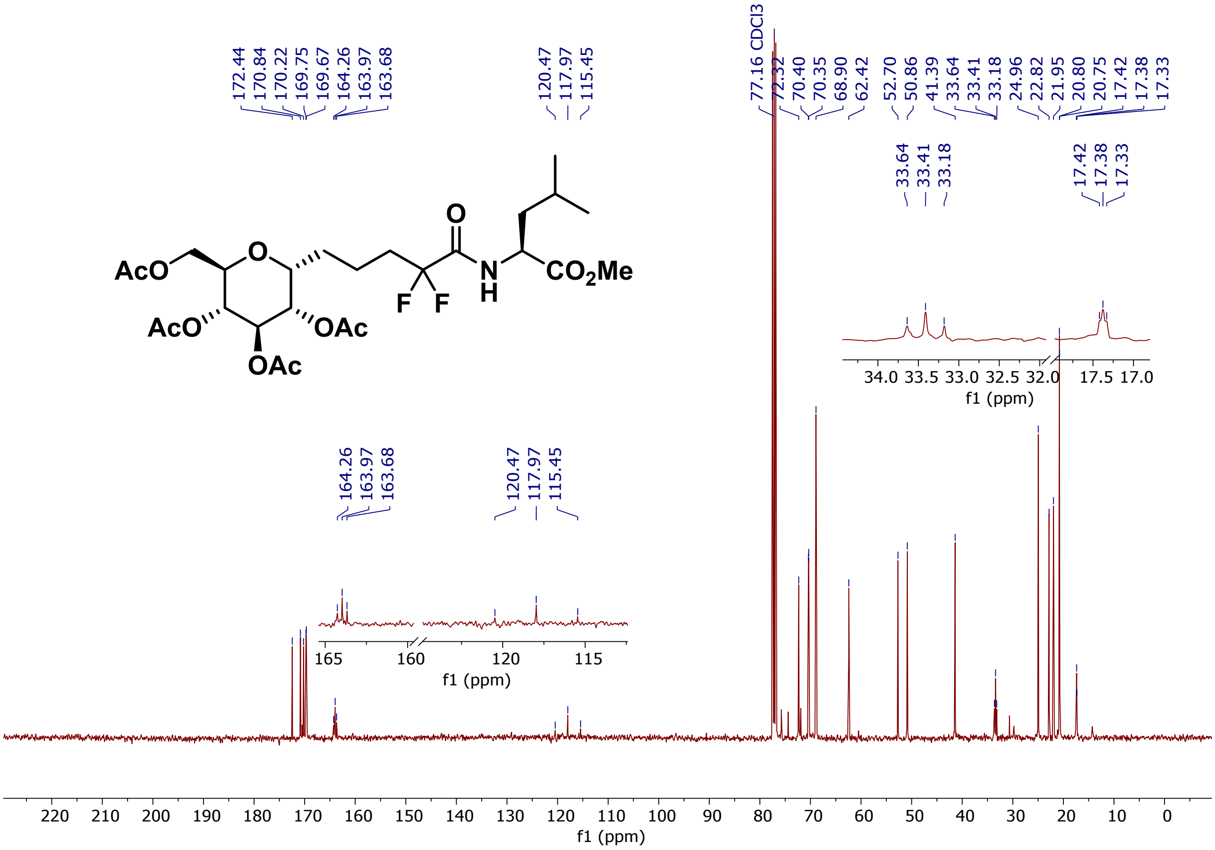

^{13}C NMR (101 MHz, $CDCl_3$) of compound 4c

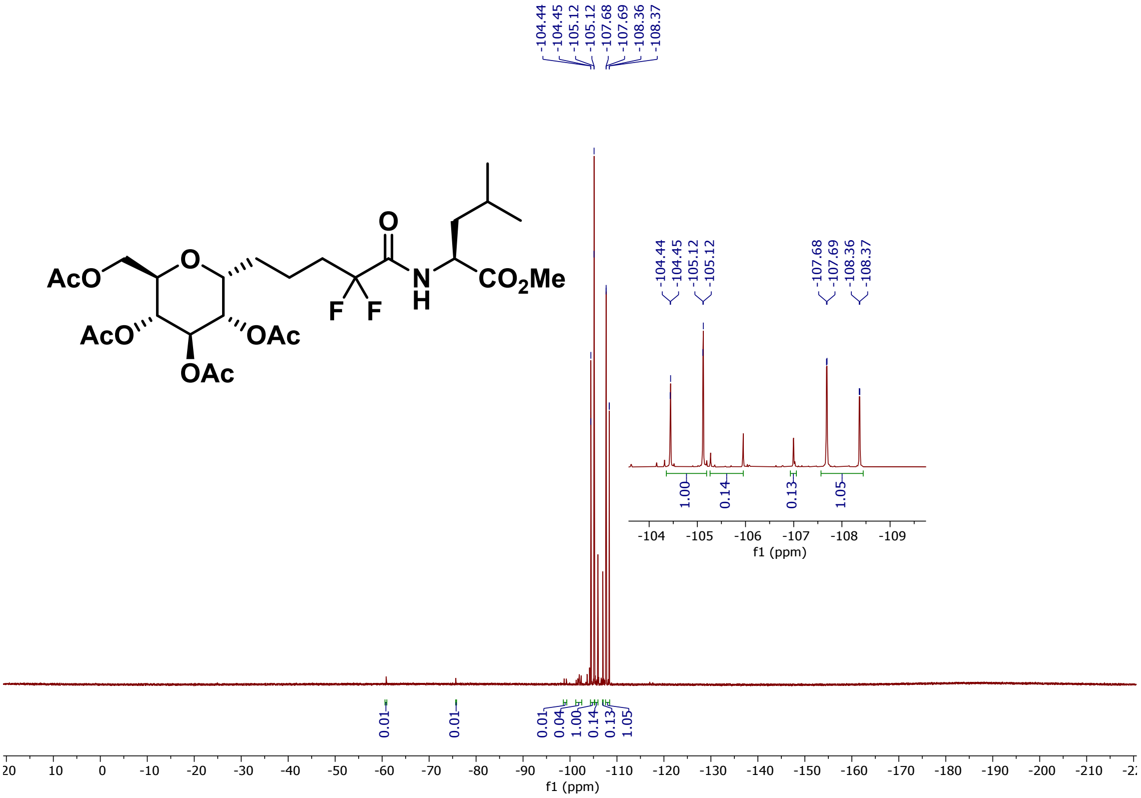

^{19}F NMR (377 MHz, $CDCl_3$) of compound 4c

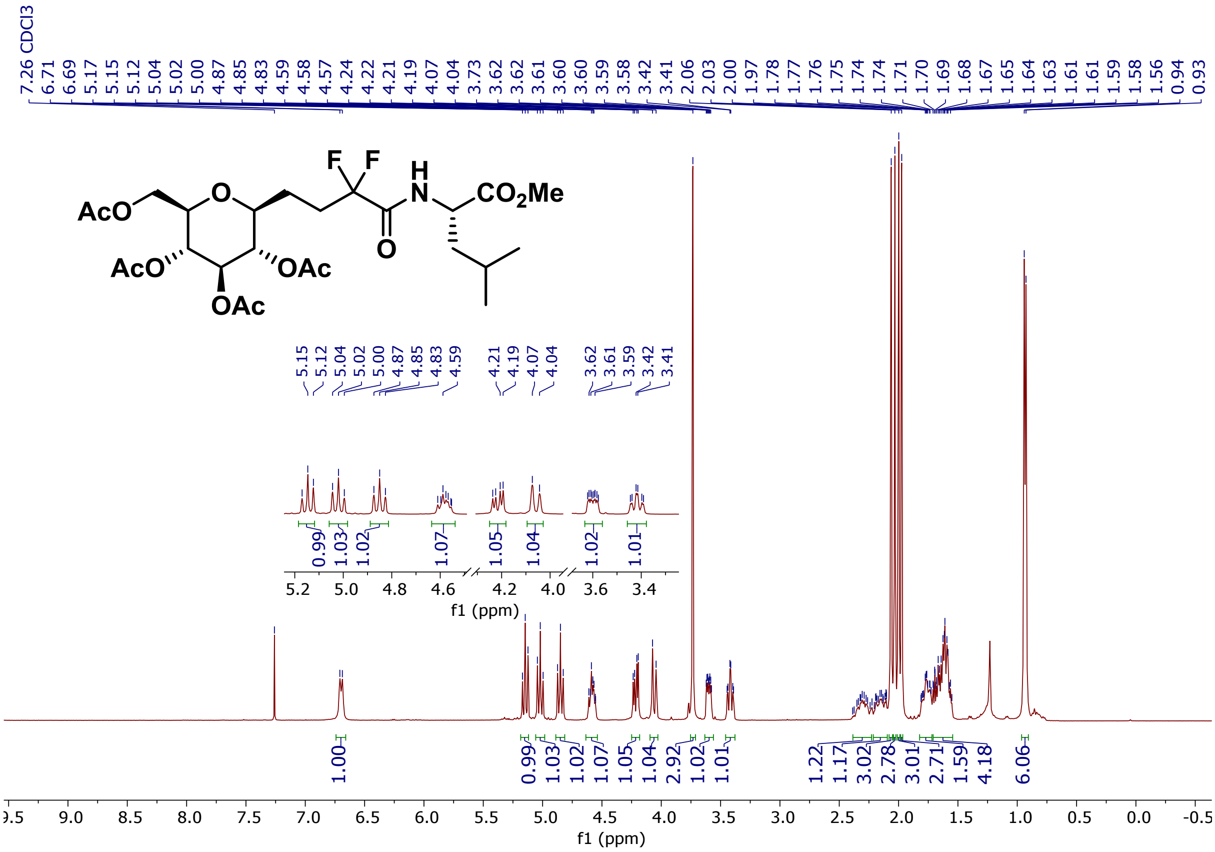

^{1}H NMR (400 MHz, $CDCl_3$) of compound 4d

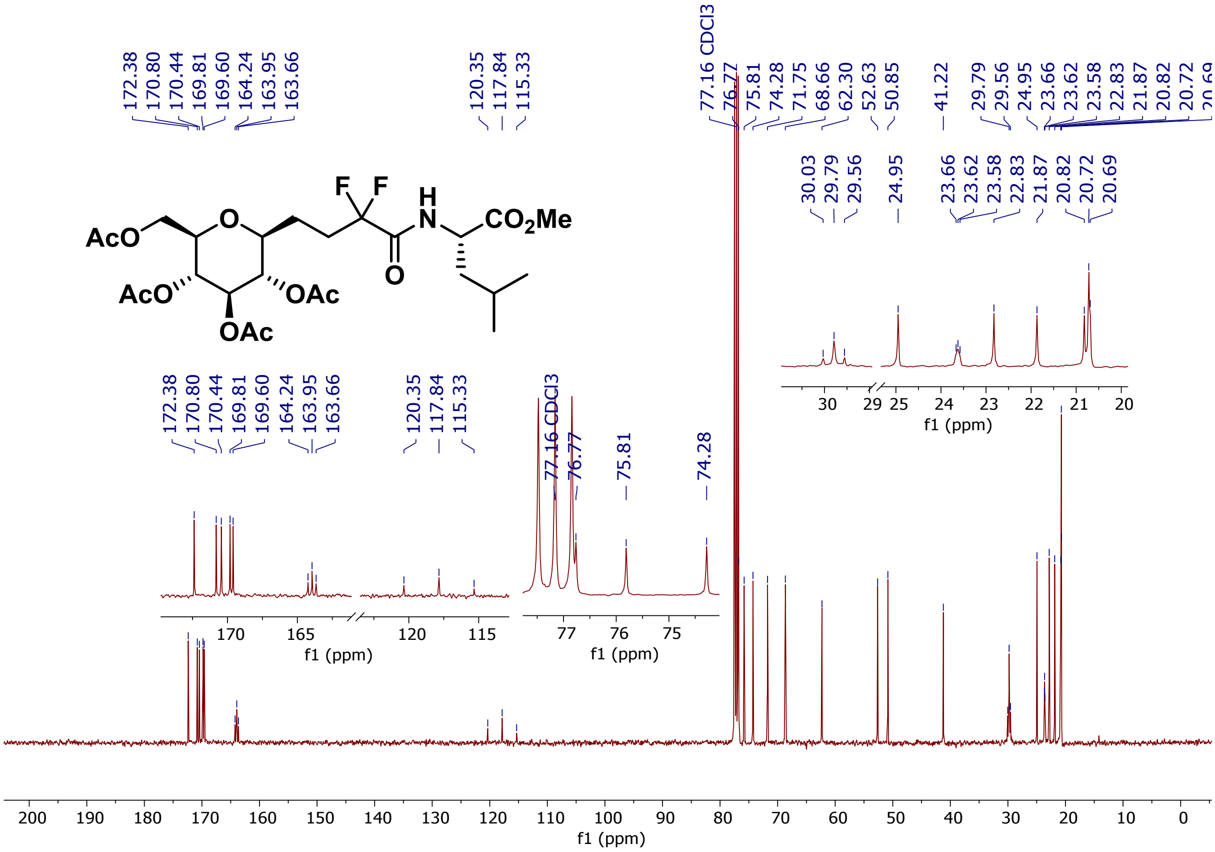

^{13}C NMR (101 MHz, $CDCl_3$) of compound 4d

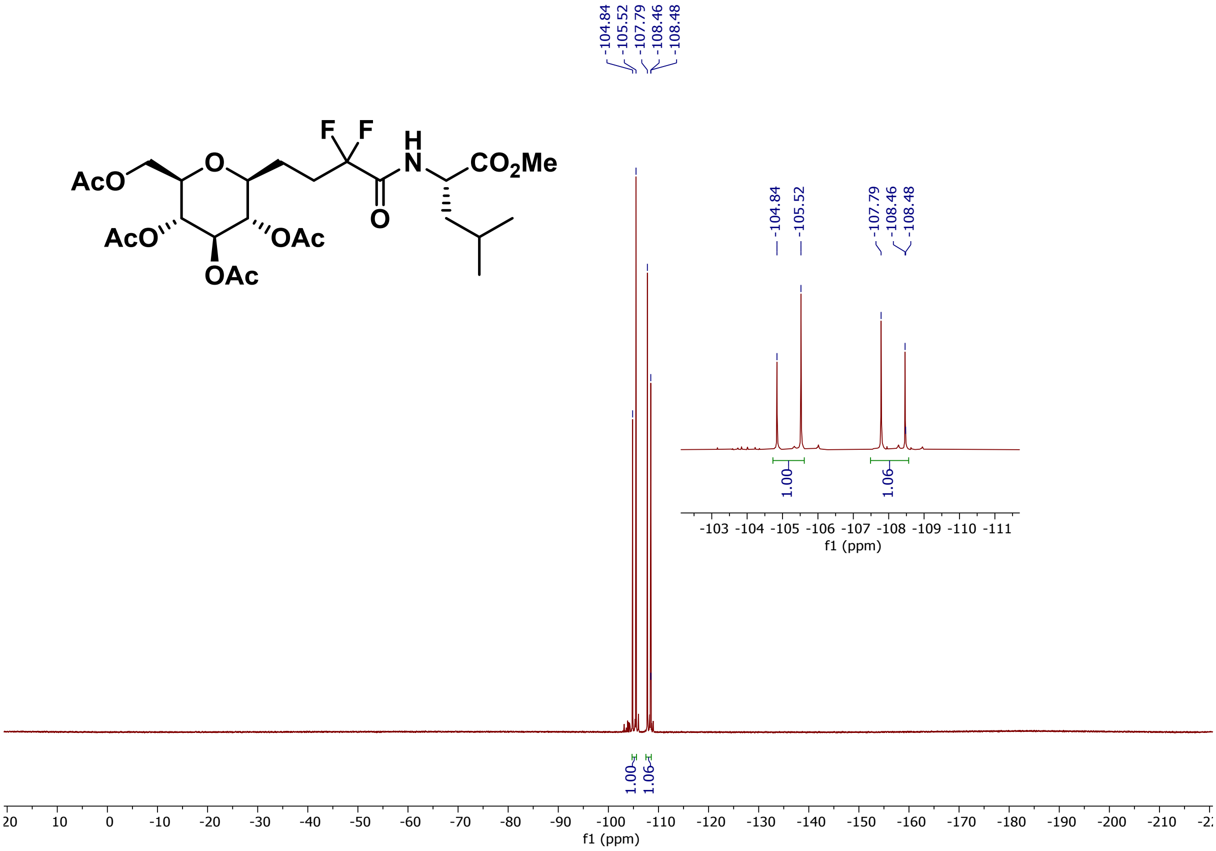

^{19}F NMR (377 MHz, $CDCl_3$) of compound 4d

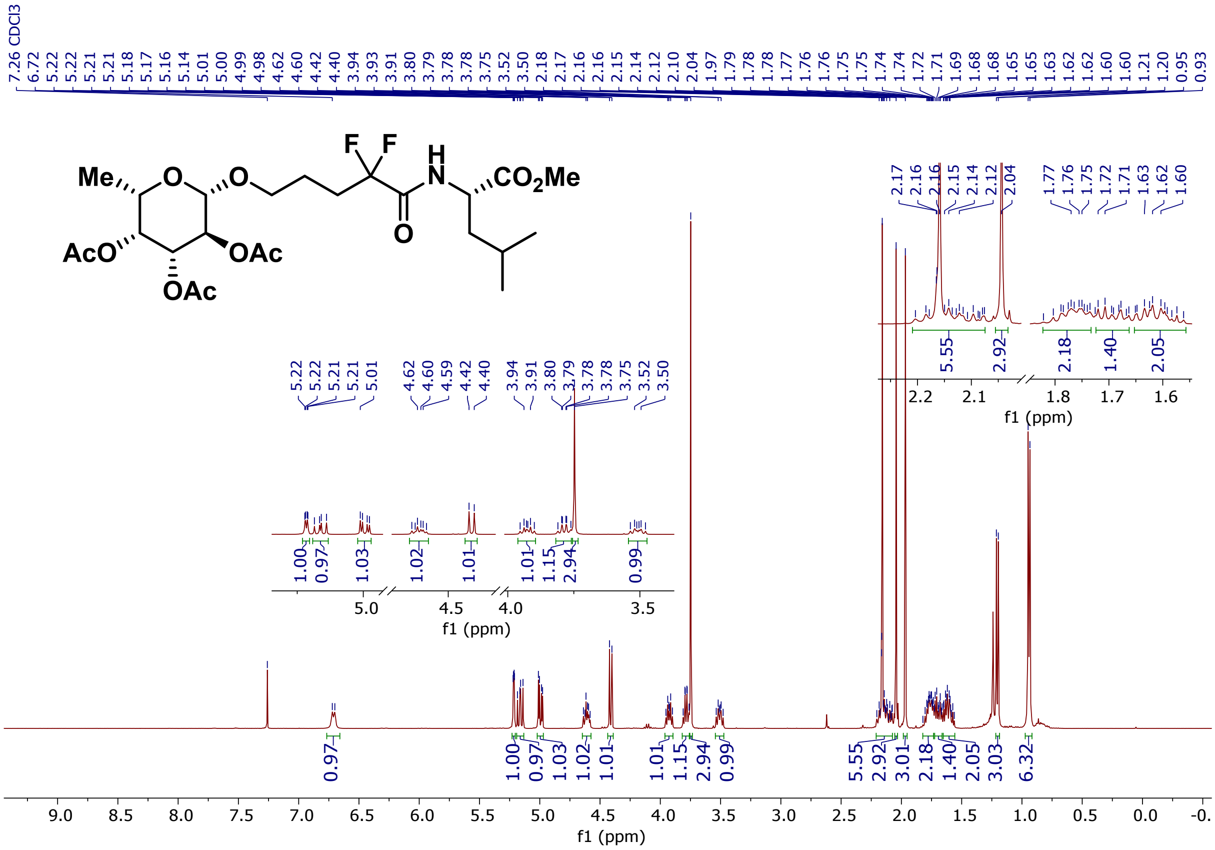

^{1}H NMR (400 MHz, $CDCl_3$) of compound 4e

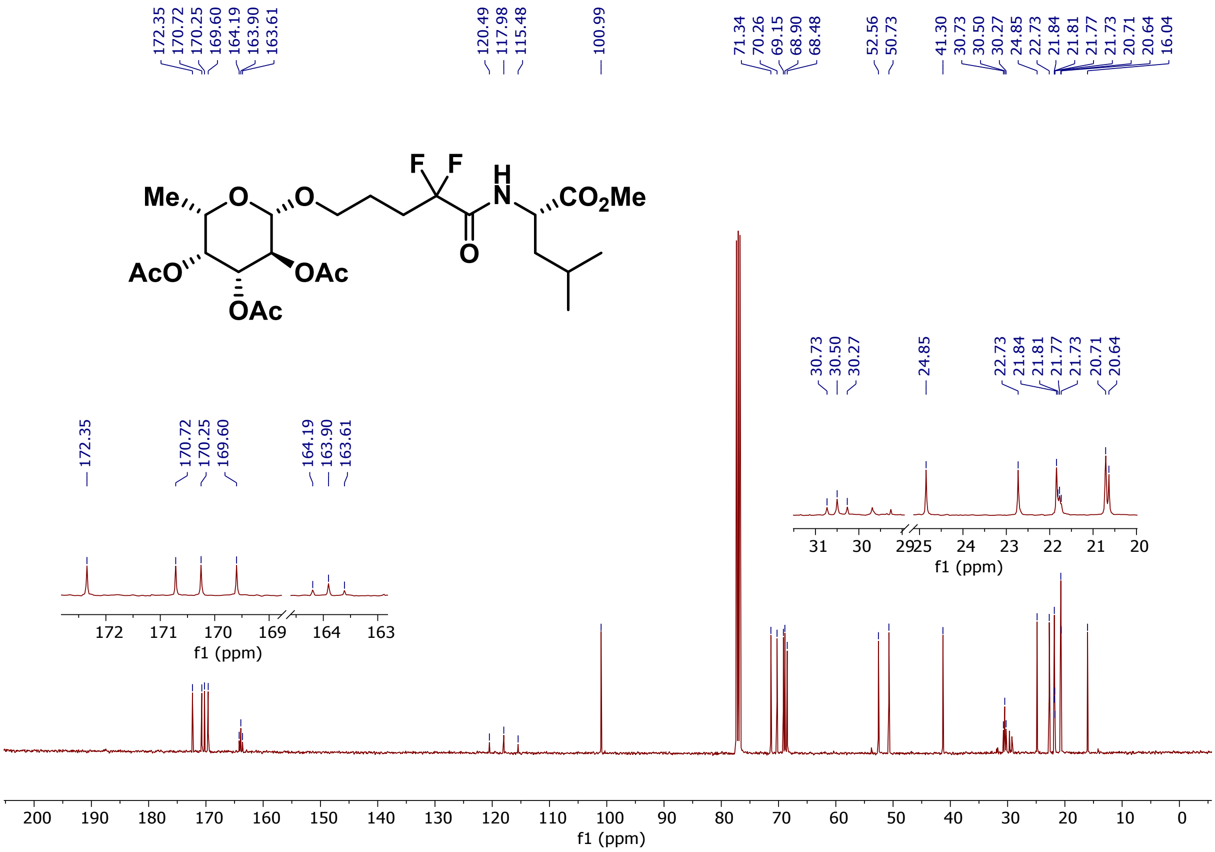

^{13}C NMR (101 MHz, $CDCl_3$) of compound 4e

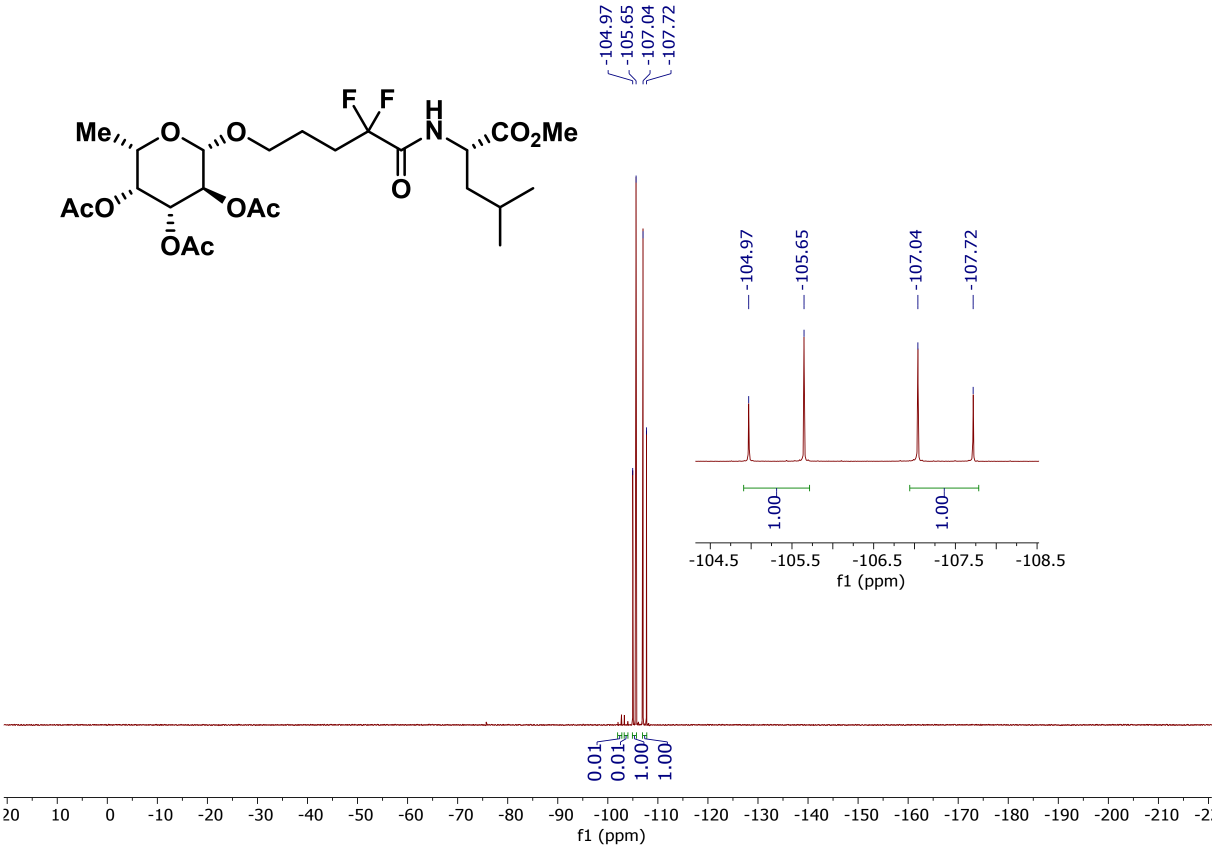

^{19}F NMR (377 MHz, $CDCl_3$) of compound 4e

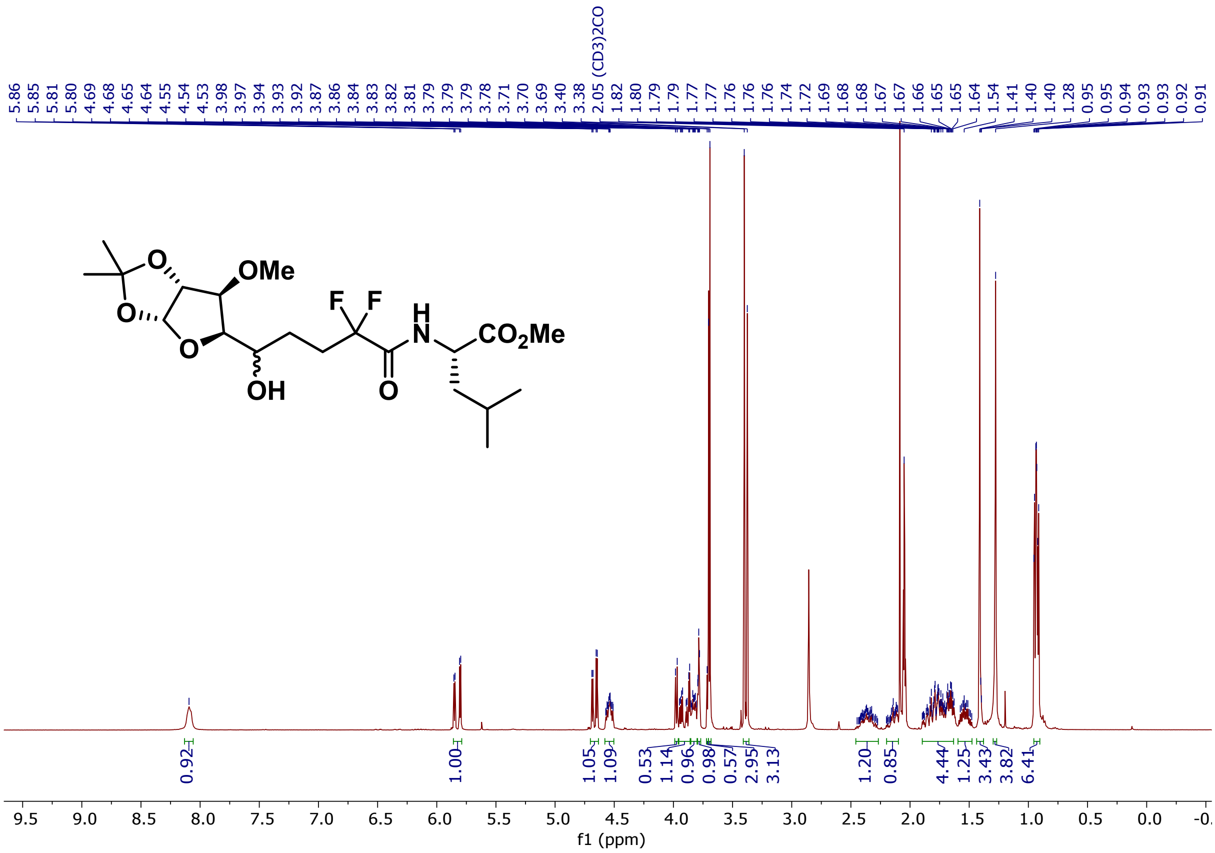

^{1}H NMR (400 MHz, acetone-d$_6$) of compound 4f

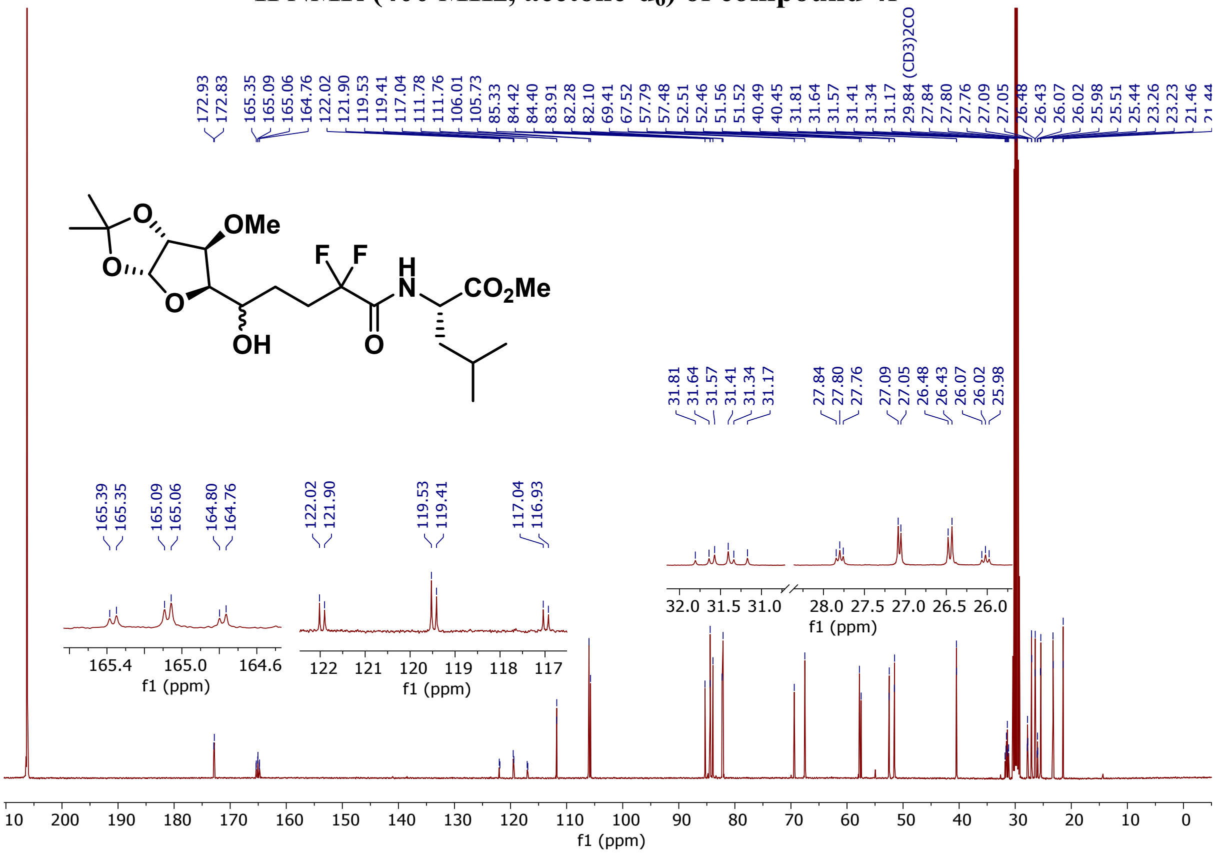

^{13}C NMR (101 MHz, acetone-d$_6$) of compound 4f

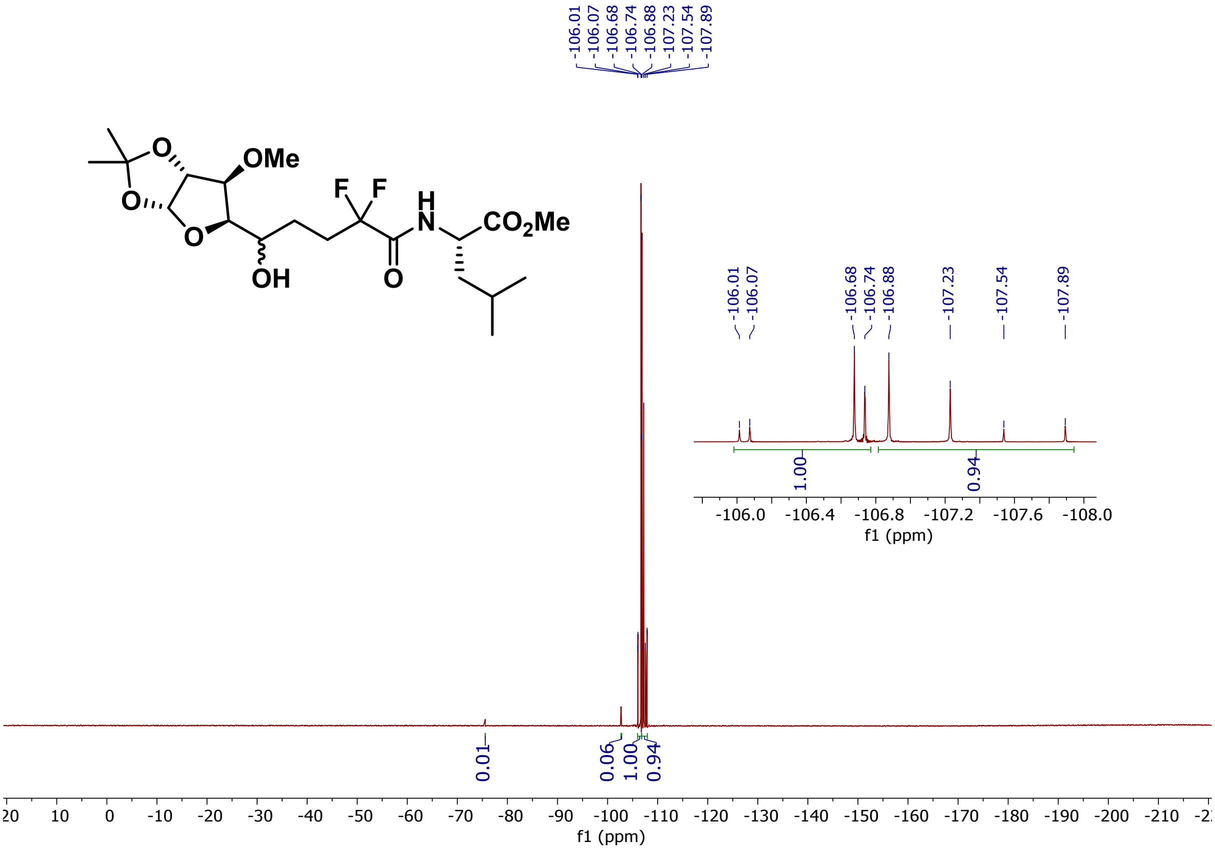

^{19}F NMR (377 MHz, acetone-d$_6$) of compound 4f

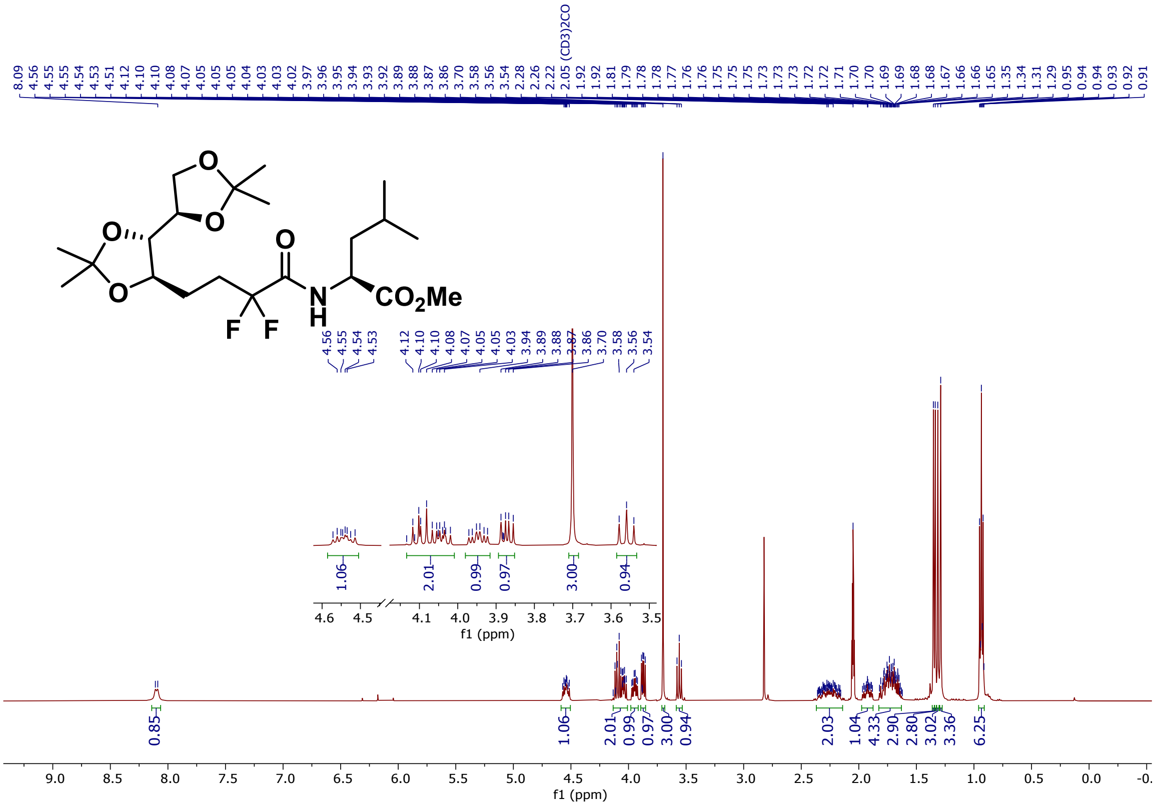

^{1}H NMR (400 MHz, acetone-d$_6$) of compound 4g

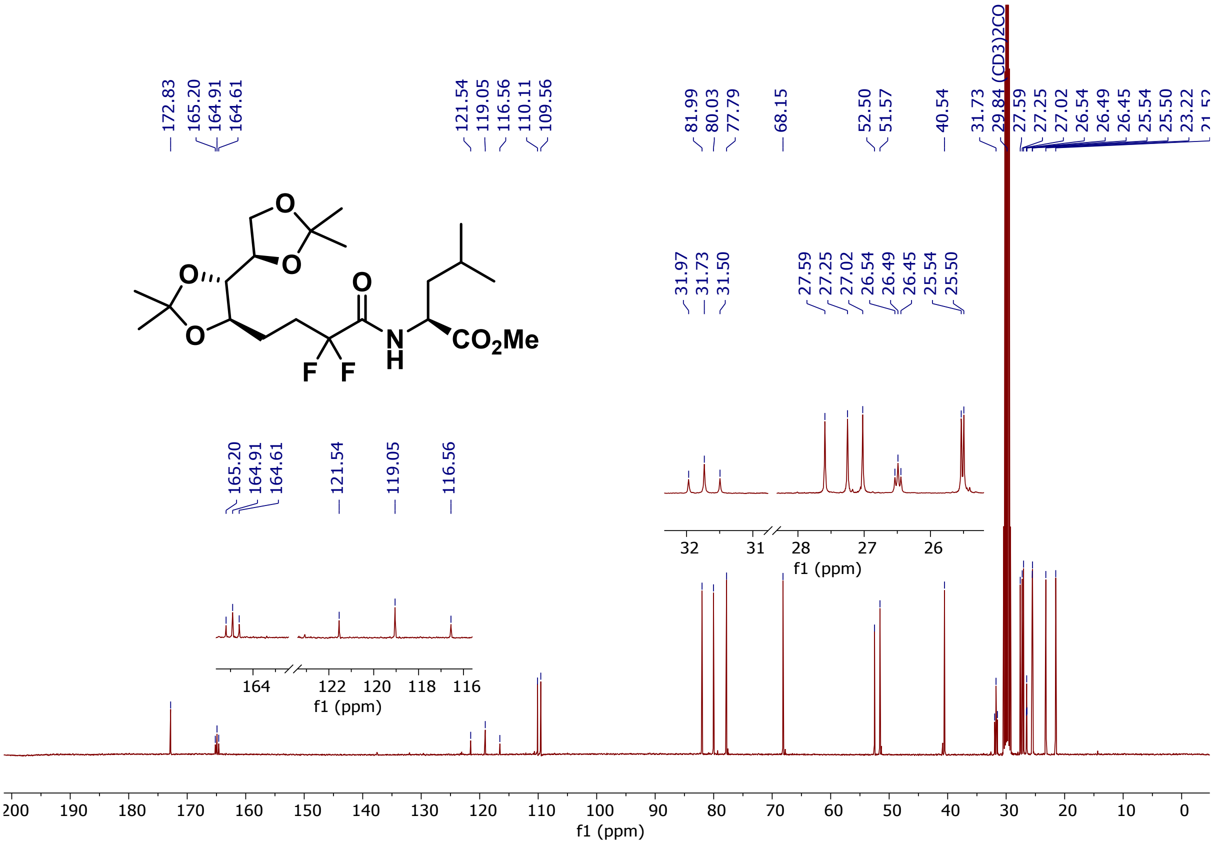

^{13}C NMR (101 MHz, acetone-d$_6$) of compound 4g

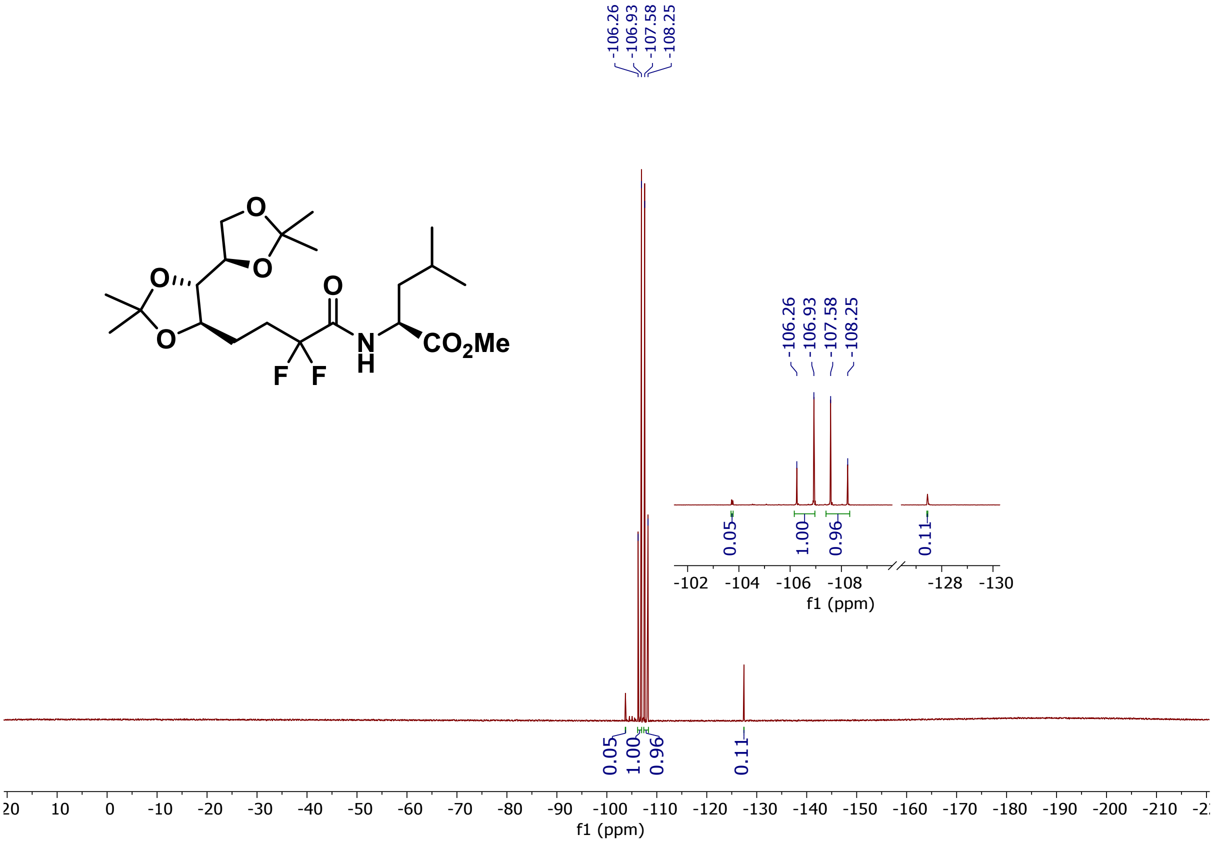

^{19}F NMR (377 MHz, acetone-d$_6$) of compound 4g

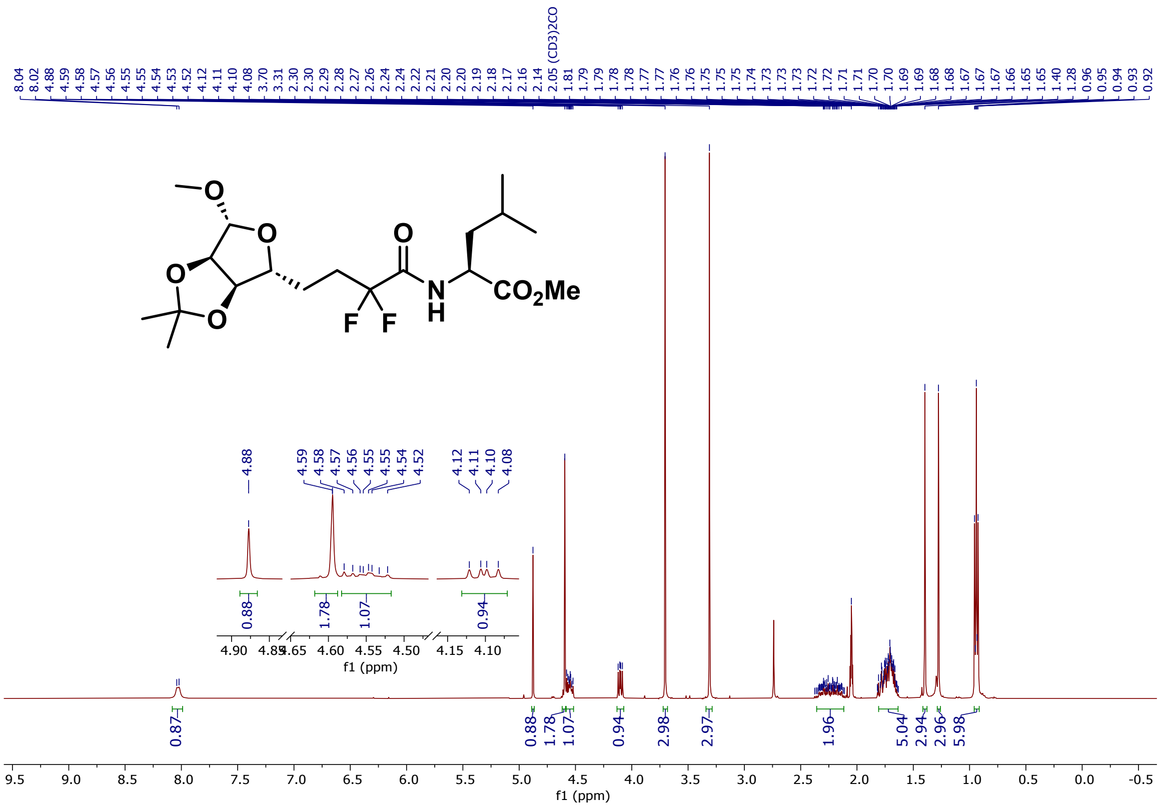

^{1}H NMR (400 MHz, acetone-d_6) of compound 4h

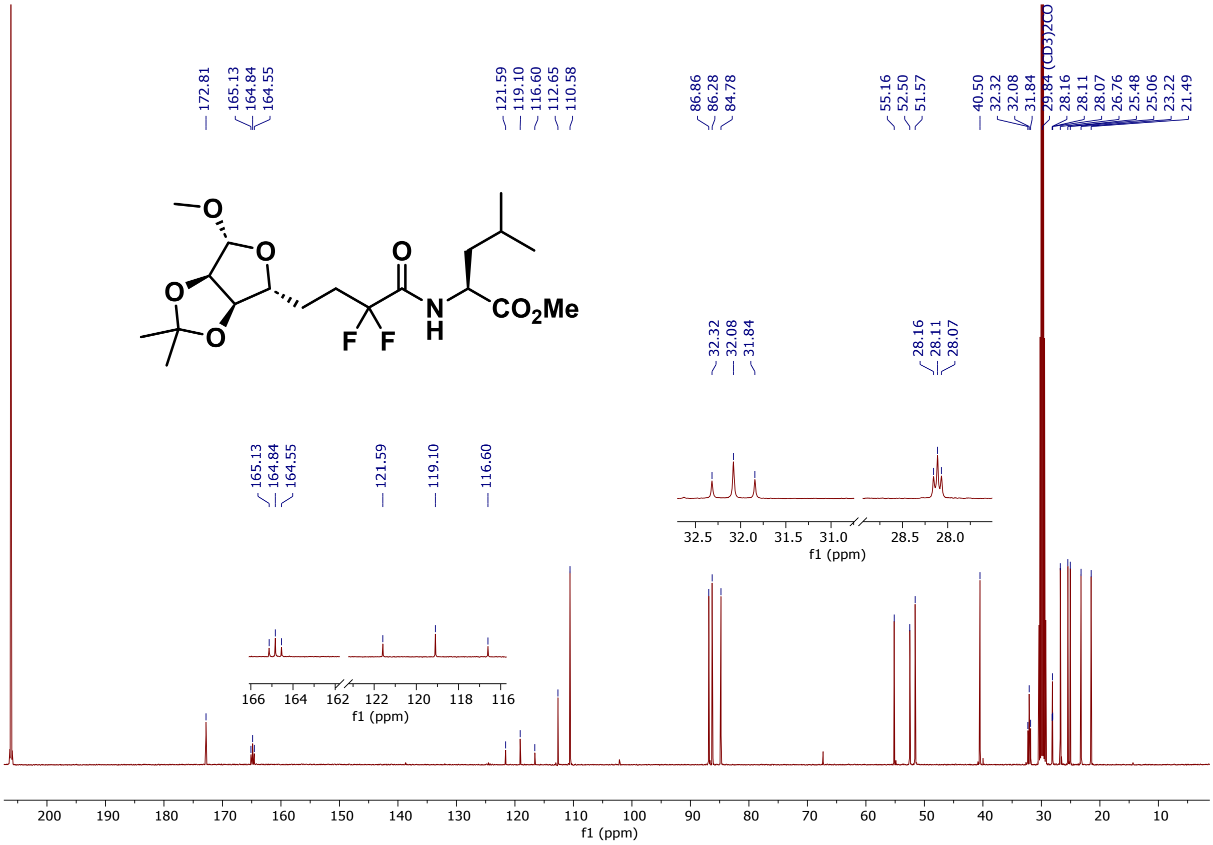

^{13}C NMR (101 MHz, acetone-d_6) of compound 4h

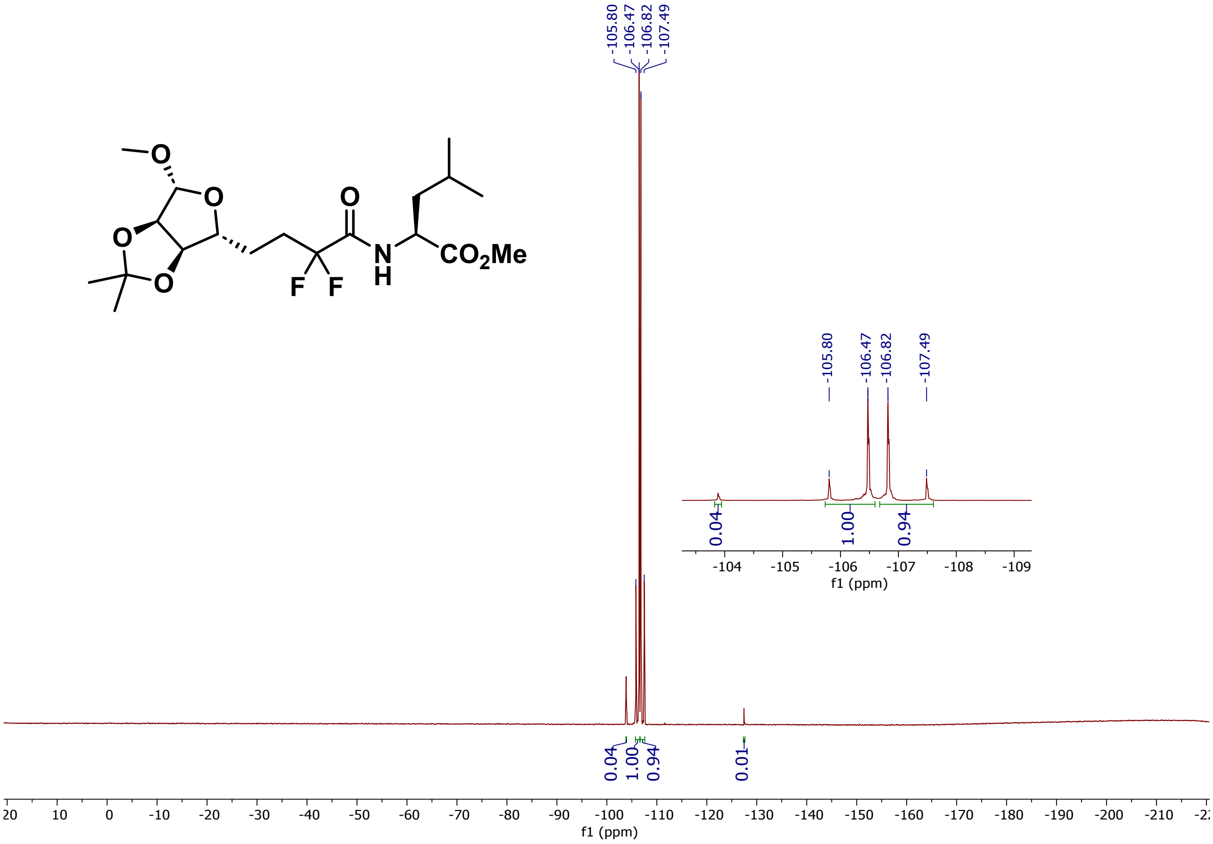

^{19}F NMR (377 MHz, acetone-d$_6$) of compound 4h

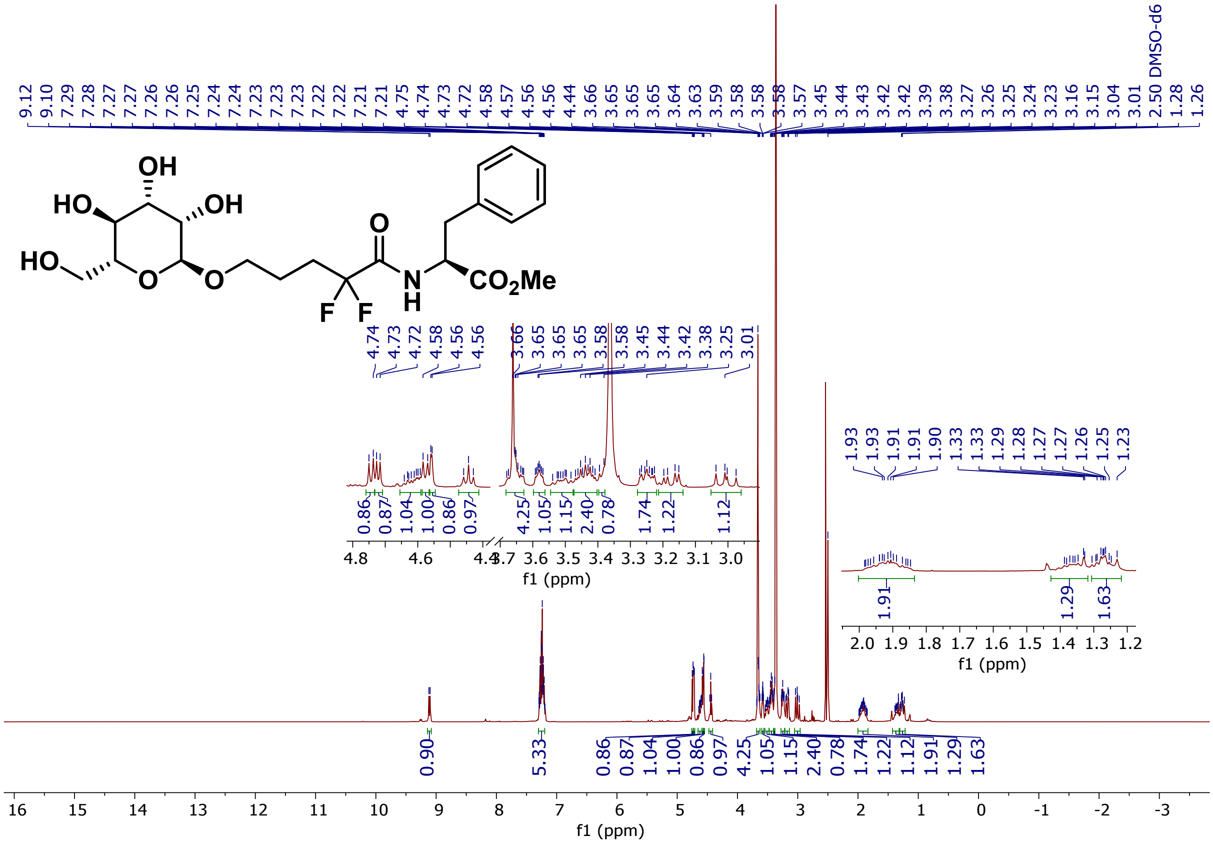

^{1}H NMR (400 MHz, DMSO-d$_6$) of compound 4i

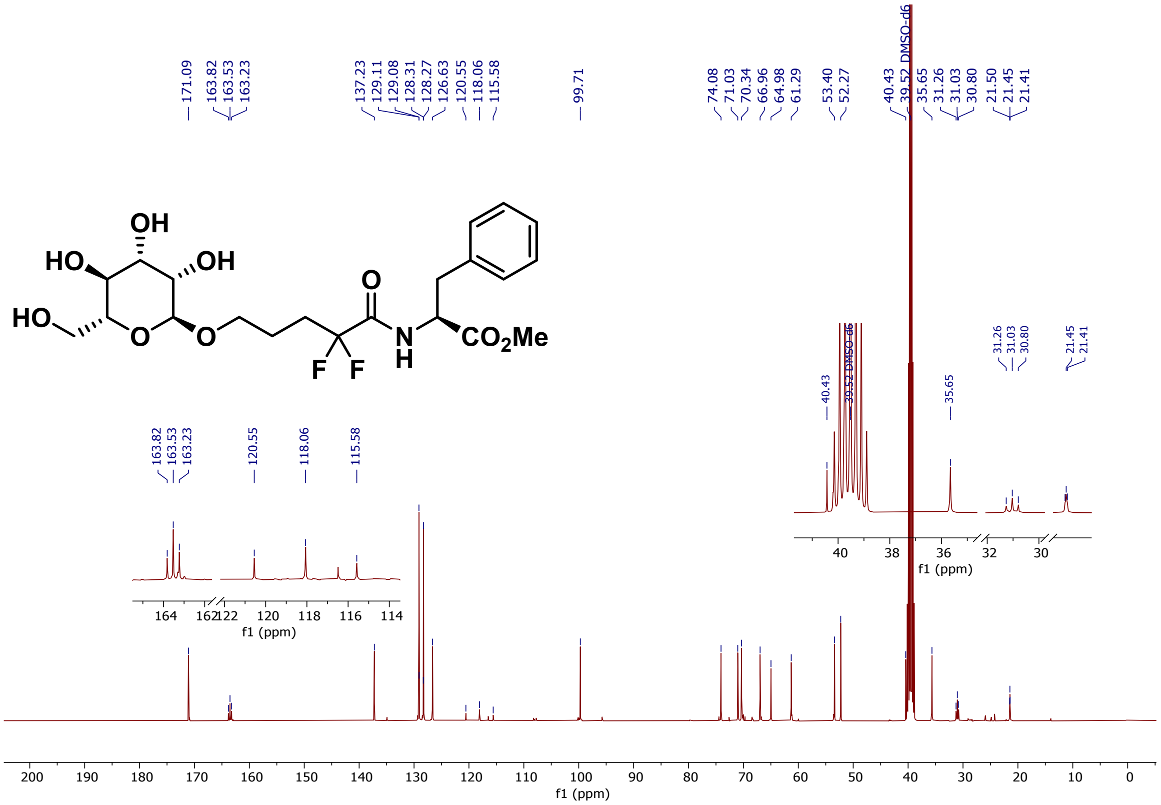

^{13}C NMR (101 MHz, DMSO-d$_6$) of compound 4i

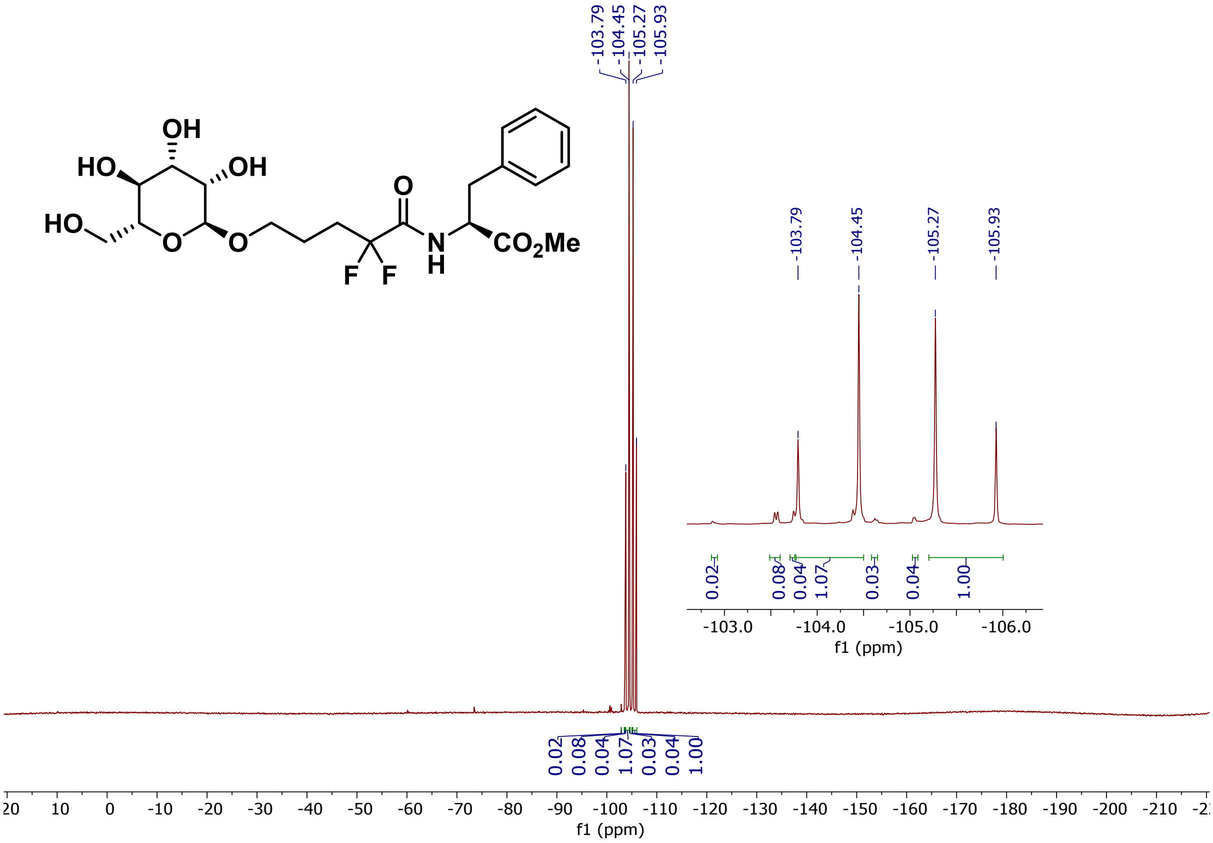

^{19}F NMR (377 MHz, DMSO-d$_6$) of compound 4i

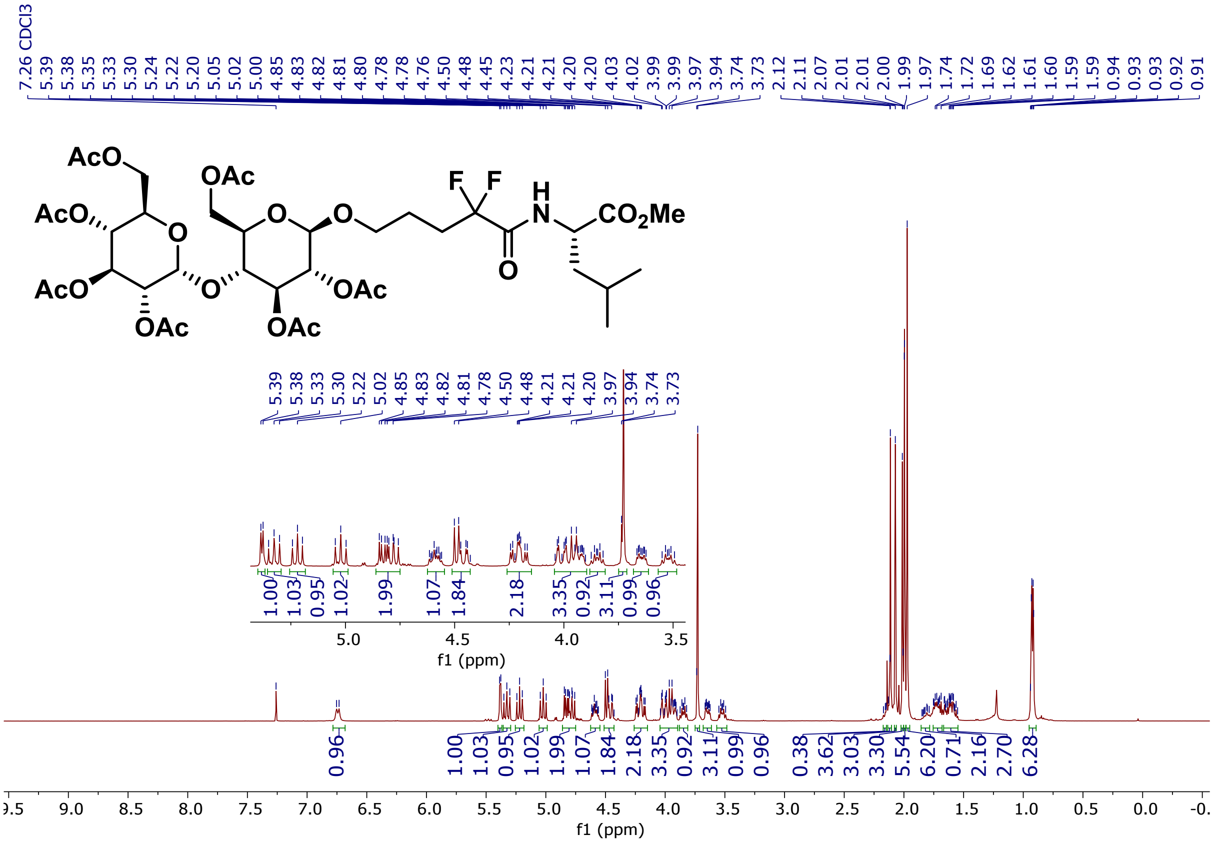

^{1}H NMR (400 MHz, CDCl$_3$) of compound 4j

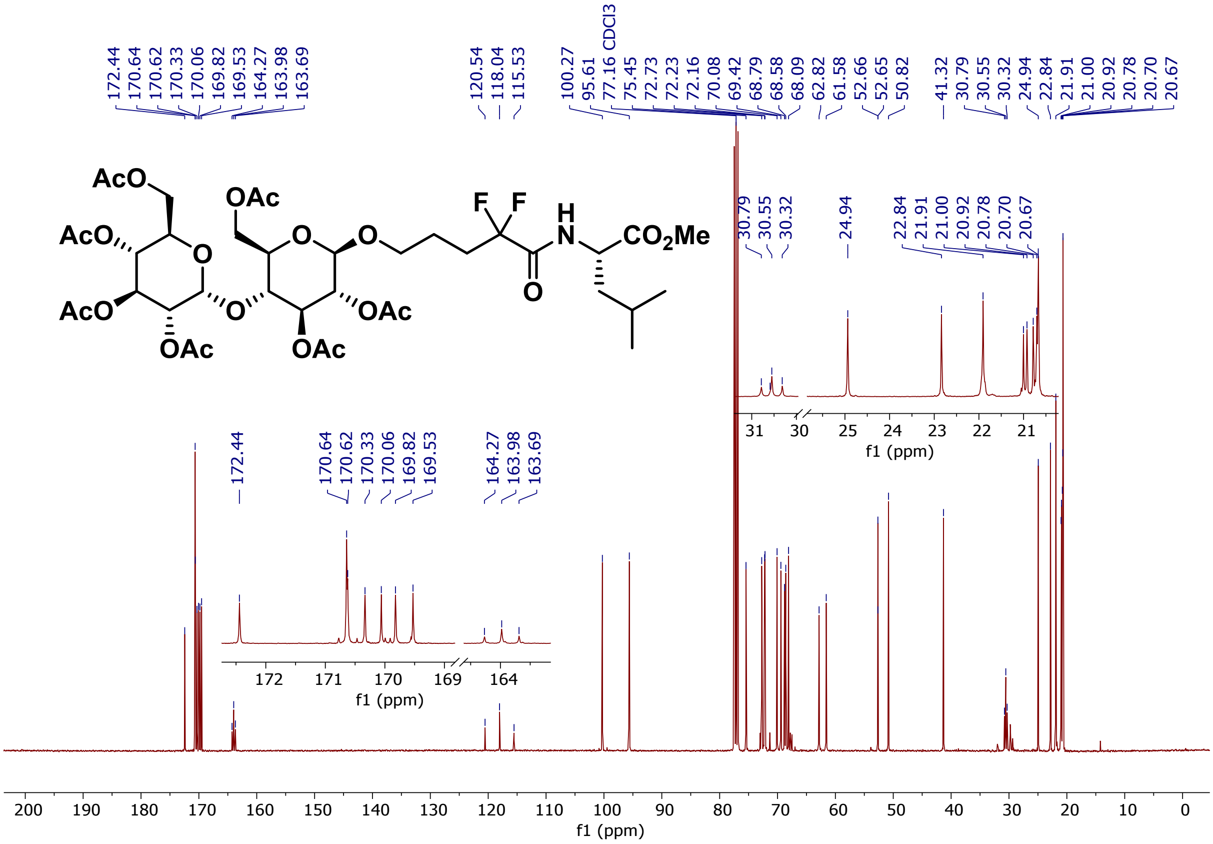

^{13}C NMR (101 MHz, CDCl$_3$) of compound 4j

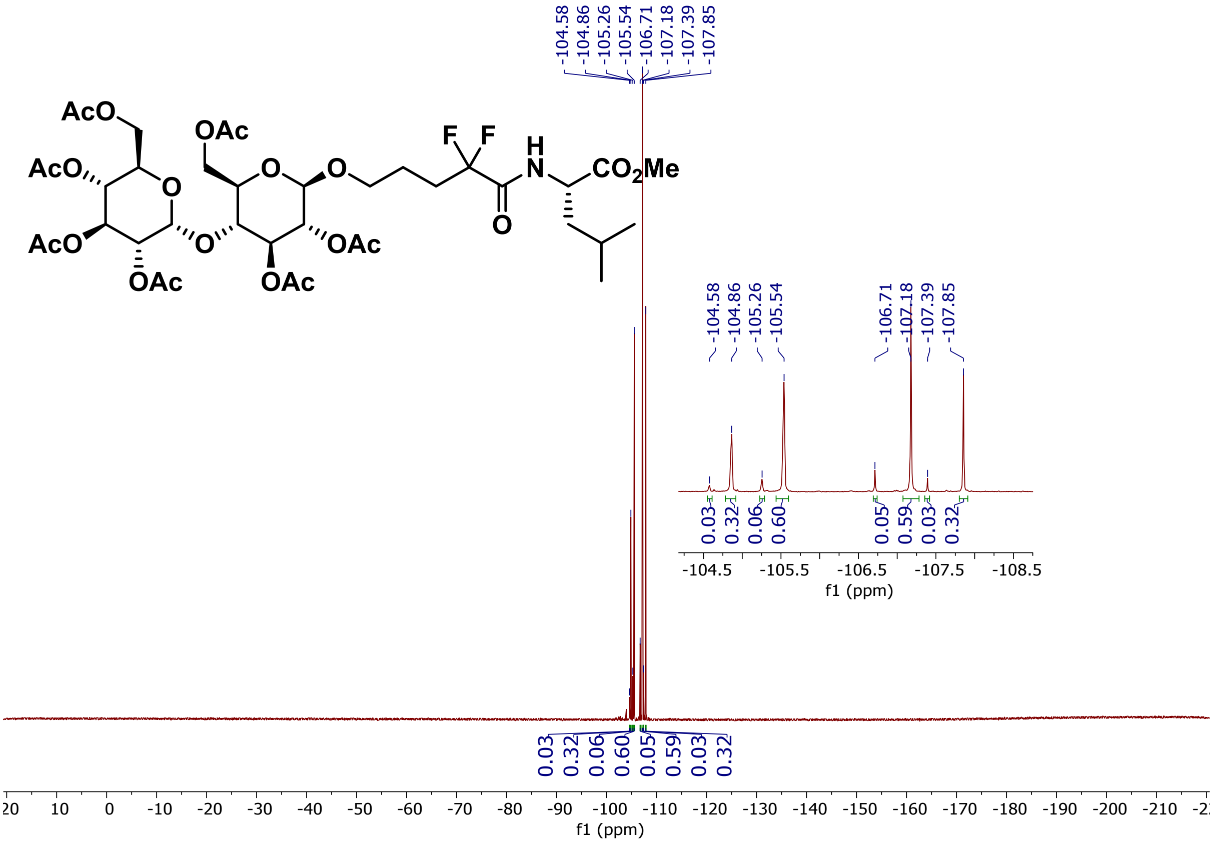

^{19}F NMR (377 MHz, $CDCl_3$) of compound 4j

16. Characterization Spectra of compounds 5a-5c

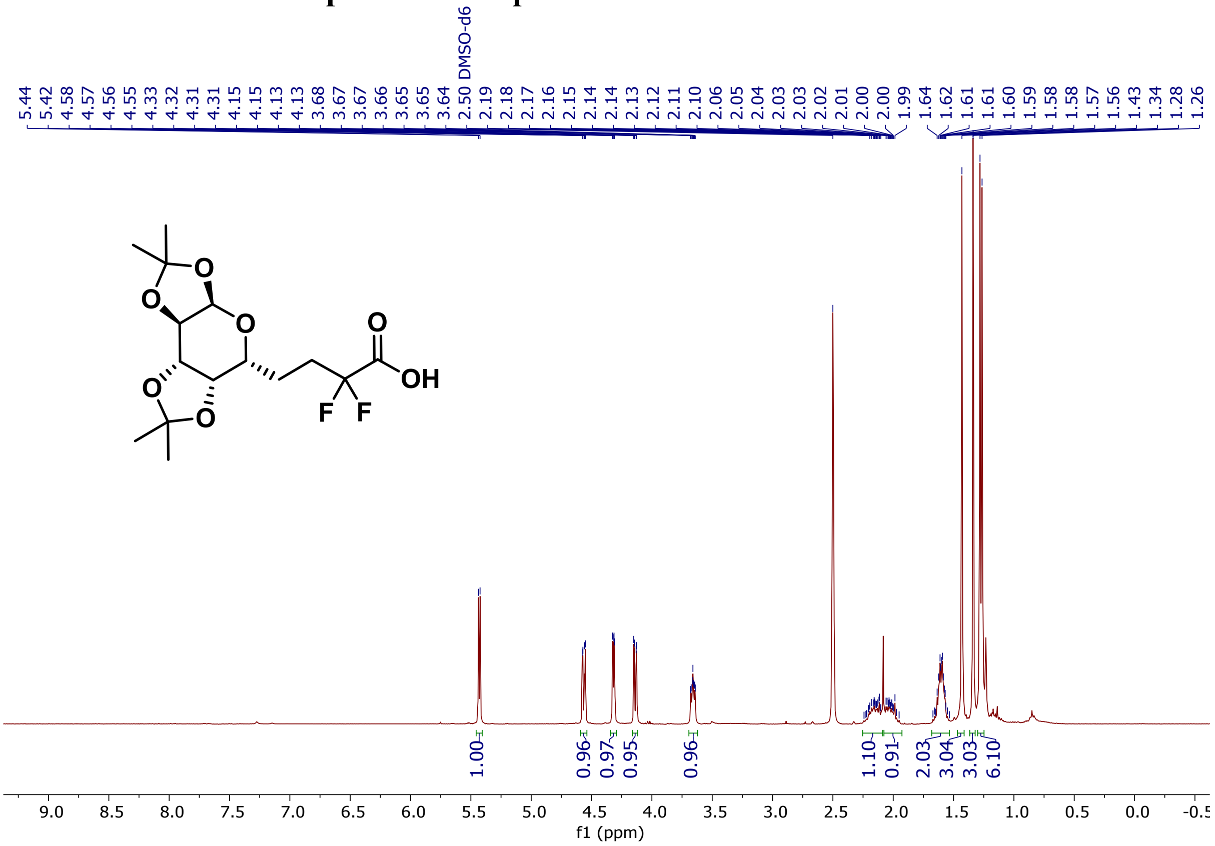

^{1}H NMR (400 MHz, DMSO-d_6) of compound 5a

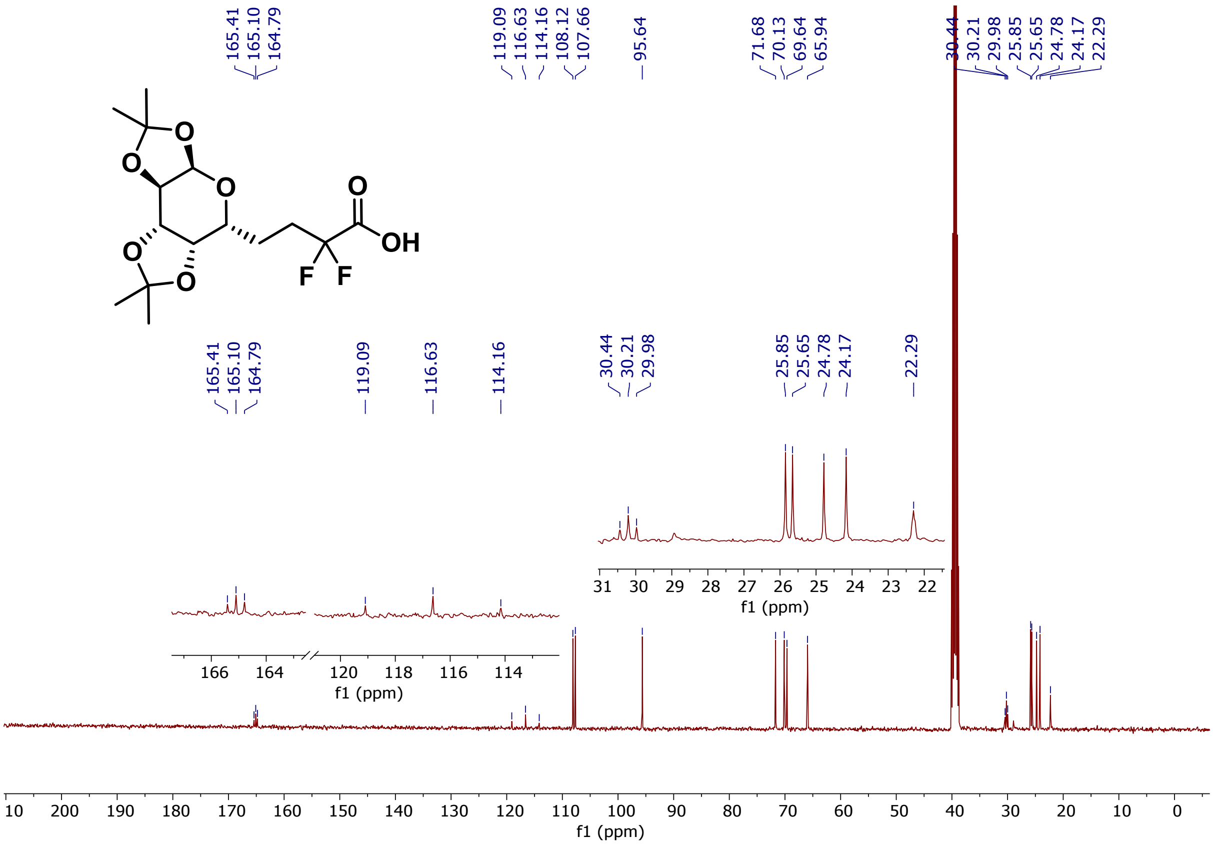

^{13}C NMR (101 MHz, DMSO-d$_6$) of compound 5a

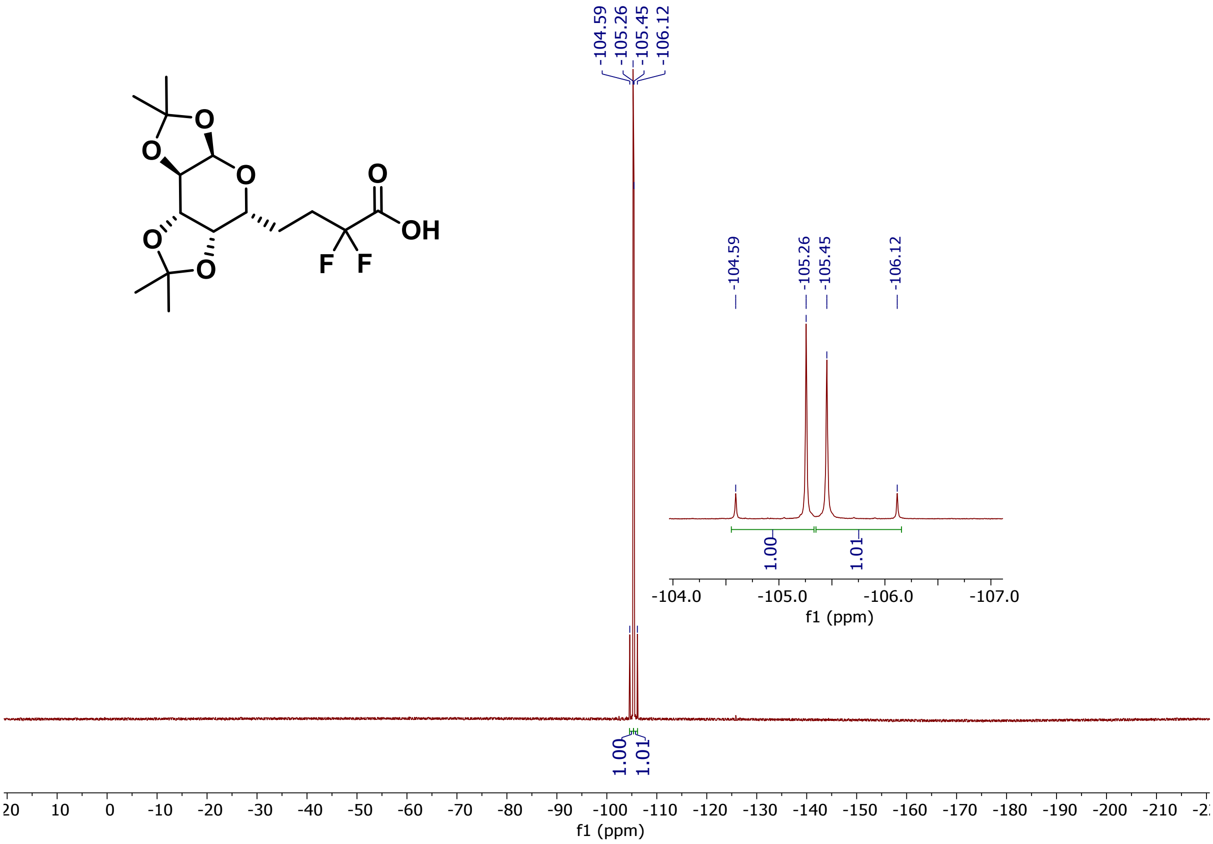

^{19}F NMR (376 MHz, DMSO-d$_6$) of compound 5a

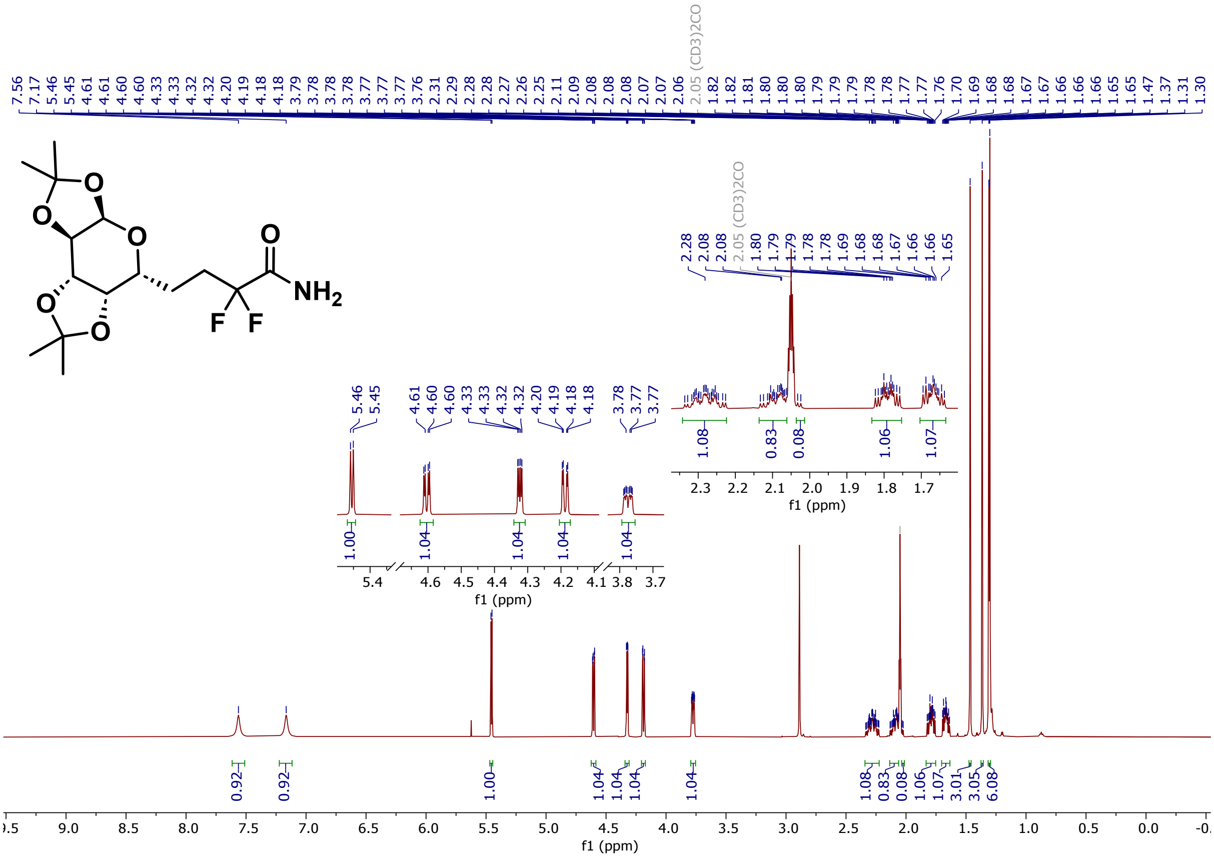

^{1}H NMR (600 MHz, acetone-d_6) of compound 5b

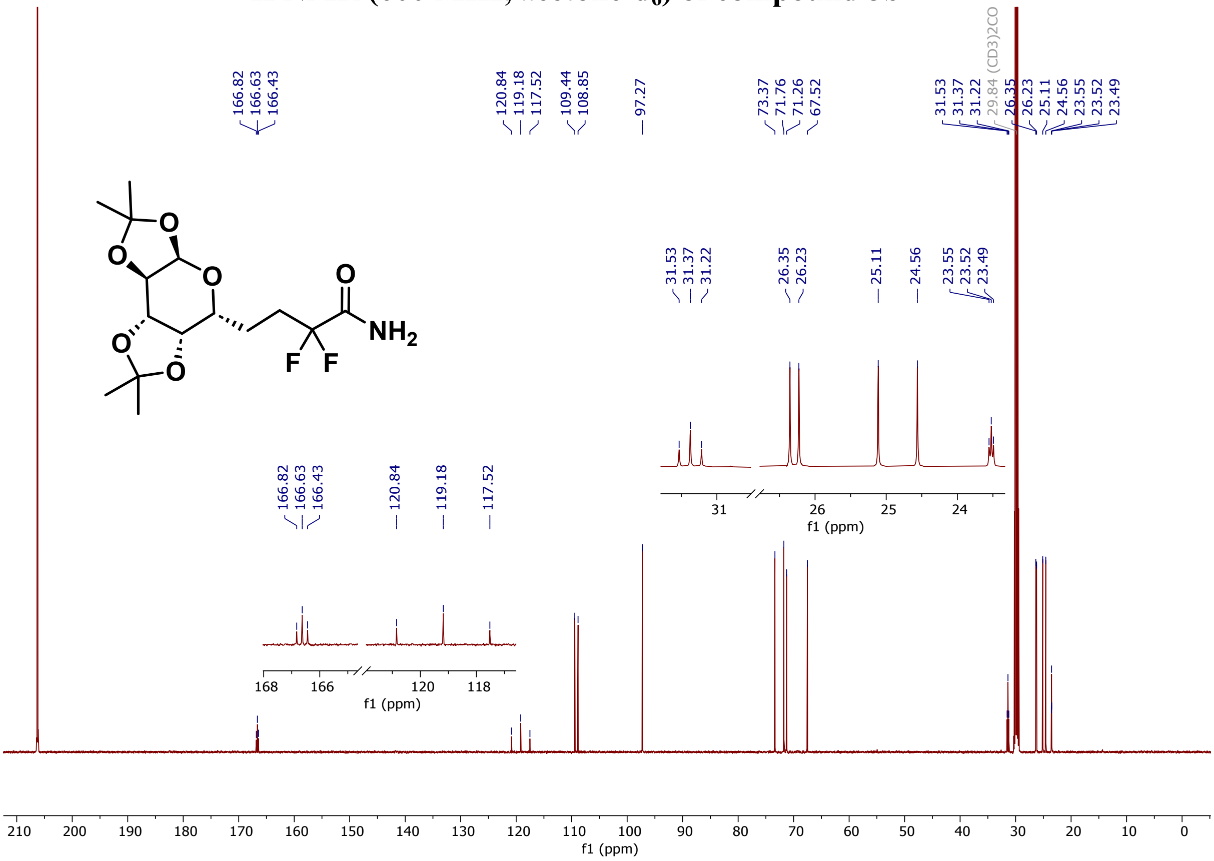

^{13}C NMR (151 MHz, acetone-d_6) of compound 5b

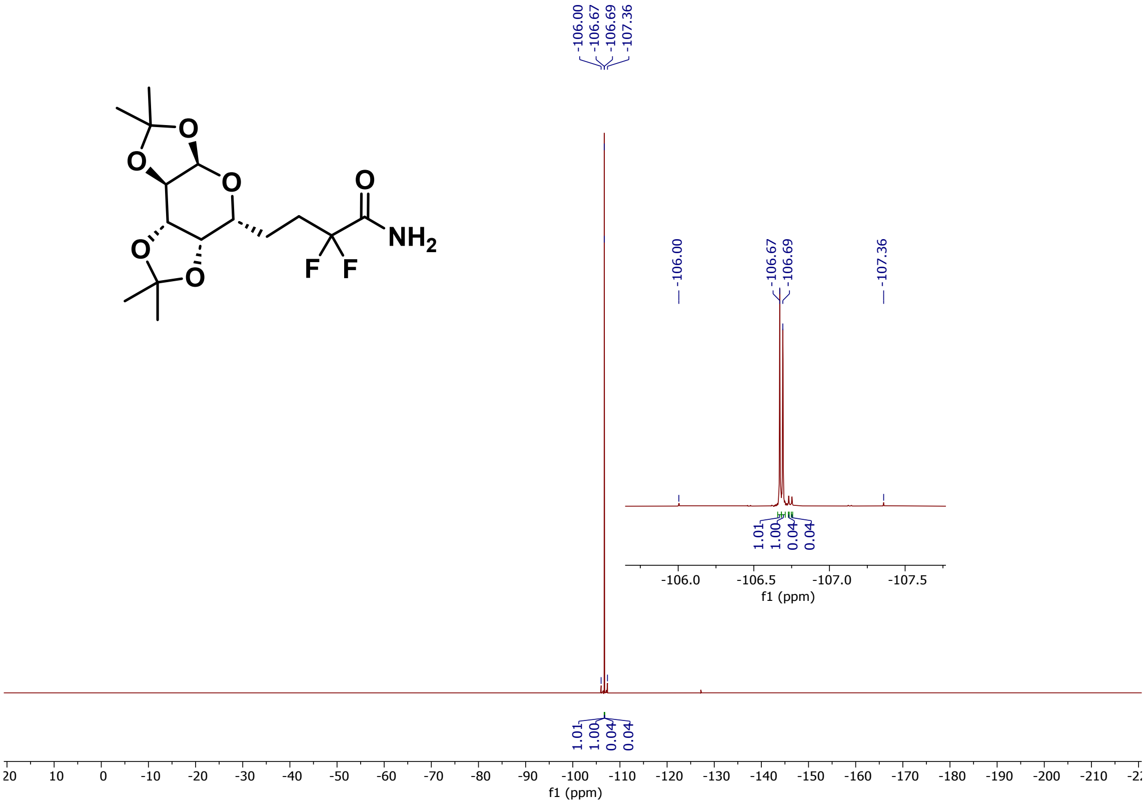

^{19}F NMR (376 MHz, acetone-d$_6$) of compound 5b

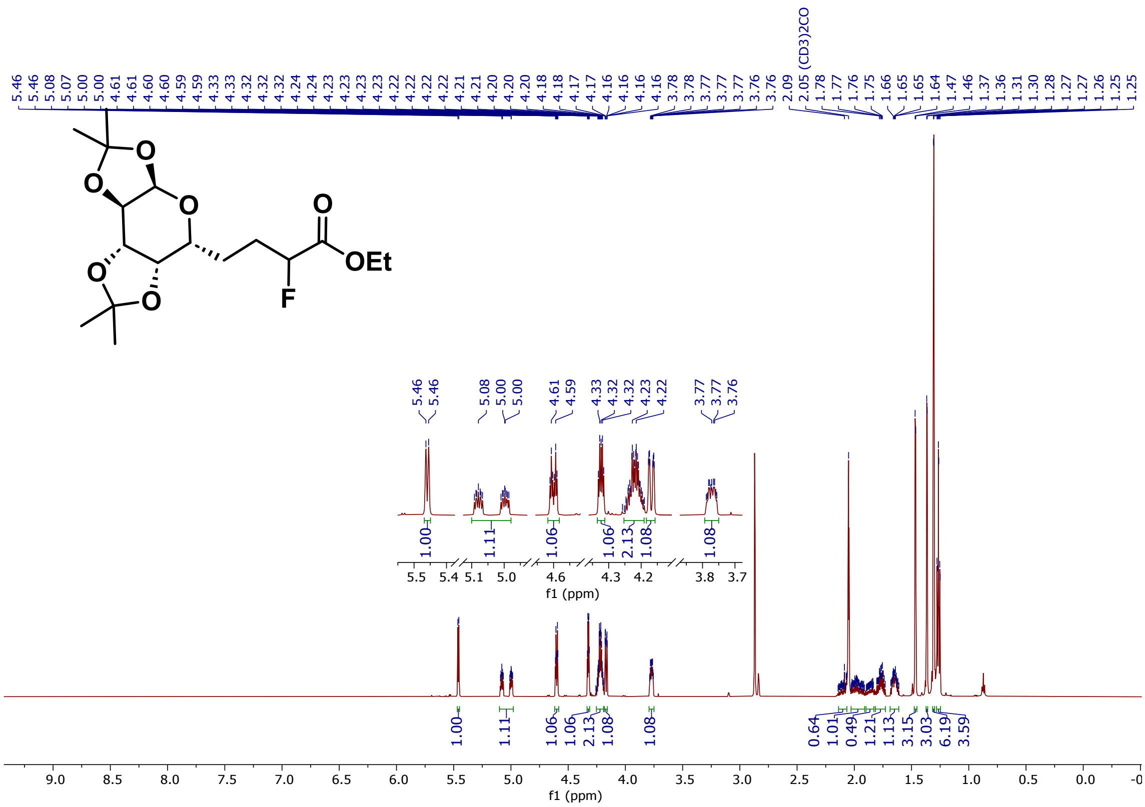

^{1}H NMR (600 MHz, acetone-d$_6$) of compound 5c

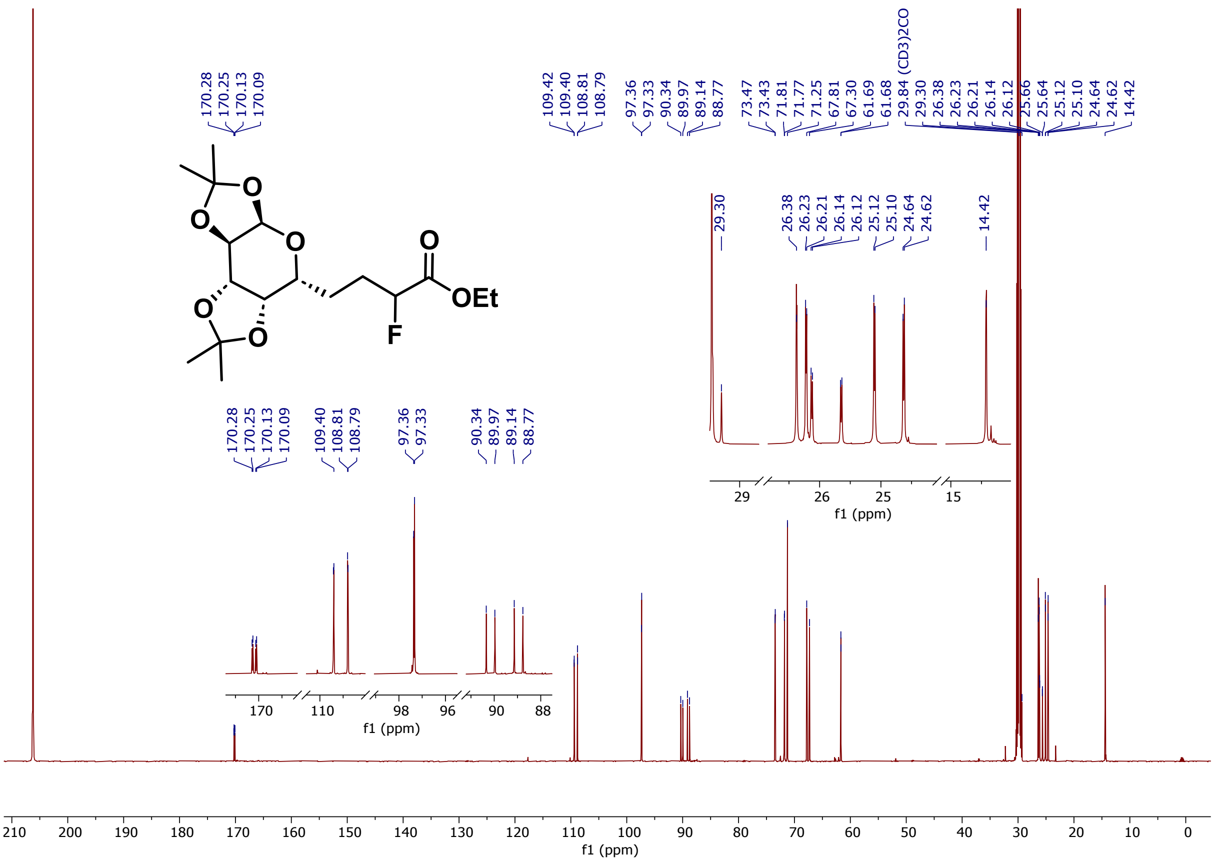

^{13}C NMR (151 MHz, acetone-d$_6$) of compound 5c

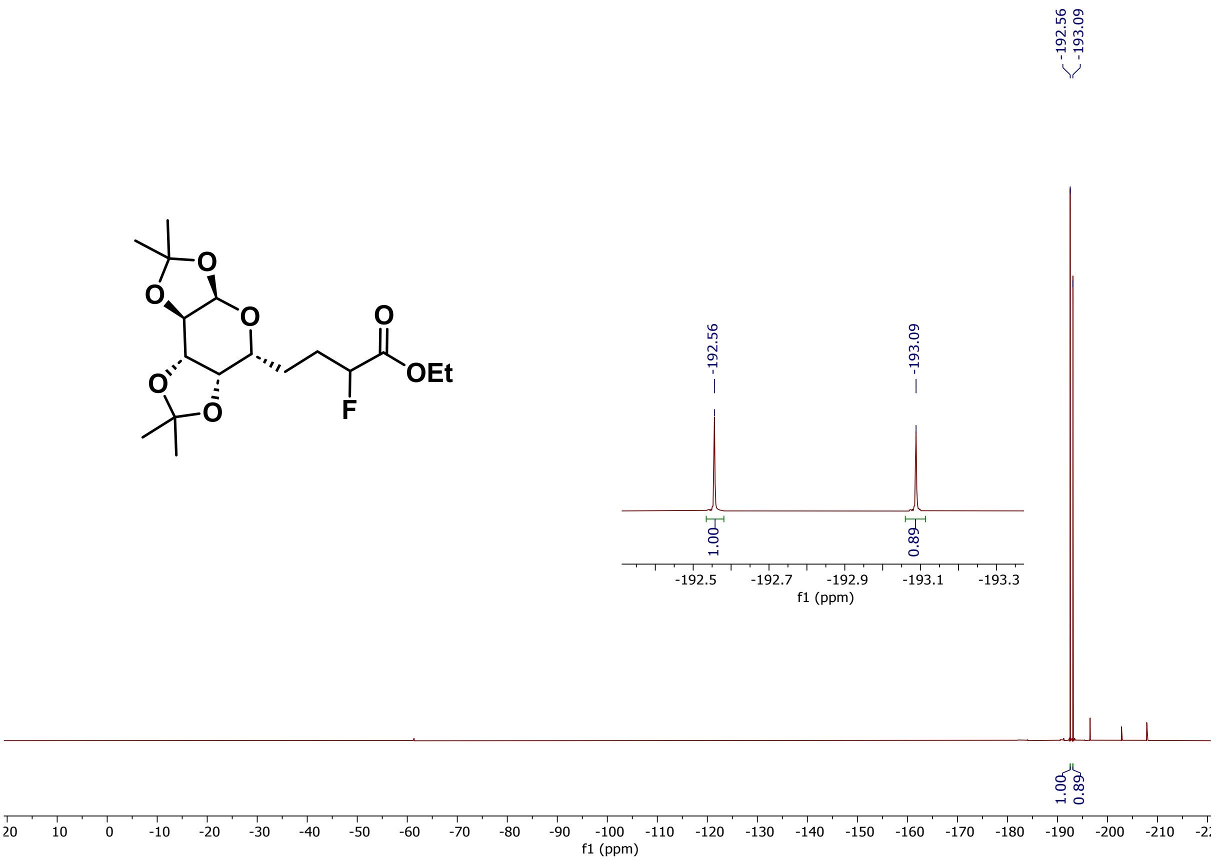

^{19}F NMR (376 MHz, acetone-d$_6$) of compound 5c

17. Characterization Spectra for 1-Bromo-1,1-Difluoroacetamides 2b-d, 2h and 2j-k

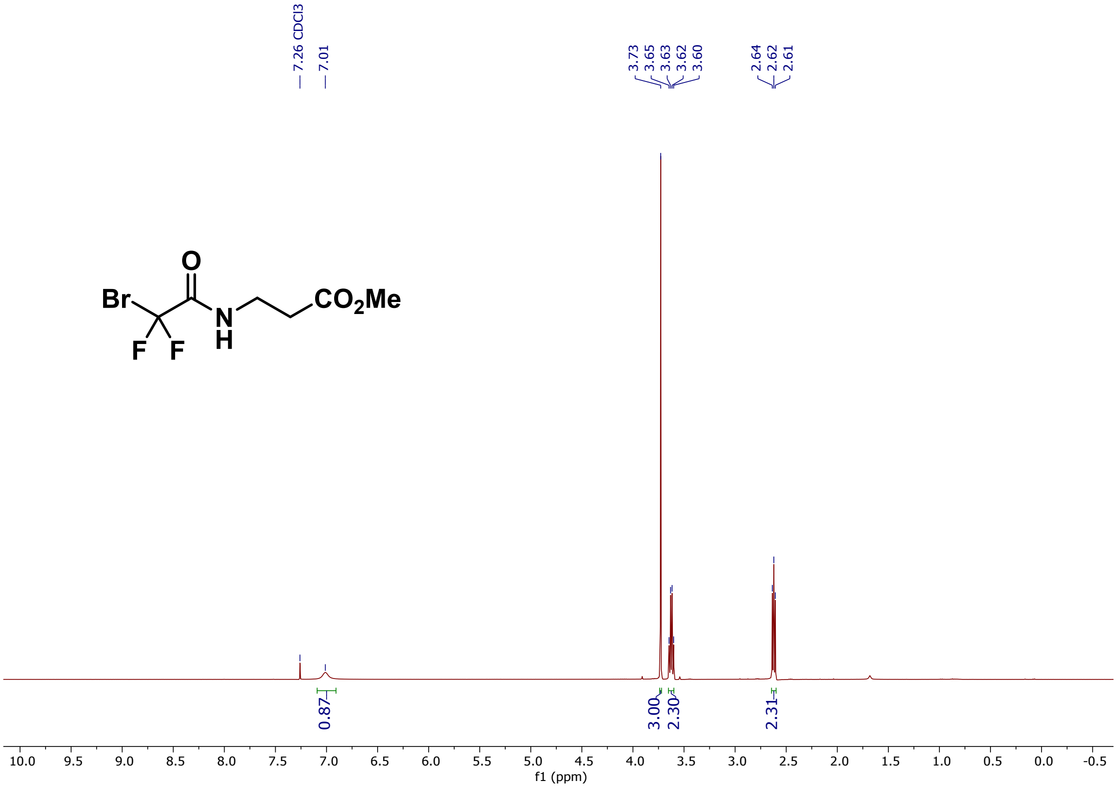

^{1}H NMR (400 MHz, $CDCl_3$) of compound 2b

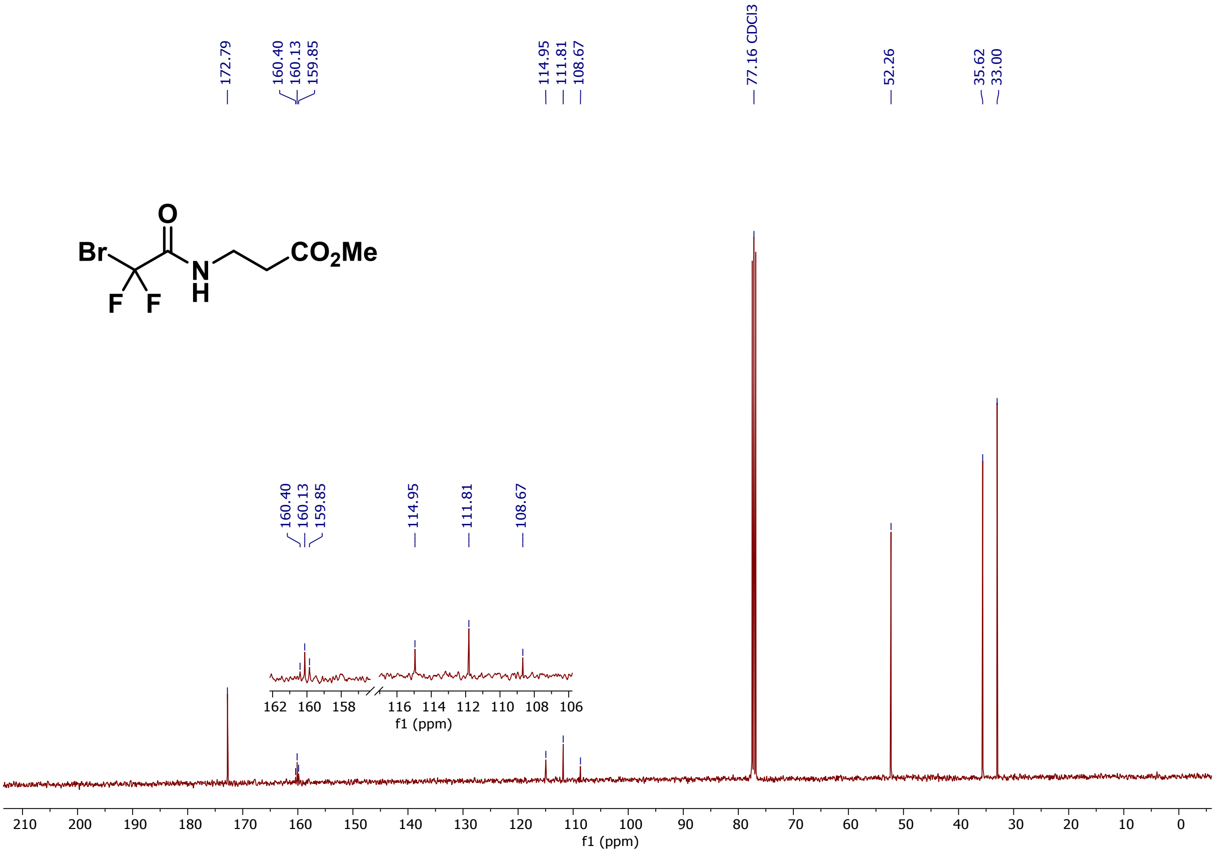

^{13}C NMR (101 MHz, $CDCl_3$) of compound 2b

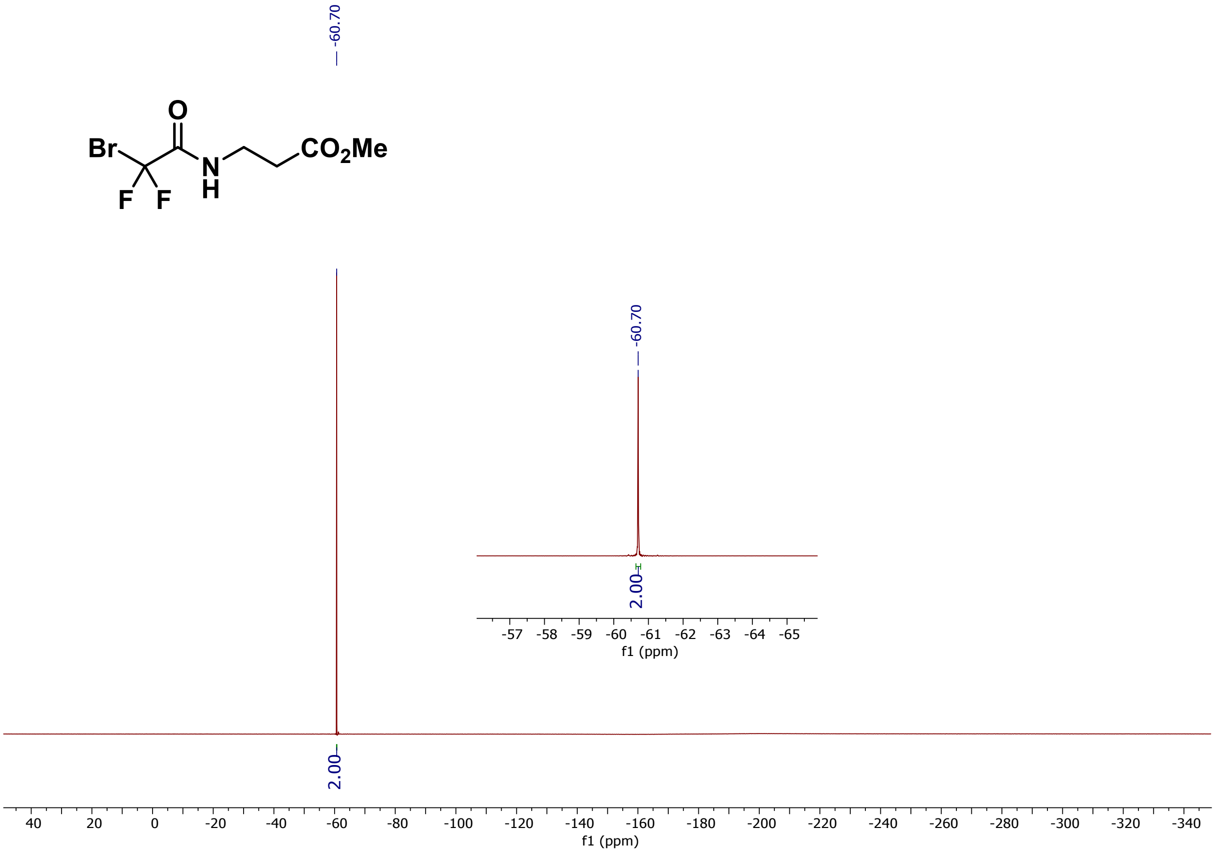

^{19}F NMR (376 MHz, $CDCl_3$) of compound 2b

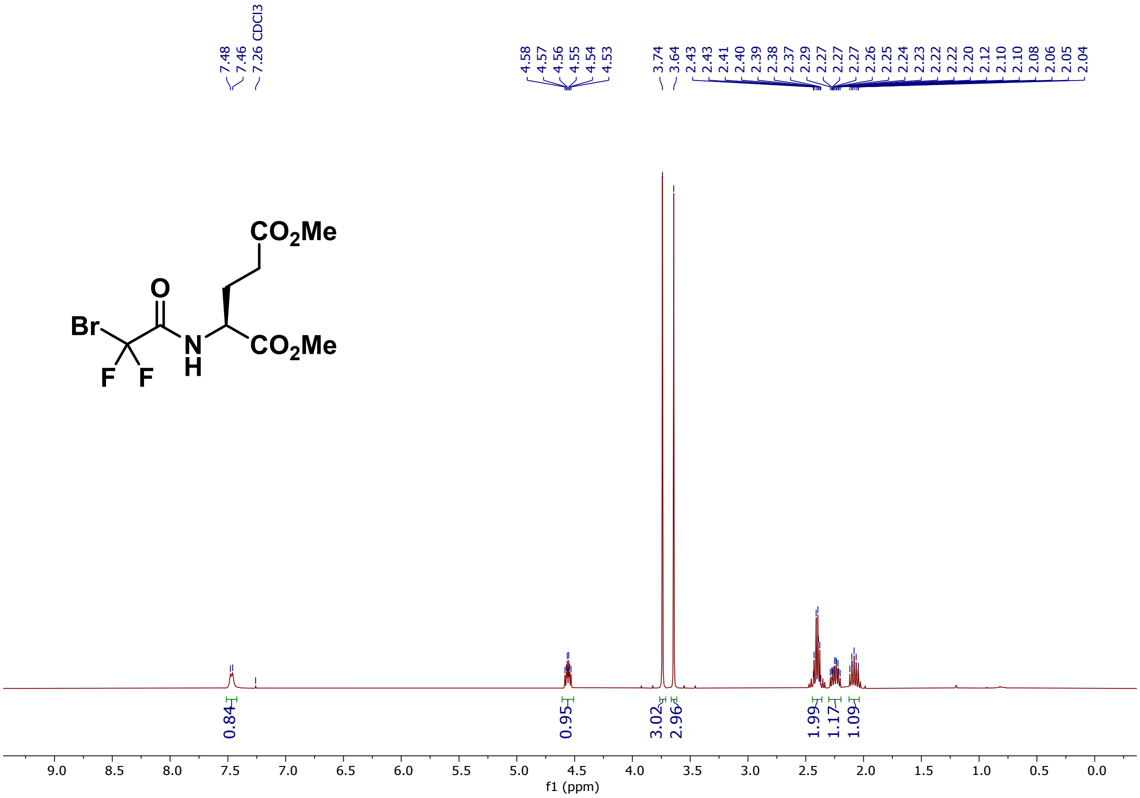

^{1}H NMR (400 MHz, $CDCl_3$) of compound 2c

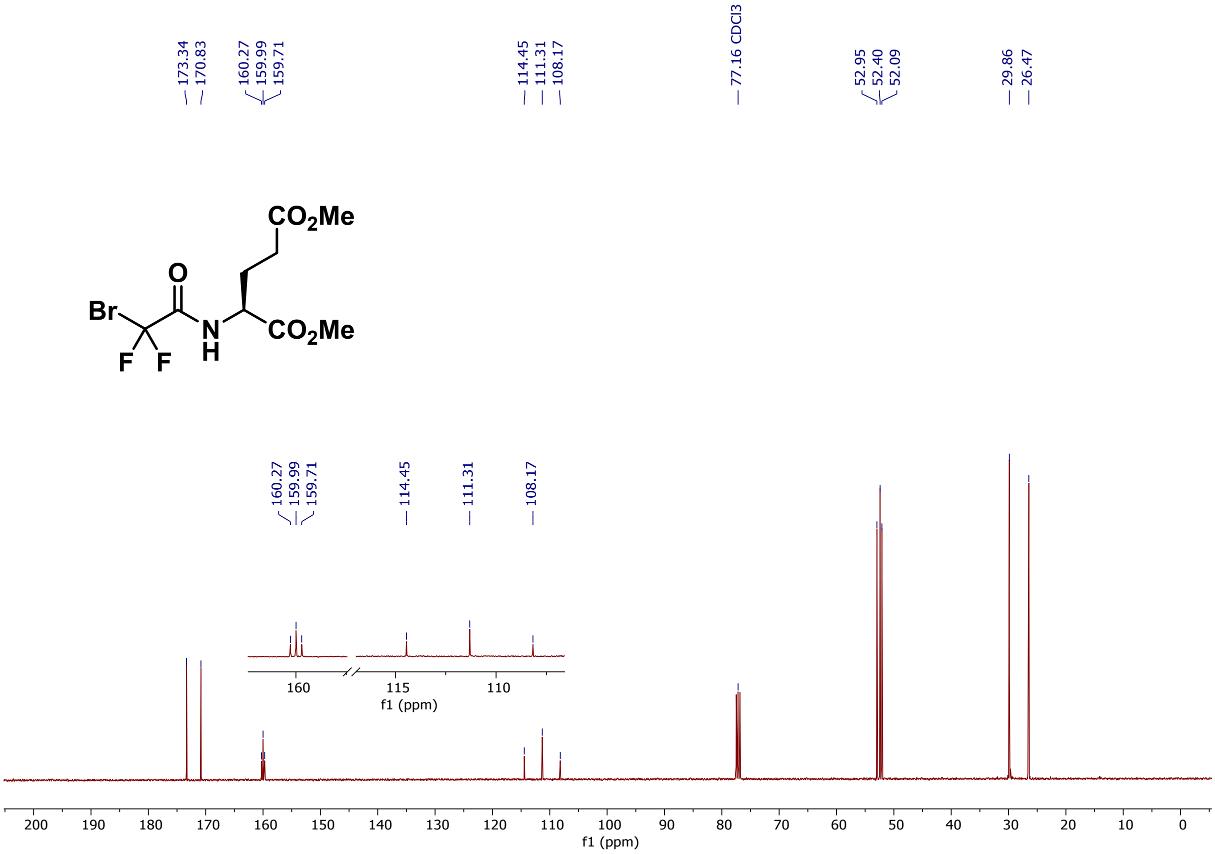

^{13}C NMR (101 MHz, $CDCl_3$) of compound 2c

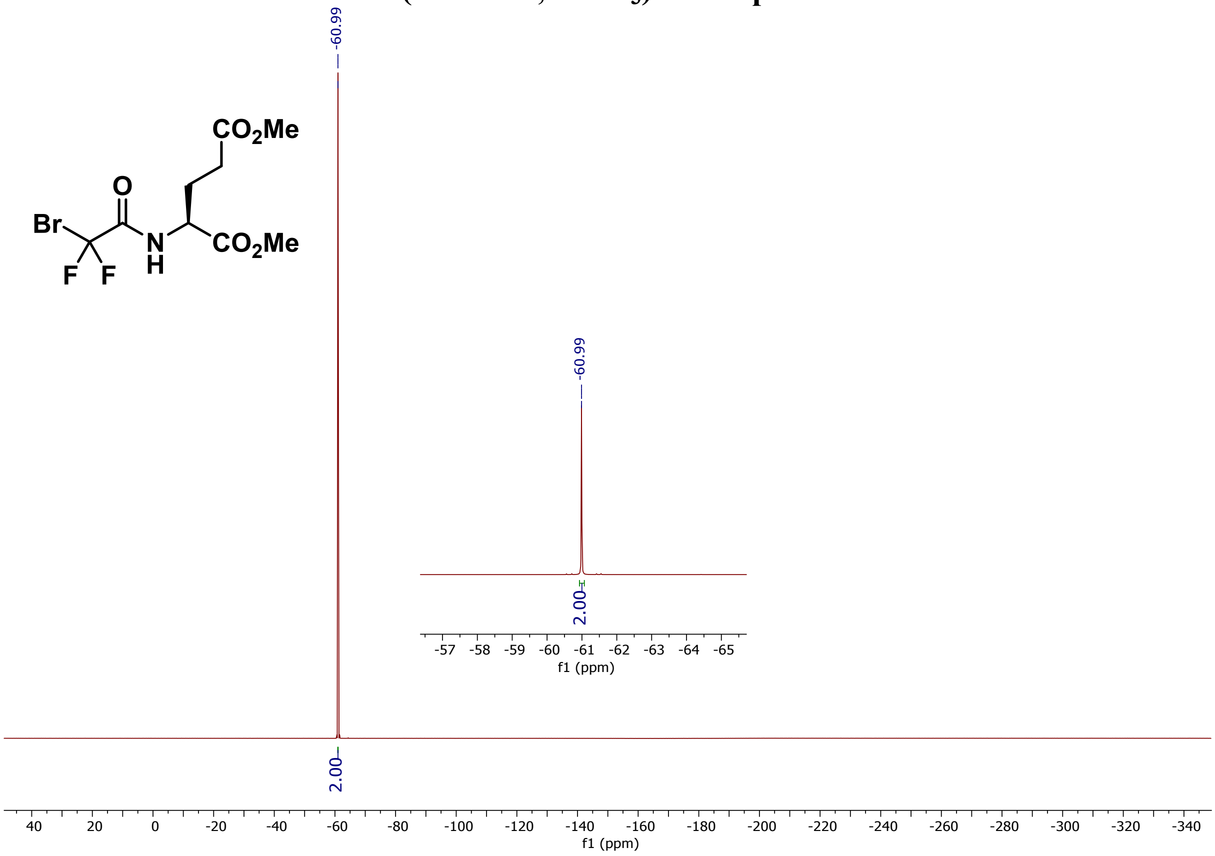

^{19}F NMR (376 MHz, $CDCl_3$) of compound 2c

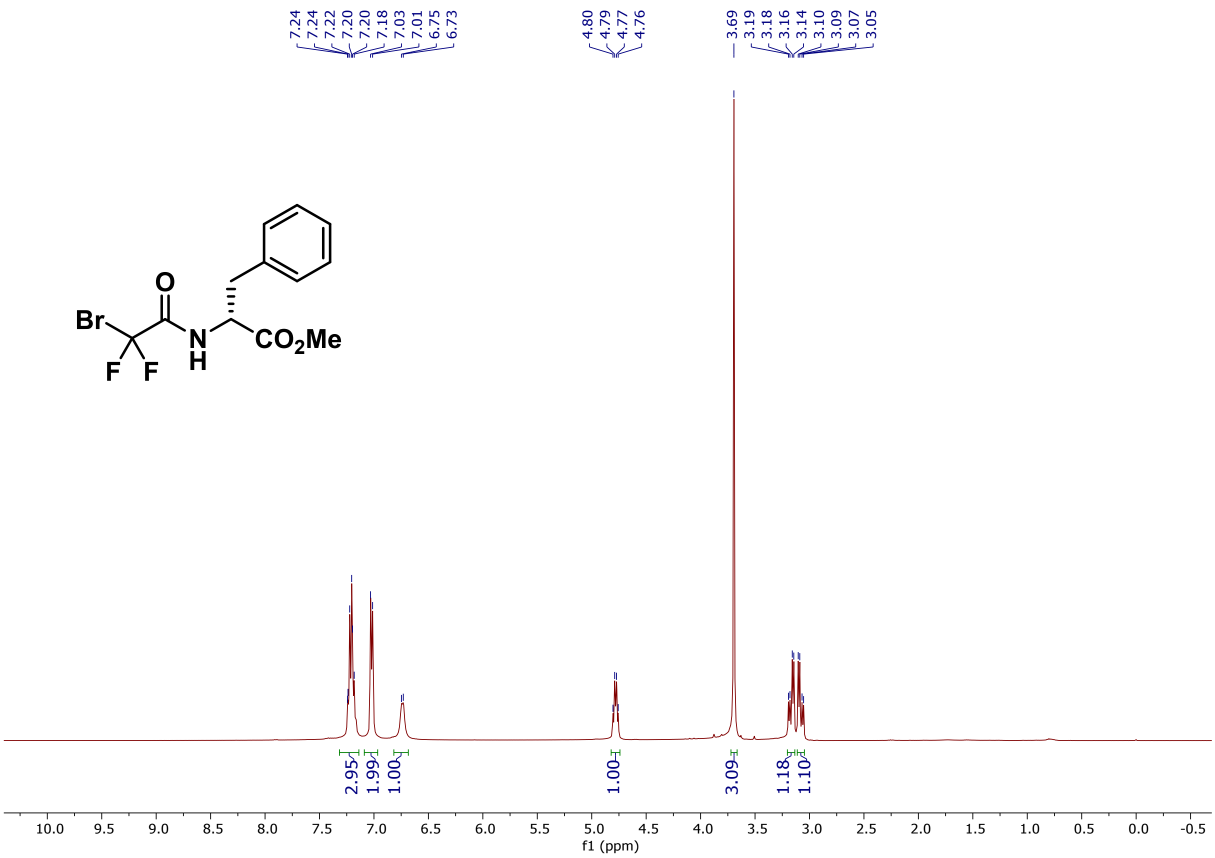

^{1}H NMR (400 MHz, CDCl$_3$) of compound 2g

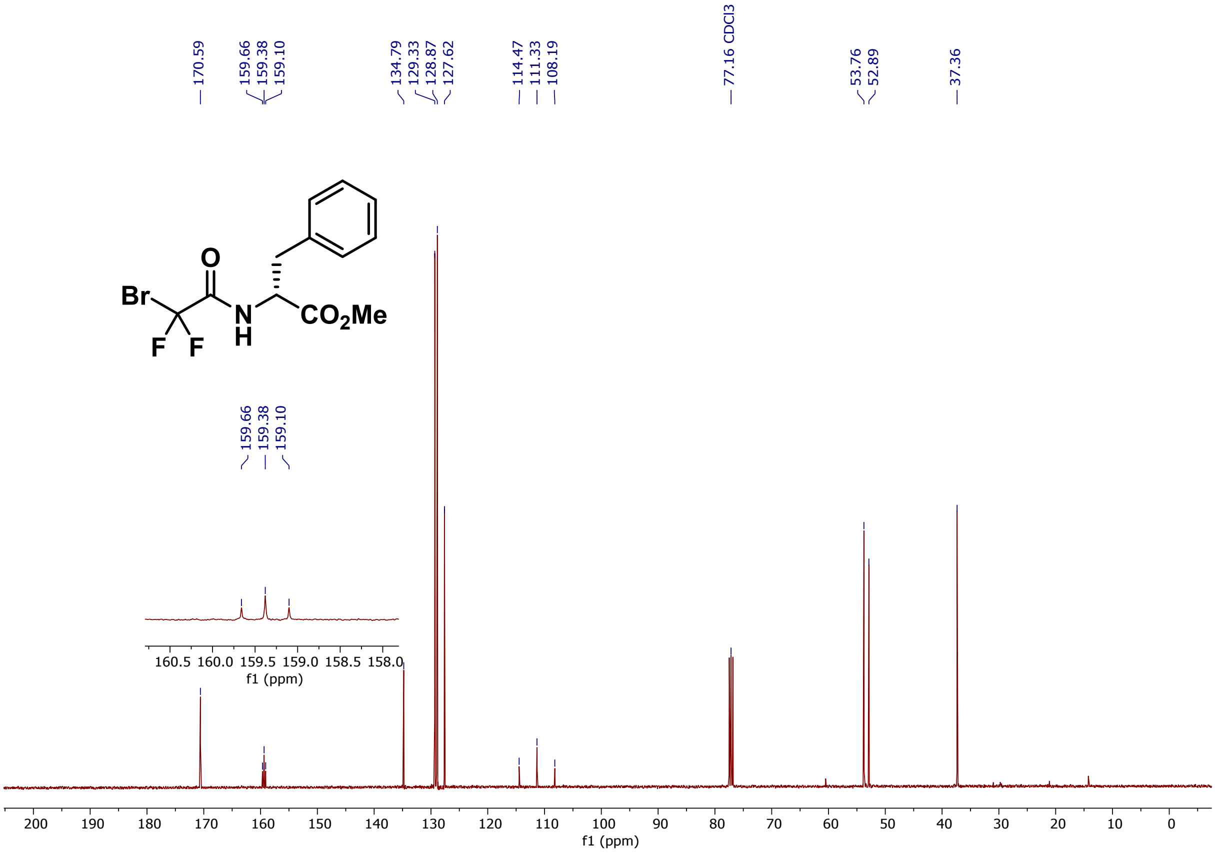

^{13}C NMR (101 MHz, CDCl$_3$) of compound 2g

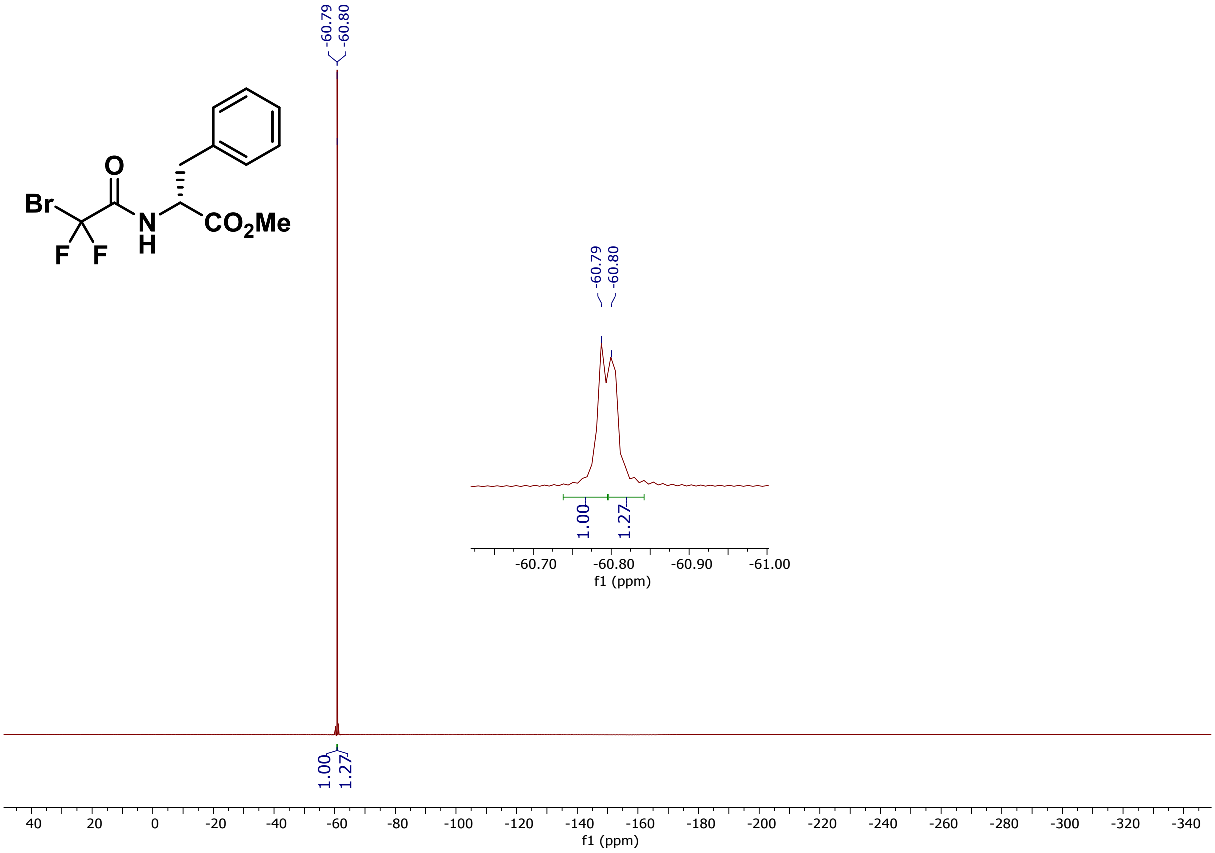

^{19}F NMR (376 MHz, $CDCl_3$) of compound 2g

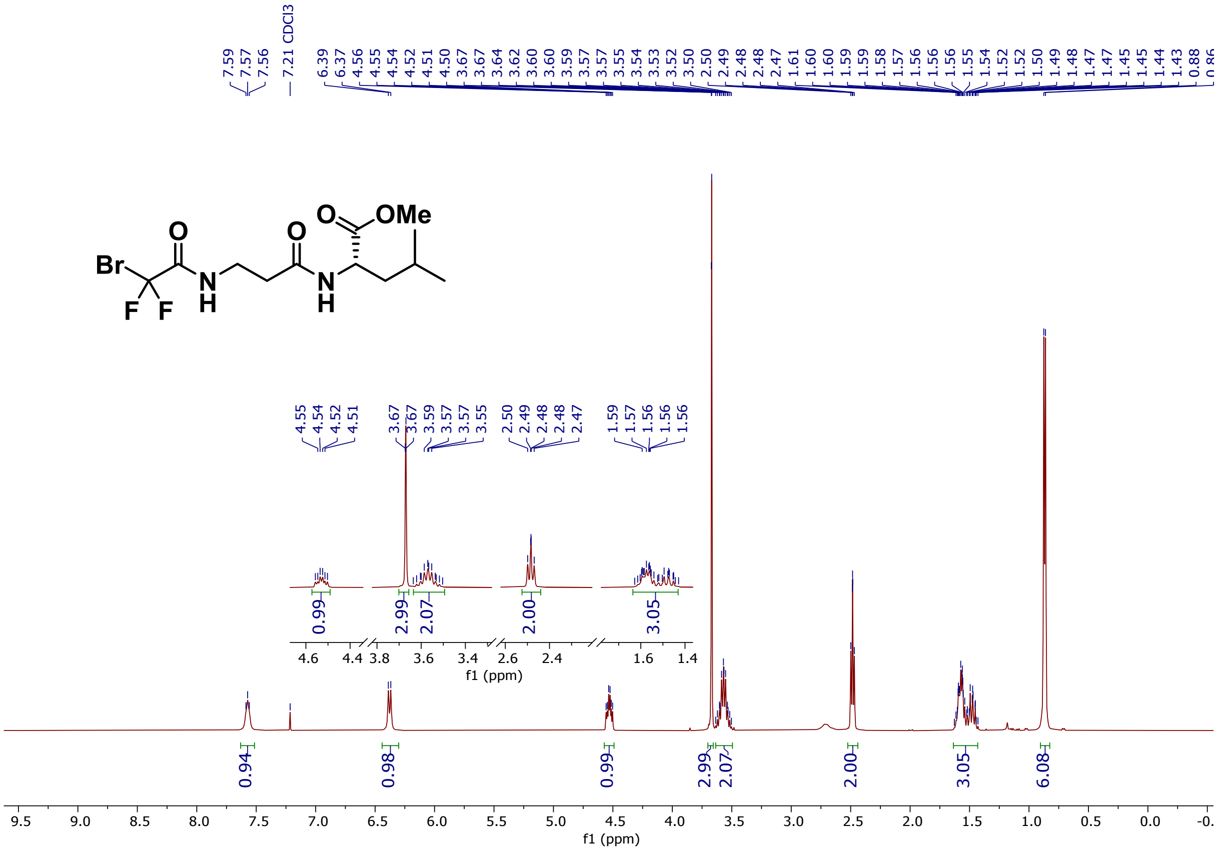

^{1}H NMR (400 MHz, $CDCl_3$) of compound 2j

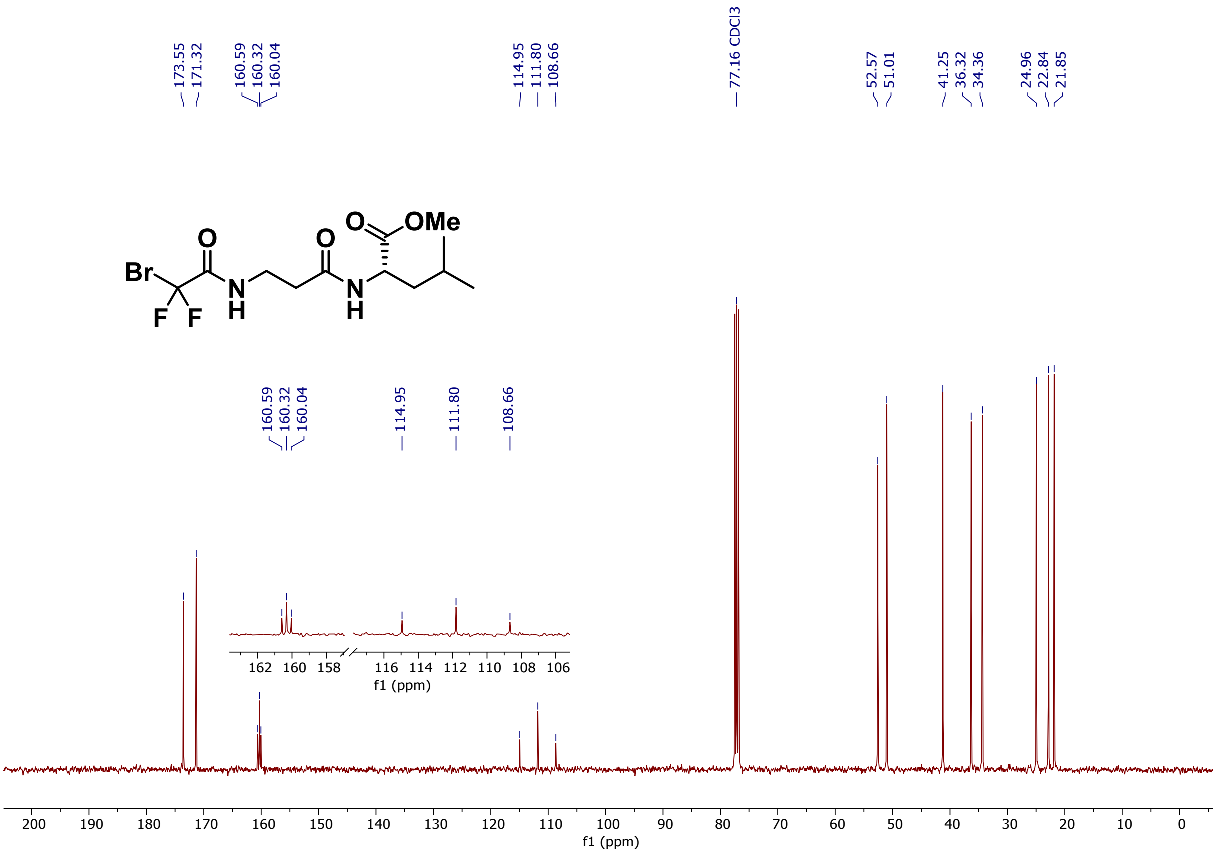

^{13}C NMR (101 MHz, $CDCl_3$) of compound 2j

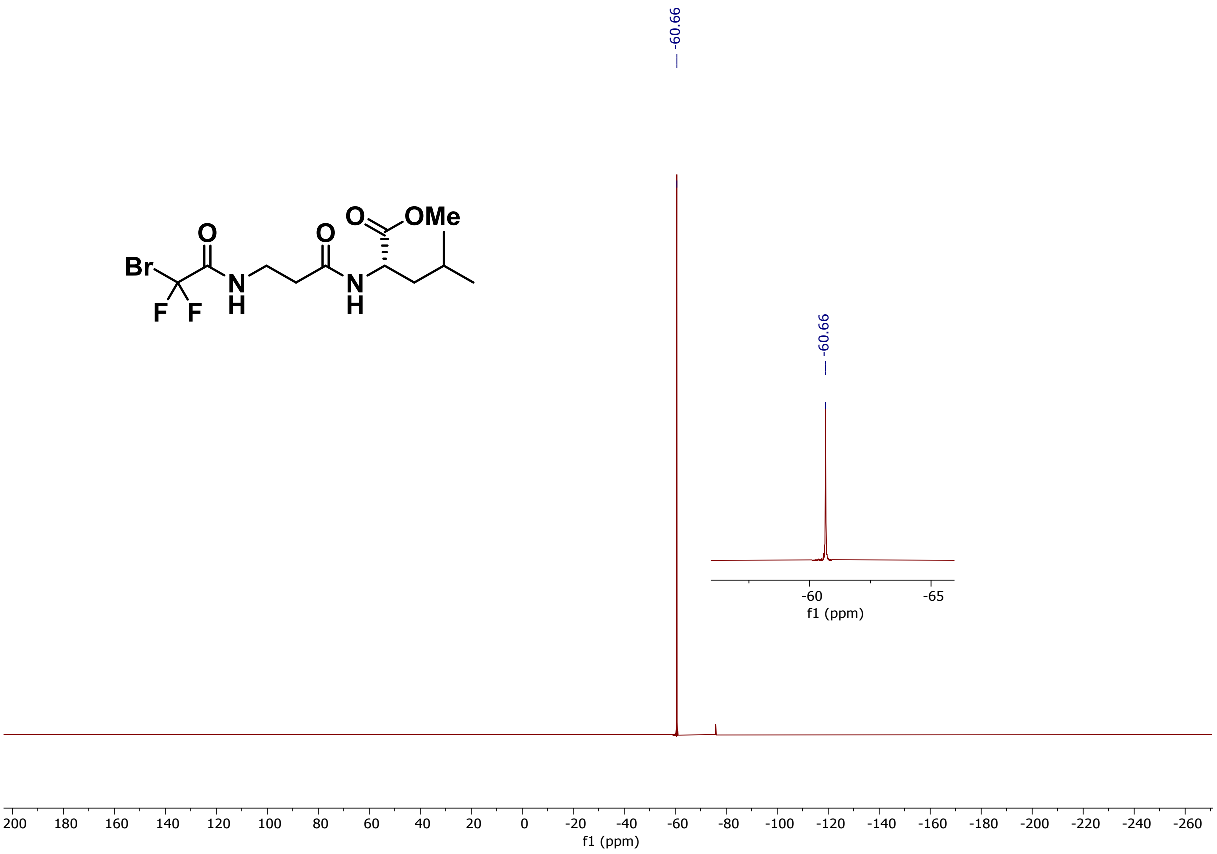

^{19}F NMR (376 MHz, $CDCl_3$) of compound 2j

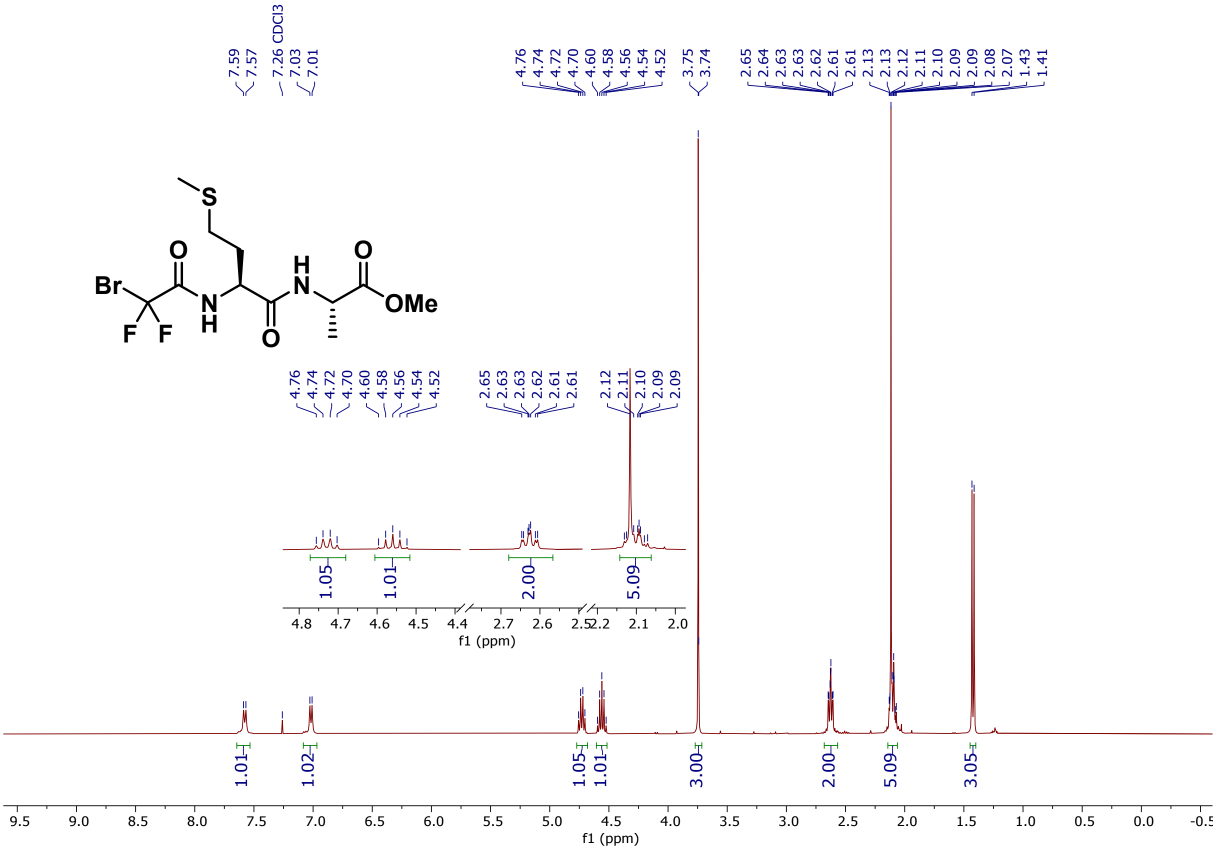

^{1}H NMR (400 MHz, CDCl$_3$) of compound 2k

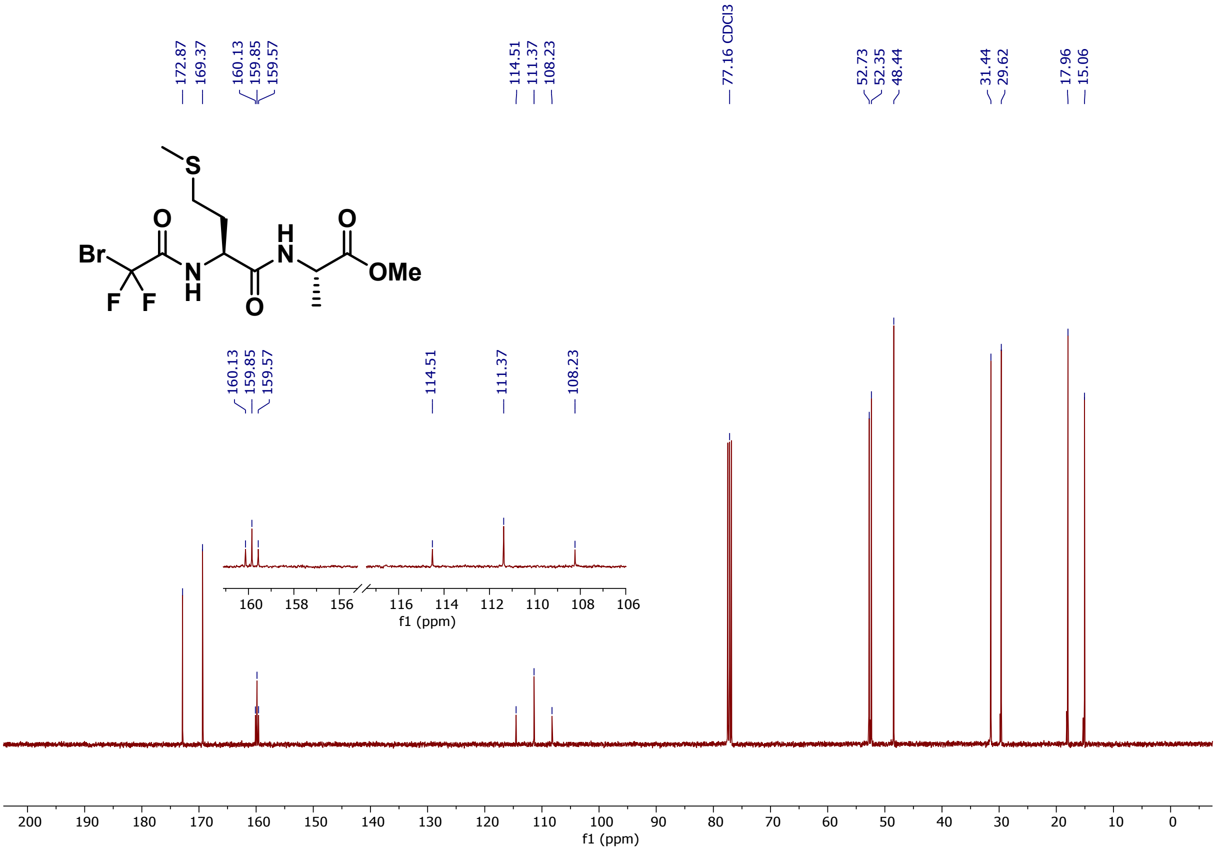

^{13}C NMR (101 MHz, CDCl$_3$) of compound 2k

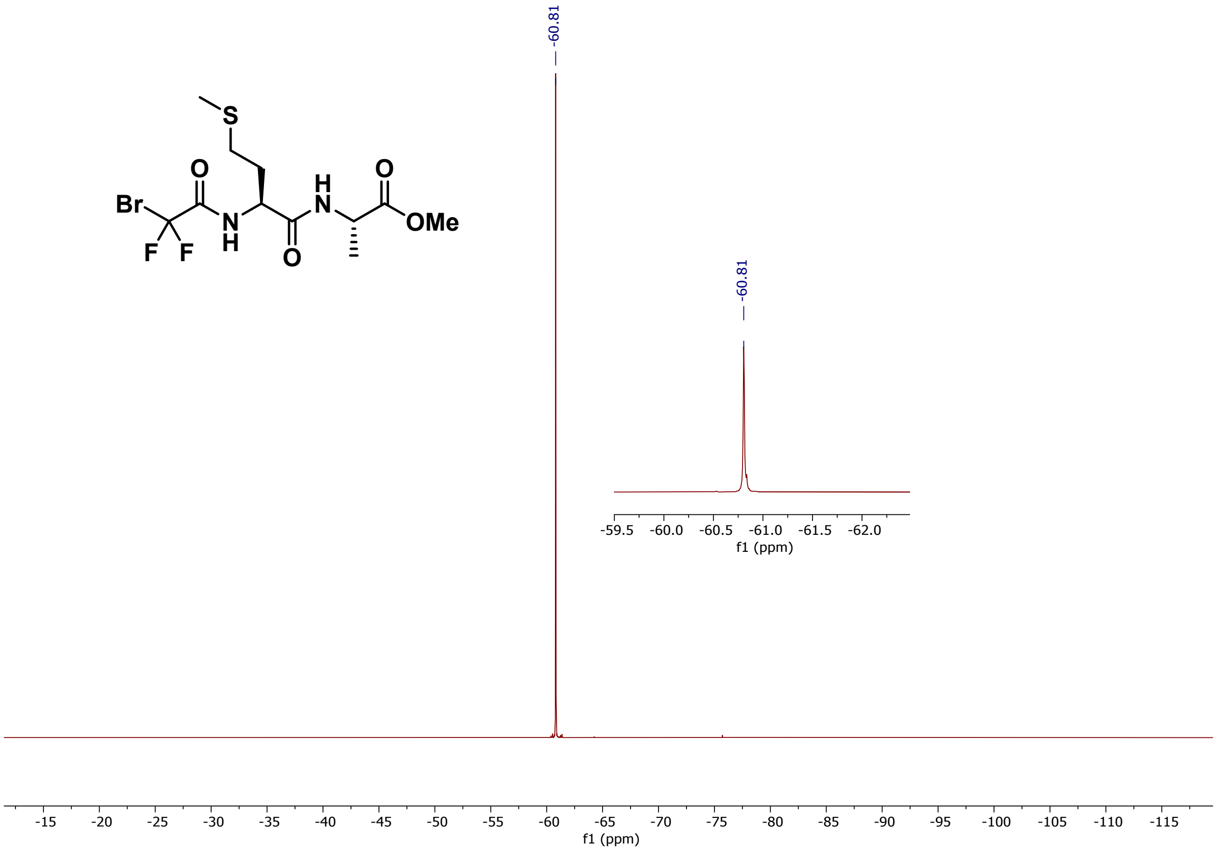

^{19}F NMR (376 MHz, $CDCl_3$) of compound 2k

References

[1] Fulmer, G. R.; Miller, A. J. M.; Sherden, N. H.; Gottlieb, H. E.; Nudelman, A.; Stoltz, B. M.; Bercaw, J. E.; Goldberg, K. I. NMR Chemical Shifts of Trace Impurities: Common Laboratory Solvents, Organics, and Gases in Deuterated Solvents Relevant to the Organometallic Chemist. *Organometallics* **2010**, 29, 2176–2179.

[2] Banks, M. R.; Cadogan, J.I.G.; Gosney, I.; Gould, R. O.; Hodgson, P. K. G.; McDougall, D.
Tetrahedron, **1998**, 54, 9765-9784.

[3] Ogawa, S.; Zhou, B.; Kimoto, Y.; Omura, K.; Kobayashi, A.; Higashi, T.; Mitamura, K.; Ikegawa, S.; Hagey, L. R.; Hofmann, A. F.; Iida, T. *Steroids*, **2013**, 78, 927-937.

[4] Zhang, P.; Hevey, R.;Ling, C.-C. Total Synthesis of β-D-ido-Heptopyranosides Related to Capsular Polysaccharides of *Campylobacter jejuni* HS:4. *J. Org. Chem.* **2017**, 82, 18, 9662–9674.

[5] Reina, J. J.; Rioboo, A.; Montenegro, J. Glycosyl Aldehydes: New Scaffolds for the Synthesis of Neoglycoconjugates via Bioorthogonal Oxime Bond Formation. *Synthesis* **2018**, 50, 831-845

[6] Umeno, K.; Oishi, T. Synthesis and Stereochemistry of the C30−C63 Section of Karlotoxin 2. *Asian J. Org. Chem.* **2020**, 9, 1597-1601.

[7] Lin, Y. A.; Chalker, J. M.; Davis, B. G. Olefin Cross-Metathesis on Proteins: Investigation of Allylic Chalcogen Effects and Guiding Principles in Metathesis Partner Selection. *J. Am. Chem. Soc.* **2010**, 132, 16805–16811.

[8] (a) Sharma, G. V. M.; Yadav, T. A.; Choudhary, M.; Kunwar, A. C. Design of β-Amino Acid with Backbone−Side Chain Interactions:Stabilization of 14/15-Helix in α/β-Peptides. *J. Org. Chem.* **2012**, 77, 6834−6848. (b) Charrier, N.; Quiclet-Sire, B.; Zard, S. Z. Allylic Alcohols as Radical Allylating Agents. An Overall Olefination of Aldehydes and Ketones. *J. Am. Chem. Soc.* **2008**, 130, 28, 8898–8899.
[9] Zhao, G.-D.; Liu, Z.-P. Structural revisions of the reported A-ring phosphine oxide synthon for ED-71 (Eldecalcitol) and a new synthesis. *Tetrahedron* **2015**, 71, 8033-8040.
[10] (a) For the protection step see: Klepper, F.; Jahn, E.-M.; Hickmann, V.; Carell, T. Synthesis of the Transfer-RNA Nucleoside Queuosine by Using aChiral Allyl Azide Intermediate. *Angew. Chem. Int. Ed.* **2007**, 46, 2325 –2327. (b) For oxidation step and Wittig reaction see ref. 2 and 3.
[11] Mamone, M.; Gonçalves, R. S. B.; Blanchard, F.; Bernadat, G.; Ongeri, S.; Milcent, T.; Crousse, B. *N*-difluoromethyl triazole as scaffold in peptidomimetic. *Chem. Commun.* **2017**, *53*, 5024.
[12] Xu, C.; Cheng, R.; Luo, Y.-C.; Wang, M.-K.; Zhang, X. trans-Selective Aryldifluoroalkylation of Endocyclic Enecarbamates and Enamides by Nickel Catalysis. *Angew. Chem. Int. Ed.* **2020**, *59*, 18741.
[13] Rao, N.; Li, Y.-Z.; Luo, Y.-C.; Zhang, Y.; Zhang, X. Nickel-Catalyzed Multicomponent Carbodifluoroalkylation of Electron-Deficient Alkenes. *ACS Catalysis* **2023**, *13*, 4111.
[14] Tarui, A.; Shinohara, S.; Sato, K.; Omote, M.; Ando, A. Nickel-Catalyzed Negishi Cross-Coupling of Bromodifluoroacetamides. *Org. Lett.* **2016** 18, 1128-1131.
[15] Chandu, P.; Srinivasu, V.; Guptaand, S.; Sureshkumar, D. Photoinduced Cascade Difluoroalkylative Ring-Opening of Vinyl Cyclopropanes. *Org. Lett.* **2023**, *25*, 2857.
